# Revealing the characteristics of ZIKV infection through tissue-specific transcriptome sequencing analysis

**DOI:** 10.1186/s12864-022-08919-5

**Published:** 2022-10-08

**Authors:** Zhi-lu Chen, Zuo-jing Yin, Tian-yi Qiu, Jian Chen, Jian Liu, Xiao-yan Zhang, Jian-qing Xu

**Affiliations:** 1 Institutes of Biomedical Sciences, Shanghai Medical College, Fudan University, Shanghai, 200032 China; 2 Shanghai Public Health Clinical Center, Fudan University, Shanghai, 201508 China; 3 Department of Immunotherapy and Shanghai Key Laboratory of Organ Transplantation, Zhongshan Hospital, Fudan University, Shanghai, 200032 People’s Republic of China

**Keywords:** Zika virus, Tissue-specific, Transcriptome sequencing analysis, Host immune responses, Type I interferon

## Abstract

**Background:**

Recently, Zika virus (ZIKV) re-emerged in India and was potentially associated with microcephaly. However, the molecular mechanisms underlying ZIKV pathogenesis remain to be explored.

**Results:**

Herein, we performed a comprehensive RNA-sequencing analysis on ZIKV-infected JEG-3, U-251 MG, and HK-2 cells versus corresponding uninfected controls. Combined with a series of functional analyses, including gene annotation, pathway enrichment, and protein–protein interaction (PPI) network analysis, we defined the molecular characteristics induced by ZIKV infection in different tissues and invasion time points. Data showed that ZIKV infection and replication in each susceptible organ commonly stimulated interferon production and down-regulated metabolic-related processes. Also, tissue-specific immune responses or biological processes (BPs) were induced after ZIKV infection, including GnRH signaling pathway in JEG-3 cells, MAPK signaling pathway in U-251 MG cells, and PPAR signaling pathway in HK-2 cells. Of note, ZIKV infection induced delayed antiviral interferon responses in the placenta-derived cell lines, which potentially explains the molecular mechanism by which ZIKV replicates rapidly in the placenta and subsequential vertical transmission occurs.

**Conclusions:**

Together, these data may provide a systemic insight into the pathogenesis of ZIKV infection in distinct human tissue-derived cell lines, which is likely to help develop prophylactic and therapeutic strategies against ZIKV infection.

**Supplementary Information:**

The online version contains supplementary material available at 10.1186/s12864-022-08919-5.

## Background

ZIKV is a single-stranded positive-sense RNA Flavivirus [1] that is primarily transmitted through the Aedes mosquitos [2]. It was first isolated in 1947 from a febrile rhesus macaque caged in Zika forest canopy in Uganda [3]. ZIKV is related to other human pathogens transmitted by arthropods including dengue virus (DENV), yellow fever virus (YFV), West Nile virus (WNV), Japanese encephalitis virus (JEV), and tick-borne encephalitis virus (TBEV) [2]. During the past decades, ZIKV has re-emerged from a relatively unknown status to causing massive epidemics in French Polynesia, South, and Central America. Although ZIKV infection causes mild fevers with rash and conjunctivitis in most cases, severe neurological phenotypes can occur including Guillain–Barre syndrome and microcephaly [4–6]. 

Notably, ZIKV exhibits a broad-spectrum tropism and persistence in body tissues and fluids, which contributes to the clinical manifestations and epidemiology observed during the epidemic [7]. In vitro studies have demonstrated that human neural progenitor cells, cerebral organoids, astrocytes, placental cells, proximal renal tubular epithelial cells, and peripheral blood mononuclear cells are susceptible to ZIKV infection [8–14]. Considerable efforts have been made to investigate the pathogenic features and molecular mechanisms of ZIKV infection in human cells through RNA-sequencing (RNA-seq) technology [15]. In the previous studies, the transcription of Toll-like receptor 3 (TLR3), retinoic acid-inducible gene I (RIG-I), and melanoma differentiation-associated gene 5 (MDA5), as well as several interferon-stimulated genes including OAS2, ISG15, and MX1, were strongly increased in human fibroblasts after ZIKV infection [16]. While ZIKV infection in human neural progenitor cells (hNPCs) [10] particularly inhibited gene expression in cell-cycle-related pathways, ZIKV-upregulated genes were primarily enriched in the transcription, protein transport, and catabolic processes including caspase-3, which were involved in the regulation of the apoptotic pathway.

In addition, ZIKV antigen was found in the chronic villi of a human placenta from a mother who gave birth to an infant with microcephaly [17], and ZIKV RNA has been isolated from the placental tissue of a mother diagnosed with ZIKV disease [18]. Vertical transmission of ZIKV from an infected mother to the developing fetus in utero reflects tropism for placental associated cells, such as placental macrophages, which are also known as Hofbauer cells (HCs) [19, 20]. Analysis of antiviral gene expression shows that type I interferon (IFN) signaling pathway, including RIG-I-like receptor (RLR) transcription as well as downstream antiviral effector genes, was up-regulated in HCs after ZIKV infection (24 and 48 h post-infection, the late time point) [19]. This delayed antiviral immune response may provide a window for drastic ZIKV replication.

Prior findings partially explained the high efficiency and pathogenicity of ZIKV infection in human placentae and developing fetal brains. However, the consequences of ZIKV infections in other human organs or tissue cells and the difference of host response induced by ZIKV infection between these susceptible cells remain elusive. More importantly, detailed analyses of the ZIKV infection-induced gene network disruption, the tissue-specific immune responses or associated BPs, and the relationships between regulated signaling pathways are still unclear. Therefore, a systematical investigation of BPs affected by ZIKV infection in various human organ samples could be necessary to identify the candidate genes for pharmaceutical intervention and potential biomarkers for diagnosis.

Previous studies have reported that human choriocarcinoma cells (JEG-3) [14], astrocytes (U-251 MG) [13], and human renal proximal tubular epithelial cells (HK-2) [21] can support ZIKV infection with higher efficiency and observable cytopathic effects. In this study, a comprehensive RNA-seq analysis was performed to investigate the effects of ZIKV infection on multiple human organs. Moreover, functional pathway enrichment analyses of Differentially Expressed Genes (DEGs) were executed to detect the best candidate signaling pathway associated with ZIKV infection by comparing the RNA-seq data from the above three cell lines. Subsequent analysis was focused on the placental infection data from indicated timepoints of ZIKV infection in human placental cells and revealed that delayed INF induction is likely to respond to enhanced ZIKV replication in human placentae.

## Results

### ZIKV infection induced distinct immune responses in diverse tissues-derived cell lines

**Fig. 1:**
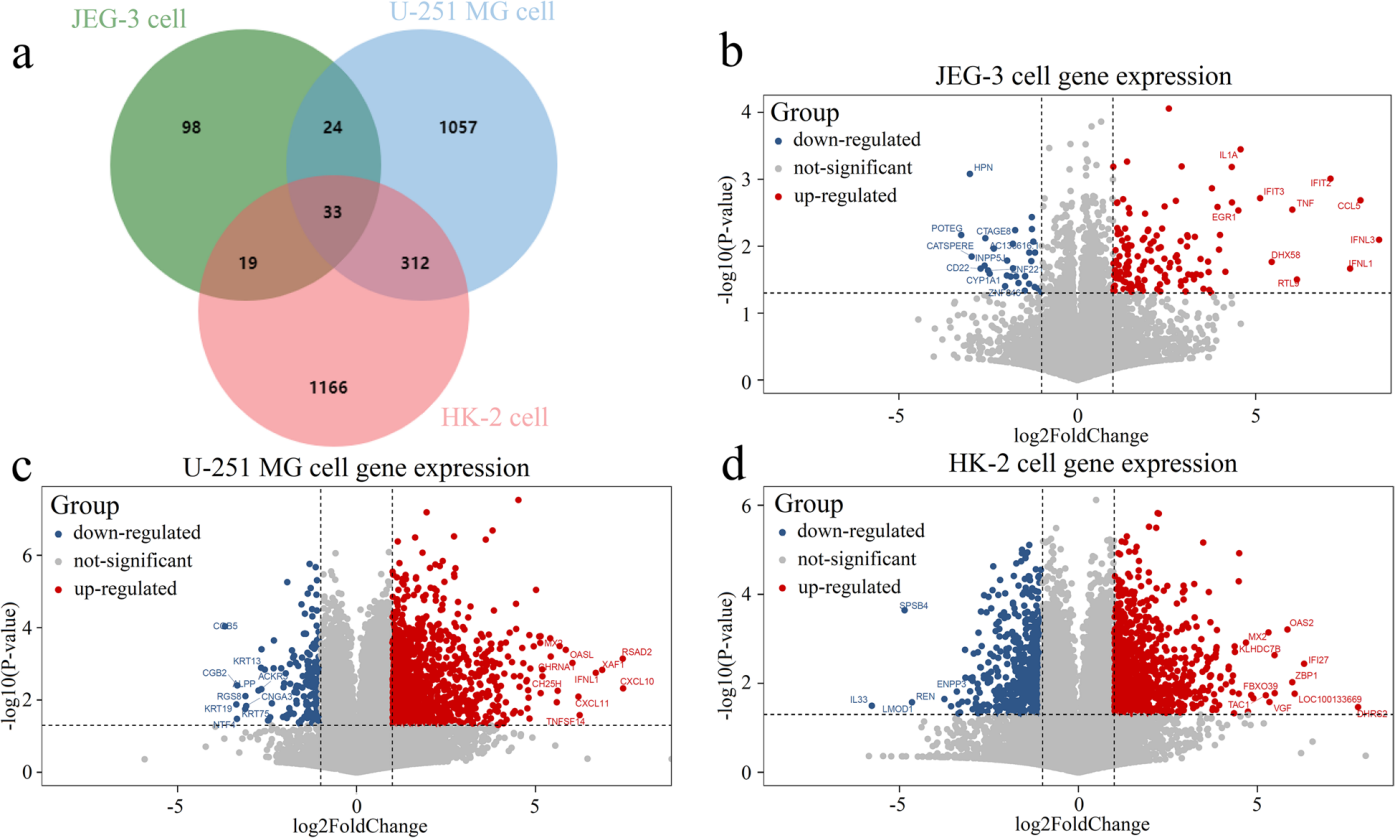
DEGs stimulated by ZIKV for 24 h. **a** DEGs in placental cells (JEG-3), nerve cells (U-251 MG), and kidney cells (HK-2). **b** DEGs stimulated by ZIKV for 24 h in JEG-3. **c** DEGs stimulated by ZIKV for 24 h in U-251 MG. **d** DEGs stimulated by ZIKV for 24 h in HK-2. Red and blue dots correspond to up-regulated and down-regulated genes, respectively. The top 10 up-regulated and down-regulated genes were determined by FC value and marked in the figures. Several labeled genes are not shown in the figures due to their overlap with other genes. JEG-3, U-251 MG, and HK-2 cells were infected with ZIKV African strain MR766 (MOI = 1). The control samples were the corresponding cells without ZIKV infections. The two-tailed student’s t-test was used in the detection of the DEGs between corresponding infected cells and uninfected cells. Each experiment was repeated for 3 times

Here, transcriptome datasets on three cell lines from multiple human tissues, including human placental choriocarcinoma cells (JEG-3), human glioblastoma cells (U-251 MG), and human renal proximal tubular epithelial cells (HK-2), were obtained from RNA-sequencing, which recorded the gene expression of uninfected controls and ZIKV-infected cells. DEGs in corresponding cell lines at 24 h.p.i. were identified by comparing with the basic transcriptome of controls, which were not infected with ZIKV. The comparison using T-test and Fold Change (FC) with defaulted thresholds (see Materials and Methods) yields a total of 142 up-regulated genes and 32 down-regulated genes in JEG-3 cell line. Besides, there were 1,261 up-regulated and 165 down-regulated genes in U-251 MG cells, while 892 genes were found to be up-regulated and 638 down-regulated in HK-2 cells (Fig. 1a). Detailed information of DEGs in JEG-3, U-251 MG and HK-2 cell lines is listed in Supplementary Tables 1, 2 and 3. All 2,973 DEGs in these three cell lines showed generally high classification performance, especially in U-251 MG and HK-2 cell lines, indicating tissue-specificity patterns of expression as well as divergent functions (Supplementary Fig. 1). We observed that only 33 DEGs were overlapped in all three cell lines (Fig. 1a), most of which were associated with antiviral functions, such as activation of the complement system (C3), inhibition the viral replication (IFIT1, IFIT5, ISG20, OAS1, OAS3, and RSAD2), regulation of antiviral innate immune response (DDX58, IFIH1), participation in T-cell activation pathway (HSH2D), regulation of type I interferon production (DHX58), and other antiviral activity (DDX60, DDX60L, IFIT2, IFIT3, and ISG15). Information of the gene names and corresponding functions summarized in NCBI database [22] are described in detail within Supplementary Table 4. Also, Supplementary Fig. 2 showed that, these 33 common DEGs displayed distinct expression patterns between the uninfected and infected samples in each cell line.

The above results showed that most of the common genes among all organs were related to general antiviral responses. Besides, some of them could also be detected in other viral responses. For instance, RSAD2 was found to inhibit several viruses including influenza virus and HIV-1, and DDX60L could inhibit hepatitis C virus replication in response to interferon stimulation in cell culture. Conversely, tissue-specific DEGs indicate the different host responses to ZIVK infections in multiple organs or tissues.

It was also revealed that ZIKV infection stimulated fewer DEGs in JEG-3 cells (Fig. 1b) than those in U-251 MG cells (Fig. 1c) and HK-2 cells (Fig. 1d), either up-regulated (red dot) or down-regulated (blue dot) ones. The top 10 up- and down-regulated genes in 3 cell lines are listed in Table 1. Among them, IFNL1, IFNL3, RSAD2, ZBP1, and OAS2 were related to innate immune responses. CCL5 and TNFSF14 were associated with T-cell mediated cell immunity, with the former encoded one of the major HIV-suppressive factors produced by CD8 + T-cells [23], whose upregulation induced human placental damage and excessive inflammation after ZIKV infection [24, 25], while the latter encoded a ligand for TNFRSF14/HVEM, co-stimulating T cell proliferation [26]. Also, it’s reported that the deletion of TNFSF14 may correlate with a decrease in IFN-γ-producing CD4 + T cells, which mediated immunopathogenesis of ocular Herpes simplex virus 1 (HSV-1) infection as the Herpesvirus entry mediator (HVEM) binding partner [27]. Besides, CXCL10 and CXCL11 bonded the receptor CXCR3 and exerted a potent chemotactic effect on activated T lymphocytes [28–30]. Additionally, CH25H was induced in response to ZIKV infection, and its enzymatic product 25HC was a critical mediator of host protection against ZIKV, which had also been characterized as a broad-spectrum antiviral drug that inhibited viruses including ZIKV [31–33].

**Table 1:** The top 10 up/down-regulated DEGs in 3 cell lines

	**Ranking/cell line**	**JEG-3**	**U-251 MG**	**HK-2**
**Up-regulated genes**	1	IFNL3	CXCL10	DHRS2
	2	CCL5	RSAD2	IFI27
	3	IFNL1	XAF1	LOC100133669
	4	IFIT2	IFNL1	ZBP1
	5	RTL9	TNFSF14	OAS2
	6	TNF	CXCL11	FBXO39
	7	DHX58	CHRNA1	KLHDC7B
	8	IFIT3	OASL	VGF
	9	IL1A	MX2	MX2
	10	EGR1	CH25H	TAC1
**Down-regulated genes**	1	POTEG	CGB5	IL33
	2	HPN	KRT19	SPSB4
	3	CATSPERE	NTF4	LMOD1
	4	CD22	CGB2	REN
	5	INPP5J	KRT75	KIAA1210
	6	CTAGE8	RGS8	ENPP3
	7	ZNF221	CNGA3	LOC100129518
	8	CYP1A1	ALPP	KCNK2
	9	AC136616.1	KRT13	ZCCHC5
	10	ZNF846	ACKR3	GMNC

Specifically, interferon lambda (IFNL) relative genes such as IFNL1 and IFNL3, were up-regulated in both the JEG-3 cell line and U-251 MG cell line. IFNL has been demonstrated to confer protection against HSV-1 and ZIKV replication [34, 35]. Differently, ZIKV infection tended to up-regulate IFNL3 with FC value of 351-fold in the JEG-3 cell line (Fig. 1b) but up-regulated IFNL1 with FC value of 102.6-fold in U-251 MG cells (Fig. 1c), indicating IFNL3 in the placenta may be more sensitive for antiviral effects. For the HK-2 cell line, the IFNL related genes were not up-regulated after ZIKV infection (Fig. 1d), but the innate immune response was significantly induced, including ZBP1, which plays a role in the innate immune response by binding to foreign DNA and inducing type-I interferon production [36], as well as OAS2, an essential protein involved in the innate immune response to viral infection [37]. Together, these results suggested that host immune response to ZIKV infection showed tissue specificity.

### ZIKV infection induced profound IFNs production

**Fig. 2:**
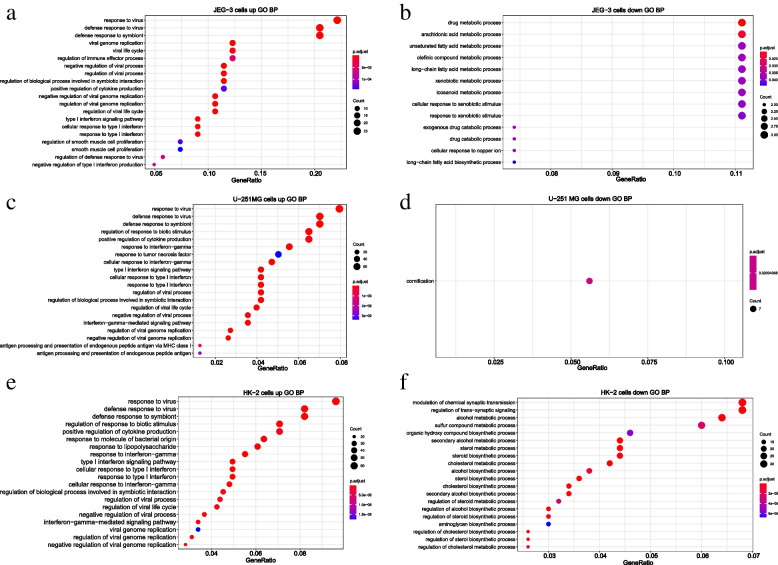
The enriched GO BP of up-regulated and down-regulated genes in JEG-3, U-251 MG, and HK-2 cells. **a-b** The enriched GO BP of up-regulated and down-regulated genes in JEG-3 cells. **c-d** The enriched GO BP of up-regulated and down-regulated genes in U-251 MG cells. **e–f** The enriched GO BP of up-regulated and down-regulated genes in HK-2 cells. Enrichment analyses were performed by *clusterProfiler* package of R software. Hypergeometric tests were used to screen enriched GO BPs, and P-values were further adjusted by the default “Holm” method. Those GO BPs with an adjusted *P*-value < 0.05 were considered significant. Here, the top 20 significant GO BPs ranked by adjusted p-value were displayed

**Fig. 3:**
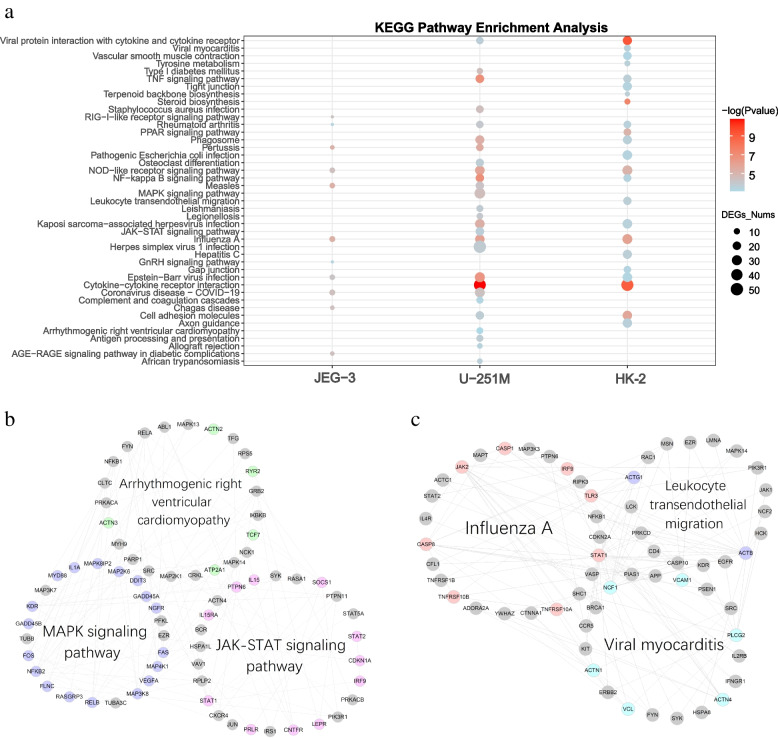
The enriched KEGG pathway of DEGs and PPI network analysis in JEG-3, U-251 MG, HK-2 cell lines. **a** The enriched KEGG pathway of DEGs in JEG-3, U-251 MG, and HK-2 cells. Here, the total top 30 significant pathways ranked by adjusted p-value in 3 cell lines were displayed. **b** The example enriched KEGG pathway of DEGs in U-251 MG cells. **c** The example enriched KEGG pathway of DEGs in HK-2 cells. Here, the lines represent the interactions in the PPI network. Node colors represent DEGs annotated on different pathways. DEGs on JEG-3 cells sporadically map to the PPI network, so their PPI network is not shown. Enrichment analyses were performed by *clusterProfiler* package of R software. Hypergeometric tests were used to screen enriched KEGG pathways, and P-values were further adjusted by the default “Holm” method. Those KEGG pathways with an adjusted *P*-value < 0.05 were considered significant

To analyze the molecular mechanism underlying ZIKV infection in cells from different tissues, corresponding DEGs were enriched through Gene Ontology Biological Process (GO BP) (Fig. 2, Supplementary Fig. 3, Supplementary Tables 5, 6, 7, 8, 9, 10, 11, 12 and 13) and pathways from KEGG (Kyoto Encyclopedia of Genes and Genomes) pathway database [38–40] (Fig. 3, Supplementary Tables 14, 15 and 16). The enrichment results on GO BP showed that the up-regulated genes in these three cell lines were preferentially enriched in defense response to virus, regulation of viral genome replication, type I IFN signaling pathway, etc. (Fig. 2a,c,e). These data indicated that ZIKV infection was likely to induce massive viral defense processes, in particular, the type I IFN signaling pathway (Supplementary Table 6, 9, 12), which might lead to the downstream antiviral immune response.

Furthermore, ZIKV down-regulated multiple metabolic-related BPs such as the arachidonic acid metabolic process in the JEG-3 cells (Fig. 2b, Supplementary Table 7), amine metabolic process in the U-251 MG cells (Supplementary Table 10), and cholesterol metabolic process in HK-2 cells (Fig. 2f, Supplementary Table 13), respectively. These results indicated that ZIKV infection could also disturb the host metabolic system, which is consistent with previous studies that ZIKV infection could trigger the host metabolic reprogramming [41, 42]. Besides, ZIKV infection also inhibited the process of cornification in U-251 MG (Fig. 2d, Supplementary Table 10). While in HK-2 cell line, ZIKV peculiarly restrained sterol biosynthetic process, secondary alcohol biosynthetic process, etc. (Fig. 2f, Supplementary Table 13).

The KEGG pathway enrichment further supports the above results and revealed that ZIKV infection is apt to regulate the IFN-related biological pathways (Fig. 3, Supplementary Tables 14, 15 and 16). For example, ZIKV infection induced the RIG-I-like receptor (retinoic acid-inducible gene I-like receptors, RLR) signaling pathway in the JEG-3 cell line (Fig. 3a, Supplementary Table 14), where RIG-I could detect viral RNAs and activate the type I IFN-mediated antiviral immune response during infection [43]. Similarly, ZIKV-infected U-251 MG could specifically affect the JAK-STAT signaling pathway (Fig. 3a, Supplementary Table 15), including IFN-stimulating gene 15 (ISG15), which was reported to restrict viral replication and spread [44]. The IFN-related antiviral effects are owing to the contest between ZIKV virus infection and host antiviral immune response.

It is further observed that ZIKV infection also induced a strong inflammatory response, immune regulatory and virus-related pathways. Typically, DEGs in JEG-3 cells were significantly enriched in other RNA virus-related pathways, such as Influenza A viruses, Coronaviruses, Epstein-Barr viruses infection, indicating a series of immune effects were evoked by ZIKV infection (Fig. 3a, Supplementary Table 14). In addition to the IFN-related pathways, ZIKV infection in U-251 MG also specifically regulates the Arrhythmogenic Right Ventricular Cardiomyopathy (Fig. 3b) and Phenylalanine metabolism pathway, where the latter was reported to be associated with vector control of Blood-feeding arthropods upon ZIKV infection [45] (Supplementary Table 15). The pathway analysis of HK-2 cell line indicated different regulation patterns in kidney by initiating pathways, such as Influenza A, Viral myocarditis, and Leukocyte transendothelial migration (Fig. 3c), the latter was reported to enhance monocyte adhesion and transmigration favoring viral dissemination to neural cells during ZIKV infection [46]. Also, other signaling pathways, such as MAPK signaling pathway and PPAR signaling pathway (Supplementary Table 16), were stimulated to combat ZIKV infection. Just as previous studies indicated, PPAR signaling pathway was dysregulated in ZIKV infected neural progenitor cells [47], and its down-regulation might dysregulate sertoli energy supplies and adversely affect spermatogenesis [48]. Together, ZIKV infection usually triggered IFN responses to combat virus replication or invasion. In addition to common mechanisms, each infected organ also simulated tissue-specific immune responses or other BPs.

### ZIKV infection induced delayed IFN induction in placenta-derived cells

**Fig. 4:**
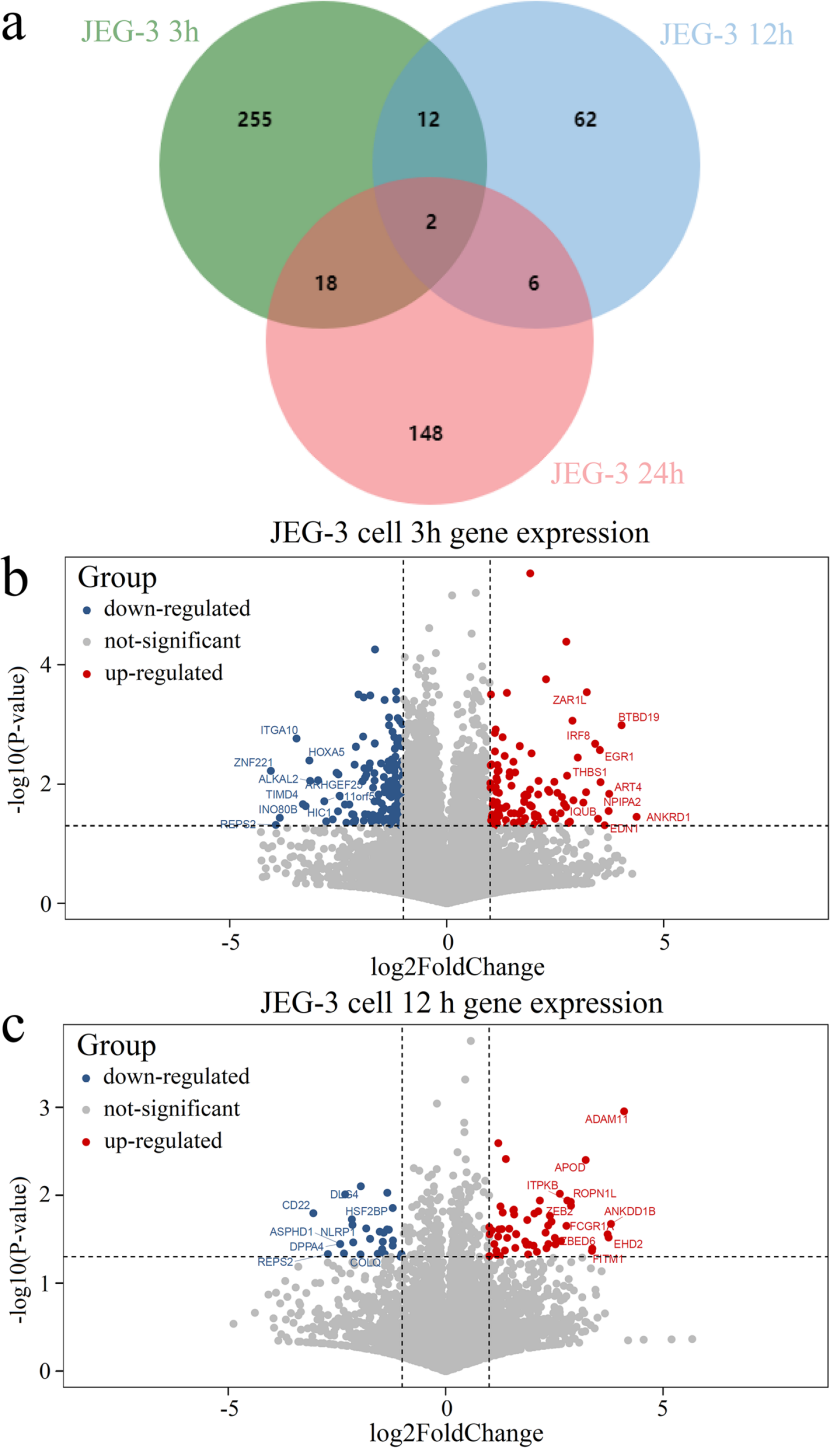
DEGs induced by ZIKV infection in JEG-3 cells at different time points. **a** DEGs in JEG-3 cells at 3 h, 12 h, and 24 h post ZIKV infection. **b** DEGs in JEG-3 cells induced at 3 h.p.i.. **c** DEGs in JEG-3 cells at 12 h.p.i.. The up-regulated genes were labeled as red points, and down-regulated genes were labeled as blue points. The top 10 up-regulated and down-regulated genes were detected by FC value and were labeled on the figures. Several labeled genes are not shown in the figures due to their overlap with other genes. JEG-3 cells were infected with ZIKV African strain MR766 (MOI = 1) at 3 time points (3 h, 12 h, 24 h). The control samples were the corresponding cells without ZIKV infections. The two-tailed student’s t-test was used in the detection of the DEGs between corresponding infected cells and uninfected cells. Each experiment was repeated for 3 times

Further, to explore the anti-ZIKV response in placentae, infected JEG-3 cells were harvested for determining host RNA levels by RNA-seq analysis at different post-infection times (3 h, 12 h, and 24 h). By comparing with un-infected control, DEGs for three time points were detected (Fig. 4a). It can be found that, 287 DEGs were detected at 3 h post-ZIKV infection, which includes 108 up-regulated and 179 down-regulated genes (Supplementary Table 17). After 12 h.p.i., the whole number of DEGs decreased to 82, of which 55 were up-regulated and 27 were down-regulated (Supplementary Table 18). Later, the DEGs increased to 174 at 24 h.p.i., which contains 142 up-regulated and 32 down-regulated ones (Supplementary Table 1).

Intriguingly, results in Fig. 4a showed that there are only 2 overlapped DEGs among three time points post-infection on the JEG-3 cell line, indicating placenta cells were at different antiviral states overtime after ZIKV infection. Notably, there were fewer genes differentially expressed at 12 h.p.i. than those at 3 or 24 h.p.i., both up-regulated (red dots) and down-regulated genes (blue dots). It might be caused by compensation reactions after 12 h of ZIKV infection. The top 10 up- and down-regulated genes at 6 h.p.i. and 12 h.p.i. are listed in Table 2, while DEGs at 24 h.p.i. were already shown in Table 1.

**Table 2:** The top 10 up/down-regulated DEGs in different time points of JEG-3 cells

	**Ranking/cell line**	**3 h**	**12 h**
**Up-regulated genes**	1	ANKRD1	ADAM11
	2	BTBD19	ANKDD1B
	3	ART4	EHD2
	4	NPIPA2	FCGR1A
	5	EDN1	ZBED6
	6	THBS1	FITM1
	7	EGR1	APOD
	8	IQUB	ZEB2
	9	IRF8	ROPN1L
	10	ZAR1L	ITPKB
**Down-regulated genes**	1	ZNF221	CD22
	2	REPS2	REPS2
	3	INO80B	ASPHD1
	4	ITGA10	DPPA4
	5	TIMD4	COLQ
	6	HIC1	DLG4
	7	HOXA5	HSF2BP
	8	ALKAL2	NLRP1
	9	ARHGEF25	CATSPERE
	10	C11orf52	CLEC18A

Specifically, only a few top up-regulated genes at 3 h.p.i. and 12 h.p.i. are associated with anti-viral infection, such as IRF8 (3 h.p.i.) which could regulate the expression of IFN-stimulating genes (ISGs), and FCGR1A (12 h.p.i.) that encodes Fc-gamma receptors, which is essential for immune response (Table 2). On the other hand, most of the top up-regulated genes at 24 h.p.i. were closely related to anti-viral responses. Of the top 3 up-regulated genes, IFNL1 and IFNL3 are related to innate immune response, while CCL5 is associated with T-cell-mediated cell immunity (Table 1). Moreover, IFIT2 and IFIT3 are IFN-stimulating genes targeted directly by IRF3, which mediated antiviral response [49]. DHX58 (LGP2) is also the up-regulated antiviral signature involved in cytoplasmic recognition of RNA viruses. Besides, as one of the cytosolic viral RNA sensors, LGP2 belongs to the RLR family and mediated the production of type I IFNs, antiviral effector genes, and pro-inflammatory cytokines [50]. Also, IL1A has been reported to involve in various immune responses [51].

Upon ZIKV infection, the expressions of IFN-related genes (such as IFNL1 and IFNL3) and ISG-related genes (such as ISG15 and ISG20) were only marginally elevated until 24 h.p.i. (Fig. 1b, Supplementary Table 1). These observations showed that ZIKV infection may stimulate substantial but delayed IFN production, suggesting that this flavivirus attenuated host antiviral response.

**Fig. 5:**
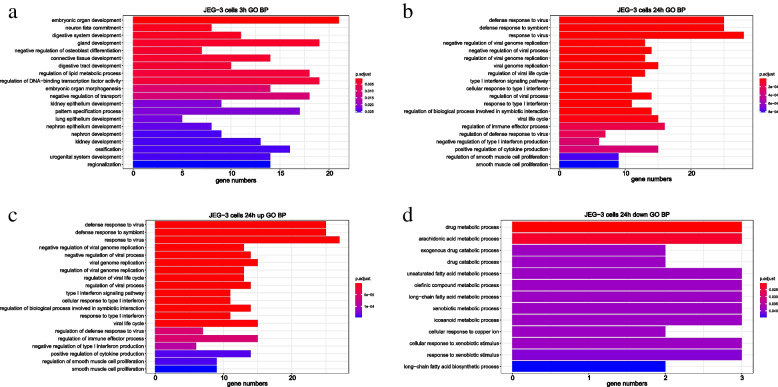
The enriched GO BP of DEGs in placental cells at different time points. **a** The enriched GO BP in JEG-3 cells at 3 h post-ZIKV infection. **b** The enriched GO BP in JEG-3 cells at 24 h post-ZIKV infection. **c-d** The enriched GO BP of up-regulated and down-regulated DEGs at 24 h.p.i.. The DEGs at 12 h.p.i. failed to enrich at any GO BP, thus not shown. The enriched GO-BP of up-regulated DEGs at 3 h post-ZIKV infection was shown in Supplementary Table 20, while down-regulated DEGs at 3 h.p.i. failed to enrich at any GO BP, thus not shown. Enrichment analyses were performed by *clusterProfiler* package of R software. Hypergeometric tests were used to screen enriched GO BPs, and *P*-values were further adjusted by the default “Holm” method. Those GO BPs with an adjusted *P*-value < 0.05 were considered significant. Here, the top 20 significant GO BPs ranked by adjusted p-value were displayed

**Fig. 6:**
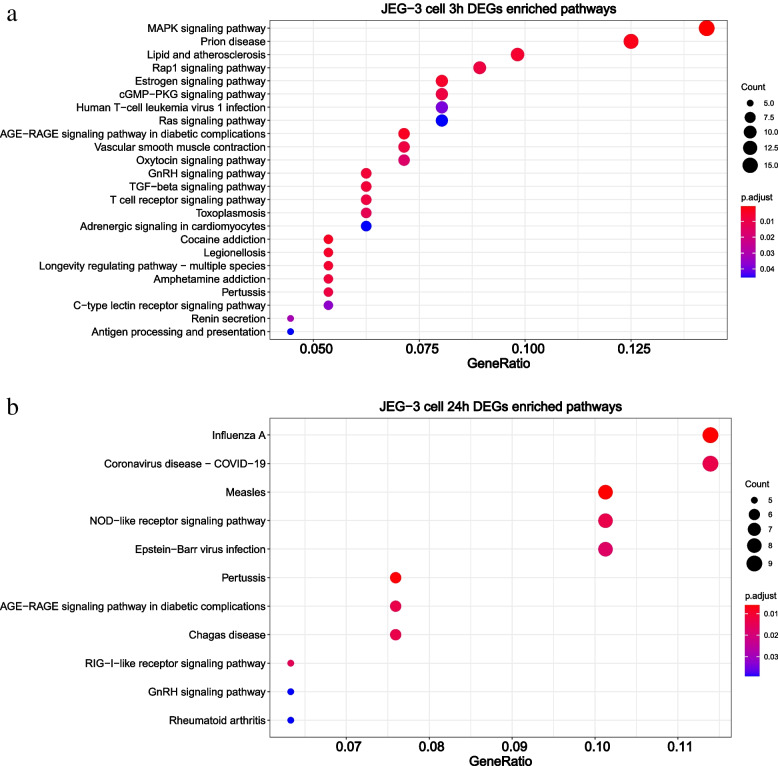
The enriched KEGG pathway of DEGs in placental cells at different time points. **a** The enriched KEGG pathway of DEGs in JEG-3 cells at 3 h post ZIKV infection. **b** The enriched KEGG pathway of DEGs in JEG-3 cells at 24 h post ZIKV infection. The DEGs at 12 h.p.i. failed to enrich at any KEGG pathway, thus not shown. Enrichment analyses were performed by *clusterProfiler* package of R software. Hypergeometric tests were used to screen enriched KEGG pathways, and P-values were further adjusted by the default “Holm” method. Those KEGG pathways with an adjusted *P*-value < 0.05 were considered significant. Here, the top 30 significant pathways ranked by adjusted *p*-value were displayed

Furthermore, DEGs at the above three time points were annotated to GO BPs (Fig. 5, Supplementary Tables 5, 6 and 7, 19, 20) and pathways (Fig. 6, Supplementary 14 and 21). As shown in Fig. 5, DEGs at 3 h and 24 h post infection are enriched in completely different top 20 BPs (Fig. 5a-b), while DEGs of 12 h after ZIKV infection failed to enrich at any BPs and pathways. This may be because the rapid response to placental infection has faded before an advanced response would be triggered.

As shown in Fig. 5a, ZIKV infection mainly regulated the process of embryonic organ development, neuron fate commitment, and digestive system development at 3 h.p.i. (Supplementary Table 19). Whereas changes at 24 h.p.i. were the regulation of viral life cycle, viral process, type I IFN, etc. (Fig. 5b, Supplementary Table 5). In the enriched GO BP of up-regulated genes, only the process of positive regulation of smooth muscle cell proliferation was common at 3 h.p.i. and 24 h.p.i. (Supplementary Table 6 and 20). BPs such as regulation of lipid metabolic process, negative regulation of osteoblast differentiation, and circadian regulation of gene expression were induced by ZIKV infection at 3 h.p.i. (Supplementary Table 20). While at 24 h.p.i., processes such as regulation of viral genome replication, regulation of viral life cycle, type I IFN signaling pathway, etc. were particularly up-regulated (Fig. 5c, Supplementary Table 6). It’s indicated that significant activation of the type I interferon pathway occurs until the late stage of ZIKV infection, mainly because virus hijacked the host protein such as AXL to interfere in the activation of IFN signaling pathway [13]. In addition, down-regulated genes at 24 h.p.i. were primarily enriched in some metabolic-related processes, such as drug metabolic process, arachidonic acid metabolic process, exogenous drug catabolic process (Fig. 5d, Supplementary Table 7).

It can be observed from KEGG pathway analysis that ZIKV-infected JEG-3 cells at 3 h.p.i. and 24 h.p.i. up-regulated these three common pathways, namely AGE-RAGE signaling pathway in diabetic complications, GnRH signaling pathway, and Pertussis (Fig. 6a,b, Supplementary Tables 14 and  2

1). Typically, DEGs at 3 h.p.i. were enriched in some canonical signaling pathways such as Estrogen signaling pathway, MAPK signaling pathway, etc. (Fig. 6a, Supplementary Table 21). As reported previously, the estrogen receptor modulators quinestrol and raloxifene effectively inhibited ZIKV, DENV, and WNV infection at low micromolar concentrations [52]. Moreover, ZIKV was reported to damage many typical astrocyte signaling pathways, including axon guidance signal, FGF signal, STAT3 signal, AMPK, and ERK/MAPK signal, etc. [53]. Besides, some immune-related processes are also specifically initiated after 3 h of ZIKV infection, such as antigen processing and presentation pathways.

After 24 h of ZIKV infection, more IFN-related biological pathways were stimulated, such as RIG-I-like receptor signaling pathway (Fig. 6b, Supplementary Table 14). Besides, other viral infection-related pathways are also stimulated, such as Coronavirus disease-COVID-19 and Epstein- Barr virus infection, indicating induced sufficient immune response at 24 h post-ZIKV infection (Fig. 6b, Supplementary Table 14). These observations show that ZIKV induced overt but delayed type I IFN responses when infecting placenta, which was similar to SAR-COV-2 in the airway epithelial cell line [54–56], suggesting that ZIKV infection perturbed host immune responses and provided mechanistic insights into the immune evasion.

## Discussion

In this study, the DEGs between control and ZIKV infection were detected in JEG-3, U-251 MG, and HK-2 cell lines via RNA-seq technology. Particularly, some DEGs in invading tissues were reported to play antiviral effects not only in combating ZIKV infection, but also in SARS-CoV-2 infection. For example, except for suppressing ZIKV infection, CH25H as one of the ISGs, was reported to be induced by SARS-CoV-2 infection in vitro and COVID-19-infected patients. Also, its product 25HC showed broad anti-coronavirus activity by blocking membrane fusion, which also inhibited SARS-CoV-2 infection in lung epithelial cells and viral entry in human lung organoids [57, 58].

The above analysis detected 33 common DEGs among three cell lines in 24 h.p.i. (Fig. 1a), which were involved in multiple antiviral functions (See *Results* part). Moreover, we detected tissue-specific DEGs for JEG-3 cells (Supplementary Table 22), U-251 MG cells (Supplementary Table 23), and HK-2 cells (Supplementary Table 24), respectively. Among 98 specific DEGs in JEG-3 cells, IFNL3 encodes a cytokine distantly related to type I interferons, and can be induced by viral infection [59]. Also, TNF has been reported for its involvement in coronavirus biology, and is involved in cytokine storm inflammatory response [60]. Moreover, targeting TNF was proved to alleviate Zika virus complications in mouse models [61]. Besides, among 1,057 specific DEGs in U-251 MG cells, IL15 regulates T and natural killer cell activation and proliferation [62]. MYD88 encodes a cytosolic adapter protein that plays a central role in the innate and adaptive immune response, which functions as an essential signal transducer in the interleukin-1 and Toll-like receptor signaling pathways [63]. Also, among 1,166 DEGs in HK-2 cells, IL33 is involved in the maturation of Th2 cells and the activation of mast cells, basophils, eosinophils and natural killer cells [64]. Also, IFNB1 encodes a cytokine that belongs to the interferon family of signaling proteins, which is released as part of the innate immune response to pathogens. The protein encoded by this gene belongs to the type I class of interferons, which are important for defense against viral infections [65]. These results indicated that when zika virus infects different cells or tissues, it not only stimulates the differential expression of the same genes, but also exhibits tissue specificity.

For time series analysis, the DEGs of JEG-3 cells in placenta tissue after infection for 3 h, 12 h and 24 h were evaluated, which involved only 2 overlapped DEGs including DLG4 and CACNA1S (Fig. 4a). Among them, DLG4 encodes a member of the membrane-associated guanylate kinase (MAGUK) family, and is involved in receptor tyrosine kinase signaling [66]. CACNA1S is previously reported to be associated with hypokalemic periodic paralysis, thyrotoxic periodic paralysis and malignant hyperthermia susceptibility [67]. In addition, there are 255 specific DEGs after 3 h of infected placenta cells (Supplementary Table 25). Among them, IRF8 controls the expression of IFNα- and IFNβ-regulated genes that are induced by viral infection [68]. Also, 62 genes were specifically differentially expressed after 12 h of treatment in JEG-3 cells (Supplementary Table 26). For example, IFITM1 is a member of interferon family that induced antiviral proteins, which restricts cellular entry by diverse viral pathogens, such as influenza A virus, Ebola virus and SARS-CoV-2 [69]. Besides, 148 genes were specifically differentially expressed in placenta cells after 24 h of infections (Supplementary Table 27). For example, IFIT3 is involved in defense response to virus, negative regulation of viral genome replication, and positive regulation of IκB/NFκB signaling [70]. Also, IL6 encodes a cytokine that functions in inflammation and the maturation of B cells, contributing to host defense during infection and tissue injury [71].

The above result illustrated that the anti-infection mechanisms triggered by the placenta tissues varied considerably during the different periods of ZIKV infection. In particular, during the early stage of placental infection (3 h), the highest number of DEGs among all three time points are detected, which were stimulated to resist ZIKV invasion and replication. During the middle stage of infection (12 h), the anti-viral infection was decreased. In contrast, during the late stage of infection (24 h), the antiviral intensity regained, particularly in triggering the response of interferon-related pathways, which meant a delayed action of interferon in the placental tissue.

Furthermore, GO BP enrichment and KEGG pathway analysis of three cell lines facilitated the discovery of highly significant genes and pathways during ZIKV infection. Collectively, the up-regulated DEGs of all three cells were enriched into multiple IFN-related signaling pathways, from the RLR pathway and NF-κB pathway related to the production of type I interferon, to the JAK-STAT pathway and ISGs production which were activated after the recognition of type I interferon. Meanwhile, the NOD-like receptor (NLR) signaling pathway, which was crucial in innate immune response, was observed to be enriched in all three cell lines (Fig. 3a, Supplementary Table 14, 15 and 16). This result suggests that ZIKV infection in these tissues can activate the up-stream pattern recognition molecules NLRs in innate immunity. While some NLRs recruit and activate inflammatory caspases into inflammasomes, others trigger inflammation via alternative routes including the NF-κB, MAPK, and regulatory factor pathways [72–74]. The critical role of NLRs and their downstream signaling components in development and physiology is demonstrated by their clear implications in human diseases. Recently, Ting et al. has found acute kidney injury induced in ZIKV infection was caused by activation of NLRP3 inflammasome and thereby suppression of BCL2 [75], which was consistent with our analysis above. Interestingly, the results of KEGG pathway analysis showed that NLR signaling pathway was enriched at 24 h.p.i. but not at 3 h.p.i. (Fig. 6, Supplementary Tables 14 and 21), suggesting that endogenous damage-related molecular pattern (DAMP) generated during the replication process of ZIKV virus in placental cells may be recognized by NLR to activate NLR signaling pathways [76]. Furthermore, in placental cells, we found that IFN induction and ISG response were significantly delayed until 24 h after virus infection. It is well known that IFN signaling is the first line for anti-viral defense, thus making ZIKV develops strategies to counteract the IFN signaling [77–79]. However, the fast induction of type I IFN production by astrocytes plays an important role in self-protection of astrocytes and suppression of ZIKV replication in the Central Nervous System (CNS). Moreover, primary human trophoblasts (PHTs) constitutively release the type III interferon-IFNL1, which may protect trophoblast and non-trophoblast cells from ZIKV infection [34]. These results further confirm that delayed IFN induction is likely to respond for rapid ZIKV replication in human placentae.

Moreover, the ZIKV infection could also provoke some extent host adaptive immune response based on GO BP enrichment analysis. The enriched GO BPs in the JEG-3 cells mainly included activated T cell proliferation and T-helper 2 cell cytokine production (Supplementary Table 6), whereas in U-251MG and HK-2 cell lines, the enriched GO BPs are mainly involved in interferon-gamma response, regulation of T cell proliferation, positive regulation of T cell-mediated cytotoxicity, and antigen processing and presentation of peptide antigen via MHC class I (Fig. 2c,e, Supplementary Tables 9 and 12). Notably, the placenta has been reported as a major tissue for ZIKV replication, and its infection in pregnant women could cause intrauterine growth restriction, spontaneous abortion, and microcephaly [80]. The GO-BP enrichment verified that the placenta mostly up-regulated the process of T cell activation and proliferation, rather than stimulating down-stream immunity response, such as positive regulation of T cell-mediated cytotoxicity, antigen processing, and presentation of endogenous peptide antigen in astrocytes, which may contribute to the serious harm of nervous infection and vibrant viral replication in human placenta.

We further uncovered that the DEGs induced in U-251 MG cells were also enriched in Arrhythmogenic Right Ventricular Cardiomyopathy (Fig. 3a-b, Supplementary Table 15), which was consistent with prior data present in ZIKV infected neonatal non-human primate pregnancy model, accompanied by microencephaly, seizures, and cardiomyopathy [81]. For other stimulated pathways**,** our KEGG enrichment analysis was also consistent with previous studies. For example, the enriched Leukocyte transendothelial migration pathway in HK-2 cells can enhance monocyte adhesion and transmigration favoring viral dissemination to neural cells (Fig. 3c) [46]. Besides, there are also reports that interpreted ZIKV infection interferes with or changes the astrocyte proteins involved in synaptic control and axon guidance [53, 82], verifying the regulatory effect of ZIKV infection on the Axon guidance pathway (Fig. 3a, Supplementary Table 16).

In addition, it’s shown that ZIKV infection in each cell line also specifically down-regulated certain BPs via enrichment analysis. For example, JEG-3 cell line specifically down-regulated the process of unsaturated fatty acid metabolic process, long-chain fatty acid biosynthetic process, long-chain fatty acid metabolic process, etc. (Fig. 2b, Supplementary Table 7). It is consistent with previous studies that ZIKV infection reprogrammed placental lipidome by impairing the lipogenesis pathways [42]. The metabolic alterations induced by ZIKV provided the basis for lipid droplet biogenesis and intracellular membrane rearrangements to support viral replication. Furthermore, lipidome reprogramming caused by ZIKV is accompanied by mitochondrial dysfunction and inflammatory immune imbalance, which contribute to placental damage [42].

Note that, different ZIKV strains might illustrate various effects on infected cells or tissues. For example, animal model experiments showed that African ZIKV strains could induce short but severe neurological symptoms followed by lethality in mice, and the Asian strain manifested prolonged signs of neuronal dysfunction and occasionally caused the death of mice. Moreover, viral RNA levels in different organs seem not associated with the pathogenicity of the different strains [83]. Another study showed dramatic differences in the inflammatory response elicited by the American ZIKV strain from Brazil and its Asian ancestral strain isolated from Cambodia. Compared with Asian strain, the experimental infection of human-induced neuroprogenitor stem cells (hiNPCs) with American ZIKV resulted in a diminished induction of IFNs stimulated genes (ISGs) and lower induction of several cytokines including IFN-α, IL-1α/β, IL-6, IL-8, and IL-15, which consequently favoring virus replication [84]. Based on the above observations, we speculated that the African ZIKV strain used in our study may induce higher levels of inflammatory cytokines and markers associated with cellular infiltration in DEGs than Asian or American strains, which may explain exacerbated pathogenesis compared to those of the Asian or American lineage.

## Conclusions

This study reveals the biological response and pathways against ZIKV infection in different human organs or tissues, such as GnRH signaling pathway in placental choriocarcinoma JEG-3 cells, MAPK signaling pathway in astrocytes U 251-MG cells, PPAR signaling pathway in renal-derived HK-2 cell lines and IFN-related pathway activation in all three cell lines. Notably, a delayed interferon response in ZIKV-infected placenta-derived cells explains the molecular mechanism why ZIKV replicates rapidly in the placenta and prompts a possibility of ZIKV vertical transmission. Besides, the above tissue-specific immune responses or BPs stimulated by ZIKV infection in this transcriptome analysis can guide the investigation about the pathogenesis of ZIKV infection in other ZIKV-tropic tissues.

## Methods

### Cell lines and viruses

*Aedes albopictus* C6/36 cells were grown in 30% RPMI-1640 (Gibco) and 60% Dulbecco’s modified Eagle’s medium (Gibco) supplemented with 10% fetal bovine serum (FBS, Gibco). U-251 MG cell line was purchased from BeNa Culture Collection (BNCC) and authenticated by short tandem repeat (STR) as described in the previous study [13]. HK-2 and JEG-3 cell lines were purchased from the American Type Culture Collection (ATCC) and were both authenticated by STR authentication. U-251 MG cells were cultured in DMEM (Gibco) supplemented with 10% FBS, 100 IU/mL of penicillin, and 100 µg/mL of streptomycin. JEG-3 cells were cultured in MEM (Gibco) supplemented with 10% FBS, 100 IU/mL of penicillin, and 100 µg/mL of streptomycin. HK-2 cells were cultured in DMEM/F12 (1:1, Gibco) supplemented with 10% FBS, 100 IU/mL of penicillin, and 100 µg/mL of streptomycin. U-251 MG, JEG-3, and HK-2 cells were maintained at 37 °C and C6/36 cells at 28 °C in a fully humidified atmosphere containing 5% CO_2_. All cell lines were tested by Saily Bio (Shanghai, China) and are free of mycoplasma contamination. The ZIKV MR766 stock was purchased from ATCC (ATCC® VR-1838™).

### ZIKV infection and samples preparation for RNA-seq analysis

Before ZIKV infection, JEG-3, U-251 MG, and HK-2 cells were seeded in 10 cm dishes (2 × 10^6^ cells per dish). At 24 h (h) after seeding, the cells were rinsed once with phosphate-buffered saline (PBS) and were then incubated with ZIKV African strain MR766 at an MOI of 1 in serum-free medium for 1 h at 37 °C unless otherwise noted. The ZIKV-containing medium was then replaced with fresh DMEM or MEM supplemented with 2% FBS and incubation for the indicated time. Cells were rinsed twice with cold PBS and were then collected into clean tubes and lysed in RNAzol® RT RNA Isolation Reagent for RNA isolation. The experiments of controls or ZIKV infected groups were conducted in three replicates for both U-251 MG and HK-2 cells. Likewise, three replicates were analyzed in JEG-3 cells at each time point.

### RNA extraction and sequencing assays

RNA extraction and RNA-seq were performed in Biowavelet Co., LTD. RNA was extracted using an RNEasy RNA isolation kit (Qiagen) according to the manufacturer’s instructions. RNASeq short reads were aligned to the human genome (GRCh38) using GSNAP with a maximum of two mismatches. Gene expression was determined as the number of short reads that fully/partially aligned to the annotated gene model using HTseq. Expressed genes were defined as those genes having more than 10 total mapped reads in all samples with at least two of three replicates having more than two reads.

### The detection of DEGs in ZIKV infected cells

The RNA-seq was performed in infected cell lines under mock and ZIKV-infected conditions, including placental cell line (JEG-3), nerve cell line (U-251 MG), and kidney cell line (HK-2). The placental cell line was measured at 3 h,12 h, and 24 h post-ZIKV infection, while the other two cell lines were uniformly measured at 24 h post infection (h.p.i.) merely. To examine inter- and intra- differences of ZIKV infection among various tissues, DEGs were firstly detected by two-tailed Student’s t-test in turn by comparing with corresponding mock infection. In each group, those genes with P values less than 0.05 and FC larger than 2 or less than 0.5 were detected as DEGs. Then DEGs in each group were depicted in volcano plot by *EnhancedVolcano* package of R software. The top 10 up-regulated and down-regulated genes were detected by FC value and were labeled on the corresponding figures.

### The functional annotation of DEGs in infected cells

To further explore the internal mechanism of ZIKV infection on different tissues, DEGs in each tissue at different infected time points were executed for functional annotations. In detail, GO BP functional annotation analysis and KEGG pathway enrichment analysis were performed for the screened DEGs, which were performed by *clusterProfiler* package of R software. Those GO-BP and KEGG pathways with an adjusted *P*-value < 0.05 were considered significant.

Moreover, to analyze the gene and function interaction of DEGs, DEGs under different scenarios were annotated into the background PPI network using Cytoscape software version 3.4.0 [85], with different colors to distinguish different functional pathways. Here, the background PPI network used in this project contained 10,462 nodes and 55,317 interactions were constructed mainly based on three databases, including HPRD version 9 [86], Mint version 2012 [87], and IntAct version 4.2.12 [88]. Biological pathways for enrichment and analysis were integrated from KEGG version 87.0 [89] and GeneCards version 4.12 [90].

## Supplementary Information

**Additional file 1.** 

**Additional file 2:** **Supplementary Fig. 1. **The classification plot of samples from three cell lines based on all the DEGs. Cells were treated with (3, 12, 24 h.p.i.) or without (normal group) ZIKV infection; **Supplementary Fig. 2. **The expression heatmap of 33 common DEGs among JEG-3, U-251 MG, and HK-2 cells; **Supplementary Fig 3. **The enriched GO BPs of DEGs from 3 cell lines, including (a) JEG-3, (b) U-251 MG, (c) HK-2 cells. **Supplementary Table 1. ** DEGs in JEG-3 cells of 24h; **Supplementary Table 2. ** DEGs in U-251 MG cells of 24h; **Supplementary Table 3. **DEGs in HK-2 cells of 24h; **Supplementary Table 4. **33 common DEGs and functions in 3 cell lines; **Supplementary Table 5. **The GO-BP enrichment of DEGs in JEG-3 cells of 24h; **Supplementary Table 6. **The GO-BP enrichment of up-regulated DEGs in JEG-3 cells of 24h; **Supplementary Table 7. **The GO-BP enrichment of down-regulated DEGs in JEG-3 cells of 24h; **Supplementary Table 8. **The GO-BP enrichment of DEGs in U-251 MG cells of 24h; **Supplementary Table 9. **The GO-BP enrichment of up-regulated DEGs in U-251 MG cells of 24h; **Supplementary Table 10. **The GO-BP enrichment of down-regulated DEGs in U-251 MG cells of 24h; **Supplementary Table 11. ** The GO-BP enrichment of DEGs in HK-2 cells of 24h; **Supplementary Table 12. **The GO-BP enrichment of up-regulated DEGs in HK-2 cells of 24h; **Supplementary Table 13. **The GO-BP enrichment of down-regulated DEGs in HK-2 cells of 24h; **Supplementary Table 14.** The pathway enrichment of DEGs in JEG-3 cells of 24h; **Supplementary Table 15. **The pathway enrichment of DEGs in U-251 MG cells of 24h; **Supplementary Table 16. **The pathway enrichment of DEGs in HK-2 cells of 24h; **Supplementary Table 17. **DEGs in JEG-3 cells of 3h; **Supplementary Table 18. **DEGs in JEG-3 cells of 12h; **Supplementary Table 19. **The GO-BP enrichment of DEGs in JEG-3 cells of 3h; **Supplementary Table 20. **The GO-BP enrichmentin of up-regulated DEGs in JEG-3 cells of 3h; **Supplementary Table 21. **The pathway enrichment of DEGs in JEG-3 cells of 3h.

## Data Availability

All data relevant to the study are included in the article and in additional files. The reagents used in this publication are available from the corresponding author on reasonable request. The Next-generation sequencing (NGS) gene analysis data were deposited in the Extended dataset.

## Abbreviations

ZIKV: Zika virus
PPI: Protein–protein interaction
BPs: Biological processes
DENV: Dengue virus
YFV: Yellow fever virus
WNV: West Nile virus
JEV: Japanese encephalitis virus
TBEV: Tick-borne encephalitis viruses
RNA-seq: RNA-sequencing
PBS: Phosphate-buffered saline
h.p.i.: Hours post infection
TLR3: Toll-like receptor 3
RIG-I: Retinoic acid-inducible gene I
MDA5: Melanoma differentiation-associated gene 5
hNPCs: Human neural progenitor cells
HCs: Hofbauer cells; IFN: interferon
RLR: RIG-I-like receptor
BNCC: BeNa Culture Collection
ATCC: American Type Culture Collection
FC: Fold change
HSV-1: Herpes simplex virus 1
HVEM: Herpesvirus entry mediator
IFNL: Interferon lambda
ISG15: IFN-stimulating gene 15
NLR: NOD-like receptor
DAMP: Damage-related molecular pattern
PHTs: Primary human trophoblasts
DEG: Differentially Expressed Genes
CNS: Central Nervous System
KEGG: Kyoto Encyclopedia of Genes and Genomes
