# Supplementary material for: Revealing the characteristics of ZIKV infection through tissue-specific transcriptome sequencing analysis
Source: BMC Genomics. 2022 Oct 8;23:697. doi: 10.1186/s12864-022-08919-5 (PMC9546753; doi:10.1186/s12864-022-08919-5)
Supplement: Supplementary file 2 — Additional file 2: Supplementary Fig. 1. The classification plot of samples from three cell lines based on all the DEGs. Cells were treated with (3, 12, 24 h.p.i.) or without (normal group) ZIKV infection; Supplementary Fig. 2. The expression heatmap of 33 common DEGs among JEG-3, U-251 MG, and HK-2 cells; Supplementary Fig 3. The enriched GO BPs of DEGs from 3 cell lines, including (a) JEG-3, (b) U-251 MG, (c) HK-2 cells. Supplementary Table 1. DEGs in JEG-3 cells of 24h; Supplementary Table 2. DEGs in U-251 MG cells of 24h; Supplementary Table 3. DEGs in HK-2 cells of 24h; Supplementary Table 4. 33 common DEGs and functions in 3 cell lines; Supplementary Table 5. The GO-BP enrichment of DEGs in JEG-3 cells of 24h; Supplementary Table 6. The GO-BP enrichment of up-regulated DEGs in JEG-3 cells of 24h; Supplementary Table 7. The GO-BP enrichment of down-regulated DEGs in JEG-3 cells of 24h; Supplementary Table 8. The GO-BP enrichment of DEGs in U-251 MG cells of 24h; Supplementary Table 9. The GO-BP enrichment of up-regulated DEGs in U-251 MG cells of 24h; Supplementary Table 10. The GO-BP enrichment of down-regulated DEGs in U-251 MG cells of 24h; Supplementary Table 11. The GO-BP enrichment of DEGs in HK-2 cells of 24h; Supplementary Table 12. The GO-BP enrichment of up-regulated DEGs in HK-2 cells of 24h; Supplementary Table 13. The GO-BP enrichment of down-regulated DEGs in HK-2 cells of 24h; Supplementary Table 14. The pathway enrichment of DEGs in JEG-3 cells of 24h; Supplementary Table 15. The pathway enrichment of DEGs in U-251 MG cells of 24h; Supplementary Table 16. The pathway enrichment of DEGs in HK-2 cells of 24h; Supplementary Table 17. DEGs in JEG-3 cells of 3h; Supplementary Table 18. DEGs in JEG-3 cells of 12h; Supplementary Table 19. The GO-BP enrichment of DEGs in JEG-3 cells of 3h; Supplementary Table 20. The GO-BP enrichmentin of up-regulated DEGs in JEG-3 cells of 3h; Supplementary Table 21. The pathway enrichment of DEGs in JEG-3 cells of 3h. [file 12864_2022_8919_MOESM2_ESM.docx]

**Supplementary information for**

**Revealing the characteristics of ZIKV infection through tissue-specific transcriptome sequencing analysis**

Zhi-lu Chen^1, 2, †^, Zuo-jing Yin^1,†^, Tian-yi Qiu^1, 3, †,^ *, Jian Chen^2^, Jian Liu^2^, Xiao-yan Zhang^1, 2, 3,^ *, Jian-qing Xu^1, 2, 3,^ *.

^1^Institutes of Biomedical Sciences, Shanghai Medical College, Fudan University, Shanghai, 200032, China；

^2^Shanghai Public Health Clinical Center, Fudan University, Shanghai, 201508, China;

^3^Department of Immunotherapy & Shanghai Key Laboratory of Organ Transplantation, Zhongshan Hospital, Fudan University, Shanghai 200032, P.R. China.

^†^These authors contributed equally.

Correspondence should be addressed to Tianyi Qiu ([ty_qiu@126.com](mailto:ty_qiu@126.com)), Xiaoyan Zhang ([zhangxiaoyan@fudan.edu.cn](mailto:zhangxiaoyan@fudan.edu.cn)), and Jianqing Xu ([xujianqing@fudan.edu.cn](mailto:xujianqing@fudan.edu.cn))

**Keywords:** *Zika virus, tissue-specific, transcriptome sequencing analysis, Host immune responses, Type I interferon*

**Supplementary Figures**


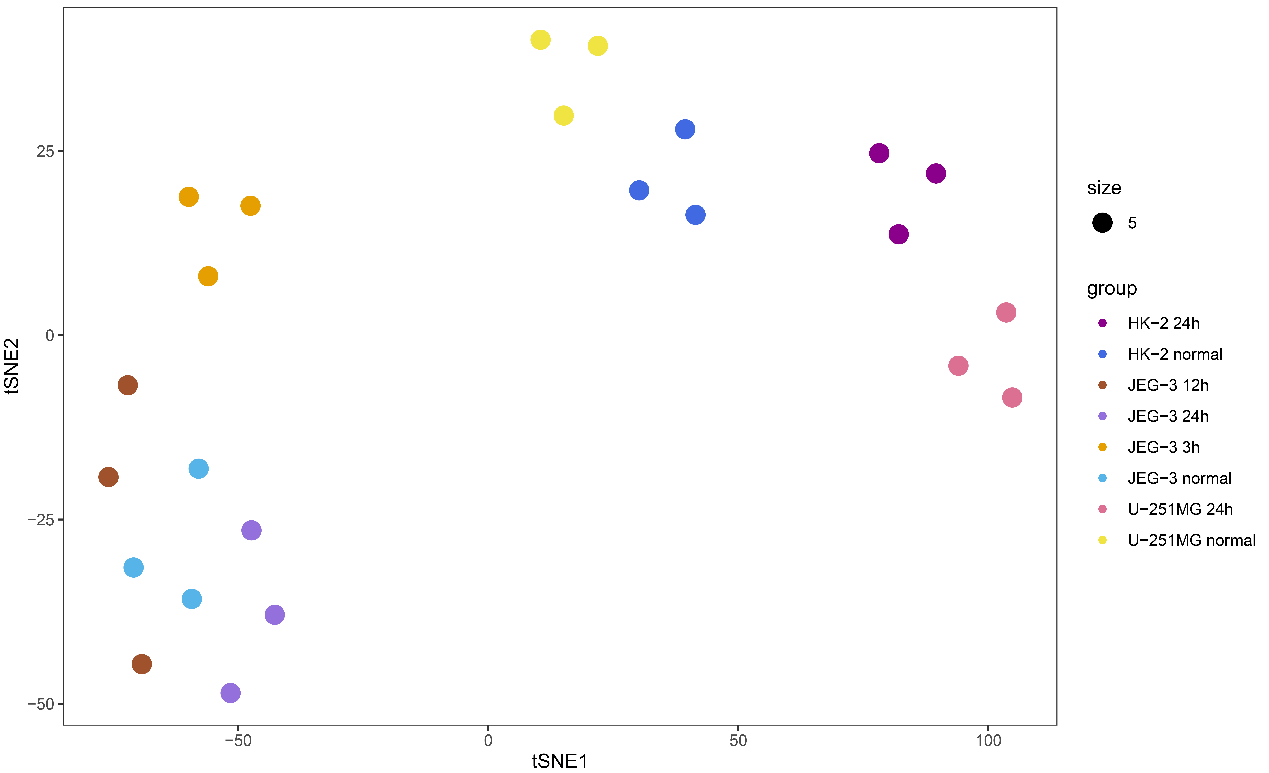


**Supplementary Figure 1. The classification plot of samples from three cell lines based on all the DEGs.** Cells were treated with (3, 12, 24 h.p.i.) or without (normal group) ZIKV infection.


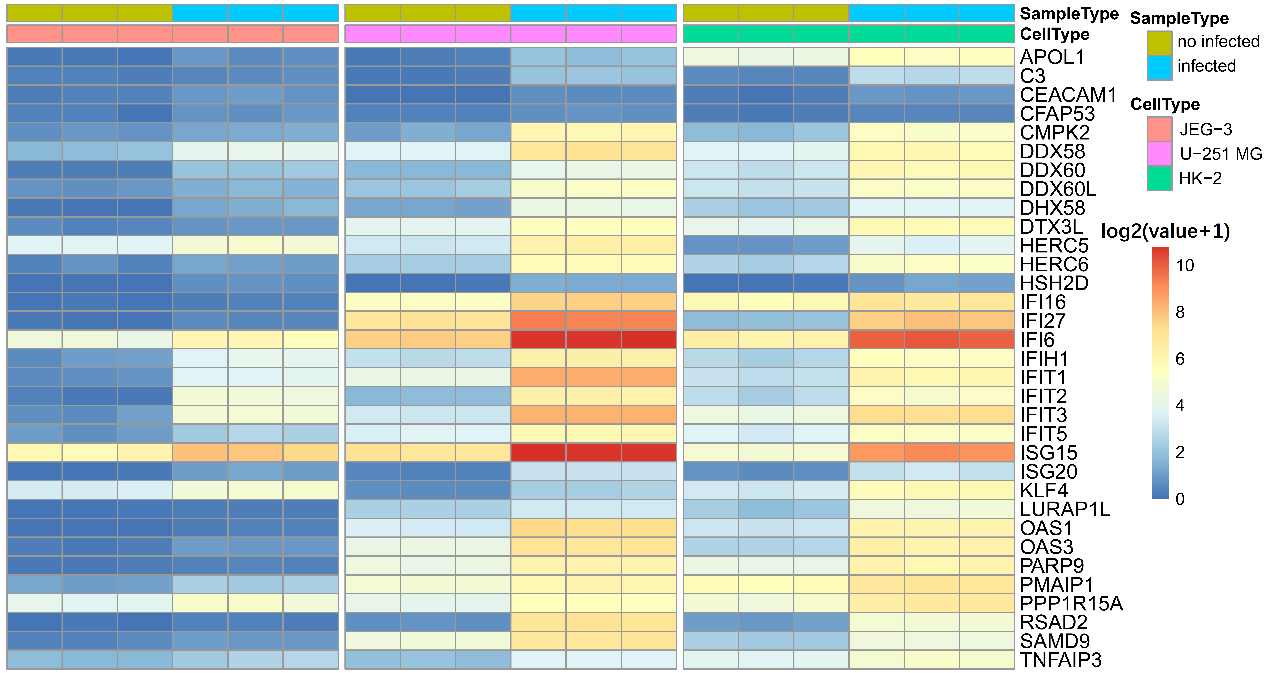


**Supplementary Figure 2**. **The expression heatmap of 33 common DEGs among JEG-3, U-251 MG, and HK-2 cells.** Rows represent genes, and columns represent samples. Samples were obtained from three cell lines, including JEG-3 cells, U-251 MG cells and HK-2 cells, and each cell contains non-infected or ZIKV-infected groups. Three experiments were repeated for each sample. The numerical values in the heatmap represent the logarithm of the expression value + 1.

**
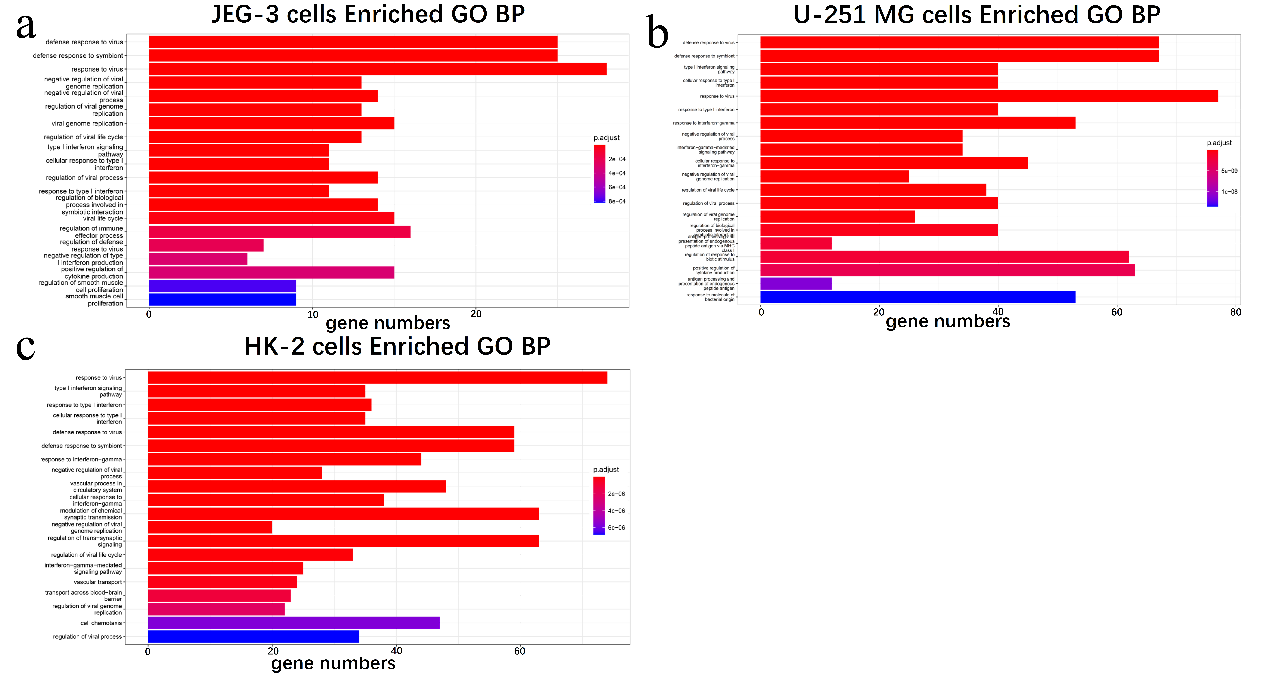
**

**Supplementary Figure 3.** The enriched GO BPs of DEGs from 3 cell lines, including **(a)** JEG-3, **(b)** U-251 MG, **(c)** HK-2 cells.

**Supplementary Tables**

**Supplementary Table 1. DEGs in JEG-3 cells of 24h**

| gene | pvalue | FC | gene | pvalue | FC | gene | pvalue | FC |
| --- | --- | --- | --- | --- | --- | --- | --- | --- |
| POTEG | 0.0068021 | 0.104624 | PARP9 | 0.008214 | 2.383603 | RFTN1 | 0.036936 | 4.978657 |
| HPN | 0.0008299 | 0.124056 | ZC4H2 | 0.0019808 | 2.43494 | LRRC75A | 0.023135 | 4.991346 |
| CATSPERE | 0.0142299 | 0.128276 | APOBEC2 | 0.0436748 | 2.450184 | POLN | 0.047277 | 5.016642 |
| CD22 | 0.0216308 | 0.152791 | PTPRO | 0.0333741 | 2.461625 | SPATC1L | 0.010338 | 5.061943 |
| INPP5J | 0.0195306 | 0.164944 | DDR2 | 0.009933 | 2.509339 | LRRC29 | 0.005464 | 5.192158 |
| CTAGE8 | 0.0075902 | 0.16725 | FIBCD1 | 0.0105061 | 2.520157 | NWD1 | 0.015298 | 5.429117 |
| ZNF221 | 0.0230753 | 0.176576 | STX11 | 0.005423 | 2.528351 | SPRY2 | 0.002534 | 5.446687 |
| CYP1A1 | 0.0256014 | 0.182003 | LYPD5 | 0.026584 | 2.543155 | TPPP | 0.029959 | 5.469811 |
| AC136616.1 | 0.0108361 | 0.197335 | RNF32 | 0.0209252 | 2.545445 | BTBD19 | 0.026813 | 5.483672 |
| ZNF846 | 0.0395855 | 0.245988 | TMPRSS3 | 0.0343769 | 2.547578 | JUN | 0.033891 | 5.722599 |
| SCN1B | 0.0274561 | 0.254272 | C10orf82 | 0.0261403 | 2.558502 | DDX58 | 8.74E-05 | 5.917426 |
| COLQ | 0.0163799 | 0.255848 | HERC6 | 0.022539 | 2.613874 | S1PR5 | 0.020547 | 5.977571 |
| CYP2D6 | 0.0284593 | 0.274064 | TNFSF9 | 0.0185036 | 2.616917 | RSAD2 | 0.017782 | 6.087494 |
| PAPLN | 0.0092069 | 0.285907 | DDX60L | 0.000543 | 2.628123 | EFCAB10 | 0.014555 | 6.760753 |
| CERS3 | 0.0213652 | 0.2866 | TMEM163 | 0.0180454 | 2.650698 | OAS3 | 0.002092 | 6.781775 |
| MT2A | 0.005732 | 0.298455 | MRGPRX1 | 0.0075582 | 2.650842 | NT5DC4 | 0.013622 | 6.913816 |
| ZNF329 | 0.0282104 | 0.304576 | KLF4 | 0.0133915 | 2.664607 | CACNA1S | 0.01688 | 7.071745 |
| CYP2U1 | 0.0353346 | 0.319111 | IFI6 | 0.0115052 | 2.666532 | SVOP | 0.021666 | 7.227817 |
| KIF5C | 0.0459591 | 0.36016 | SYT12 | 0.0204333 | 2.676214 | LURAP1L | 0.008394 | 7.444656 |
| ACTG2 | 0.028248 | 0.362157 | SPANXB1 | 0.0424779 | 2.695993 | SMIM9 | 0.038903 | 7.475825 |
| POTEF | 0.0278301 | 0.362605 | SPAG17 | 0.0026941 | 2.71231 | APOL1 | 0.033569 | 7.503151 |
| PAQR5 | 0.0365084 | 0.391797 | PLXDC1 | 0.0260669 | 2.719326 | CDH4 | 0.000642 | 7.591684 |
| TAGLN | 0.0123898 | 0.392912 | GCA | 0.0096352 | 2.722478 | NR2F1 | 0.031872 | 8.164325 |
| C1orf50 | 0.0166139 | 0.408793 | PPARGC1A | 0.0168306 | 2.72861 | AC068896.1 | 0.047304 | 8.18688 |
| DTWD2 | 0.0036749 | 0.412821 | GJA1 | 0.0032355 | 2.751199 | SPATA32 | 0.047304 | 8.186881 |
| DLG4 | 0.0055287 | 0.413665 | POTEI | 0.0367297 | 2.763104 | 2-Mar | 0.047304 | 8.186881 |
| CCDC134 | 0.0085141 | 0.425652 | MTHFS | 0.0373023 | 2.777463 | TRANK1 | 0.010095 | 8.432079 |
| CARD11 | 0.0406779 | 0.437352 | CES1 | 0.0368407 | 2.835792 | BDKRB2 | 0.00687 | 8.450368 |
| ZNF512 | 0.0124051 | 0.439459 | ANKRD34A | 0.0098087 | 2.858765 | IFI27 | 0.007256 | 8.565738 |
| TUBB2A | 0.0428466 | 0.467141 | SMIM22 | 0.0480446 | 2.85999 | SLC45A3 | 0.026428 | 8.614628 |
| NBEA | 0.0432592 | 0.491321 | ARHGAP30 | 0.0402934 | 2.913777 | KRT17 | 0.026839 | 9.256445 |
| CALML6 | 0.0477892 | 0.494932 | ZFP36 | 0.0384248 | 3.215559 | RARRES3 | 0.031214 | 9.637539 |
| CRISPLD1 | 0.0006481 | 2.011399 | SULT2A1 | 0.0304897 | 3.225748 | KCNV1 | 0.007241 | 9.800581 |
| SLC1A6 | 0.0456199 | 2.016069 | PPM1K | 0.0141026 | 3.229906 | MYO15A | 0.026027 | 10.10702 |
| TMIGD2 | 0.0181612 | 2.019501 | MAOA | 0.0343739 | 3.264965 | OAS1 | 0.015437 | 10.95246 |
| DIO2 | 0.0403657 | 2.033205 | CDC42 | 0.0224291 | 3.366375 | CCDC102B | 0.026745 | 11.01734 |
| PLSCR4 | 0.0270087 | 2.045244 | CHST3 | 0.0427529 | 3.388106 | RND1 | 0.043264 | 11.59962 |
| NR4A3 | 0.0462271 | 2.067417 | HIST1H4E | 0.0227516 | 3.463925 | DAAM2 | 0.016919 | 12.68798 |
| FAM107B | 0.0386941 | 2.071599 | BTN3A1 | 0.0392031 | 3.490021 | MRVI1 | 0.024288 | 12.81425 |
| C3 | 0.0227241 | 2.078208 | AFAP1L2 | 0.0420993 | 3.506081 | AL139142.2 | 0.045069 | 12.99083 |
| HERC5 | 0.0114115 | 2.099158 | POU3F2 | 0.0079763 | 3.563828 | CTSS | 0.049887 | 13.46785 |
| TNFAIP3 | 0.0300837 | 2.118139 | DUSP23 | 0.0462081 | 3.570637 | FAM149A | 0.001359 | 13.6493 |
| PPP1R15A | 0.016582 | 2.12032 | CEACAM1 | 0.0193036 | 3.588594 | DDX60 | 0.0026 | 15.22637 |
| DTX3L | 0.0022459 | 2.156444 | PMAIP1 | 0.0075349 | 3.669569 | ISG20 | 0.011262 | 15.66712 |
| DUSP13 | 0.0256121 | 2.160161 | COLEC11 | 0.0320415 | 3.684103 | IFIH1 | 0.006791 | 15.98012 |
| CERS4 | 0.0053254 | 2.174823 | ISG15 | 0.0169065 | 3.698793 | EMX2 | 0.02411 | 17.73423 |
| APOL2 | 0.0022101 | 2.177652 | ESRRG | 0.0032606 | 3.730669 | IFIT1 | 0.000657 | 20.09326 |
| SEMA3D | 0.006599 | 2.187249 | IFI16 | 0.0059089 | 3.925152 | HSH2D | 0.00222 | 20.15745 |
| CMPK2 | 0.0058509 | 2.212427 | CFAP53 | 0.011188 | 3.946854 | EGR1 | 0.002914 | 22.83682 |
| GAS7 | 0.0254917 | 2.231039 | HESX1 | 0.0056645 | 3.968988 | IL1A | 0.000357 | 23.83098 |
| FUT1 | 0.0269499 | 2.231134 | SYNPO2 | 0.009213 | 4.139504 | IFIT3 | 0.001918 | 34.80977 |
| ZC3HAV1 | 0.019086 | 2.233968 | MDH1B | 0.0115363 | 4.139991 | DHX58 | 0.017066 | 43.68162 |
| ABLIM2 | 0.0306277 | 2.237774 | IL6 | 0.0168468 | 4.206754 | TNF | 0.002841 | 65.16502 |
| FZD4 | 0.0140701 | 2.267503 | ARL4A | 0.0084775 | 4.392189 | RTL9 | 0.031294 | 71.23277 |
| FAM111A | 0.0240909 | 2.305508 | IFIT5 | 0.0068941 | 4.669817 | IFIT2 | 0.000978 | 136.627 |
| SAMD9 | 0.006413 | 2.318724 | OTUD1 | 0.0214185 | 4.68138 | IFNL1 | 0.021629 | 200.1875 |
| TCHH | 0.0312375 | 2.352249 | NAT2 | 0.0436887 | 4.72804 | CCL5 | 0.002065 | 245.0662 |
| CDKN2C | 0.0106893 | 4.848358 | ARHGEF28 | 0.0212233 | 4.75894 | IFNL3 | 0.008029 | 351.0346 |

**Supplementary Table 2. DEGs in U-251 MG cells of 24h**

| gene | pvalue | FC | gene | pvalue | FC | gene | pvalue | FC |
| --- | --- | --- | --- | --- | --- | --- | --- | --- |
| CGB5 | 9.17E-05 | 0.078025 | NFKBIE | 0.000285 | 2.358603 | EPS8L2 | 0.007455 | 3.652801 |
| KRT19 | 0.013241 | 0.097347 | ARID5A | 0.000577 | 2.359103 | ST7-AS1 | 0.032381 | 3.675048 |
| NTF4 | 0.032788 | 0.098566 | ZNF773 | 0.007333 | 2.359597 | BEST1 | 0.004809 | 3.681698 |
| CGB2 | 0.003942 | 0.099222 | LINC01209 | 0.015501 | 2.360247 | ADAMTS14 | 0.004422 | 3.682074 |
| KRT75 | 0.017545 | 0.115614 | NYAP1 | 0.018576 | 2.360963 | RAB20 | 0.000135 | 3.69381 |
| RGS8 | 0.00778 | 0.116124 | IDO1 | 0.012249 | 2.361108 | CRABP2 | 1.72E-05 | 3.694743 |
| CNGA3 | 0.014663 | 0.117857 | IL16 | 0.011338 | 2.36285 | BASP1 | 0.019767 | 3.715559 |
| ALPP | 0.005476 | 0.149529 | FAM117A | 0.000178 | 2.363618 | NKPD1 | 0.006597 | 3.719041 |
| KRT13 | 0.001288 | 0.157117 | EIF4EBP3 | 0.045076 | 2.365801 | DLL1 | 0.004558 | 3.720358 |
| ACKR3 | 0.004952 | 0.158022 | TMEM253 | 0.025143 | 2.367389 | SPATA32 | 0.012763 | 3.724661 |
| UCA1 | 0.000392 | 0.158702 | PELI1 | 0.000188 | 2.36892 | BNIP3 | 0.000202 | 3.729955 |
| CGB8 | 0.001441 | 0.168952 | LAMC2 | 0.001267 | 2.373128 | OGFR | 0.000619 | 3.735993 |
| RPS10-NUDT3 | 0.036706 | 0.179845 | ASPHD2 | 0.008842 | 2.375486 | NR1H4 | 0.005236 | 3.742392 |
| LOC101927481 | 0.036802 | 0.181876 | C16orf86 | 0.01725 | 2.375896 | MIR210HG | 0.001058 | 3.743396 |
| RPL21P28 | 0.029937 | 0.187618 | OVGP1 | 0.029526 | 2.376573 | SYS1-DBNDD2 | 0.003649 | 3.761639 |
| CGB | 0.012413 | 0.193835 | MLC1 | 0.001803 | 2.377138 | BTN3A2 | 0.002189 | 3.764892 |
| SAMD11 | 0.001318 | 0.200418 | DDIT3 | 0.00245 | 2.379023 | ADAP1 | 0.010744 | 3.765184 |
| CGB1 | 0.000225 | 0.201702 | ATP2A1 | 0.031928 | 2.384711 | MUC3A | 0.03508 | 3.766022 |
| KRT81 | 0.001314 | 0.232183 | ZNF878 | 0.003446 | 2.384801 | HES7 | 0.019673 | 3.768472 |
| FAM20A | 0.029488 | 0.240608 | SLC16A6 | 0.006529 | 2.385193 | ZC3H12D | 0.0224 | 3.768784 |
| UG0898H09 | 0.004405 | 0.242749 | MAB21L1 | 0.022994 | 2.38523 | PDE4C | 0.01791 | 3.772691 |
| GABRQ | 0.003446 | 0.246163 | CCDC116 | 0.042498 | 2.385279 | TNFRSF9 | 5.27E-05 | 3.773042 |
| HMCN1 | 0.001812 | 0.253713 | CASQ1 | 0.01329 | 2.386061 | CA12 | 0.00074 | 3.781406 |
| LINC00889 | 0.027471 | 0.254153 | CLIC2 | 0.012049 | 2.386613 | TRIM5 | 0.000603 | 3.800258 |
| TAS2R3 | 0.031324 | 0.258799 | HSD17B7P2 | 0.002467 | 2.388139 | LBH | 1.24E-05 | 3.808182 |
| RHOBTB3 | 5.49E-06 | 0.260937 | SQRDL | 0.001402 | 2.390387 | GRIA1 | 0.030283 | 3.814501 |
| TSSK1B | 0.0257 | 0.262371 | MAFF | 0.002547 | 2.391551 | RAB3D | 0.042619 | 3.81528 |
| PTGES | 0.016846 | 0.271636 | RNF43 | 7.55E-05 | 2.393526 | HS3ST5 | 0.008929 | 3.825711 |
| ANXA2P1 | 0.024698 | 0.272677 | IER3 | 5.87E-05 | 2.393781 | PNPT1 | 0.000504 | 3.840275 |
| ATOH8 | 0.001 | 0.275154 | CCDC184 | 0.007161 | 2.39593 | ARRDC4 | 0.003176 | 3.844758 |
| KRT15 | 0.00354 | 0.276292 | FAM71E1 | 0.004115 | 2.396625 | AVIL | 0.025054 | 3.847562 |
| FOXS1 | 0.021215 | 0.277168 | INHBE | 0.006637 | 2.398339 | ACSL5 | 0.037445 | 3.849511 |
| TAGLN | 0.000424 | 0.286029 | CCT6P1 | 0.006157 | 2.407627 | SLC11A1 | 0.01685 | 3.854676 |
| ARHGDIB | 0.000672 | 0.292401 | TLL2 | 0.01628 | 2.407735 | DDIT4 | 4.84E-05 | 3.856855 |
| GALNT6 | 0.024112 | 0.295917 | HEXDC | 0.001715 | 2.40898 | TFAP2E | 0.023044 | 3.858268 |
| SORL1 | 0.00059 | 0.298395 | DRAXIN | 0.035408 | 2.409783 | LINC00865 | 0.031814 | 3.865215 |
| DLX6-AS1 | 0.006 | 0.299998 | ZNF385B | 0.008413 | 2.412214 | IL23A | 0.013859 | 3.872176 |
| COL6A3 | 0.000923 | 0.307901 | NRN1 | 5.59E-06 | 2.414642 | LOC154761 | 0.001114 | 3.877353 |
| ROS1 | 0.004588 | 0.309868 | LOC101928525 | 0.028697 | 2.414718 | AP3B2 | 6.49E-08 | 3.879435 |
| DAB1 | 0.010453 | 0.311805 | HLA-J | 0.016381 | 2.415312 | MLXIPL | 0.046326 | 3.883705 |
| ISLR | 0.012506 | 0.313222 | MPZ | 0.036516 | 2.416687 | SYT5 | 0.02297 | 3.894914 |
| 4-Mar | 0.004015 | 0.313882 | HOXD4 | 0.008705 | 2.4167 | MYOM3 | 0.013396 | 3.898098 |
| ZNF469 | 0.006224 | 0.315307 | MST1 | 0.008445 | 2.417538 | KMO | 0.014984 | 3.898108 |
| CRYAB | 0.001137 | 0.322949 | HIST1H1C | 0.00805 | 2.418591 | WARS | 0.002387 | 3.902844 |
| HIPK2 | 0.004174 | 0.326337 | C19orf38 | 0.0297 | 2.41894 | MAPK8IP2 | 0.02728 | 3.909754 |
| KRT7 | 0.0413 | 0.327398 | PPFIA3 | 4.36E-05 | 2.420514 | WEE2-AS1 | 0.00218 | 3.91308 |
| LINC01561 | 0.044979 | 0.332999 | TBC1D3C | 0.021405 | 2.420766 | LINC00602 | 0.046324 | 3.915291 |
| FAM81A | 2.27E-05 | 0.34408 | LMNTD2 | 0.022437 | 2.422194 | LOC101929372 | 0.020311 | 3.91658 |
| PPAPDC1A | 0.035531 | 0.344868 | TMEM198 | 0.016304 | 2.42297 | TGM4 | 0.017067 | 3.923227 |
| MEGF6 | 0.000663 | 0.347393 | GRINA | 8.01E-05 | 2.423805 | ACSM3 | 0.029561 | 3.929152 |
| SLC16A7 | 0.004522 | 0.350246 | MIR503HG | 0.017431 | 2.425155 | HLF | 0.012789 | 3.933123 |
| PALM3 | 0.002152 | 0.352265 | TRIM25 | 0.001267 | 2.426184 | DRD4 | 0.023974 | 3.964835 |
| FAM106A | 0.007902 | 0.360117 | CLDN1 | 4.51E-06 | 2.426925 | YPEL4 | 0.03981 | 3.990281 |
| SLIT3 | 0.000147 | 0.360171 | MAP3K8 | 0.000819 | 2.428709 | SOWAHD | 0.048849 | 3.990418 |
| CNBD2 | 0.028991 | 0.364805 | F3 | 0.000388 | 2.429688 | ACHE | 0.000414 | 3.996309 |
| TMOD2 | 4.12E-05 | 0.367555 | SAXO2 | 0.031275 | 2.430022 | RRAD | 0.012448 | 3.997552 |
| DCLK1 | 0.000145 | 0.367573 | TREX1 | 0.003861 | 2.430993 | FOS | 0.026186 | 3.998528 |
| ANKRD36BP1 | 0.003145 | 0.3746 | COL4A3 | 0.001002 | 2.432488 | ADGRE1 | 0.003388 | 4.002549 |
| DANCR | 0.000146 | 0.375157 | HIST3H2A | 0.000139 | 2.435353 | GSTO2 | 0.020839 | 4.008862 |
| LINC01132 | 0.013392 | 0.376839 | ARC | 0.044728 | 2.436496 | ALB | 0.018673 | 4.01501 |
| REREP3 | 0.011187 | 0.378213 | NRN1L | 0.043596 | 2.438692 | PARP14 | 0.00132 | 4.044071 |
| MBNL3 | 0.003523 | 0.379226 | COPZ2 | 0.017972 | 2.439861 | MUM1L1 | 0.039798 | 4.049327 |
| PADI3 | 0.002157 | 0.380637 | ASGR1 | 7.01E-05 | 2.440508 | REC8 | 0.005293 | 4.054506 |
| SEMA3F | 0.0219 | 0.383465 | LOC101928841 | 0.015212 | 2.441038 | BAHCC1 | 0.010957 | 4.067735 |
| FAM106B | 0.031585 | 0.384777 | SNHG15 | 0.010558 | 2.441183 | PLAC8 | 0.001404 | 4.092313 |
| ID1 | 0.001978 | 0.384897 | IER2 | 0.001225 | 2.441968 | LOC100507006 | 0.01593 | 4.092939 |
| MAL | 0.02734 | 0.385456 | GBP2 | 0.000282 | 2.447998 | APOL2 | 0.000323 | 4.097242 |
| SLC4A8 | 0.000832 | 0.385672 | ELFN1 | 0.008137 | 2.448168 | DDIT4L | 0.005115 | 4.112269 |
| LPP | 0.003745 | 0.38578 | C5AR1 | 0.011267 | 2.449414 | MUC6 | 0.040915 | 4.123181 |
| TRHDE | 0.000497 | 0.385805 | LINC01198 | 0.00687 | 2.449801 | NT5E | 0.003805 | 4.12413 |
| CFAP126 | 0.023737 | 0.387976 | HAS1 | 0.044354 | 2.449963 | BBC3 | 0.000294 | 4.127979 |
| CNR1 | 1.14E-05 | 0.388399 | FAM69B | 0.031683 | 2.451132 | IL4I1 | 8.53E-05 | 4.132301 |
| AHNAK | 0.001103 | 0.388513 | GRAMD1C | 0.045341 | 2.45174 | NUPR1 | 0.006188 | 4.136626 |
| APCDD1L | 0.019087 | 0.392601 | CILP2 | 0.041806 | 2.452775 | FBXO6 | 0.032851 | 4.140105 |
| SACS | 0.001389 | 0.39291 | SYTL2 | 0.001477 | 2.453748 | TNFAIP3 | 2.21E-06 | 4.144984 |
| LONRF2 | 0.001189 | 0.394106 | PVT1 | 0.000508 | 2.455743 | N4BP2L1 | 0.040133 | 4.149863 |
| CCDC144B | 0.004004 | 0.397478 | EFCAB5 | 0.004858 | 2.457333 | CACNG8 | 0.005417 | 4.17504 |
| THRIL | 0.00086 | 0.398375 | TMEM254-AS1 | 0.019863 | 2.457694 | CCDC154 | 0.041991 | 4.180305 |
| SYCP2 | 0.013293 | 0.399291 | SRCIN1 | 0.002952 | 2.459215 | LAP3 | 7.84E-06 | 4.188284 |
| PODXL | 1.73E-06 | 0.402725 | FITM1 | 0.01486 | 2.460442 | PTPN6 | 0.019829 | 4.193127 |
| SOX18 | 0.000553 | 0.402926 | PKLR | 0.049782 | 2.460848 | PLCH2 | 0.018656 | 4.194662 |
| FREM2 | 0.007718 | 0.404941 | ERCC6-PGBD3 | 0.008672 | 2.461449 | TNXA | 0.005428 | 4.196286 |
| CDH5 | 0.002535 | 0.405786 | MST1L | 0.017018 | 2.464482 | CETN4P | 0.015816 | 4.203478 |
| FBN2 | 8.01E-06 | 0.41086 | C1orf106 | 0.000439 | 2.466566 | SPANXN1 | 0.034331 | 4.217796 |
| PCDHGA8 | 0.007171 | 0.414325 | AGRN | 0.005222 | 2.469179 | RCAN2 | 0.0087 | 4.221895 |
| POMK | 0.023072 | 0.41632 | PSME1 | 0.003845 | 2.471697 | GOLGA7B | 0.00768 | 4.223293 |
| TCF7 | 0.002032 | 0.419313 | ZNF709 | 0.000989 | 2.475044 | AS3MT | 0.041035 | 4.227221 |
| DCHS2 | 0.004316 | 0.419913 | CHGB | 8.10E-05 | 2.475952 | SCN3A | 0.00051 | 4.227771 |
| ACO1 | 0.000337 | 0.420884 | CHST15 | 0.003528 | 2.477181 | ECM2 | 0.016571 | 4.275347 |
| TBC1D5 | 0.001602 | 0.421647 | LRRTM2 | 0.004331 | 2.478124 | SOST | 0.00208 | 4.281792 |
| ADAM12 | 3.09E-05 | 0.422625 | TSC22D3 | 1.67E-05 | 2.479714 | IFITM2 | 0.006332 | 4.28397 |
| AFP | 0.042378 | 0.423413 | PLEKHF1 | 0.000212 | 2.480031 | MTVR2 | 0.018818 | 4.286445 |
| CCDC80 | 4.84E-05 | 0.424875 | MAP7D2 | 0.020523 | 2.484081 | TMEM51-AS1 | 0.001561 | 4.291822 |
| DIO2 | 0.00317 | 0.425117 | EIF2AK2 | 0.00739 | 2.48492 | KYNU | 0.026441 | 4.298233 |
| CLMP | 0.023719 | 0.425635 | TMEM171 | 0.013707 | 2.485486 | TXLNB | 0.001216 | 4.301652 |
| KSR2 | 0.000353 | 0.428074 | SLC2A3 | 0.001984 | 2.485744 | EGLN3 | 0.000111 | 4.314332 |
| KIAA1549L | 0.026066 | 0.428622 | NAPA | 6.36E-06 | 2.488144 | ZNF442 | 0.000132 | 4.314359 |
| ABCB11 | 0.034503 | 0.429421 | GLS2 | 0.043075 | 2.490126 | PYY | 0.012407 | 4.316075 |
| PSD4 | 0.00268 | 0.429658 | ITGB2 | 0.04622 | 2.490661 | ANGPT2 | 0.000805 | 4.323452 |
| LINC01279 | 0.006632 | 0.430355 | LOC554206 | 0.028137 | 2.493506 | AKNAD1 | 0.01368 | 4.325929 |
| OLIG1 | 0.028963 | 0.433518 | TPTE2 | 0.04852 | 2.493717 | SPDEF | 0.000122 | 4.327605 |
| LEPR | 0.000275 | 0.436149 | CD99P1 | 0.004961 | 2.497261 | ZNF625-ZNF20 | 0.029034 | 4.341146 |
| ASTN1 | 0.004416 | 0.436374 | SLC25A28 | 2.86E-05 | 2.497492 | CFAP43 | 0.0003 | 4.349864 |
| KCNC4 | 0.003426 | 0.436374 | ALS2CR12 | 0.003079 | 2.498654 | STAT2 | 0.000283 | 4.360536 |
| CYP27C1 | 0.012837 | 0.436552 | ZNF606 | 0.002929 | 2.498745 | FCGR1B | 0.002812 | 4.361244 |
| AKR1B10 | 0.003563 | 0.44037 | CYB5R2 | 0.031994 | 2.499928 | NCF2 | 0.005838 | 4.363686 |
| DPYSL2 | 0.000385 | 0.441023 | UBE2F-SCLY | 0.000521 | 2.502707 | B2M | 0.000432 | 4.366648 |
| TMEM19 | 0.000368 | 0.441535 | TNFRSF13C | 0.001753 | 2.504921 | SAT1 | 0.000381 | 4.383228 |
| CERK | 0.000285 | 0.442746 | C2orf72 | 0.01439 | 2.505576 | PPM1K | 0.002235 | 4.389756 |
| SOGA1 | 0.001362 | 0.442815 | KCNE2 | 0.018659 | 2.506084 | GOLGA6A | 0.033262 | 4.390491 |
| AR | 0.000891 | 0.444228 | LOC100132057 | 0.005073 | 2.507052 | WDR93 | 0.011579 | 4.392329 |
| FAM101B | 0.002554 | 0.444314 | PAPPA2 | 0.007823 | 2.509506 | PHF11 | 0.000191 | 4.401849 |
| CBX5 | 0.001177 | 0.445435 | TG | 0.035838 | 2.51163 | CDK18 | 0.000101 | 4.41567 |
| URGCP-MRPS24 | 0.029478 | 0.445532 | MDK | 0.004744 | 2.51184 | CEMIP | 0.00169 | 4.421792 |
| MAP2K6 | 0.043559 | 0.447503 | RELB | 0.000302 | 2.513244 | CDH22 | 0.036303 | 4.424187 |
| ITGBL1 | 2.12E-06 | 0.452676 | OSGIN1 | 0.001843 | 2.513335 | LRRC16B | 0.047642 | 4.425019 |
| CCDC144CP | 0.048647 | 0.452921 | ZNF625 | 0.008891 | 2.516303 | FLJ22447 | 0.019124 | 4.440194 |
| FITM2 | 0.001622 | 0.453727 | EPHB1 | 0.012833 | 2.521035 | TMEM27 | 0.001138 | 4.452602 |
| CCDC144A | 0.009204 | 0.454448 | NR4A3 | 0.029252 | 2.522992 | SP100 | 0.000543 | 4.464927 |
| KCTD12 | 0.001125 | 0.45576 | MTUS2 | 0.004355 | 2.523892 | GCH1 | 8.29E-06 | 4.487745 |
| SAP30L-AS1 | 0.014264 | 0.456346 | ZC3HAV1 | 0.000388 | 2.524176 | FOXA3 | 0.001021 | 4.515779 |
| ENG | 0.006859 | 0.45742 | MILR1 | 0.012808 | 2.526207 | C1R | 0.001579 | 4.524482 |
| RAB3B | 1.22E-05 | 0.459717 | SLC3A1 | 0.046585 | 2.526913 | SAMHD1 | 0.001747 | 4.53297 |
| MMP16 | 0.011306 | 0.460251 | AMY2B | 0.000545 | 2.529093 | MYO1A | 0.019415 | 4.585675 |
| FGFRL1 | 0.007485 | 0.460703 | HEXA-AS1 | 0.015234 | 2.529271 | ZNFX1 | 1.77E-06 | 4.588485 |
| VIT | 8.79E-05 | 0.460993 | TYMP | 0.017053 | 2.530824 | HEPH | 0.000167 | 4.601403 |
| SFT2D2 | 0.016842 | 0.462314 | RAI2 | 0.00867 | 2.531772 | CPA2 | 0.045689 | 4.604023 |
| SLC12A8 | 0.019725 | 0.462959 | LOC100130705 | 0.000481 | 2.532156 | GBP3 | 1.31E-05 | 4.626677 |
| ATP2B4 | 3.34E-05 | 0.463715 | CREBRF | 0.006397 | 2.533226 | TCAF2P1 | 1.77E-05 | 4.629292 |
| MICAL2 | 0.000222 | 0.464208 | MFI2 | 0.003509 | 2.53509 | SLC6A13 | 0.036559 | 4.633274 |
| KDR | 4.90E-06 | 0.464256 | MGP | 0.016997 | 2.535635 | FAM167A | 0.002553 | 4.659574 |
| FLG2 | 0.006021 | 0.467965 | ANKRD31 | 0.009303 | 2.536987 | NR1D1 | 0.000539 | 4.669093 |
| SERTAD4-AS1 | 0.003325 | 0.468047 | MYH3 | 0.000275 | 2.542015 | TAL2 | 0.033981 | 4.69064 |
| SLC4A4 | 0.002625 | 0.469352 | SYT2 | 0.010074 | 2.54763 | IFITM3 | 0.00491 | 4.703829 |
| GPR161 | 0.006321 | 0.469886 | RHOXF1-AS1 | 0.01633 | 2.549351 | TIE1 | 0.002171 | 4.707095 |
| LINC00342 | 0.020718 | 0.471871 | CITED2 | 1.42E-05 | 2.549991 | TCAF2 | 9.55E-06 | 4.707973 |
| LOC100129461 | 0.027717 | 0.4722 | PIH1D2 | 0.023064 | 2.551849 | GPD1 | 0.013317 | 4.72832 |
| TLR8-AS1 | 0.03119 | 0.473069 | ZNF627 | 0.004329 | 2.55843 | CHRM5 | 0.005053 | 4.736939 |
| CYP4F11 | 0.028364 | 0.473091 | PER1 | 0.005937 | 2.559237 | SERPINB9P1 | 0.044953 | 4.739741 |
| ERMP1 | 0.000299 | 0.47476 | PAN3-AS1 | 0.00762 | 2.559882 | IFI16 | 8.41E-05 | 4.753685 |
| TMPPE | 0.036574 | 0.475259 | HIST1H2AI | 0.007422 | 2.560242 | SAMD9 | 0.002723 | 4.757187 |
| KRT8 | 0.000326 | 0.475443 | MUC20 | 0.007764 | 2.562262 | PTP4A3 | 0.014097 | 4.772678 |
| GFAP | 0.001188 | 0.478067 | UNC13A | 0.022023 | 2.564757 | PLA2G4E-AS1 | 0.046503 | 4.783956 |
| SCARA3 | 0.000827 | 0.478397 | PIWIL2 | 0.030481 | 2.572834 | ENKUR | 0.005842 | 4.786594 |
| SEMA5A | 0.000635 | 0.478771 | ENAM | 0.033609 | 2.574311 | C3orf14 | 0.007775 | 4.790271 |
| PRR11 | 0.000131 | 0.479324 | CTH | 0.000342 | 2.576839 | RELN | 0.04299 | 4.792395 |
| SAMD5 | 0.023965 | 0.481886 | MYH15 | 0.000732 | 2.578027 | FRZB | 0.038696 | 4.795638 |
| AHNAK2 | 0.004039 | 0.48189 | IL1A | 0.013701 | 2.580853 | TAPBPL | 0.011304 | 4.801391 |
| ATP8B1 | 0.007656 | 0.48201 | IGFBP4 | 3.47E-06 | 2.582496 | CD22 | 0.009018 | 4.806611 |
| ZNF488 | 0.024552 | 0.485843 | BTBD11 | 0.045462 | 2.584605 | FSTL5 | 0.009312 | 4.84632 |
| PRKAR2A | 0.002165 | 0.488034 | TMEM62 | 0.002538 | 2.587904 | VGLL2 | 0.001108 | 4.852325 |
| NACC2 | 0.000682 | 0.488349 | SMPDL3B | 0.008211 | 2.592654 | TUBAL3 | 0.008782 | 4.852474 |
| CDON | 0.001965 | 0.488761 | CYTH1 | 0.001026 | 2.597025 | LOC101927666 | 0.047659 | 4.858153 |
| GATSL2 | 0.004929 | 0.491022 | TMEM132A | 0.002986 | 2.598312 | IFI30 | 0.000643 | 4.868634 |
| TENM3 | 0.000179 | 0.491229 | GREM2 | 0.01433 | 2.598676 | CPXM1 | 0.022302 | 4.887714 |
| NIN | 0.000138 | 0.49146 | KCNMB2 | 0.034089 | 2.599903 | SPEF1 | 0.000211 | 4.889539 |
| ILDR2 | 0.000259 | 0.492332 | HOXB5 | 4.58E-06 | 2.599903 | DDO | 0.000683 | 4.891699 |
| SLC26A2 | 0.004694 | 0.493053 | WDR63 | 0.035103 | 2.608093 | ALOXE3 | 7.80E-05 | 4.895995 |
| KLF12 | 0.001309 | 0.493347 | DNAH10 | 0.002942 | 2.610256 | SEC16B | 0.030486 | 4.899856 |
| EGFR | 0.004651 | 0.495189 | ZNF425 | 0.00015 | 2.611132 | CYP2J2 | 0.000623 | 4.903272 |
| MYO7B | 0.039712 | 0.496347 | CHRNE | 0.037886 | 2.616883 | EXTL1 | 0.004303 | 4.912921 |
| CASC5 | 0.000985 | 0.496426 | FHAD1 | 0.012271 | 2.61974 | FLT3LG | 0.004142 | 4.945274 |
| NEGR1 | 0.00014 | 0.497209 | LOC101926911 | 0.049837 | 2.620138 | CYP21A2 | 0.015577 | 4.947477 |
| DOK6 | 0.025671 | 0.498498 | MYLK3 | 0.008826 | 2.621159 | NIM1K | 0.000145 | 4.948536 |
| HIP1 | 0.001614 | 0.499865 | CHAC1 | 0.000231 | 2.623575 | LOC100505530 | 0.028889 | 4.95215 |
| TUBB4A | 0.000257 | 2.001669 | HIP1R | 0.000727 | 2.625366 | PSMB8 | 0.000399 | 4.957612 |
| ANKZF1 | 5.01E-05 | 2.002699 | PDK1 | 0.000102 | 2.627251 | DMBT1 | 0.00198 | 4.961714 |
| HEXIM2 | 0.002614 | 2.003494 | VPS9D1-AS1 | 0.001858 | 2.632078 | LOC643201 | 0.048588 | 4.962845 |
| CDKL3 | 0.030131 | 2.004222 | NOCT | 0.00085 | 2.636017 | TNFSF10 | 1.97E-06 | 5.009476 |
| CCDC71L | 2.82E-06 | 2.009056 | CDKN1A | 0.002822 | 2.63821 | TNFRSF10D | 0.047308 | 5.013627 |
| SMPD3 | 0.018572 | 2.01036 | ALDH1L1 | 0.049147 | 2.639024 | LTK | 0.018697 | 5.019929 |
| TRPM3 | 0.045595 | 2.011759 | LOC221946 | 0.006406 | 2.642036 | ATF3 | 0.000122 | 5.023108 |
| TSGA10 | 0.008687 | 2.011973 | ZFYVE28 | 0.001335 | 2.643578 | IFIT5 | 0.001758 | 5.03851 |
| LINC00173 | 0.002951 | 2.012775 | CACNG7 | 0.043752 | 2.645388 | LINC00473 | 0.000212 | 5.048459 |
| LINC01003 | 0.002816 | 2.012781 | C11orf96 | 0.01026 | 2.645951 | FCRLA | 0.001649 | 5.056764 |
| P4HA1 | 0.006249 | 2.012975 | OLFM2 | 0.006313 | 2.648142 | IFI27 | 0.001274 | 5.066335 |
| JPH4 | 0.008548 | 2.01307 | NPIPB8 | 0.006064 | 2.65213 | TMEM151B | 0.037478 | 5.094313 |
| LRP3 | 0.003787 | 2.013445 | NFASC | 0.016045 | 2.65443 | SP8 | 3.21E-05 | 5.104208 |
| CNP | 0.000404 | 2.014161 | APOBEC3D | 9.04E-06 | 2.65534 | C8orf46 | 0.000178 | 5.125173 |
| LURAP1L | 1.41E-05 | 2.014239 | TTC21A | 0.002325 | 2.655819 | SLC6A15 | 0.032749 | 5.132111 |
| GLIPR1L2 | 0.00421 | 2.015277 | IFITM10 | 0.000547 | 2.656106 | MATN1 | 0.010208 | 5.145775 |
| TMPRSS9 | 0.011904 | 2.017454 | CACNA1G | 0.00537 | 2.658369 | HLA-A | 0.000125 | 5.146984 |
| SEPT7P2 | 0.000551 | 2.017708 | BEND5 | 0.009347 | 2.659989 | NCAN | 0.037155 | 5.155901 |
| CMTR1 | 0.000687 | 2.017781 | KCNIP1 | 0.006507 | 2.662449 | HLA-H | 0.001894 | 5.157459 |
| FAM86HP | 0.030162 | 2.018473 | GPR146 | 0.001199 | 2.666198 | NCF1 | 0.000172 | 5.160318 |
| CERS1 | 0.000226 | 2.018559 | CEBPA | 0.00996 | 2.672885 | OPRD1 | 0.046498 | 5.185647 |
| FOXD4L1 | 0.019162 | 2.01891 | DCDC2B | 0.032981 | 2.674827 | FAM181B | 0.001415 | 5.187047 |
| FOXD4 | 0.017281 | 2.021163 | PRR16 | 0.002973 | 2.676433 | LTA | 0.030529 | 5.199029 |
| TRIM46 | 0.032868 | 2.021543 | C1orf162 | 0.000765 | 2.677202 | FES | 0.018562 | 5.204571 |
| PANO1 | 0.005678 | 2.021977 | PSORS1C1 | 0.007005 | 2.677697 | HRASLS2 | 0.003903 | 5.211098 |
| SYTL3 | 0.008822 | 2.022902 | LOC100131496 | 0.047378 | 2.679943 | OOSP1 | 0.031239 | 5.231147 |
| RNF112 | 0.035221 | 2.023563 | TRIM67 | 0.020861 | 2.681405 | SCRT1 | 0.031764 | 5.250452 |
| ZNF20 | 0.021334 | 2.024146 | COL11A2 | 0.006518 | 2.681767 | S100A14 | 0.043776 | 5.250856 |
| HIST1H2AC | 0.003431 | 2.024274 | ITIH4 | 0.000111 | 2.683127 | DMGDH | 3.14E-05 | 5.255491 |
| LOC728730 | 0.014629 | 2.025324 | MYLIP | 1.33E-05 | 2.687586 | OTUD1 | 0.008937 | 5.256643 |
| MMP19 | 0.031639 | 2.025498 | VLDLR-AS1 | 0.033871 | 2.691962 | DIRC3 | 2.30E-05 | 5.260333 |
| ZNF461 | 0.007979 | 2.028546 | FCGR2A | 0.020861 | 2.692975 | SMIM24 | 0.048384 | 5.263428 |
| ZNF596 | 0.012833 | 2.029229 | LOC100506606 | 0.032768 | 2.693746 | NKD1 | 0.00332 | 5.277631 |
| SH3GL2 | 0.009839 | 2.02932 | IL7 | 0.001291 | 2.694138 | RGAG1 | 1.44E-06 | 5.296371 |
| ARHGEF2 | 3.42E-06 | 2.031355 | AURKC | 0.013118 | 2.694871 | CARD16 | 0.015828 | 5.298318 |
| DNAJB9 | 0.003069 | 2.03244 | GAS6-AS2 | 0.00161 | 2.697424 | FAM182A | 0.00106 | 5.388349 |
| GOLGA6L4 | 0.022788 | 2.032787 | PWWP2B | 0.003338 | 2.69858 | ADM | 1.54E-05 | 5.391116 |
| PLCB4 | 0.013851 | 2.033389 | GADD45A | 0.001168 | 2.70166 | GMPR | 0.002961 | 5.42204 |
| PRR22 | 0.043575 | 2.034418 | LOC100996351 | 0.044325 | 2.702504 | KCNH8 | 0.022878 | 5.440955 |
| RNA45S5 | 0.048891 | 2.035041 | ZNF474 | 0.015496 | 2.703823 | IL17RE | 0.005271 | 5.454968 |
| NOMO3 | 0.039337 | 2.035356 | GGT1 | 0.000517 | 2.704447 | BTN3A1 | 5.43E-06 | 5.471393 |
| SEMA3B | 0.001555 | 2.036437 | SSTR2 | 0.003242 | 2.70542 | DUOX2 | 0.010163 | 5.475892 |
| ERAP1 | 5.32E-05 | 2.036988 | PRLR | 0.001631 | 2.706657 | IL12A | 0.008146 | 5.485882 |
| PRKRIP1 | 0.000249 | 2.038611 | IGFBP7 | 0.000146 | 2.706946 | TNFSF8 | 0.012843 | 5.489138 |
| CDC14A | 0.010903 | 2.0388 | LDB3 | 0.035976 | 2.710944 | SDK2 | 0.017206 | 5.499302 |
| CHRD | 0.001946 | 2.039559 | HOXB4 | 0.016467 | 2.716066 | SRD5A3-AS1 | 0.001341 | 5.538312 |
| FLG | 0.002129 | 2.039658 | CIART | 0.001788 | 2.720863 | CXCL3 | 0.01222 | 5.596462 |
| NPAP1 | 0.04944 | 2.039958 | RIMKLA | 0.004507 | 2.727651 | NGFR | 0.000121 | 5.618908 |
| HIST1H3E | 0.032086 | 2.041131 | ZNF433 | 0.00023 | 2.729174 | TFR2 | 0.0243 | 5.624319 |
| THUMPD2 | 0.002929 | 2.04116 | LINGO3 | 0.04409 | 2.730759 | RNF5P1 | 0.030116 | 5.629134 |
| GSDMD | 0.00536 | 2.043045 | TGM1 | 0.001512 | 2.734407 | RFPL1S | 0.013111 | 5.641176 |
| TTPA | 0.003717 | 2.044063 | BEX1 | 0.036995 | 2.734837 | C11orf86 | 0.042448 | 5.641486 |
| DACT3 | 0.013401 | 2.044267 | CD68 | 0.000637 | 2.738403 | HOTTIP | 0.022635 | 5.645162 |
| RAET1G | 0.004379 | 2.045706 | PLIN1 | 0.003725 | 2.739199 | FCGR1A | 0.030562 | 5.657486 |
| SCG5 | 0.019031 | 2.046213 | TRIM74 | 0.001209 | 2.739686 | HTR6 | 0.004905 | 5.658842 |
| TBKBP1 | 0.015518 | 2.046444 | FAM131C | 0.03347 | 2.74512 | CEACAM22P | 0.004117 | 5.660777 |
| BTN2A3P | 0.014657 | 2.04714 | TMEM45A | 0.000444 | 2.74586 | GRID1 | 0.044295 | 5.667413 |
| FAM229A | 0.015433 | 2.04808 | SPTBN5 | 0.000375 | 2.749159 | TXNIP | 0.000664 | 5.685399 |
| ZNF610 | 0.02764 | 2.04816 | ADCY10 | 0.001158 | 2.750316 | OR7D2 | 0.041719 | 5.686654 |
| LIN37 | 0.013122 | 2.049411 | HIST1H2BC | 0.035506 | 2.751026 | NDRG1 | 0.000228 | 5.691057 |
| LOC646626 | 0.022769 | 2.049589 | SH3TC1 | 0.01803 | 2.75987 | LOC101929679 | 0.048919 | 5.735019 |
| NKX6-1 | 0.025233 | 2.049786 | SLC10A4 | 0.032637 | 2.762217 | TNXB | 0.008076 | 5.755774 |
| NFKBIL1 | 0.001117 | 2.050073 | ZNF763 | 0.000102 | 2.764414 | GPRC5C | 0.04321 | 5.761647 |
| STC1 | 5.16E-05 | 2.050615 | NT5C3A | 0.000986 | 2.765946 | RASL10A | 0.038404 | 5.768436 |
| KCNAB2 | 0.000352 | 2.052173 | CHRNB1 | 0.000444 | 2.766727 | GPR62 | 0.004395 | 5.77172 |
| APOBR | 0.031888 | 2.052206 | LRGUK | 0.040853 | 2.773994 | MIR155HG | 0.043931 | 5.804927 |
| MACROD1 | 7.92E-05 | 2.052243 | CD14 | 0.026717 | 2.776322 | NUPR1L | 0.035529 | 5.815096 |
| PDZRN3 | 0.003019 | 2.052898 | C4orf19 | 0.000166 | 2.777742 | C1S | 0.015194 | 5.819242 |
| CX3CL1 | 0.000338 | 2.055588 | LINC01024 | 0.026436 | 2.778964 | CTAGE15 | 0.020136 | 5.827321 |
| EID2 | 0.007005 | 2.055888 | RHBDD3 | 0.00036 | 2.780373 | CXCR5 | 0.043573 | 5.830965 |
| USP25 | 3.73E-05 | 2.056389 | CD34 | 0.009047 | 2.788932 | MATN4 | 0.005237 | 5.886901 |
| ABHD17C | 0.001003 | 2.057588 | NTNG2 | 0.003927 | 2.788955 | ZMAT4 | 0.003653 | 5.901146 |
| SCART1 | 0.002101 | 2.057694 | RILP | 0.006336 | 2.791822 | SP110 | 0.000155 | 5.926466 |
| ABHD16B | 0.019298 | 2.057833 | ADAM11 | 0.012897 | 2.794127 | PLEK | 0.048162 | 5.935621 |
| HIST1H2BK | 0.000923 | 2.058023 | CITED4 | 0.003036 | 2.796487 | STAT1 | 0.000419 | 5.977765 |
| RGMB-AS1 | 0.000517 | 2.058293 | LOC101929066 | 0.026647 | 2.805949 | SOWAHB | 0.025676 | 6.016668 |
| NPIPB9 | 0.007457 | 2.059081 | STRC | 0.019511 | 2.809135 | DNM1P46 | 0.007421 | 6.018412 |
| SEMA7A | 0.00302 | 2.059605 | PINLYP | 0.004924 | 2.809756 | FOSB | 0.040243 | 6.027987 |
| LTF | 0.033051 | 2.06006 | FAM227A | 0.007504 | 2.813469 | SAMD9L | 0.00122 | 6.038412 |
| ROR2 | 4.21E-05 | 2.061586 | TINCR | 0.014275 | 2.814928 | CECR7 | 0.021849 | 6.042989 |
| IGDCC4 | 0.011876 | 2.062646 | GIPR | 0.012759 | 2.82043 | CTXN2 | 0.02873 | 6.047365 |
| FBXO16 | 0.010804 | 2.062855 | ARHGAP4 | 0.001447 | 2.82219 | GLRA2 | 0.000168 | 6.077649 |
| LOC400043 | 0.000373 | 2.064503 | ZNF441 | 0.005551 | 2.829083 | IRF1 | 0.001205 | 6.092289 |
| TTLL3 | 0.003454 | 2.065216 | SUGT1P1 | 0.010235 | 2.830678 | C3orf20 | 0.00184 | 6.107427 |
| LINC00312 | 0.031183 | 2.06667 | GDF15 | 0.000455 | 2.831255 | CD27-AS1 | 0.00104 | 6.125446 |
| ADPRM | 0.004547 | 2.06762 | CEBPB-AS1 | 0.015835 | 2.831418 | TAP2 | 0.000417 | 6.136184 |
| C2orf27A | 0.000467 | 2.068105 | AOC2 | 0.000115 | 2.833289 | PAX2 | 0.012519 | 6.17867 |
| LOC254896 | 0.012909 | 2.06835 | PDCD1LG2 | 0.001022 | 2.836544 | SHH | 0.027124 | 6.201957 |
| SLC26A11 | 0.001352 | 2.070453 | PPP1R15A | 0.000317 | 2.840318 | HLA-DOB | 0.03734 | 6.322362 |
| LOC101928063 | 0.023733 | 2.072358 | PTPRH | 0.002853 | 2.842635 | TRIM21 | 0.000369 | 6.374683 |
| IL15 | 0.008304 | 2.072848 | N4BP1 | 0.006562 | 2.84352 | HOXB8 | 0.006472 | 6.426271 |
| CEP83-AS1 | 0.049609 | 2.076043 | HIST2H2BE | 0.01266 | 2.845818 | SEMA4A | 0.01187 | 6.479873 |
| RCAN1 | 0.001374 | 2.077398 | FAM186B | 0.029795 | 2.852197 | DUSP15 | 0.000193 | 6.493203 |
| C2orf82 | 0.010342 | 2.077604 | GPR68 | 0.019609 | 2.852345 | FAAH2 | 0.016077 | 6.534916 |
| SCN3B | 0.037781 | 2.079902 | KLLN | 0.003291 | 2.852499 | OAS3 | 0.000265 | 6.539402 |
| MTUS1 | 0.005998 | 2.082115 | P4HA3 | 0.00976 | 2.854271 | APOBEC3G | 0.000183 | 6.544238 |
| DNAJC12 | 0.003483 | 2.083545 | AGTRAP | 0.000269 | 2.856144 | VWA3A | 0.001488 | 6.57218 |
| NFKBIZ | 9.62E-05 | 2.083902 | LOC440896 | 0.045653 | 2.857463 | ATP12A | 0.004246 | 6.572914 |
| SNHG11 | 0.036218 | 2.083957 | NIPAL1 | 0.044566 | 2.86208 | BTN3A3 | 3.00E-07 | 6.59179 |
| NFKB2 | 5.48E-05 | 2.084276 | CXCL2 | 0.000217 | 2.864987 | PLEKHA4 | 3.31E-06 | 6.612948 |
| TBX2 | 0.000339 | 2.085477 | COLQ | 0.005153 | 2.867751 | PLSCR1 | 0.001042 | 6.625029 |
| ZNF296 | 0.012592 | 2.0872 | ISL2 | 0.000955 | 2.87371 | HES4 | 3.92E-06 | 6.636933 |
| SNHG12 | 0.001923 | 2.088766 | CTSS | 0.035573 | 2.876464 | NAT16 | 0.015179 | 6.638958 |
| PDE4B | 0.004433 | 2.088838 | SPSB1 | 0.00012 | 2.879183 | HLA-E | 7.48E-05 | 6.648252 |
| MMRN2 | 0.007255 | 2.089312 | C6orf15 | 0.000915 | 2.883788 | CAMKV | 0.00598 | 6.663287 |
| BAMBI | 0.000182 | 2.09015 | GRIK5 | 0.017826 | 2.886057 | RNU1-2 | 0.035575 | 6.677164 |
| C12orf79 | 0.016509 | 2.090379 | GRIN2C | 0.00046 | 2.889763 | RND1 | 0.000611 | 6.7257 |
| FAM89A | 0.018728 | 2.091166 | ST7-OT4 | 0.008703 | 2.890085 | HOTAIR | 0.00223 | 6.755581 |
| HPCA | 0.01776 | 2.091759 | DLX2 | 0.027895 | 2.893807 | PARP12 | 2.27E-06 | 6.783977 |
| ZNF747 | 0.002892 | 2.092566 | NLGN3 | 0.008703 | 2.896254 | HOXA-AS3 | 0.006232 | 6.805944 |
| ZNF835 | 0.020395 | 2.092958 | RHEBL1 | 0.00054 | 2.898109 | ABCA4 | 0.00248 | 6.819311 |
| HCN3 | 0.004972 | 2.095578 | MSX1 | 0.000544 | 2.900024 | LINC00163 | 0.017365 | 6.819965 |
| CROCCP2 | 0.000113 | 2.095931 | SYBU | 0.000212 | 2.903056 | BST2 | 9.39E-05 | 6.880584 |
| NUDT17 | 0.013223 | 2.095973 | EXD1 | 0.01433 | 2.905925 | PARP10 | 0.000993 | 6.882574 |
| C7orf57 | 0.019056 | 2.096203 | FA2H | 0.025949 | 2.910976 | OR1F1 | 0.008751 | 6.940385 |
| HIATL2 | 0.018051 | 2.098612 | SERPINF2 | 0.002176 | 2.91395 | APOL6 | 0.002754 | 6.95933 |
| CDK5R2 | 0.007859 | 2.098641 | SESN2 | 0.000336 | 2.918027 | TSPYL6 | 0.011077 | 7.026936 |
| MPP4 | 0.003762 | 2.101962 | NPIPB6 | 0.000334 | 2.920995 | KLHDC7B | 0.000867 | 7.032402 |
| SAMD3 | 0.002097 | 2.102164 | ARHGAP27 | 0.005797 | 2.925884 | PCDHA3 | 0.011792 | 7.043015 |
| AGAP11 | 0.003892 | 2.10302 | SLC6A12 | 0.047113 | 2.928114 | NCALD | 0.002465 | 7.067595 |
| CDRT1 | 0.009554 | 2.103662 | LAMA1 | 0.024208 | 2.928276 | FLJ13224 | 0.000212 | 7.088966 |
| FAS | 0.000405 | 2.104336 | MGAM | 0.025553 | 2.929513 | ABCG1 | 4.16E-05 | 7.093372 |
| OPTN | 0.000881 | 2.10441 | VIP | 0.000403 | 2.932034 | SULF1 | 1.95E-05 | 7.133164 |
| SLFN5 | 0.010329 | 2.104847 | STEAP1 | 0.003865 | 2.933748 | RPS6KA6 | 0.02106 | 7.163557 |
| MAGEA2 | 0.008915 | 2.105938 | ERAP2 | 0.000203 | 2.934011 | IL22RA1 | 0.023987 | 7.166959 |
| LOC143666 | 0.02379 | 2.10689 | DARS-AS1 | 0.005964 | 2.935636 | GCNT4 | 0.019165 | 7.168004 |
| SPATS2L | 0.000145 | 2.107149 | HIST3H2BB | 0.041544 | 2.93755 | AMY2A | 0.024555 | 7.201816 |
| PNRC1 | 0.000335 | 2.107272 | NAALAD2 | 0.024906 | 2.94131 | C5orf46 | 0.0135 | 7.238215 |
| PTGES3L | 0.005962 | 2.109459 | LSMEM1 | 0.002475 | 2.946024 | C1QTNF4 | 0.038244 | 7.265305 |
| C17orf67 | 0.039635 | 2.110516 | PFKFB4 | 0.000397 | 2.94825 | KLF4 | 0.004498 | 7.275905 |
| GAA | 0.000124 | 2.111231 | AVPI1 | 0.001047 | 2.954011 | LRRN3 | 0.013693 | 7.279523 |
| KAZALD1 | 0.009625 | 2.111957 | TF | 0.037546 | 2.963625 | AZGP1 | 0.001551 | 7.292651 |
| TMEM86A | 0.022349 | 2.113306 | FLJ31104 | 0.00584 | 2.965077 | FCGBP | 0.009133 | 7.293995 |
| GPNMB | 0.001915 | 2.114051 | TMEM255B | 0.049109 | 2.965506 | PNPLA1 | 0.000729 | 7.373489 |
| DIRAS2 | 0.034449 | 2.115531 | MEF2BNB-MEF2B | 0.003625 | 2.971465 | IL15RA | 0.017443 | 7.389571 |
| CBX4 | 0.001922 | 2.116613 | LOC101926941 | 0.033794 | 2.97203 | DHRS2 | 0.000363 | 7.398775 |
| RBM43 | 0.002212 | 2.11695 | ADAMTS13 | 0.049157 | 2.97684 | LOC105747689 | 0.042065 | 7.439191 |
| LIPA | 0.001123 | 2.117859 | IGF2 | 0.026835 | 2.97685 | ARHGAP9 | 0.011711 | 7.469587 |
| CPT1B | 0.001057 | 2.117935 | RFPL4A | 0.04157 | 2.978402 | CPLX3 | 0.020919 | 7.481007 |
| TMEM108 | 0.010186 | 2.118056 | LGI4 | 0.042396 | 2.978765 | NMI | 0.002112 | 7.564898 |
| PCDH1 | 0.001411 | 2.118274 | ANKK1 | 0.005751 | 2.97998 | HCLS1 | 0.042537 | 7.660876 |
| GATA6 | 0.002964 | 2.118966 | COL11A1 | 0.002497 | 2.98527 | TMEM110-MUSTN1 | 0.007877 | 7.762867 |
| GADD45B | 5.27E-05 | 2.119207 | TRIM38 | 0.000112 | 2.987912 | NFATC4 | 0.007099 | 7.779275 |
| CCDC30 | 0.008284 | 2.119506 | CCDC144NL-AS1 | 0.037103 | 2.991121 | SOCS1 | 0.000386 | 7.779605 |
| SERTAD1 | 0.000533 | 2.119874 | JUNB | 0.00125 | 3.022612 | RAET1L | 0.000341 | 7.809025 |
| LINC00921 | 0.035741 | 2.122709 | RBCK1 | 0.000939 | 3.022638 | ART3 | 0.006999 | 7.831563 |
| ZNF563 | 0.034191 | 2.124306 | LAPTM5 | 0.003527 | 3.025076 | IFI44 | 9.63E-05 | 7.839365 |
| HOXD3 | 0.001775 | 2.124694 | VSIG10L | 0.004263 | 3.030463 | MGAT4EP | 0.009057 | 7.870897 |
| CCDC96 | 0.009906 | 2.124926 | PSME2 | 1.25E-05 | 3.030943 | LOC101926975 | 0.00996 | 7.905769 |
| SIX1 | 0.00036 | 2.127011 | DNM1P35 | 0.049565 | 3.034008 | VCAM1 | 0.019345 | 7.933667 |
| ZFAS1 | 0.001463 | 2.12941 | DGCR9 | 0.000554 | 3.040564 | PLA2G4C | 0.000636 | 7.950755 |
| ZGLP1 | 0.006054 | 2.129549 | SCX | 0.008911 | 3.044919 | DDX60 | 0.002301 | 7.991386 |
| FBXL6 | 0.000108 | 2.130223 | PCSK1 | 0.012655 | 3.054246 | HCG26 | 0.000531 | 8.024358 |
| CMAHP | 0.009929 | 2.131754 | HOXB9 | 0.003017 | 3.054546 | PLG | 0.009629 | 8.030881 |
| SEPP1 | 0.000198 | 2.134346 | PREX2 | 0.020496 | 3.058241 | TTLL6 | 0.007251 | 8.035595 |
| SCML1 | 0.005188 | 2.13464 | PRDM8 | 0.014626 | 3.061165 | GBP1 | 0.000111 | 8.073742 |
| LOC100289511 | 0.025553 | 2.134895 | NLRP1 | 0.016767 | 3.067998 | GZMM | 0.00547 | 8.28096 |
| MOB3C | 0.000456 | 2.135673 | EXTL3-AS1 | 0.018055 | 3.071811 | C8orf31 | 0.011678 | 8.329302 |
| C4B_2 | 0.017916 | 2.139012 | HTR7 | 0.000149 | 3.075558 | TLDC2 | 0.000647 | 8.353328 |
| BRI3 | 0.001439 | 2.140194 | CRLF2 | 0.032948 | 3.090164 | SOX30 | 0.019287 | 8.401737 |
| LTBP2 | 0.008223 | 2.147441 | EFNA3 | 0.011292 | 3.094669 | CLEC7A | 0.041325 | 8.410824 |
| BACH2 | 0.031303 | 2.149589 | FAAH | 0.001007 | 3.101688 | ATP6V0A4 | 0.012402 | 8.458507 |
| SCOC-AS1 | 0.04186 | 2.149669 | CXCR6 | 0.044547 | 3.10319 | CD200R1 | 0.010955 | 8.462522 |
| MYCN | 0.016552 | 2.150257 | NYAP2 | 0.014324 | 3.105827 | PPFIA4 | 0.000797 | 8.480801 |
| LOC101060553 | 0.03534 | 2.150773 | GDA | 0.004062 | 3.106876 | GBGT1 | 0.01531 | 8.541256 |
| COL17A1 | 0.001512 | 2.151767 | GPR35 | 0.003236 | 3.1069 | SLC5A4 | 0.022361 | 8.623604 |
| AK4 | 4.36E-05 | 2.153046 | MOV10 | 3.18E-07 | 3.107255 | HERC5 | 0.001073 | 8.655871 |
| RNF122 | 0.021433 | 2.153096 | SCAMP1-AS1 | 0.000905 | 3.10916 | TLR3 | 5.18E-05 | 8.670353 |
| ZNF14 | 0.008308 | 2.153361 | SEC1P | 0.005402 | 3.117742 | TBX4 | 0.036477 | 8.682167 |
| ACP5 | 0.003198 | 2.153616 | LINC00106 | 0.049518 | 3.119264 | PSMB8-AS1 | 0.010182 | 8.730052 |
| GBP4 | 0.024874 | 2.154648 | ALDH8A1 | 0.002509 | 3.12365 | SERPING1 | 0.000837 | 8.747164 |
| KCNC3 | 0.005802 | 2.156768 | C9orf24 | 0.028872 | 3.134792 | GJD3 | 0.025625 | 8.75362 |
| SLC30A3 | 0.000538 | 2.156848 | TBR1 | 0.028081 | 3.136812 | LOC286059 | 0.002702 | 8.929324 |
| TMEM88 | 0.013723 | 2.160313 | TRIM14 | 0.003332 | 3.141754 | CACNA2D4 | 0.016432 | 8.981008 |
| CFAP53 | 0.009915 | 2.16046 | PRSS27 | 0.045311 | 3.144557 | HYDIN2 | 0.031028 | 9.006382 |
| MAEL | 0.03452 | 2.160464 | HORMAD2-AS1 | 0.018001 | 3.144908 | HLA-C | 2.57E-05 | 9.068818 |
| ZCCHC12 | 0.019585 | 2.160596 | BHLHE41 | 0.000205 | 3.147493 | HELZ2 | 0.003303 | 9.146292 |
| WHAMM | 1.98E-05 | 2.162834 | VEGFA | 4.39E-06 | 3.151332 | CASP1 | 0.004919 | 9.212255 |
| JUND | 0.00043 | 2.16302 | TNFSF18 | 0.013698 | 3.153036 | IFI6 | 0.001786 | 9.256098 |
| SLC16A3 | 0.000448 | 2.165698 | ANKRD24 | 0.01299 | 3.156438 | HECTD2-AS1 | 0.011212 | 9.283466 |
| DAPK2 | 0.0005 | 2.166355 | KIAA1683 | 0.002469 | 3.157494 | LOC101927740 | 0.043083 | 9.377454 |
| LRRC56 | 0.019152 | 2.167435 | HIST2H2AA3 | 0.000435 | 3.16378 | ACTN3 | 0.023345 | 9.402675 |
| DUSP5 | 0.009975 | 2.170771 | SMOX | 4.02E-06 | 3.166952 | FAM183B | 0.017087 | 9.461089 |
| CEBPB | 0.001861 | 2.171898 | FLNC | 0.018519 | 3.1682 | FBXO39 | 0.040782 | 9.483039 |
| SYT3 | 0.011476 | 2.173921 | PRRG4 | 0.00779 | 3.176994 | FOXJ1 | 0.045281 | 9.526196 |
| RELL2 | 0.000141 | 2.174323 | WASIR2 | 0.033684 | 3.178505 | TNNC2 | 0.016887 | 9.760659 |
| MGC12916 | 0.002808 | 2.17721 | SLC7A5P1 | 0.000369 | 3.185793 | HRK | 0.0122 | 9.817377 |
| SFN | 0.001218 | 2.178076 | LRRC4B | 0.001623 | 3.189104 | PIK3AP1 | 0.001925 | 9.902966 |
| ZMYM5 | 0.005958 | 2.178307 | NBPF13P | 0.030132 | 3.190652 | DDX60L | 0.002224 | 9.966666 |
| SP140L | 0.00481 | 2.179128 | ZNF844 | 0.00412 | 3.191027 | THEMIS2 | 0.016866 | 9.986111 |
| PPARGC1A | 4.01E-06 | 2.180364 | RGS2 | 0.000118 | 3.191311 | GLUD1P7 | 0.025628 | 10.09625 |
| TCEA3 | 0.003157 | 2.181886 | DTX3L | 0.001763 | 3.19704 | PNLDC1 | 0.00054 | 10.10491 |
| LAT2 | 0.000598 | 2.183611 | UNC79 | 0.007262 | 3.217801 | ANKDD1B | 0.00735 | 10.10663 |
| PLEKHA5 | 0.013336 | 2.185543 | HNRNPUL2-BSCL2 | 0.007869 | 3.217867 | PATL2 | 0.027044 | 10.14108 |
| CPLX1 | 0.019095 | 2.185861 | RNF165 | 0.025188 | 3.21801 | TNFAIP6 | 0.010367 | 10.20607 |
| KLF10 | 1.72E-05 | 2.186303 | GRIN2D | 0.001888 | 3.218177 | RPLP0P2 | 0.000229 | 10.24881 |
| CTC-338M12.4 | 0.036222 | 2.186415 | MAN1B1-AS1 | 0.005648 | 3.220216 | DDX58 | 0.000935 | 10.28027 |
| LRRC37B | 1.62E-05 | 2.18712 | RASGRP3 | 0.000893 | 3.22023 | TMEM100 | 0.031679 | 10.38132 |
| C8orf4 | 0.045162 | 2.18991 | LOC145845 | 0.016065 | 3.224177 | KCNN1 | 0.007134 | 10.40651 |
| RPSAP9 | 0.017309 | 2.190614 | FAM71E2 | 0.01258 | 3.228548 | NLRC5 | 0.002161 | 10.66411 |
| HAS2 | 0.000778 | 2.192466 | SELL | 0.004155 | 3.229306 | TRIM69 | 0.000396 | 10.74499 |
| OGDHL | 0.006068 | 2.192984 | RORA | 0.014241 | 3.235845 | NEURL3 | 0.001524 | 10.76185 |
| FGF11 | 0.000314 | 2.194697 | MLKL | 9.04E-06 | 3.236589 | C5orf56 | 0.003468 | 10.83807 |
| PPAP2B | 0.006938 | 2.19498 | PGM5 | 0.006467 | 3.253353 | LOC339166 | 0.001859 | 10.88554 |
| NR3C2 | 0.000789 | 2.196672 | ASPRV1 | 0.036455 | 3.253871 | CFAP57 | 0.00907 | 10.9592 |
| MST1P2 | 0.00125 | 2.197463 | TNFRSF8 | 0.040178 | 3.25603 | RSPH1 | 0.049741 | 10.96806 |
| GLTPD2 | 0.028128 | 2.197781 | UPK1A-AS1 | 0.013693 | 3.257801 | FST | 0.003819 | 10.98385 |
| AHRR | 0.0002 | 2.199236 | CCDC162P | 0.034606 | 3.258534 | TNFRSF14 | 0.000149 | 11.12094 |
| USP43 | 0.01581 | 2.200991 | CYP2B7P | 0.03232 | 3.258622 | LMO2 | 0.00147 | 11.29187 |
| CCT6B | 0.000764 | 2.202982 | BCL11B | 0.025326 | 3.259375 | TRANK1 | 0.000991 | 11.31314 |
| IER5 | 0.000691 | 2.204405 | SYNPO2 | 0.004833 | 3.262128 | USP18 | 0.00062 | 11.40211 |
| MST1R | 0.025005 | 2.204421 | C3AR1 | 0.009197 | 3.262483 | CFB | 0.006435 | 11.43805 |
| ABCD1 | 3.39E-05 | 2.205409 | CPEB3 | 0.027349 | 3.262553 | MX1 | 0.000179 | 11.47435 |
| ACER2 | 0.011306 | 2.206597 | RASEF | 0.046094 | 3.271316 | ATP10A | 0.001632 | 11.7839 |
| SERINC2 | 1.93E-05 | 2.206809 | DNAH17 | 0.001161 | 3.278162 | SPINK1 | 0.041386 | 11.87372 |
| PLAC1 | 0.025668 | 2.208014 | TH | 0.024154 | 3.278226 | HERC6 | 0.000533 | 11.92427 |
| RAB3A | 0.000757 | 2.208252 | PSMB10 | 0.000285 | 3.278932 | IFIH1 | 0.000528 | 12.06299 |
| BTG1 | 0.00025 | 2.208812 | PHYHIPL | 0.028692 | 3.281787 | HEPACAM2 | 0.001549 | 12.17202 |
| TECTA | 0.003026 | 2.211103 | TTC25 | 0.003977 | 3.282957 | ANGPTL6 | 3.67E-07 | 12.17959 |
| TNS1 | 0.01339 | 2.212213 | SHC4 | 8.30E-05 | 3.286307 | DCC | 0.028238 | 12.20573 |
| MICU3 | 0.000308 | 2.21507 | EBI3 | 0.009826 | 3.286919 | MMP13 | 0.00075 | 12.27666 |
| FSCN2 | 0.013961 | 2.216424 | NR4A2 | 5.56E-05 | 3.290226 | LIN28A | 0.001117 | 12.30156 |
| TRIM26 | 4.15E-07 | 2.218167 | ITGA11 | 0.000322 | 3.292829 | PLEKHG6 | 0.008128 | 12.38127 |
| ANKLE1 | 0.010551 | 2.218442 | DYSF | 0.033259 | 3.295891 | IFI35 | 0.000927 | 12.38272 |
| H1F0 | 0.000659 | 2.219073 | PML | 7.84E-06 | 3.297055 | SPATA18 | 0.006476 | 12.46964 |
| HDX | 0.001857 | 2.220021 | KLF15 | 0.019728 | 3.300217 | TTC9B | 0.00105 | 12.49787 |
| IGFBP2 | 2.46E-05 | 2.22078 | WDR49 | 0.02501 | 3.306946 | CCM2L | 0.043607 | 12.63184 |
| IL32 | 0.04257 | 2.224892 | DBP | 0.010842 | 3.307194 | HCP5 | 0.000454 | 12.67855 |
| EML2 | 0.03423 | 2.224977 | SIRT4 | 0.014399 | 3.319322 | CALB1 | 0.005619 | 12.754 |
| APOBEC3B | 0.006153 | 2.225504 | TRPC4 | 0.001852 | 3.32582 | HLA-B | 0.000155 | 12.95134 |
| FAM13A-AS1 | 0.006015 | 2.227372 | HCG27 | 0.026763 | 3.326438 | MFNG | 0.007316 | 13.07268 |
| TIMP3 | 0.001553 | 2.228308 | PARP8 | 0.016705 | 3.328459 | CSAG3 | 0.002907 | 13.13145 |
| PHF21B | 0.002924 | 2.229573 | MAP1LC3B2 | 0.022628 | 3.328585 | C9orf84 | 0.021081 | 13.19633 |
| KDM4D | 0.017944 | 2.230055 | GLI1 | 0.001085 | 3.3289 | LY6E | 0.000277 | 13.42824 |
| LIPG | 0.011984 | 2.231288 | PCSK1N | 0.018844 | 3.3292 | LOC100505817 | 0.032117 | 13.46524 |
| HAP1 | 0.000795 | 2.236702 | TMEM173 | 0.000466 | 3.336606 | IFI44L | 0.00039 | 13.51427 |
| PEX11G | 0.049307 | 2.24516 | ZNF385C | 0.033683 | 3.336973 | TRIM22 | 0.000274 | 13.61157 |
| ANKRD37 | 0.016793 | 2.245564 | FOXF1 | 0.000217 | 3.348136 | C19orf66 | 0.00084 | 13.65524 |
| SLC6A8 | 9.06E-06 | 2.245901 | PIK3IP1 | 0.000848 | 3.356262 | BCO1 | 0.002195 | 13.80155 |
| DNAAF3 | 0.024023 | 2.246917 | SLC6A10P | 0.038901 | 3.35835 | TAP1 | 2.06E-07 | 13.9161 |
| RTCA-AS1 | 0.048195 | 2.248412 | CFH | 0.000747 | 3.366886 | TMEM229B | 0.000872 | 13.99079 |
| ENDOG | 0.002564 | 2.248675 | BMP5 | 0.034711 | 3.369657 | CNTFR | 0.013928 | 14.01545 |
| ADAMTS6 | 0.000157 | 2.250274 | SSPO | 0.018128 | 3.370345 | DHX58 | 0.001242 | 14.06072 |
| RASIP1 | 0.007868 | 2.250404 | LOC101928053 | 0.0032 | 3.370769 | NOD2 | 3.36E-05 | 14.27239 |
| CASP7 | 0.000162 | 2.250873 | CCR10 | 0.021077 | 3.383305 | PACRG | 0.003111 | 14.35695 |
| PAOX | 0.005163 | 2.251956 | TRIM34 | 0.009028 | 3.383874 | KNDC1 | 0.01886 | 14.65346 |
| ZNF44 | 0.01321 | 2.252515 | CEBPD | 0.007588 | 3.385667 | ISG15 | 0.00095 | 14.94059 |
| HK2 | 0.000952 | 2.253653 | JHDM1D-AS1 | 0.000432 | 3.386611 | TNFRSF11A | 0.004145 | 14.99904 |
| ZFP36 | 0.000583 | 2.257532 | BOLA1 | 0.040666 | 3.388666 | APOL1 | 0.002308 | 15.09258 |
| ARRDC3 | 0.000394 | 2.258887 | CCND2 | 0.024283 | 3.388925 | ACTN2 | 0.005345 | 15.26319 |
| MYD88 | 0.004083 | 2.261463 | TMEM40 | 0.0016 | 3.390366 | DMKN | 0.009733 | 15.28788 |
| EPB41L3 | 0.034912 | 2.263626 | KLRG1 | 0.021498 | 3.392164 | HORMAD2 | 0.020745 | 15.34137 |
| NKX2-1 | 0.000215 | 2.265395 | HIST1H2AL | 0.035101 | 3.392295 | HAVCR1 | 0.048274 | 15.35988 |
| RASGRP1 | 0.013204 | 2.265665 | SDPR | 0.000151 | 3.393866 | CD69 | 0.020622 | 15.37731 |
| FAM162A | 0.009702 | 2.266142 | MAP4K1 | 0.000663 | 3.409115 | IGFN1 | 0.049251 | 15.53052 |
| CAPN3 | 0.038943 | 2.269648 | RYR2 | 0.003153 | 3.420251 | CCDC67 | 0.014621 | 15.56707 |
| SLC4A5 | 0.007632 | 2.271021 | WNK4 | 0.001203 | 3.42063 | PAX5 | 0.007903 | 15.87215 |
| PLAG1 | 0.015162 | 2.271254 | BTC | 0.005392 | 3.423256 | MYH16 | 0.001001 | 15.98164 |
| LINC01578 | 0.000318 | 2.274397 | FAM46A | 0.001353 | 3.425371 | HLA-F | 0.000385 | 16.03662 |
| LINC01126 | 0.031649 | 2.275303 | TDRD7 | 0.001772 | 3.425479 | LINC00917 | 0.041219 | 16.32019 |
| BIRC3 | 0.000116 | 2.275612 | WIPF3 | 0.034578 | 3.427804 | PIK3R6 | 0.004086 | 16.33917 |
| MDGA1 | 0.011544 | 2.276543 | C2 | 0.020028 | 3.431344 | OAS1 | 0.000277 | 16.37756 |
| MXRA8 | 0.037111 | 2.278794 | BISPR | 5.96E-05 | 3.431364 | C6orf58 | 0.025455 | 17.1592 |
| SREBF1 | 0.00251 | 2.279528 | SHISA2 | 0.000207 | 3.435345 | ABCA9 | 0.043902 | 17.23258 |
| NUDT18 | 0.007581 | 2.280525 | CARD14 | 0.049392 | 3.438198 | UBE2L6 | 0.001871 | 17.46374 |
| CHD5 | 0.006042 | 2.283639 | HIF1A-AS1 | 0.020903 | 3.43885 | MIR3648-1 | 0.000259 | 17.9992 |
| STX11 | 0.001271 | 2.28573 | KCTD16 | 0.005789 | 3.446713 | MAFB | 0.011403 | 17.99924 |
| APOBEC3F | 0.001958 | 2.286985 | SLC12A3 | 0.049167 | 3.450322 | SLC8A2 | 0.034564 | 18.2817 |
| EPB41L4A-AS1 | 0.01012 | 2.290535 | IRF9 | 0.00222 | 3.451948 | APOL3 | 0.000621 | 18.76781 |
| TIPARP | 0.000782 | 2.292341 | PTK6 | 0.001769 | 3.455747 | IFIT1 | 0.000136 | 19.39444 |
| IL11 | 0.005417 | 2.293951 | LOC100652768 | 0.014854 | 3.457858 | C3 | 0.000888 | 20.2655 |
| MICB | 1.64E-06 | 2.294045 | HIST1H3B | 0.047679 | 3.460363 | PTGER3 | 0.023487 | 20.68048 |
| LOC101929705 | 0.045263 | 2.294953 | LIPH | 0.000334 | 3.464556 | CACNA1I | 0.022199 | 20.91457 |
| CSRNP1 | 0.002559 | 2.295907 | MAK | 0.001167 | 3.465209 | GRIP2 | 0.002217 | 21.04756 |
| GUCA1B | 0.013838 | 2.296708 | LOC100134368 | 0.003758 | 3.467204 | EVX1 | 0.02015 | 21.4169 |
| VNN1 | 0.014801 | 2.298799 | LOC100268168 | 0.036467 | 3.47057 | CA9 | 2.17E-05 | 21.92082 |
| ITGA10 | 0.012181 | 2.299143 | MATN1-AS1 | 0.004236 | 3.485555 | UBA7 | 0.000109 | 21.99034 |
| RNF138P1 | 0.04955 | 2.29978 | NDUFA4L2 | 0.00314 | 3.507273 | ISG20 | 2.93E-08 | 22.93575 |
| CCT6P3 | 0.000801 | 2.301823 | COLEC11 | 0.02034 | 3.51203 | NPTX1 | 0.004306 | 23.44756 |
| CFAP70 | 0.042971 | 2.308149 | CXCL16 | 0.000101 | 3.516389 | LGALS9 | 0.002172 | 23.81007 |
| PRKCDBP | 0.008404 | 2.311217 | EHF | 0.002043 | 3.516436 | FAM209B | 0.022275 | 23.94211 |
| PMAIP1 | 0.003227 | 2.31175 | ADAMTS1 | 0.000139 | 3.518684 | OAS2 | 0.000362 | 24.72191 |
| PRKD2 | 7.50E-05 | 2.313994 | CYP2E1 | 0.000528 | 3.519944 | HSH2D | 0.000965 | 25.22202 |
| RAPGEFL1 | 0.005309 | 2.316438 | GBP5 | 0.003156 | 3.524439 | PSMB9 | 0.00307 | 26.76284 |
| SNTB1 | 0.002074 | 2.320928 | PRKCQ | 8.89E-05 | 3.540229 | RARRES3 | 0.002794 | 27.09138 |
| VWCE | 0.0181 | 2.322134 | CASP10 | 0.008984 | 3.540996 | ODF3B | 0.016926 | 27.33495 |
| ASMTL-AS1 | 0.021637 | 2.322167 | RSPH4A | 0.003832 | 3.546345 | FAP | 0.00532 | 27.70471 |
| ENPP2 | 0.003107 | 2.323268 | MMP10 | 0.003736 | 3.546701 | CCL5 | 0.008605 | 27.91572 |
| MAOA | 0.027428 | 2.32502 | RFPL3S | 0.031522 | 3.575066 | LOC653786 | 0.000159 | 28.38687 |
| TMEM158 | 0.001147 | 2.328495 | CASS4 | 0.013948 | 3.578991 | LOC401242 | 0.032756 | 28.46216 |
| SLC25A45 | 0.000469 | 2.329763 | LOC100419583 | 7.64E-05 | 3.582223 | BATF2 | 0.001355 | 28.91957 |
| STARD4 | 0.001986 | 2.329908 | PSORS1C2 | 0.025994 | 3.583506 | IFIT2 | 0.000326 | 30.86279 |
| ZNF222 | 0.000891 | 2.33317 | PARP9 | 8.50E-07 | 3.584164 | PLVAP | 8.99E-06 | 32.3125 |
| BRINP1 | 0.005308 | 2.333922 | DNALI1 | 0.042908 | 3.595407 | REM2 | 0.000174 | 34.32908 |
| LOC643355 | 0.029637 | 2.334717 | ZFP69 | 0.022964 | 3.59628 | SLC15A3 | 0.000263 | 35.00884 |
| EN2 | 0.0357 | 2.335456 | PTGER4 | 5.57E-05 | 3.598596 | IFIT3 | 0.000172 | 35.24611 |
| TNFSF13B | 0.00712 | 2.336683 | HBA2 | 0.020142 | 3.60407 | ETV7 | 0.006402 | 35.28727 |
| SOX3 | 0.000361 | 2.336731 | LOC101928100 | 0.025672 | 3.604585 | GBP1P1 | 0.001417 | 36.43601 |
| LOC101927237 | 0.023137 | 2.341054 | AOC3 | 0.006281 | 3.604661 | IFITM1 | 0.00222 | 36.56708 |
| CEL | 0.022113 | 2.343167 | MOV10L1 | 0.032471 | 3.609689 | CMPK2 | 0.000197 | 42.45737 |
| NFIL3 | 0.000165 | 2.345055 | GRAMD1B | 0.000673 | 3.61227 | RTP4 | 0.000624 | 42.95023 |
| ADAR | 0.000405 | 2.346463 | PPP1R3C | 6.12E-05 | 3.612391 | CEACAM1 | 0.011528 | 48.247 |
| ADGRF3 | 0.033704 | 2.346707 | FAM231D | 0.025435 | 3.615726 | CH25H | 0.005541 | 49.08436 |
| TMEM200C | 0.007929 | 2.349056 | TAF7L | 0.009352 | 3.621518 | MX2 | 0.000322 | 51.0236 |
| KCNG1 | 0.013795 | 2.350257 | ANGPTL4 | 0.02085 | 3.623964 | OASL | 0.000411 | 57.39722 |
| UNC5CL | 0.0051 | 2.350399 | STC2 | 1.40E-05 | 3.624122 | CHRNA1 | 0.000936 | 65.34348 |
| ZCCHC2 | 0.002431 | 2.354298 | LGALS3BP | 0.000151 | 3.624146 | CXCL11 | 0.008103 | 73.32661 |
| ABCA10 | 0.038916 | 2.354804 | LOC102724301 | 0.017129 | 3.624963 | TNFSF14 | 0.025866 | 75.20793 |
| ZDHHC11 | 0.013819 | 2.356036 | KCNK9 | 0.016643 | 3.642533 | IFNL1 | 0.001765 | 102.6174 |
| CXCL10 | 0.004707 | 174.2795 | APOE | 0.004332 | 3.651989 | XAF1 | 0.001467 | 116.2615 |
| RSAD2 | 0.000718 | 173.0969 |  |  |  |  |  |  |

**Supplementary Table 3. DEGs in HK-2 cells of 24h**

| gene | pvalue | FC | gene | pvalue | FC |
| --- | --- | --- | --- | --- | --- |
| IL33 | 0.031892103 | 0.018273271 | SLC25A1 | 0.004534095 | 0.457504137 |
| SPSB4 | 0.000227755 | 0.034473691 | SLC4A8 | 0.014319172 | 0.457744596 |
| LMOD1 | 0.026550851 | 0.039786808 | PTGER2 | 8.70E-05 | 0.457808911 |
| REN | 0.022511992 | 0.074475127 | MMP11 | 0.026973555 | 0.458320163 |
| KIAA1210 | 0.032692234 | 0.084787568 | LAYN | 0.011515555 | 0.458582791 |
| ENPP3 | 0.015298396 | 0.094832215 | PET100 | 0.021398377 | 0.459220703 |
| LOC100129518 | 0.047599053 | 0.097901947 | FAP | 0.007905299 | 0.459469429 |
| KCNK2 | 0.029560032 | 0.098060274 | PLEKHB1 | 0.039438701 | 0.459565804 |
| ZCCHC5 | 0.044889189 | 0.101010618 | SYTL4 | 4.07E-05 | 0.459727067 |
| GMNC | 0.025985836 | 0.104817799 | PAPSS2 | 0.003325175 | 0.460021573 |
| SERPINB7 | 0.001770152 | 0.111104342 | ANKRD34A | 0.003352706 | 0.460314345 |
| FAM13C | 0.007480301 | 0.111994566 | FAM117A | 0.021192296 | 0.460444973 |
| LINC01336 | 0.025811181 | 0.116950894 | LOC403323 | 0.005636537 | 0.460515437 |
| COL4A2-AS1 | 0.01500241 | 0.12270719 | DPYSL3 | 0.000842157 | 0.461239409 |
| MBOAT4 | 0.007755267 | 0.123374924 | FAM127C | 0.002514355 | 0.461699923 |
| LOC101927100 | 0.007507767 | 0.128279611 | DNASE1L1 | 0.009100861 | 0.461928908 |
| XIRP1 | 0.007292145 | 0.128543946 | SLC16A2 | 0.015188368 | 0.462758095 |
| COL6A5 | 0.008724826 | 0.128644199 | IL17RD | 0.003265585 | 0.463032176 |
| CPA4 | 0.037913674 | 0.129038652 | ACO1 | 0.009189028 | 0.463339373 |
| GALNT5 | 0.00214542 | 0.131644163 | POLE2 | 0.04281563 | 0.463404893 |
| MRVI1 | 0.027974917 | 0.137097273 | ARHGAP33 | 0.000802847 | 0.464589912 |
| FABP4 | 0.030937836 | 0.137416251 | NIPSNAP1 | 3.88E-05 | 0.464844955 |
| EPHA6 | 0.021640893 | 0.138014581 | LOC101927322 | 0.045938758 | 0.465309626 |
| LOC101928358 | 0.010179276 | 0.141946832 | GSTM1 | 0.000428129 | 0.465363327 |
| NRTN | 0.001997662 | 0.142540775 | NCKAP5 | 0.039269472 | 0.465587213 |
| MPP4 | 0.046639097 | 0.142621567 | INPP5D | 0.026940778 | 0.466555343 |
| TMEM35 | 0.013355183 | 0.142961194 | CCDC153 | 0.039888902 | 0.467137375 |
| IGSF10 | 0.037842581 | 0.143270194 | MIAT | 0.011806719 | 0.467214364 |
| HSPA2 | 0.0007251 | 0.144145734 | CCDC176 | 0.00037548 | 0.467882333 |
| SACS-AS1 | 0.046628105 | 0.144197209 | IL20RA | 0.00588229 | 0.468467552 |
| TMOD1 | 0.038862484 | 0.145165151 | SPATC1L | 5.43E-05 | 0.469190299 |
| TM4SF4 | 0.016078114 | 0.147482815 | GPATCH11 | 0.002164688 | 0.469516224 |
| CAPN14 | 0.033209233 | 0.148120157 | FBXW9 | 0.004101773 | 0.469822969 |
| EFR3B | 0.004335482 | 0.149204225 | TMEM106C | 0.025750173 | 0.470158658 |
| SYTL5 | 0.004390959 | 0.150894661 | ZNF703 | 0.004246151 | 0.470414911 |
| RASL11A | 0.000242064 | 0.151280448 | TP53INP2 | 0.001938582 | 0.471029955 |
| FAM133A | 0.001446951 | 0.153394572 | CHPF2 | 0.004274567 | 0.471133207 |
| UBD | 0.040708004 | 0.154801083 | BOK | 0.006942616 | 0.472732028 |
| AGTR1 | 0.030051489 | 0.159688696 | ST3GAL6 | 0.005394083 | 0.472847939 |
| ACAN | 0.007589163 | 0.160239831 | SALRNA1 | 0.013652237 | 0.473832371 |
| MGP | 0.006669735 | 0.160347006 | RABAC1 | 0.002252173 | 0.474256671 |
| MGARP | 0.000104659 | 0.160847104 | TRIM62 | 0.01489747 | 0.474776838 |
| PPFIA4 | 0.000252491 | 0.168345883 | LOC154761 | 0.009069708 | 0.474943161 |
| LOC101929122 | 0.042320124 | 0.171513061 | P3H3 | 0.011879412 | 0.474988437 |
| MEOX2 | 0.029943986 | 0.17202288 | GNRHR2 | 0.026468663 | 0.476159344 |
| C14orf37 | 0.006855791 | 0.172546847 | ERCC2 | 0.012810357 | 0.476561656 |
| SCUBE3 | 0.009684757 | 0.172862591 | GDF11 | 0.008096889 | 0.476791984 |
| LOC728392 | 0.007617922 | 0.173480024 | NDRG3 | 0.000625981 | 0.47681707 |
| SLC47A1 | 0.006183018 | 0.174165082 | ACTB | 0.014940514 | 0.476854164 |
| GREM2 | 0.003393411 | 0.174596501 | PPFIA3 | 0.027484082 | 0.476994825 |
| CSDC2 | 0.000113239 | 0.175276729 | CLDN6 | 0.006023391 | 0.477474299 |
| C5orf46 | 0.032858729 | 0.178707345 | PRADC1 | 0.014336799 | 0.47770345 |
| AP1M2 | 0.009042211 | 0.18062311 | NLGN4X | 0.007050256 | 0.477703846 |
| GFRA1 | 0.007966582 | 0.18139898 | LMO7 | 0.028217862 | 0.478052492 |
| FAM171A2 | 0.002166792 | 0.181631799 | STARD4-AS1 | 0.009079698 | 0.478183062 |
| C1orf110 | 0.005509061 | 0.184312039 | GALK1 | 0.000580251 | 0.478547681 |
| FABP3 | 0.000205514 | 0.184740931 | SH3BGRL2 | 0.005284474 | 0.478676865 |
| CDH8 | 8.02E-05 | 0.189362337 | HIBCH | 0.000234284 | 0.478919874 |
| SLITRK4 | 2.35E-05 | 0.192035509 | LINC01419 | 0.035392576 | 0.478970295 |
| GPR137C | 0.002442513 | 0.192683946 | CARNS1 | 0.019357068 | 0.479979177 |
| CSPG5 | 0.014852666 | 0.196147915 | TMEM86A | 0.007178121 | 0.480158694 |
| TRIM55 | 0.000669017 | 0.201236279 | NACAD | 0.002065132 | 0.480424334 |
| OLR1 | 0.033752694 | 0.201491571 | RAB30 | 0.004804262 | 0.480579607 |
| LRRC17 | 0.017854043 | 0.20250874 | HTR1B | 0.048857468 | 0.480599859 |
| LOC729950 | 0.004439766 | 0.202551219 | NSDHL | 0.025605249 | 0.480749519 |
| PLET1 | 0.049608127 | 0.203152073 | GAS6 | 0.001898039 | 0.480779245 |
| LOC90246 | 0.024073794 | 0.203393068 | CSRP1 | 0.027949084 | 0.480783708 |
| ST3GAL5 | 0.000468859 | 0.20393474 | NRP2 | 0.001763356 | 0.480807493 |
| STAR | 0.001574143 | 0.204363504 | FAT3 | 0.032974534 | 0.481331599 |
| DKK2 | 0.013525919 | 0.208555598 | FAM65C | 0.016738403 | 0.48135192 |
| CNN1 | 0.005324027 | 0.208863312 | CNN2 | 0.014063062 | 0.481447956 |
| HIST1H3E | 0.006927205 | 0.209036767 | LYRM4 | 0.005240263 | 0.481574918 |
| CHAD | 0.040979793 | 0.209855834 | BDH1 | 0.013619102 | 0.481875713 |
| PALMD | 0.002666311 | 0.211466021 | SNHG21 | 0.011948309 | 0.482208782 |
| RAB26 | 0.006848089 | 0.211582807 | CALU | 0.000738562 | 0.482556248 |
| NEXN | 0.000103495 | 0.212072195 | NLGN2 | 2.80E-05 | 0.483486952 |
| GALNT15 | 0.046774326 | 0.213210379 | PROCR | 0.031774735 | 0.483517227 |
| CDKN2C | 0.046072665 | 0.2133969 | SAMD11 | 0.039419481 | 0.48419011 |
| PCDH10 | 0.000411033 | 0.213529972 | CSRP2 | 0.003344124 | 0.484481877 |
| LINC01247 | 0.045851591 | 0.213891027 | KRT8 | 0.001648508 | 0.484772131 |
| MAST1 | 0.048465783 | 0.214970078 | PPAPDC3 | 0.007792403 | 0.485161953 |
| C2CD4C | 0.032645927 | 0.215403376 | MFNG | 0.016650955 | 0.485227043 |
| CHRM2 | 0.000595031 | 0.216024078 | TP53INP1 | 0.006146156 | 0.485699567 |
| SLITRK5 | 0.011975112 | 0.216465679 | CD302 | 0.00241144 | 0.485710361 |
| CEMIP | 0.000882316 | 0.216580148 | NPIPB15 | 0.017493236 | 0.485747655 |
| ACTA2 | 0.04042935 | 0.216901805 | DCHS2 | 0.045957622 | 0.486221648 |
| SFRP1 | 0.020706665 | 0.2186721 | CDR1 | 0.01239786 | 0.486398153 |
| ENPP1 | 4.74E-05 | 0.219532467 | HOXA-AS3 | 0.014631596 | 0.486685621 |
| MBNL1-AS1 | 0.001904134 | 0.220649424 | MAGEL2 | 0.007525182 | 0.487831374 |
| CXCL12 | 0.000295452 | 0.222607394 | DOK6 | 0.012798358 | 0.487908253 |
| KCND2 | 0.022415923 | 0.224931541 | TTC9 | 0.016218037 | 0.488052282 |
| TMEM221 | 0.016371485 | 0.225445064 | ZNF771 | 0.026353565 | 0.488128787 |
| EPHA5 | 0.034255338 | 0.226703445 | PCYOX1 | 0.003006298 | 0.488410146 |
| GP6 | 0.032881083 | 0.23034021 | CCDC74B | 0.000788497 | 0.488666224 |
| SEMA3G | 0.024146081 | 0.232743689 | GRIK2 | 0.013299692 | 0.48911575 |
| CACNG7 | 0.025012104 | 0.234079059 | DIRAS1 | 0.0009933 | 0.489258998 |
| ST6GAL2 | 0.031982412 | 0.234575743 | LINC01356 | 0.028669382 | 0.489356285 |
| GXYLT2 | 0.000254212 | 0.236444692 | FAM64A | 0.030564203 | 0.489449527 |
| FAXDC2 | 0.014344693 | 0.236572417 | STX1B | 0.018186547 | 0.489559165 |
| MEST | 0.000339402 | 0.238615745 | SPOCK3 | 0.035880003 | 0.490033815 |
| LINC00312 | 0.035124457 | 0.239794053 | GSN | 0.000816744 | 0.490036273 |
| NPPA | 0.017643321 | 0.241447339 | POP5 | 0.016949837 | 0.490255928 |
| AKAP5 | 9.48E-05 | 0.242155923 | SERTAD4 | 0.00095905 | 0.490567283 |
| FOXA1 | 0.000888311 | 0.242880391 | ZBTB18 | 0.003054795 | 0.490633373 |
| ALDOC | 0.007379403 | 0.245415875 | VCL | 0.002277378 | 0.490899609 |
| PKD1L2 | 0.024319442 | 0.246119769 | TRO | 0.000817379 | 0.491641265 |
| PC | 0.002351773 | 0.246477376 | GATS | 0.009929151 | 0.49182718 |
| SLC2A12 | 0.001680378 | 0.247922043 | LGI3 | 0.035021041 | 0.492235697 |
| IGFBP5 | 0.002030426 | 0.248157099 | SMPD1 | 0.000306819 | 0.492619815 |
| ASIC1 | 0.003690378 | 0.251886894 | OBSL1 | 0.00024497 | 0.49263872 |
| PSG5 | 0.011128464 | 0.252634297 | COL24A1 | 0.011826446 | 0.492737377 |
| CACNG4 | 0.002439035 | 0.252961496 | PLXDC2 | 0.010280872 | 0.493545571 |
| FILIP1L | 0.000788853 | 0.253055025 | ADAM22 | 0.009923785 | 0.493586269 |
| MVD | 0.030542868 | 0.253693102 | ALDH4A1 | 0.000486137 | 0.493647698 |
| CDH10 | 0.000318663 | 0.25485131 | FCER1G | 0.037588775 | 0.494821138 |
| NPR3 | 0.028978659 | 0.255040232 | NREP | 0.010396367 | 0.494997467 |
| LOC100506100 | 0.028801916 | 0.257027863 | MOK | 0.005945145 | 0.495307397 |
| ERMN | 0.038525663 | 0.258191132 | AQP3 | 0.023983435 | 0.495355061 |
| NTF3 | 0.012717613 | 0.258756496 | BTD | 0.002692101 | 0.496049797 |
| CXADRP3 | 0.029100018 | 0.258970946 | NDUFS5 | 0.006941683 | 0.496206959 |
| HTR1D | 0.001618608 | 0.259136479 | NEIL1 | 0.012692605 | 0.497079732 |
| LINC00562 | 0.045235505 | 0.259940883 | C1QTNF3 | 0.028125218 | 0.497121991 |
| OXTR | 0.012732373 | 0.260442472 | ZSCAN16-AS1 | 0.008912542 | 0.497139578 |
| SLA | 0.017424454 | 0.261900981 | SSC4D | 0.012964259 | 0.497256974 |
| B3GALT2 | 0.043488592 | 0.262943449 | GM2A | 0.003707753 | 0.497739496 |
| LINC00632 | 0.048423915 | 0.26308783 | FAM114A1 | 0.009622218 | 0.498252247 |
| LDLRAD2 | 0.011041178 | 0.265761531 | INPP5J | 0.025681034 | 0.49869873 |
| MAB21L2 | 0.016144383 | 0.268081849 | CSRNP3 | 0.027545604 | 0.499163581 |
| HMGCS1 | 0.034214866 | 0.268296406 | CNTN4 | 0.048558184 | 0.499328352 |
| RGS4 | 0.018419202 | 0.272114026 | KLHL29 | 0.000726191 | 2.000754884 |
| PRR15L | 0.024004755 | 0.272434814 | ZBTB7B | 0.002674608 | 2.002437458 |
| GAS1 | 0.033895848 | 0.275100648 | TAF1A-AS1 | 0.021425856 | 2.004818942 |
| DCLK2 | 0.00069261 | 0.277588585 | SH2B2 | 0.033751766 | 2.008274476 |
| PBX4 | 0.038913863 | 0.278588014 | SSX2IP | 0.004701786 | 2.00895645 |
| EXTL1 | 0.02878963 | 0.279730368 | HLA-DOB | 0.012515896 | 2.009255024 |
| TUBA1A | 0.020731854 | 0.279794919 | PAK1IP1 | 0.000321252 | 2.012761203 |
| TTC6 | 0.03222039 | 0.279886053 | LINC00266-1 | 0.024629767 | 2.013675391 |
| PIANP | 0.000155158 | 0.2815335 | HMOX1 | 0.041329327 | 2.015900856 |
| SLC6A6 | 0.0001653 | 0.281641198 | TCN2 | 0.001228686 | 2.016202422 |
| ACTG2 | 0.027579697 | 0.281869441 | PHACTR1 | 0.030381322 | 2.016818275 |
| LUM | 0.002959937 | 0.283267032 | HIST1H2BD | 0.040725348 | 2.02014669 |
| CNGA1 | 0.008912561 | 0.284535709 | NSMAF | 0.001845535 | 2.020291545 |
| SYT12 | 0.00111873 | 0.284610202 | CCT6P1 | 0.03021633 | 2.021232535 |
| VASH2 | 0.000856403 | 0.284797548 | MED10 | 0.004240941 | 2.021931654 |
| PTRF | 3.88E-05 | 0.286061266 | NTN4 | 0.019910757 | 2.022714 |
| GPR162 | 0.037944275 | 0.286609435 | C1GALT1C1L | 0.009502787 | 2.024134706 |
| BZRAP1-AS1 | 0.031606202 | 0.288228969 | CPEB4 | 0.000951879 | 2.024181246 |
| DMD | 0.00353253 | 0.288850657 | PTP4A1 | 3.90E-05 | 2.024468024 |
| DAPK3 | 0.000205743 | 0.289343387 | GJB2 | 0.008695933 | 2.024468037 |
| NRXN3 | 0.004218514 | 0.290802094 | SLC2A3 | 0.015442176 | 2.024807848 |
| COPG2IT1 | 0.01272252 | 0.290918756 | WBSCR27 | 0.035030666 | 2.02519501 |
| ALDH1B1 | 0.000267173 | 0.29237913 | CEBPB | 0.006948689 | 2.026000993 |
| FA2H | 0.036751876 | 0.293346969 | ATP8A2 | 0.00292616 | 2.027580557 |
| EFNB3 | 0.00117765 | 0.293638755 | ARL4A | 0.000606868 | 2.028046771 |
| DHCR7 | 0.004427965 | 0.293765367 | PTP4A3 | 0.001345793 | 2.029779977 |
| CBR3-AS1 | 0.009820163 | 0.294893719 | EXOSC8 | 0.001039848 | 2.032817572 |
| EGR3 | 0.025942167 | 0.295418837 | CCND2 | 0.035445065 | 2.033443097 |
| PTGES3L-AARSD1 | 0.027341028 | 0.295426674 | SQSTM1 | 0.002840861 | 2.036182903 |
| FDPS | 0.017318716 | 0.29555836 | TMEM62 | 0.006133036 | 2.037552369 |
| ARHGDIB | 0.005354565 | 0.295985212 | CEP126 | 0.01928657 | 2.039061779 |
| RIMS1 | 0.001770294 | 0.296100122 | FMNL2 | 0.008054589 | 2.039390014 |
| GPR18 | 0.041708497 | 0.296132945 | SLC25A37 | 0.000883526 | 2.042779096 |
| PRG4 | 0.000841614 | 0.297269705 | CCPG1 | 0.018690158 | 2.043249586 |
| MTUS1 | 0.03284333 | 0.298103738 | RNF19B | 0.002073789 | 2.044123904 |
| PLCE1 | 0.026057506 | 0.298455413 | ABCC2 | 0.038843539 | 2.045034895 |
| LRRC45 | 0.000908069 | 0.298956849 | SLC44A2 | 0.002133994 | 2.045904475 |
| NNAT | 0.013067944 | 0.298998851 | LOC100996437 | 0.007166073 | 2.046579467 |
| PIK3IP1 | 2.02E-05 | 0.300271682 | CNBP | 0.00219864 | 2.047204277 |
| RNASE4 | 0.002912318 | 0.301683205 | AIMP2 | 0.00905971 | 2.047262762 |
| ANXA8 | 0.038120752 | 0.30311572 | CASP8 | 0.008070732 | 2.049998707 |
| SNAI3 | 0.015915987 | 0.303735616 | SLC9A3R2 | 0.00742403 | 2.05008211 |
| LRP4-AS1 | 0.024571923 | 0.305223417 | LOC728613 | 0.022326415 | 2.050153367 |
| HOPX | 0.024932077 | 0.305578184 | MOV10 | 0.003658643 | 2.050311451 |
| DACT3 | 0.045057688 | 0.308522936 | ISM1 | 0.046711262 | 2.050336981 |
| LRFN1 | 0.024646528 | 0.308975983 | TM6SF2 | 0.035754523 | 2.052222167 |
| TMEM108 | 0.013942076 | 0.309704225 | L1CAM | 0.000237191 | 2.052328584 |
| EMILIN1 | 0.006745487 | 0.3115253 | MFSD2A | 0.0303741 | 2.052494047 |
| CCDC80 | 0.000956115 | 0.312772186 | KLF13 | 0.00188509 | 2.052577418 |
| SEMA4G | 0.000308516 | 0.312875114 | CMSS1 | 0.001876281 | 2.053490815 |
| LZTS1 | 6.34E-05 | 0.313251151 | TMCC1-AS1 | 0.006461233 | 2.056029617 |
| IGFBPL1 | 0.005618229 | 0.313779598 | CDC42EP4 | 0.000916813 | 2.057617055 |
| ACTBL2 | 0.001969493 | 0.314463649 | BCL2L1 | 0.019843994 | 2.057918942 |
| WISP1 | 0.028110552 | 0.314771187 | LINC00607 | 0.02796648 | 2.058167245 |
| B4GALNT4 | 0.016141785 | 0.315408544 | NUDCD1 | 0.00057991 | 2.059609875 |
| MAGED1 | 0.004720183 | 0.315445451 | CHKB-CPT1B | 0.02931311 | 2.061987803 |
| LOC100130987 | 0.004765507 | 0.31557599 | GSTO2 | 0.037732546 | 2.062815327 |
| PCDH7 | 0.001321923 | 0.315635557 | PECR | 0.032140322 | 2.0633335 |
| LPAR4 | 0.00227102 | 0.3179263 | NOP58 | 0.000203215 | 2.064179137 |
| THBS1 | 0.004383926 | 0.318085601 | BLACAT1 | 0.033922882 | 2.065363761 |
| IQGAP2 | 0.037747828 | 0.318746311 | FAM86B3P | 0.011220154 | 2.067288379 |
| RNF128 | 0.013666465 | 0.318999049 | SPATA6L | 0.048783605 | 2.067510434 |
| MPP2 | 0.011696183 | 0.319504981 | SUSD6 | 0.009823036 | 2.067856294 |
| IDH2 | 0.009236533 | 0.31954739 | LINC00623 | 0.008848212 | 2.068545054 |
| GATSL2 | 0.013026667 | 0.320207621 | CCDC113 | 0.020083504 | 2.069336292 |
| TM7SF2 | 0.002869059 | 0.320338701 | NUDT4 | 0.005650155 | 2.069371826 |
| FER1L4 | 0.00134665 | 0.32124706 | PCGF5 | 0.000636546 | 2.070256774 |
| LINC01139 | 0.009976147 | 0.321426461 | WDR78 | 0.001901473 | 2.073157458 |
| UTS2B | 0.010260544 | 0.322014177 | PHF11 | 0.001444183 | 2.074867575 |
| LDHD | 0.032733471 | 0.322244104 | ADRA1B | 0.023234836 | 2.075386899 |
| PRSS23 | 0.001244552 | 0.323923328 | EXOSC5 | 0.000319066 | 2.075647888 |
| LMCD1 | 0.005705219 | 0.324314445 | FIBIN | 0.030149369 | 2.079394307 |
| MAGED4B | 0.047991451 | 0.32550027 | SLMO1 | 0.015910529 | 2.080590861 |
| ACSS2 | 0.015576003 | 0.326279812 | TNFRSF10A | 0.004137056 | 2.081100649 |
| PNPLA3 | 0.011025113 | 0.326441746 | SNHG8 | 0.004234275 | 2.081692847 |
| MAGEE1 | 0.001618354 | 0.327121355 | CCDC67 | 0.030162826 | 2.082750607 |
| SMTN | 0.027334554 | 0.327525209 | TRIM47 | 0.012811878 | 2.084853561 |
| SCD | 0.007105535 | 0.328532564 | ZNF311 | 0.011732686 | 2.085546464 |
| SCD5 | 0.005692268 | 0.328729007 | PSMA4 | 0.007999534 | 2.087505559 |
| REEP2 | 0.004456169 | 0.329348896 | UBIAD1 | 7.59E-05 | 2.088046145 |
| ASB16 | 0.041353079 | 0.329552994 | RHBDF2 | 0.001027429 | 2.090021815 |
| MMP24 | 1.80E-05 | 0.329727116 | EVA1C | 0.006984796 | 2.090242274 |
| SEPT5-GP1BB | 0.022623022 | 0.329855787 | RELN | 0.000652086 | 2.090362806 |
| CYP51A1-AS1 | 0.00884179 | 0.330989818 | KCNMA1 | 0.007080611 | 2.095449537 |
| RFTN2 | 0.044719173 | 0.33247705 | TFAP4 | 0.005352323 | 2.096872834 |
| ADAMTS5 | 0.023781331 | 0.332868267 | NMNAT2 | 0.021625531 | 2.097637739 |
| DAAM2 | 0.032953054 | 0.333028899 | SCNN1A | 0.002767489 | 2.099541518 |
| GNG7 | 0.003418298 | 0.333277759 | PXK | 0.003789702 | 2.099729103 |
| KIF20A | 0.033729273 | 0.334018173 | SNAI2 | 0.000934988 | 2.100152568 |
| LOC101929532 | 0.012936904 | 0.334463944 | LOC728323 | 0.032679281 | 2.101849634 |
| VAT1L | 0.016229207 | 0.334658308 | KCTD16 | 0.02064065 | 2.108915561 |
| FASN | 0.04230176 | 0.334941572 | RCC2 | 0.000233453 | 2.109859289 |
| LXN | 1.19E-05 | 0.335133155 | CLDN4 | 0.004052576 | 2.111559717 |
| GDF5 | 0.013000481 | 0.335323615 | SCO2 | 0.001827132 | 2.111617338 |
| TMCC2 | 1.00E-05 | 0.335430925 | AVIL | 0.006513727 | 2.112961168 |
| SLC1A1 | 0.044845832 | 0.335505119 | PRDM6 | 0.006314105 | 2.114069629 |
| ANGPTL7 | 0.049744368 | 0.336217166 | CEBPG | 0.001130802 | 2.114261458 |
| RASL10B | 0.017789184 | 0.336329249 | SNHG17 | 0.015407421 | 2.114710217 |
| PADI1 | 0.006786699 | 0.337078751 | MSR1 | 0.024428919 | 2.117124874 |
| LOC105274304 | 0.005805419 | 0.337481004 | GAL3ST4 | 0.00324653 | 2.117488759 |
| SEMA5A | 0.010824094 | 0.337962996 | FAM135A | 0.009960913 | 2.118624261 |
| ANXA8L1 | 0.021293231 | 0.338274345 | TNFRSF10B | 0.001227311 | 2.120736845 |
| TLL1 | 0.006288783 | 0.338459803 | FICD | 0.007325193 | 2.1220351 |
| MYL6 | 9.98E-05 | 0.340012701 | FAM169A | 0.00460319 | 2.12466842 |
| PDGFRL | 0.003113954 | 0.342304322 | SPP1 | 0.047385968 | 2.124777145 |
| LOC101927359 | 0.02236153 | 0.342366315 | ITPRIP | 0.005512169 | 2.125325719 |
| B3GNT8 | 0.008092202 | 0.343035236 | CYGB | 0.004026937 | 2.126004644 |
| ACOX2 | 0.003328824 | 0.343468796 | C4orf32 | 0.006089762 | 2.126432834 |
| SATL1 | 0.038785226 | 0.345014917 | EPHA3 | 0.005156725 | 2.126660796 |
| EPHA5-AS1 | 0.02898306 | 0.346654763 | KIAA0040 | 0.000501951 | 2.128860067 |
| PCAT6 | 0.009789566 | 0.346664437 | ABCC4 | 0.021026051 | 2.130752455 |
| SULF1 | 0.011180591 | 0.346845488 | LYRM1 | 0.005796351 | 2.130887632 |
| PCDH20 | 0.019851878 | 0.347906535 | HLA-E | 0.000164791 | 2.133559433 |
| ID3 | 0.002229106 | 0.348160134 | PRKCQ | 0.003321335 | 2.133711683 |
| KDELR3 | 0.020511839 | 0.34883026 | IFI16 | 0.000929651 | 2.134758683 |
| 5-Sep | 0.016136663 | 0.349151445 | ARRDC2 | 0.01366534 | 2.135033033 |
| NRGN | 0.00158948 | 0.349316688 | LOC374443 | 0.003350547 | 2.13910927 |
| GSTM2 | 0.000179102 | 0.349758 | JAK2 | 0.004683764 | 2.14282186 |
| PCDHB12 | 0.003140656 | 0.349804728 | ASPHD2 | 0.00449165 | 2.142847238 |
| CYP4F35P | 8.92E-05 | 0.350060452 | SLCO3A1 | 0.024124458 | 2.145313746 |
| RPL13AP20 | 0.004823759 | 0.350432916 | RRN3P1 | 0.010086108 | 2.146069631 |
| SLC22A17 | 0.005660562 | 0.351986961 | ZNF528-AS1 | 0.008555721 | 2.146299188 |
| CHST9 | 0.045065822 | 0.353242968 | KIF21B | 0.010975916 | 2.146322396 |
| GPR155 | 4.48E-05 | 0.354396428 | ULBP1 | 0.015115507 | 2.147082422 |
| ACTR3C | 0.015885999 | 0.355205774 | NCOA7 | 0.001492484 | 2.149185753 |
| CCNT2-AS1 | 0.009687456 | 0.355328865 | CLDN3 | 0.040754715 | 2.150315838 |
| LDB3 | 0.002828019 | 0.356137626 | TMCC3 | 0.005455194 | 2.152092787 |
| YPEL3 | 0.009354349 | 0.356158375 | RNF32 | 0.026208713 | 2.152672292 |
| SEMA6C | 0.018798728 | 0.356241831 | OPTN | 0.00021629 | 2.153935317 |
| PRUNE2 | 0.000266443 | 0.356337912 | IFITM3 | 0.000190822 | 2.156001785 |
| LOC100130417 | 0.006339795 | 0.35762095 | LINC00909 | 0.000827904 | 2.156566017 |
| AADACL4 | 0.00044433 | 0.357851393 | LINC01270 | 0.040675872 | 2.157628539 |
| DIRAS2 | 0.016299237 | 0.358031699 | SP2-AS1 | 0.004811785 | 2.157809831 |
| PDE5A | 1.33E-05 | 0.358075899 | EMP1 | 0.000496045 | 2.159919761 |
| TMEM170B | 0.006296348 | 0.358359226 | RALA | 0.003699957 | 2.161257367 |
| INHBA | 0.000387678 | 0.358660398 | ADGRE5 | 0.003928127 | 2.164618752 |
| TMEM63C | 0.023804952 | 0.358840185 | STPG1 | 1.19E-05 | 2.165698683 |
| ADAMTS2 | 1.53E-05 | 0.358848221 | C19orf66 | 0.002570264 | 2.170164002 |
| ABAT | 0.0008053 | 0.359000757 | TDRD7 | 0.000454979 | 2.171353543 |
| MYH10 | 0.00155085 | 0.36018719 | MYH7B | 0.014859089 | 2.17164936 |
| TMEM150C | 7.08E-05 | 0.360295106 | KRBOX1 | 0.035343842 | 2.173811454 |
| BMPER | 0.0423518 | 0.360765678 | HLA-H | 0.023759842 | 2.176321102 |
| EMP2 | 0.009291532 | 0.360864888 | THBD | 0.015632256 | 2.176414463 |
| TXNDC16 | 0.007185961 | 0.362329961 | GTPBP10 | 0.00010303 | 2.177276855 |
| QPRT | 0.000608864 | 0.3629856 | TGIF1 | 4.99E-05 | 2.178135547 |
| FAM46B | 0.009941564 | 0.365347388 | RINL | 0.026690422 | 2.180015145 |
| COL4A4 | 0.003168716 | 0.365475861 | SLC7A11 | 0.016076431 | 2.181626848 |
| LOC91450 | 0.021033969 | 0.365798915 | PARP8 | 4.75E-05 | 2.182046486 |
| SESN3 | 0.000363413 | 0.367721353 | TAF1A | 0.010253409 | 2.183029382 |
| CTGF | 0.016128255 | 0.367764431 | TRIM21 | 6.46E-05 | 2.183108756 |
| HTR1F | 0.014671503 | 0.368458367 | DNPEP | 0.000108928 | 2.185582631 |
| SLC17A5 | 0.014565886 | 0.370421761 | BEX2 | 0.006443871 | 2.18597259 |
| SNRNP25 | 0.017524413 | 0.371567894 | ADAP1 | 0.015224546 | 2.187938511 |
| PRKCDBP | 0.00096476 | 0.371732612 | CCDC154 | 0.042681052 | 2.190665905 |
| EPPK1 | 0.010793025 | 0.371788948 | PPAP2C | 0.040783258 | 2.190963682 |
| PDPN | 1.14E-05 | 0.371871242 | JADE2 | 0.00579243 | 2.191181495 |
| LINC00685 | 0.012846603 | 0.372129777 | DYNLL2 | 0.014214837 | 2.191879818 |
| LOC401052 | 0.014468142 | 0.372619331 | APOL1 | 0.004859774 | 2.195175302 |
| GCAT | 0.000247618 | 0.373313266 | THNSL1 | 0.002665456 | 2.196333653 |
| OSR2 | 0.005352903 | 0.373614054 | ACVRL1 | 0.006866049 | 2.197148759 |
| PRRT2 | 0.009432333 | 0.37512938 | NUDT4P1 | 0.000905012 | 2.20057212 |
| NT5DC2 | 0.01509277 | 0.376018282 | COL5A3 | 0.034715587 | 2.203680483 |
| CACNG8 | 0.007383683 | 0.376428921 | NCR3LG1 | 0.011240216 | 2.205373792 |
| FAM198B | 0.020361832 | 0.378713919 | ARHGAP27 | 0.010955696 | 2.206737792 |
| LRRC32 | 0.014481997 | 0.37880464 | ABTB2 | 0.004327738 | 2.208027671 |
| TNFAIP8L1 | 0.015246205 | 0.378998728 | CDC42EP3 | 0.002161608 | 2.209663265 |
| SLC29A2 | 0.02263104 | 0.379371783 | BTG1 | 0.01006974 | 2.210986878 |
| ADAMTS12 | 0.001553007 | 0.379640247 | UNC13D | 0.004500849 | 2.213606212 |
| STON1 | 0.018086089 | 0.379960143 | ADAR | 0.000581192 | 2.214777541 |
| NPC2 | 3.93E-05 | 0.380786082 | ZNF501 | 0.001673488 | 2.217461363 |
| SLC24A5 | 0.022242562 | 0.381213908 | NT5E | 0.000797801 | 2.219023355 |
| HMCN1 | 9.74E-05 | 0.381765748 | HOXD3 | 0.005148887 | 2.219160172 |
| RAB3A | 0.047809058 | 0.382666378 | SLC3A2 | 0.001194573 | 2.219719737 |
| ADAMTS16 | 0.006329296 | 0.382689886 | C9orf91 | 0.000892403 | 2.220528672 |
| PTGIS | 0.004828204 | 0.383043203 | HLA-A | 6.21E-05 | 2.220543609 |
| ZBED2 | 0.02051601 | 0.384125273 | ZMYND12 | 0.04057736 | 2.222940047 |
| PURG | 0.025124124 | 0.385394697 | TNFAIP3 | 0.002904444 | 2.223422922 |
| NYNRIN | 7.81E-06 | 0.385526173 | DDIT4 | 0.001449492 | 2.228078109 |
| GYG2 | 3.99E-05 | 0.386590766 | FBXL13 | 0.027693135 | 2.232298796 |
| TSPAN2 | 0.017890946 | 0.386742183 | UNC93B1 | 0.000401673 | 2.233669221 |
| NDRG4 | 0.015804352 | 0.386809764 | NUP50-AS1 | 0.005261178 | 2.236736822 |
| IGF2 | 9.03E-05 | 0.387383302 | FAM231A | 0.010671144 | 2.238796583 |
| C10orf10 | 0.003770551 | 0.388175878 | XPO5 | 1.26E-05 | 2.239266742 |
| PCDHGA3 | 0.006678339 | 0.388560376 | RAB3D | 0.02086794 | 2.239961342 |
| CCDC74A | 4.77E-05 | 0.388590095 | LINC00944 | 0.046073222 | 2.24235451 |
| AIF1L | 0.004727127 | 0.389630417 | GBP3 | 0.000168821 | 2.243527988 |
| TMEM191A | 0.022319967 | 0.390147412 | DOK5 | 0.002806719 | 2.244131351 |
| NOX4 | 0.018083263 | 0.390350294 | LOC100506178 | 0.016249008 | 2.245430418 |
| KCNMB4 | 0.037691647 | 0.390667549 | TAP2 | 0.000159128 | 2.245569752 |
| CALD1 | 0.002825987 | 0.390690433 | ZNF844 | 0.010414769 | 2.24707141 |
| MYL9 | 0.008328337 | 0.390850132 | NEURL1 | 0.004377881 | 2.249369621 |
| PCSK9 | 0.005306053 | 0.390897873 | GPR143 | 0.001365745 | 2.2511608 |
| LINC01279 | 4.80E-05 | 0.391242645 | ETS1 | 0.007994955 | 2.252152507 |
| TNFRSF19 | 0.000362879 | 0.391649258 | TNFRSF6B | 0.017522589 | 2.25219214 |
| RCN3 | 0.004502232 | 0.391928115 | KLF10 | 8.38E-05 | 2.255473038 |
| FAM127A | 0.000539575 | 0.392103146 | CHIC2 | 0.036035903 | 2.256991852 |
| ENTPD7 | 0.001604641 | 0.392500466 | GTPBP2 | 0.000286505 | 2.257162279 |
| NEFM | 0.008287176 | 0.39298359 | GUCA1B | 0.036830907 | 2.258943319 |
| TUBB2B | 0.001864815 | 0.39310048 | FTH1 | 0.01725949 | 2.261912037 |
| DHCR24 | 0.026670163 | 0.395384977 | ZNF274 | 0.000482695 | 2.26408221 |
| DNAJB5 | 0.001501416 | 0.396197732 | RENBP | 0.011193043 | 2.26459476 |
| MAGED4 | 0.001548297 | 0.396221481 | LBX2-AS1 | 0.009480946 | 2.265477873 |
| PDGFD | 0.004449841 | 0.397378322 | PLEKHG4 | 0.000711469 | 2.265924442 |
| TMEM160 | 0.008562706 | 0.397721276 | PRRT3-AS1 | 0.004152068 | 2.269862522 |
| STK38L | 0.000940395 | 0.398650323 | FLT3LG | 0.00356957 | 2.271022366 |
| TMEM130 | 0.019878798 | 0.398689775 | SLC6A9 | 0.026018756 | 2.271894638 |
| ZNF491 | 0.008503754 | 0.399285006 | IL7 | 0.038099272 | 2.275893151 |
| ANKRD44 | 0.003574317 | 0.400266608 | FAM86FP | 0.015177234 | 2.27621239 |
| DBI | 0.004578095 | 0.400496151 | SLC38A1 | 0.002920578 | 2.279966699 |
| MAGI1 | 0.006441959 | 0.401318537 | GDAP1 | 0.000613994 | 2.281673327 |
| ANKS1B | 0.014453323 | 0.402538123 | STC2 | 0.005369311 | 2.283463712 |
| ABCA8 | 0.007337554 | 0.403256596 | ZC3H12C | 0.001595072 | 2.285297895 |
| LSS | 0.007491057 | 0.404630813 | SDSL | 0.000608418 | 2.28804781 |
| CAND2 | 0.000114526 | 0.404974301 | DDO | 0.019728847 | 2.2891677 |
| GAMT | 0.001420263 | 0.404995446 | SLC38A2 | 0.001272059 | 2.290922003 |
| SLC44A3 | 0.026092939 | 0.40521816 | ZNF610 | 0.004757521 | 2.293153493 |
| HHIP | 0.043844097 | 0.406354384 | PSMB10 | 0.008720336 | 2.297417889 |
| AP1S3 | 0.004142324 | 0.406434365 | AOC3 | 0.010118433 | 2.299009684 |
| DANCR | 0.001271157 | 0.406454325 | KLF5 | 0.000380834 | 2.299568575 |
| FOXO4 | 0.006224508 | 0.406537692 | CARD9 | 0.03696013 | 2.300140655 |
| GPRIN3 | 0.005311193 | 0.407511726 | JAM2 | 0.018137712 | 2.301563655 |
| SNHG18 | 0.003306912 | 0.407704065 | PNCK | 0.048769435 | 2.302130254 |
| PIFO | 0.011527144 | 0.408260158 | RASGEF1B | 0.021254191 | 2.303060815 |
| P2RY6 | 0.008251085 | 0.408427317 | EGFR | 0.000339811 | 2.304697197 |
| SLC25A23 | 7.26E-05 | 0.408792317 | PMAIP1 | 0.003667743 | 2.307103527 |
| PRR36 | 0.001941503 | 0.409543819 | ABCC3 | 0.000239951 | 2.307525526 |
| GSTM3 | 0.004912623 | 0.409812269 | ZHX2 | 6.45E-06 | 2.309996229 |
| ANG | 0.018601003 | 0.410268025 | ZBTB16 | 0.04066382 | 2.310515625 |
| ACTN4 | 0.005194728 | 0.410387612 | DUOX1 | 0.017329108 | 2.314345456 |
| FAM69B | 0.000899999 | 0.410439699 | C10orf2 | 0.00672322 | 2.314465106 |
| FAM78A | 0.001884107 | 0.410530316 | RFPL1 | 0.030151543 | 2.314635688 |
| ANXA6 | 0.003412231 | 0.410816886 | ZC3H12A | 0.027192214 | 2.316811876 |
| B4GAT1 | 0.000280556 | 0.41113956 | SH2D4A | 5.35E-05 | 2.31750776 |
| PTOV1 | 0.012552298 | 0.411281069 | APOD | 0.023023354 | 2.31760315 |
| C1orf198 | 0.005155605 | 0.411584112 | FOSL1 | 0.002120579 | 2.317841174 |
| IKZF2 | 0.033259251 | 0.411796039 | KLF2 | 0.04904758 | 2.322951773 |
| MXD4 | 0.000377397 | 0.412161312 | CX3CL1 | 0.003520662 | 2.324179395 |
| ACTG1 | 0.007666773 | 0.412295367 | CNR1 | 0.01878321 | 2.328734365 |
| NDRG2 | 0.008081568 | 0.412358542 | PLCB1 | 0.017160785 | 2.329964175 |
| TMEM97 | 0.024019102 | 0.413108932 | LOC101927027 | 0.002254949 | 2.332329231 |
| HSD17B14 | 0.007002486 | 0.413193068 | FAM86EP | 0.001475647 | 2.332659421 |
| FADS2 | 0.0156079 | 0.413261267 | FAM71F2 | 0.013442631 | 2.334699044 |
| XYLT1 | 0.020441022 | 0.414082011 | LAMC2 | 0.007492004 | 2.337265338 |
| SPEG | 0.000748365 | 0.414637285 | PYROXD1 | 0.000104628 | 2.337928749 |
| RUNDC3A-AS1 | 0.005632616 | 0.414878979 | ADM2 | 0.006058495 | 2.339199983 |
| LOC101927056 | 0.044844843 | 0.415004281 | SLC25A28 | 0.001352334 | 2.340926816 |
| TRPC4 | 0.011281853 | 0.41514163 | TM6SF1 | 0.000340427 | 2.342119018 |
| CORO6 | 0.005580163 | 0.415332477 | TMEM232 | 0.006628957 | 2.342957124 |
| TUBB6 | 0.003307544 | 0.416945513 | GFPT1 | 0.003911273 | 2.347620778 |
| ENC1 | 0.001044987 | 0.416998795 | AHR | 0.000395285 | 2.349971131 |
| TUBB3 | 0.040406194 | 0.417047802 | PFDN2 | 0.002997999 | 2.350129745 |
| IDI1 | 0.044367337 | 0.417127995 | ATF5 | 0.000333253 | 2.350343602 |
| SALL2 | 0.00388292 | 0.417286954 | ELF3 | 0.033012736 | 2.350345324 |
| LINC00957 | 0.002093703 | 0.417560533 | SP100 | 7.96E-05 | 2.35212367 |
| BMF | 0.001528787 | 0.417634705 | CLDN16 | 0.046566206 | 2.353341045 |
| SYNGR1 | 0.004638682 | 0.418388534 | SFRP5 | 0.008018105 | 2.353421455 |
| CPT1C | 0.001933926 | 0.418940767 | ZNF296 | 0.009268123 | 2.358080287 |
| MVK | 0.008072235 | 0.41913297 | ZFP69B | 0.001655779 | 2.367178431 |
| MYH9 | 0.007329537 | 0.419456318 | PLD6 | 0.003063208 | 2.36867539 |
| LOC100134868 | 0.015418905 | 0.419904508 | ACRBP | 0.048547918 | 2.371838972 |
| TEK | 0.022152364 | 0.419945692 | CCRL2 | 0.006190978 | 2.379799058 |
| TPPP3 | 0.003623238 | 0.420435775 | SESN2 | 0.002819868 | 2.380947603 |
| ADAM12 | 0.002365167 | 0.420718241 | HTR2B | 0.025698866 | 2.382493813 |
| ARSJ | 0.000518616 | 0.420786853 | SPAG17 | 0.01032508 | 2.382678548 |
| FSCN1 | 0.008139722 | 0.421661699 | DOCK4 | 0.00169263 | 2.38657036 |
| HOXA11-AS | 0.003292132 | 0.422245153 | RET | 0.047215091 | 2.390330749 |
| ARSE | 0.013643831 | 0.422831489 | BISPR | 0.019355755 | 2.393743303 |
| RAB40B | 0.024216446 | 0.422935172 | CPEB3 | 0.030255201 | 2.394039926 |
| GDPD1 | 0.00827195 | 0.423365282 | CARD16 | 0.009981939 | 2.398959216 |
| SLC46A3 | 0.002771 | 0.424172043 | CNIH3 | 0.000580227 | 2.400950401 |
| HDAC5 | 0.003890505 | 0.425229149 | ARSG | 0.001077042 | 2.404561357 |
| TUBB2A | 0.005123159 | 0.425307284 | C6orf48 | 0.003474603 | 2.404582937 |
| TBC1D8B | 0.000589756 | 0.425601846 | EFCAB2 | 0.001752941 | 2.405964301 |
| TDRKH | 0.001509208 | 0.425683161 | ARFGEF3 | 0.043112582 | 2.407085439 |
| ARHGEF25 | 0.003802841 | 0.425764684 | MKNK2 | 0.017833224 | 2.407332163 |
| SQLE | 0.01725488 | 0.426015946 | PRSS54 | 0.008973605 | 2.407861536 |
| FAM101B | 0.014546134 | 0.426408014 | HOXD8 | 0.000270866 | 2.408180751 |
| TSPAN15 | 0.000296183 | 0.427387135 | NUP62CL | 0.038025285 | 2.408678222 |
| EBP | 0.047976913 | 0.427862183 | NSUN7 | 0.039900007 | 2.419787427 |
| PCDHGA7 | 0.011988146 | 0.427915683 | ZDHHC14 | 0.032201514 | 2.42208474 |
| APCDD1L | 0.002721418 | 0.428202283 | VPS9D1-AS1 | 0.004416516 | 2.422339682 |
| AK5 | 0.001538933 | 0.429190848 | C11orf70 | 0.010698785 | 2.422778653 |
| CLEC11A | 0.000725227 | 0.429647048 | ATP6V1E2 | 0.017385654 | 2.423085116 |
| SMIM10L2B | 0.00833251 | 0.431562383 | PIK3AP1 | 0.004429907 | 2.423160836 |
| RTL1 | 0.04340798 | 0.431711831 | TMEM116 | 0.001219879 | 2.424400925 |
| HMG20B | 0.003178778 | 0.432135745 | MYCBPAP | 0.042372052 | 2.425058028 |
| ST8SIA4 | 0.010631157 | 0.432632469 | FAIM | 0.003442703 | 2.426423921 |
| CRISPLD2 | 0.004856073 | 0.433031779 | HP09053 | 0.009096635 | 2.428798126 |
| EPHX4 | 0.017977434 | 0.433114661 | HES7 | 0.022623037 | 2.430896487 |
| KRT17 | 0.017007723 | 0.433149949 | ZC3H8 | 0.001271253 | 2.432583465 |
| TNFRSF10D | 0.033338306 | 0.43339544 | IL32 | 0.021956869 | 2.440233363 |
| OCEL1 | 0.001720532 | 0.43351214 | PYGB | 0.003152872 | 2.442237667 |
| MIR210HG | 0.042990958 | 0.433584309 | STON2 | 0.041430567 | 2.443246304 |
| SMAD9 | 0.00240805 | 0.433660447 | TWIST2 | 0.02234126 | 2.450628925 |
| VWA1 | 0.025921851 | 0.433792315 | GADD45A | 0.028187975 | 2.453577605 |
| TMEM2 | 0.002250407 | 0.43386166 | RAD9B | 0.013072978 | 2.460161464 |
| SLC4A4 | 0.001044802 | 0.434306938 | CCND2-AS1 | 0.009305406 | 2.462964484 |
| RHOJ | 0.00164067 | 0.434843923 | STAG3 | 0.024374302 | 2.464245674 |
| OLFML2B | 0.007830121 | 0.434983482 | MXD1 | 0.000316888 | 2.466044972 |
| LOC101927181 | 0.011082696 | 0.435079841 | PSMB8 | 0.003806645 | 2.469382665 |
| MSMO1 | 0.014988337 | 0.43525676 | STK24 | 0.006460443 | 2.46960411 |
| EFHD1 | 0.039922287 | 0.435457203 | IRF9 | 6.79E-06 | 2.472810195 |
| ATP6V0E2 | 8.35E-05 | 0.435945791 | CSPG4 | 0.000336786 | 2.472961638 |
| PROM1 | 0.013229747 | 0.436178507 | NUDT6 | 0.002107086 | 2.473676408 |
| ARID5B | 0.004616419 | 0.436620005 | CYP11A1 | 0.001719516 | 2.478409202 |
| DHRS1 | 0.002114125 | 0.437107328 | ALOX5 | 0.00469695 | 2.485433923 |
| HSD11B1L | 0.036250698 | 0.437425743 | ARRDC3 | 7.91E-05 | 2.489480007 |
| LBH | 2.73E-05 | 0.437719996 | FRAS1 | 0.044347355 | 2.496757138 |
| CCDC167 | 0.015855258 | 0.437937991 | TMCO4 | 0.000733137 | 2.497529875 |
| NINJ2 | 0.030204504 | 0.438637524 | SNAP91 | 0.042950435 | 2.501893364 |
| INSR | 0.011129957 | 0.438645623 | TLR3 | 0.002124096 | 2.502511801 |
| SATB1 | 0.010703657 | 0.438917609 | RPP40 | 0.000690287 | 2.510140738 |
| ALAD | 0.001035882 | 0.439011606 | SYT1 | 0.001280568 | 2.510186107 |
| VCAN | 0.001709413 | 0.439092 | P2RX4 | 0.00144413 | 2.511805331 |
| SLC2A4 | 0.045518308 | 0.439136045 | PLIN2 | 0.016181337 | 2.512626289 |
| KBTBD3 | 0.011112835 | 0.43941999 | ISLR | 0.017166631 | 2.514040892 |
| LPPR2 | 0.001252533 | 0.439570035 | SAT1 | 0.00300595 | 2.514287363 |
| VAMP1 | 0.0011288 | 0.439626771 | GGT5 | 0.002322233 | 2.516666455 |
| TPGS1 | 0.009021787 | 0.440139297 | SYT14 | 0.030403178 | 2.51860578 |
| RORB | 0.041459994 | 0.440300452 | MAFF | 0.032724972 | 2.526054282 |
| FKBP14 | 0.012359677 | 0.441117296 | MYO15B | 0.011533733 | 2.526337048 |
| TRPV4 | 0.000417115 | 0.441212134 | CXCL16 | 0.001437038 | 2.526963609 |
| CHST6 | 0.012458606 | 0.441647705 | LRRC61 | 0.030250252 | 2.528284538 |
| RARB | 0.00017927 | 0.442233489 | PLXNC1 | 0.005578199 | 2.530041676 |
| EFNA4 | 0.001157884 | 0.442962823 | IL34 | 0.004405463 | 2.530906223 |
| TSPAN13 | 0.022875758 | 0.444198382 | NRROS | 0.020614231 | 2.535195093 |
| NFATC4 | 0.001341706 | 0.444569605 | SP110 | 9.49E-05 | 2.538882555 |
| VASH1 | 0.000848744 | 0.44481316 | HDAC9 | 0.00308625 | 2.541873604 |
| ELFN2 | 0.003375999 | 0.444843276 | C12orf66 | 0.042450352 | 2.543307537 |
| CLSTN2 | 0.034196561 | 0.445169463 | EFHB | 0.039313577 | 2.543397169 |
| HMGCR | 0.027110753 | 0.445193551 | N4BP3 | 0.005265514 | 2.544366804 |
| CGNL1 | 0.005642992 | 0.445209616 | FAM86HP | 0.00183562 | 2.545243845 |
| KIAA1161 | 0.010691508 | 0.445673104 | EML1 | 0.005380988 | 2.545901724 |
| GIPC3 | 0.011566444 | 0.446062234 | TRPC3 | 0.000446036 | 2.546302324 |
| ACTN1 | 0.020293513 | 0.446093474 | TRABD2A | 0.042841194 | 2.546928825 |
| ECM1 | 0.018067399 | 0.446116329 | PLCG2 | 0.003598958 | 2.547170971 |
| MAGEH1 | 0.004737274 | 0.446952221 | DDIT3 | 0.003974815 | 2.548253473 |
| COX6B1 | 0.016080742 | 0.44708888 | PNPT1 | 4.96E-06 | 2.550478095 |
| PCYT2 | 0.022244004 | 0.447328696 | NT5C3A | 0.001735781 | 2.553563985 |
| FAM26E | 0.007141131 | 0.447350202 | FCRLB | 0.000544319 | 2.559064446 |
| WNT4 | 0.019772616 | 0.447843562 | PSME2 | 0.009787 | 2.573354744 |
| TRIM9 | 0.027282025 | 0.44784558 | CFAP53 | 0.014499529 | 2.57509888 |
| DENND2C | 0.009989941 | 0.447962787 | CELF6 | 0.009693585 | 2.575149944 |
| QPCTL | 0.006518314 | 0.448049839 | PABPC1L | 0.002530665 | 2.575677685 |
| TSPAN7 | 0.047821252 | 0.448390095 | IQCH | 0.015251452 | 2.576626188 |
| SBK1 | 0.006177422 | 0.448562794 | FAM86B1 | 0.00735363 | 2.581494318 |
| SUGCT | 0.000433651 | 0.449115754 | ADTRP | 0.005611628 | 2.581535498 |
| TM4SF1 | 0.007386524 | 0.449257205 | CRMP1 | 0.002956046 | 2.583661581 |
| FLJ16779 | 0.000875638 | 0.449711166 | LOC101927746 | 0.012822888 | 2.584129436 |
| GDPD5 | 0.00242683 | 0.450130163 | ZNF239 | 0.00240722 | 2.585579416 |
| FDFT1 | 0.02205753 | 0.450245406 | E2F7 | 0.001839054 | 2.587401794 |
| SLC5A3 | 0.001752796 | 0.450877126 | HSPA9 | 0.000142027 | 2.587985177 |
| MYB | 0.011729505 | 0.451586406 | ARHGEF16 | 0.017287898 | 2.594268034 |
| BMP4 | 0.000604333 | 0.451727331 | HIST2H3D | 0.021238425 | 2.595131889 |
| SORT1 | 0.001770774 | 0.452021986 | NKX3-1 | 0.001785715 | 2.600985894 |
| SLC39A10 | 0.007410614 | 0.45208755 | MBNL3 | 0.004602514 | 2.603143366 |
| DPF1 | 0.019885622 | 0.452287665 | KL | 0.016276078 | 2.608880616 |
| SLIT3 | 0.023974769 | 0.452308094 | EPB41L4A-AS1 | 0.000200723 | 2.608948681 |
| GBGT1 | 0.002378596 | 0.45256132 | PKD1L1 | 0.024392368 | 2.609531573 |
| CNN3 | 0.003695586 | 0.452932546 | PIWIL4 | 0.009990409 | 2.611761677 |
| CCDC28B | 0.003385047 | 0.453237894 | MSC | 0.002223414 | 2.613225241 |
| BACH2 | 0.0347217 | 0.453976897 | CSF1 | 3.47E-05 | 2.616618674 |
| COL8A1 | 0.0002424 | 0.454257243 | MFSD7 | 0.047533875 | 2.620110452 |
| RTN2 | 0.000667117 | 0.454700389 | CCDC158 | 0.015367585 | 2.623667796 |
| ADAM23 | 0.000531995 | 0.455177843 | C9orf43 | 0.035510736 | 2.638611591 |
| NEGR1 | 0.013551451 | 0.455213707 | TRIM22 | 0.007900289 | 2.639342096 |
| FAM63A | 0.019144685 | 0.455237276 | LOC100506207 | 0.041039788 | 2.647643185 |
| DYRK1B | 0.000658892 | 0.455289098 | UST | 0.018037109 | 2.64958104 |
| SCUBE2 | 0.049225621 | 0.455388576 | PDZK1IP1 | 0.021583781 | 2.650365163 |
| QARS | 0.000124084 | 0.455397456 | SLC13A3 | 9.79E-05 | 2.651735433 |
| HSD17B7P2 | 0.008129801 | 0.455450381 | MST1R | 0.004950482 | 2.65345758 |
| CNIH2 | 0.038900583 | 0.456121406 | LOC100506801 | 0.031795647 | 2.657400172 |
| GPC4 | 0.00659743 | 0.456137596 | FLRT1 | 0.015382216 | 2.662639166 |
| SELENBP1 | 0.006331416 | 0.456148969 | PRDM8 | 0.009245602 | 2.662977452 |
| SLC25A42 | 0.004794585 | 0.456476146 | TOP1 | 1.10E-05 | 2.666731417 |
| LINC00672 | 0.032538196 | 0.456594327 | ARHGEF2 | 0.001598277 | 2.678144509 |
| LRRN1 | 0.008535111 | 0.456754782 | TNFAIP2 | 0.006165914 | 2.681315791 |
| FMN2 | 0.003226413 | 0.457112955 | SPTBN5 | 0.005158386 | 2.691874583 |
| NR4A1 | 0.013680746 | 0.45744215 | FYN | 0.00103546 | 2.692422675 |
| GJA1 | 0.00177068 | 0.457498308 | TNFRSF14 | 0.000177937 | 2.702208996 |
| DHRS2 | 0.03419185 | 224.6715209 | LOC101929555 | 0.038814979 | 2.705067479 |
| CDCP1 | 0.000740512 | 2.707560852 | PARP10 | 0.000120288 | 4.159057879 |
| CMKLR1 | 0.000421811 | 2.707702064 | KCNJ5 | 0.013078806 | 4.162173356 |
| TCEA1 | 4.04E-05 | 2.708191933 | LINC01152 | 0.005363312 | 4.171178786 |
| STYK1 | 0.009987135 | 2.710230998 | TMEM52 | 0.04000106 | 4.171216695 |
| HSPA5 | 0.011751357 | 2.711836768 | KLF8 | 0.001566316 | 4.180037216 |
| SYK | 0.025179864 | 2.712760292 | PARP14 | 0.004983391 | 4.181763619 |
| ZNF442 | 0.029287605 | 2.71309244 | SLC7A2 | 0.001952717 | 4.185935515 |
| ASS1 | 0.004402149 | 2.714090374 | MILR1 | 0.016804943 | 4.198323317 |
| SNHG1 | 0.004972946 | 2.716451274 | WBP2NL | 0.000888793 | 4.211559606 |
| TMC6 | 0.001187539 | 2.720055544 | SYN1 | 0.015825601 | 4.2151619 |
| PCLO | 0.001035927 | 2.724849077 | LY6E | 0.000389236 | 4.219900491 |
| ADD2 | 0.032895595 | 2.725955572 | FAM19A5 | 0.04628217 | 4.221647224 |
| C1S | 0.004875357 | 2.729916562 | RASGRP3 | 0.00259134 | 4.231342214 |
| SUSD3 | 0.013804606 | 2.733425116 | LYPD5 | 0.00417971 | 4.253787818 |
| PPP1R26-AS1 | 0.011799384 | 2.73475014 | STAT1 | 0.003193422 | 4.266209792 |
| RHOU | 0.002060051 | 2.745617843 | IP6K3 | 0.01073424 | 4.272845229 |
| GALNT12 | 0.029128447 | 2.746680795 | LOC101927040 | 0.011916381 | 4.341609869 |
| CYP2J2 | 0.001280621 | 2.746916219 | TAS2R5 | 0.004077697 | 4.354555266 |
| SEPHS2 | 0.000484972 | 2.751974874 | FOXP3 | 0.036589298 | 4.406072957 |
| SYTL3 | 0.000142304 | 2.752144143 | ABCG2 | 0.020891039 | 4.411062327 |
| EIF2AK2 | 0.005962417 | 2.753800525 | ADRB1 | 0.01917506 | 4.430140854 |
| HPSE | 0.035478842 | 2.755487704 | CASP1 | 0.000136091 | 4.443064289 |
| EID3 | 0.021368833 | 2.756211907 | SAMHD1 | 0.005088352 | 4.449262339 |
| SGPP2 | 0.030072949 | 2.757285156 | C14orf39 | 0.006282111 | 4.450633958 |
| THBS4 | 0.04455436 | 2.759192939 | NGF | 0.042885384 | 4.474385651 |
| ZC3H12D | 0.035830706 | 2.760457137 | P2RX7 | 0.001811832 | 4.475008366 |
| CACNA1A | 0.044873792 | 2.766248059 | DRD2 | 0.010171023 | 4.477271808 |
| SMOX | 0.00797025 | 2.766465964 | DDX58 | 0.002899656 | 4.483661101 |
| ADPRH | 0.000254758 | 2.772585808 | HAPLN4 | 0.011919138 | 4.489789474 |
| PTPRN2 | 0.004514917 | 2.776917213 | MMP13 | 0.001289274 | 4.498578341 |
| ODC1 | 0.002268456 | 2.779994586 | TNFRSF1B | 3.20E-06 | 4.507404582 |
| NFIL3 | 0.003336011 | 2.782069007 | TRIM36 | 0.000359928 | 4.514639817 |
| ELFN1 | 0.024270846 | 2.785152001 | DDX60L | 7.27E-05 | 4.53579358 |
| ARL4D | 0.014243157 | 2.785809119 | SLC16A9 | 0.000577415 | 4.545190925 |
| C17orf67 | 0.003221284 | 2.788364785 | GCH1 | 0.019509101 | 4.594982647 |
| PHLDA1 | 0.000720103 | 2.790847058 | LONRF3 | 0.003303843 | 4.619776255 |
| NUB1 | 0.001876695 | 2.792852614 | GJD3 | 0.006830436 | 4.630257104 |
| SYNE3 | 0.000973578 | 2.799772776 | ESAM | 0.015859009 | 4.633025332 |
| ESRP2 | 0.003822545 | 2.803278468 | IRF1 | 1.49E-06 | 4.635063395 |
| HGD | 0.033128301 | 2.805323095 | CASQ1 | 0.008383608 | 4.643332074 |
| ATP2B1 | 8.59E-05 | 2.81034572 | GPR1 | 0.003064968 | 4.653636095 |
| ANGPTL1 | 0.016186167 | 2.812474561 | GCSAML-AS1 | 0.022405551 | 4.660927257 |
| LOC101926963 | 0.045995932 | 2.814681932 | CPEB1 | 0.00043649 | 4.694205164 |
| AKNA | 0.00347955 | 2.821140697 | TNNT1 | 0.030876058 | 4.700721545 |
| MAL2 | 0.013726314 | 2.822169584 | DUSP5P1 | 0.020506531 | 4.708366989 |
| TMEM140 | 0.000473762 | 2.830362453 | LOC101060389 | 0.015646257 | 4.711102515 |
| SLC27A6 | 0.042963293 | 2.832559526 | ICOSLG | 0.020247853 | 4.739170763 |
| SLC43A3 | 0.001529512 | 2.847300858 | C5orf56 | 1.54E-06 | 4.741337922 |
| GOT1 | 0.003644518 | 2.853635137 | GS1-259H13.2 | 0.041987123 | 4.742870293 |
| PDZD2 | 0.00426888 | 2.856069723 | CXCL1 | 0.020685265 | 4.756566831 |
| IDNK | 0.022387024 | 2.858919538 | INHBE | 0.000998057 | 4.762232143 |
| HIST2H2BA | 0.008620715 | 2.865810858 | TMEM132D | 0.023409912 | 4.794370529 |
| ACTG1P4 | 0.022260686 | 2.869758542 | MSX1 | 0.006461963 | 4.829792821 |
| FBXO6 | 0.000276919 | 2.870585857 | ABLIM3 | 0.0312638 | 4.847066318 |
| NAGS | 0.009300497 | 2.872851588 | DLX1 | 0.021223433 | 4.86540646 |
| C5AR1 | 0.036785543 | 2.873910559 | ANKRD31 | 0.019996335 | 4.867196753 |
| ADGRF4 | 0.003038967 | 2.880299875 | KCNJ6 | 0.041119794 | 4.892362715 |
| GPR146 | 0.049853054 | 2.884431333 | PDE4B | 0.000503872 | 4.904535642 |
| SLC38A5 | 0.004163789 | 2.885792051 | RIMS2 | 0.000606366 | 4.914811844 |
| HIST2H2BF | 0.025760973 | 2.885951806 | CLDN7 | 0.00319999 | 4.917634526 |
| MYH3 | 0.036482371 | 2.886463366 | CEACAM1 | 0.001032446 | 4.927822922 |
| TECTA | 0.042228357 | 2.886928586 | MT1X | 0.012389712 | 4.942781095 |
| APOL6 | 0.003709698 | 2.888228641 | FAM167A | 0.002662685 | 4.956982273 |
| FAM110B | 0.001773686 | 2.890867552 | CXCL5 | 0.015436692 | 4.963821286 |
| ISL2 | 0.042821935 | 2.893633212 | NGEF | 0.000842959 | 5.013162047 |
| LINC01547 | 0.029640408 | 2.89499456 | CCNA1 | 0.026422098 | 5.029539462 |
| LBX2 | 0.041770071 | 2.896817638 | LINC00571 | 0.004770019 | 5.058530139 |
| PRKCQ-AS1 | 0.000161936 | 2.902742646 | PAPPA2 | 0.003406686 | 5.142447734 |
| ANKRD7 | 0.013175908 | 2.910608315 | CATSPER3 | 0.042705872 | 5.2016744 |
| NRSN2-AS1 | 0.04199775 | 2.913885333 | OGFRP1 | 0.040889233 | 5.203651536 |
| SGTB | 0.000102802 | 2.914808474 | NRCAM | 0.016348513 | 5.204316868 |
| SH2D2A | 0.005072188 | 2.915105007 | TNRC6C-AS1 | 8.26E-05 | 5.218459956 |
| CLDN1 | 0.020270127 | 2.915106946 | TMEM254-AS1 | 0.017299153 | 5.226846394 |
| PDCD1LG2 | 0.021826001 | 2.916527843 | IRF7 | 0.006183171 | 5.24162147 |
| ARHGAP4 | 0.002837142 | 2.944998386 | IL18RAP | 0.029725027 | 5.305437277 |
| ATF3 | 0.00992097 | 2.945600244 | ESRP1 | 0.017214338 | 5.320114536 |
| HIST1H2AC | 0.000133073 | 2.94614854 | NRL | 0.048376067 | 5.32296952 |
| FOXA3 | 0.019697432 | 2.950533507 | MT1E | 0.001544491 | 5.33571767 |
| AMDHD1 | 0.018440944 | 2.951154658 | CDH4 | 0.010496659 | 5.392006929 |
| PLA2G4C | 0.011484455 | 2.95172016 | SPX | 0.024362767 | 5.414461462 |
| CCDC152 | 0.00576804 | 2.952589154 | IL15RA | 0.012926116 | 5.432758121 |
| NAMPT | 0.010579279 | 2.962777355 | CH25H | 0.046125721 | 5.473131828 |
| HLA-C | 8.81E-05 | 2.965052815 | ERICH2 | 0.033342842 | 5.491734873 |
| AHNAK2 | 0.022784112 | 2.965527685 | SAMD9 | 0.001829035 | 5.49657407 |
| C9orf50 | 0.048434723 | 2.968134353 | IL18R1 | 0.003232607 | 5.501738554 |
| LAP3 | 0.001532764 | 2.984976722 | LINC00341 | 0.000810704 | 5.508830116 |
| MGC70870 | 0.004380053 | 2.987952673 | OR52K2 | 0.035884669 | 5.519040525 |
| RSPO4 | 0.01093053 | 2.990753805 | NCF1 | 0.015875055 | 5.5295997 |
| DLL1 | 0.001917012 | 2.994682615 | PRR18 | 0.004182195 | 5.553454635 |
| AIFM2 | 1.09E-05 | 2.997199347 | THSD7B | 0.03613608 | 5.581147649 |
| ERN1 | 0.000224945 | 2.999100724 | L3MBTL4 | 6.43E-05 | 5.587899152 |
| ITPR3 | 0.010600316 | 2.99975339 | UPP1 | 0.012983189 | 5.665952404 |
| ZCCHC2 | 0.006203807 | 3.000366969 | CXCL6 | 0.022469273 | 5.706626151 |
| DLGAP1-AS2 | 0.036670812 | 3.000724072 | ITIH4 | 0.044508588 | 5.708083964 |
| ARHGAP9 | 0.020116115 | 3.001011595 | ZDHHC11 | 0.011300709 | 5.709381098 |
| KCNJ14 | 0.019643748 | 3.008120936 | DDIT4L | 0.028797654 | 5.729115068 |
| FZD8 | 0.034751 | 3.013727517 | AP3B2 | 0.013270965 | 5.733415817 |
| PRKCE | 0.001289621 | 3.014461426 | POU5F1B | 0.007436658 | 5.733907847 |
| RAB3IL1 | 0.004888181 | 3.035714722 | SECTM1 | 0.023414535 | 5.738300259 |
| LINC00574 | 0.040263018 | 3.035986907 | STRA6 | 0.003714543 | 5.742339203 |
| NUDT16P1 | 0.003244339 | 3.041849184 | LY75 | 0.01887291 | 5.747288557 |
| SH3TC1 | 0.005766079 | 3.047710115 | EPSTI1 | 0.000572063 | 5.766495667 |
| SLC9C1 | 0.03369562 | 3.0526937 | C16orf74 | 0.011297517 | 5.810228889 |
| MISP | 0.003822338 | 3.05591284 | VIP | 0.014438953 | 5.815625602 |
| GAS5 | 0.017301105 | 3.056915009 | KLF4 | 0.003119659 | 5.889347017 |
| SERPINA3 | 0.000384739 | 3.058973022 | NTRK1 | 0.02407574 | 5.905266969 |
| TRIB3 | 0.000742486 | 3.064640326 | IFI30 | 0.019030104 | 5.929226845 |
| ADGRE2 | 0.003315813 | 3.07685718 | IL1RL2 | 0.00476573 | 5.954449426 |
| LOC100294362 | 0.007007863 | 3.077420319 | STEAP1 | 0.026180414 | 5.971730711 |
| AIM1 | 8.60E-06 | 3.081408427 | IFIT2 | 0.003544904 | 6.052436053 |
| IRAK3 | 0.003629142 | 3.085420727 | LOC101928796 | 0.041900674 | 6.074992538 |
| ADAMTS9 | 0.000314816 | 3.096618186 | PDE2A | 0.022636697 | 6.096372791 |
| LOC101929224 | 0.040497305 | 3.100297129 | C8orf31 | 0.003388078 | 6.137091353 |
| TLR9 | 0.013602364 | 3.109628706 | TMIE | 0.006567308 | 6.18088638 |
| BAIAP2L2 | 0.00038014 | 3.112175584 | ZNF385C | 0.030945535 | 6.225647842 |
| VCAM1 | 0.009223836 | 3.115277794 | ARHGAP40 | 0.029432195 | 6.303176199 |
| BEST1 | 0.015846621 | 3.131194608 | RAB19 | 0.014520203 | 6.31596053 |
| ATP2A3 | 0.007931305 | 3.142932027 | CYP26A1 | 0.001730125 | 6.394825251 |
| NKAPL | 0.024279915 | 3.152813459 | CD38 | 0.003568833 | 6.411110749 |
| OXCT2 | 0.002379352 | 3.154134965 | RTP4 | 0.010874385 | 6.485629084 |
| FLRT2 | 0.013945539 | 3.16415719 | GBP4 | 0.011263017 | 6.492086751 |
| C3AR1 | 0.041056637 | 3.166339852 | TMEM71 | 0.002611881 | 6.493478522 |
| RNF213 | 0.015486281 | 3.167303061 | CCAT1 | 0.015801431 | 6.55900922 |
| SLC16A14 | 0.015357651 | 3.172164794 | SLC6A12 | 0.007560539 | 6.585816914 |
| TM4SF19 | 0.029402365 | 3.175672961 | SLCO4A1 | 0.016604017 | 6.694086584 |
| SKAP1 | 0.033632191 | 3.183208717 | IL4I1 | 0.01006862 | 6.713162399 |
| FUT1 | 0.010074845 | 3.184975553 | LURAP1L | 0.000696499 | 6.713413816 |
| HLA-B | 4.07E-05 | 3.185111872 | TSLP | 0.010734824 | 6.717434879 |
| SH3RF3-AS1 | 0.004787012 | 3.186383986 | ISLR2 | 0.000161575 | 6.727067173 |
| LOC645752 | 0.016140393 | 3.188983072 | HKDC1 | 0.005257529 | 6.808765651 |
| MDK | 0.003889935 | 3.198807702 | EPB41L4B | 0.042259131 | 6.874610417 |
| LRRC46 | 0.006608493 | 3.199287943 | EPHB1 | 0.011439173 | 6.919050691 |
| FAM86JP | 0.009139662 | 3.202631775 | TH | 0.027853388 | 6.960972127 |
| SH3RF3 | 2.88E-05 | 3.212202215 | LOC101927497 | 0.026722967 | 6.961695443 |
| ZFP42 | 0.009743085 | 3.221277639 | SRRM2-AS1 | 0.01203237 | 6.980017752 |
| GMPR | 1.79E-05 | 3.222567328 | TRIM69 | 0.030959121 | 7.112484945 |
| ZNFX1 | 0.000667772 | 3.22854309 | CXCL11 | 0.011949158 | 7.115088724 |
| CUZD1 | 0.035528589 | 3.241044431 | COL15A1 | 0.04465195 | 7.16535623 |
| STAC | 0.001968041 | 3.242150076 | TMEM196 | 0.041170525 | 7.226766795 |
| VEGFA | 0.001153166 | 3.244076996 | TMEM200C | 0.01534134 | 7.238501377 |
| DHX58 | 0.000528796 | 3.247831042 | PLSCR1 | 0.002688057 | 7.253804657 |
| NKAIN1 | 0.007049186 | 3.253961244 | ADGRG2 | 0.019258245 | 7.283995737 |
| SYBU | 0.000314357 | 3.264263119 | KRT86 | 0.010862549 | 7.353927592 |
| CCDC169 | 0.034252591 | 3.26822505 | LOC644554 | 0.009294114 | 7.375380828 |
| CYP4V2 | 0.019486776 | 3.274192207 | SLPI | 0.028793833 | 7.379354587 |
| HIST1H4K | 0.043807002 | 3.274749957 | PTPRH | 0.016926579 | 7.385395204 |
| ARRDC4 | 0.000641372 | 3.289748297 | HERC6 | 0.000611322 | 7.423447003 |
| RND3 | 0.00015774 | 3.305983106 | HLA-F | 9.04E-05 | 7.544122165 |
| BATF3 | 0.03920463 | 3.310188609 | CPO | 0.015458909 | 7.546414425 |
| SLC16A6 | 0.000544783 | 3.314022297 | DDX60 | 0.000514944 | 7.560849292 |
| GLI1 | 0.000926957 | 3.326794184 | ACSL5 | 0.007421981 | 7.570341115 |
| RBCK1 | 0.001711454 | 3.336123357 | MT1F | 6.39E-05 | 7.654870849 |
| LIPE-AS1 | 0.023844204 | 3.342896169 | TIMP4 | 0.000130707 | 7.660129932 |
| FAM129A | 0.000516107 | 3.344526917 | CXCL8 | 0.039945372 | 7.677213548 |
| LOC100506368 | 0.010562633 | 3.348868933 | ETV7 | 0.006379411 | 7.687980722 |
| MRPL42P5 | 0.03616127 | 3.359791422 | IFIT3 | 0.001733347 | 7.707914976 |
| IFIT5 | 6.50E-05 | 3.364316997 | USP30-AS1 | 0.031647495 | 7.754136468 |
| TRIM14 | 0.002396734 | 3.375345932 | TNFRSF9 | 0.004792764 | 7.781006311 |
| LIPC | 0.007074004 | 3.392836224 | BATF2 | 8.00E-05 | 7.799852116 |
| APOBEC3D | 0.001055336 | 3.398687325 | EXOC3L4 | 0.016326098 | 7.842088278 |
| LOC100287072 | 0.006398271 | 3.417240376 | LINC01512 | 0.011679327 | 7.876964883 |
| GDA | 0.011432011 | 3.418451826 | LTK | 0.002643422 | 7.904729061 |
| ANGPTL4 | 0.03877272 | 3.419544508 | S100A14 | 0.020228457 | 7.922137515 |
| SCN9A | 0.017088373 | 3.419884184 | PCDH17 | 0.00353347 | 7.977330646 |
| RPP25 | 0.002892747 | 3.423011244 | IFIH1 | 0.000450934 | 8.071907108 |
| ACKR4 | 0.032522582 | 3.427013842 | CXCL3 | 0.015927761 | 8.333493632 |
| GCNT7 | 0.002172297 | 3.450901436 | SLC6A15 | 0.014943175 | 8.334385821 |
| NMI | 0.000736136 | 3.459163595 | NGFR | 0.010730481 | 8.371656963 |
| PLA1A | 0.000967128 | 3.472781872 | OAS1 | 0.000562287 | 8.391629112 |
| AOX1 | 0.00528479 | 3.474551602 | LOC101927468 | 0.006821844 | 8.43789315 |
| DTX3L | 0.000149447 | 3.481370969 | EDAR | 0.015412173 | 8.48916859 |
| SNHG15 | 0.014177781 | 3.482349529 | HYDIN | 0.017225359 | 8.642095779 |
| C2 | 0.003194614 | 3.486052049 | LOC101926975 | 0.005537758 | 8.691989419 |
| DNM1P35 | 0.001081548 | 3.48927644 | TBX15 | 0.005962564 | 8.791893971 |
| F13A1 | 0.018893082 | 3.498149427 | HIST1H4J | 0.008633782 | 8.979165236 |
| MAPK8IP2 | 0.001398876 | 3.499338283 | IFIT1 | 0.003615969 | 8.990618078 |
| RASGRP2 | 0.003569957 | 3.49973476 | RGS16 | 0.008467589 | 9.00508845 |
| CDRT1 | 0.046559645 | 3.505787939 | LINC01587 | 0.029855607 | 9.084628519 |
| PKN2-AS1 | 0.048710513 | 3.508811406 | HYDIN2 | 0.006510033 | 9.212276348 |
| RGS7 | 0.003974694 | 3.510336349 | RANBP3L | 0.035754046 | 9.247874951 |
| CPT1B | 0.002413717 | 3.525400747 | IFITM1 | 5.82E-05 | 9.40594608 |
| LOC100419583 | 0.000901001 | 3.535797668 | SAA1 | 0.001529309 | 9.434325953 |
| CAPN3 | 0.031897597 | 3.538296338 | HIST2H3C | 0.000329962 | 9.488561656 |
| TAP1 | 0.000531226 | 3.552923017 | HIST2H3A | 0.000329962 | 9.488561656 |
| EPAS1 | 0.007979792 | 3.558938963 | PNMT | 0.015426654 | 9.521253294 |
| B3GNT3 | 0.024188711 | 3.566652238 | BHMT | 0.021386626 | 9.580049138 |
| MKX | 0.010672049 | 3.567650229 | KLK1 | 0.021562783 | 9.777292814 |
| PPP1R15A | 0.00461198 | 3.583933379 | LINC01133 | 0.003001155 | 9.817152184 |
| FGF2 | 0.000228115 | 3.589894193 | SLC13A5 | 0.032395374 | 9.826358664 |
| PTGER1 | 0.003772192 | 3.591993147 | USP18 | 0.000530487 | 9.849333657 |
| HELZ2 | 0.002654767 | 3.593216649 | SAMD9L | 0.002397407 | 9.946395073 |
| CA2 | 0.01862583 | 3.595206558 | CXCL2 | 0.001783864 | 10.29260029 |
| PLXNA2 | 0.00085802 | 3.599428658 | CH17-408M7.1 | 0.015924808 | 10.41058404 |
| VLDLR-AS1 | 0.035985131 | 3.60783863 | POU2F2 | 0.019989988 | 10.53817001 |
| TMEM27 | 0.012484095 | 3.612512156 | EREG | 0.002181361 | 10.57872879 |
| SLC30A1 | 0.000927346 | 3.613541894 | SGCG | 0.014425578 | 10.72411493 |
| UBAC2-AS1 | 0.008310468 | 3.631699968 | SCN4A | 0.028403962 | 10.73990283 |
| CLDN23 | 0.006270124 | 3.636419804 | H1F0 | 0.001009562 | 10.80553678 |
| HCG26 | 0.037266258 | 3.642706588 | AREG | 0.005446317 | 11.03862592 |
| LINC00865 | 0.003978632 | 3.648553011 | CCL20 | 0.012457995 | 11.05605901 |
| LINC00936 | 0.005253943 | 3.652238204 | IL20RB | 6.90E-06 | 11.20874195 |
| PARP9 | 0.004835356 | 3.657896596 | TNFSF13B | 0.014046694 | 11.32889765 |
| MOCOS | 0.00481104 | 3.659887396 | DUSP15 | 0.022378005 | 11.64327099 |
| SLC22A15 | 0.000319765 | 3.665424175 | SLC10A5 | 0.004873439 | 11.8043842 |
| TXNIP | 0.000411299 | 3.66969867 | SAA2 | 0.002569323 | 11.99449615 |
| SLFN5 | 0.013236357 | 3.690822599 | IFI44 | 0.004345449 | 12.09099673 |
| HSF5 | 0.00734249 | 3.697980306 | SLC15A3 | 0.003368819 | 12.28538567 |
| PSMB9 | 0.004076388 | 3.710165898 | WIPF3 | 0.014732246 | 12.29265798 |
| CPT1A | 0.032556368 | 3.725078694 | XAF1 | 8.90E-05 | 12.49028875 |
| ITPR1 | 0.016968099 | 3.728499041 | IL6 | 0.007099583 | 12.74586625 |
| LINC01234 | 0.028260145 | 3.730050728 | ISG20 | 0.006688385 | 12.82898271 |
| SPATA41 | 0.033730711 | 3.740431728 | ITPKA | 0.011365833 | 12.86302155 |
| UBE2L6 | 0.00218093 | 3.744890829 | LOC101927630 | 0.000423809 | 12.86759586 |
| SMC1B | 0.03294107 | 3.749453889 | TYMP | 0.006578686 | 13.03377279 |
| UNC5B | 0.000135523 | 3.763687004 | CMPK2 | 0.002780448 | 13.04002515 |
| DUSP5 | 0.002579888 | 3.764572369 | PATL2 | 0.039613099 | 13.40680672 |
| NEB | 0.041539301 | 3.76548686 | DMGDH | 0.035802641 | 13.52166541 |
| IFNB1 | 0.025631714 | 3.790221615 | IFI6 | 0.002542316 | 13.70515109 |
| IFI35 | 0.002510167 | 3.79625374 | GBP1P1 | 0.017428723 | 13.80331915 |
| FMNL1 | 0.029581424 | 3.812039517 | G0S2 | 0.025640387 | 14.03664834 |
| ADCY4 | 0.037992141 | 3.819171954 | C3 | 0.002608046 | 14.23676755 |
| FAM132B | 0.010737434 | 3.826916946 | OAS3 | 0.002162476 | 14.27982466 |
| TPH1 | 0.037478897 | 3.835978616 | RPLP0P2 | 0.001773039 | 14.37559709 |
| WARS | 0.002003179 | 3.850248566 | TMPRSS3 | 0.039103161 | 14.39710157 |
| LOC101929705 | 0.016340508 | 3.858711077 | PTX3 | 0.001529213 | 14.5096174 |
| APOBEC3G | 0.00756124 | 3.870528455 | MX1 | 0.000630729 | 14.60977616 |
| ETS2 | 0.000182875 | 3.876060836 | SYT7 | 0.030767521 | 15.26147367 |
| AKAP3 | 0.002352301 | 3.882679213 | HERC5 | 0.007556521 | 15.53132099 |
| LRRC49 | 0.000326635 | 3.884239458 | LGALS9 | 0.006972455 | 15.63094468 |
| SOX30 | 0.037100672 | 3.884790566 | ALMS1P | 0.013556464 | 15.80517714 |
| BTC | 0.007550773 | 3.890168857 | RNF165 | 0.010458841 | 17.35197055 |
| TPTE2 | 0.013329245 | 3.897887682 | ISG15 | 0.004764151 | 18.29381237 |
| MYHAS | 0.000811203 | 3.898140016 | PCDH1 | 0.004205707 | 18.38186278 |
| TMEM229B | 0.00102921 | 3.901587454 | IFI44L | 0.00902662 | 19.46965905 |
| ZBED3-AS1 | 0.029422017 | 3.902311027 | CFB | 0.015659861 | 19.76959893 |
| HCP5 | 3.23E-05 | 3.908226946 | BIRC3 | 0.006667995 | 19.91376834 |
| KCNQ5 | 0.004009549 | 3.911260956 | CXCL10 | 0.047048707 | 20.32567936 |
| FRMD3 | 0.000573981 | 3.912352091 | OASL | 0.001464896 | 20.59538309 |
| APOL3 | 5.20E-05 | 3.913461037 | ODF3B | 0.001965819 | 20.67612069 |
| GAL | 0.011736573 | 3.916150448 | RAB39B | 5.12E-05 | 22.29717457 |
| PARP12 | 3.04E-06 | 3.916902711 | LAMP3 | 0.01697806 | 22.32860565 |
| GDF15 | 0.005162724 | 3.925729944 | MT1M | 1.19E-05 | 22.47759708 |
| SOHLH2 | 0.019072516 | 3.931930855 | RSAD2 | 0.001204992 | 25.62703578 |
| ZFAS1 | 0.020242826 | 3.931934061 | PRDM16 | 0.043114447 | 26.60849521 |
| SOD2 | 0.021027401 | 3.932001788 | HSH2D | 0.018190312 | 28.3818512 |
| TYRP1 | 0.049774091 | 3.955628928 | LCN2 | 0.021435876 | 29.58206789 |
| TRIM25 | 0.011745848 | 3.985227108 | TAC1 | 0.018471548 | 37.64166983 |
| FBLL1 | 0.000956855 | 3.99119527 | MX2 | 0.00071667 | 39.6627502 |
| RGS22 | 0.011501946 | 3.994439955 | VGF | 0.026109227 | 40.37633329 |
| FTCDNL1 | 0.036241399 | 4.011247482 | KLHDC7B | 0.002339399 | 44.5444081 |
| TNFRSF8 | 0.035673777 | 4.04060839 | FBXO39 | 0.016565949 | 44.70567088 |
| SPATA1 | 0.017448643 | 4.040989464 | OAS2 | 0.000619097 | 57.21897426 |
| SNHG5 | 0.009245566 | 4.044117014 | ZBP1 | 0.009445303 | 62.82367638 |
| NOV | 0.040523455 | 4.078046459 | LOC100133669 | 0.017139668 | 65.75391007 |
| AOC2 | 0.045598284 | 4.081644472 | IFI27 | 0.003597786 | 79.06240561 |

**Supplementary Table 4. 33 common DEGs and functions in 3 cell lines**

| **Common DEGs** | **Function summarized in NCBI** |
| --- | --- |
| DDX60 | This gene encodes a DEXD/H box RNA helicase that functions as an antiviral factor and promotes RIG-I-like receptor-mediated signaling. |
| ISG15 | The protein encoded by this gene is a ubiquitin-like protein that is conjugated to intracellular target proteins upon activation by interferon-alpha and interferon-beta. antiviral activity during viral infections |
| C3 | Complement component C3 plays a central role in the activation of complement system |
| IFIT5 | Enables nucleic acid binding activity. Involved in defense response to virus; negative regulation of viral genome replication |
| ISG20 | Enables 3'-5' exonuclease activity and RNA binding activity. Involved in defense response to virus; negative regulation of viral genome replication; and nucleobase-containing compound catabolic process. Located in cytoplasm and nuclear lumen. |
| DHX58 | Enables double-stranded RNA binding activity; single-stranded RNA binding activity; and zinc ion binding activity. Involved in negative regulation of defense response and negative regulation of type I interferon production. Predicted to be active in cytoplasm |
| DDX60L | In addition to functions in RNA metabolism, members of this family are involved in anti-viral immunity and act as cytosolic sensors of viral nucleic acids. The protein encoded by this gene has been shown to inhibit hepatitis C virus replication in response to interferon stimulation in cell culture. |
| IFIH1 | IFIH1 encodes MDA5 which is an intracellular sensor of viral RNA that triggers the innate immune response. |
| HERC5 | Pro-inflammatory cytokines upregulate expression of this gene in endothelial cells.The protein also acts as a modulator of the antiviral immune response. |
| IFIT3 | Enables identical protein binding activity. Involved in negative regulation of apoptotic process; negative regulation of cell population proliferation; and response to virus. Located in cytosol and mitochondrion. |
| IFIT2 | Enables RNA binding activity. Involved in negative regulation of protein binding activity; positive regulation of apoptotic process; and response to virus. Located in endoplasmic reticulum. |
| OAS1 | This gene is induced by interferons and encodes a protein that synthesizes 2',5'-oligoadenylates (2-5As). This protein activates latent RNase L, which results in viral RNA degradation and the inhibition of viral replication. |
| RSAD2 | The protein encoded by this gene is an interferon-inducible antiviral protein that belongs to the S-adenosyl-L-methionine (SAM) superfamily of enzymes. |
| DDX58 | It is involved in viral double-stranded (ds) RNA recognition and the regulation of the antiviral innate immune response. |
| HSH2D | T-cell activation requires 2 signals: recognition of antigen by the T-cell receptor (see TCR; MIM 186880) and a costimulatory signal provided primarily by CD28 (MIM 186760) in naive T cells. HSH2 is a target of both of these signaling pathways |
| TNFAIP3 | The protein encoded by this gene is a zinc finger protein and ubiqitin-editing enzyme, and has been shown to inhibit NF-kappa B activation as well as TNF-mediated apoptosis |
| IFIT1 | This gene encodes a protein containing tetratricopeptide repeats that was originally identified as induced upon treatment with interferon. The encoded protein may inhibit viral replication and translational initiation. |
| OAS3 | This enzyme family plays a significant role in the inhibition of cellular protein synthesis and viral infection resistance. |
| PPP1R15A | This gene is a member of a group of genes whose transcript levels are increased following stressful growth arrest conditions and treatment with DNA-damaging agents. The induction of this gene by ionizing radiation occurs in certain cell lines regardless of p53 status, and its protein response is correlated with apoptosis following ionizing radiation. |
| DTX3L | DTX3L functions as an E3 ubiquitin ligase |
| CMPK2 | This gene encodes one of the enzymes in the nucleotide synthesis salvage pathway that may participate in terminal differentiation of monocytic cells. |
| SAMD9 | The encoded protein localizes to the cytoplasm and may play a role in regulating cell proliferation and apoptosis |
| PARP9 | Enables several functions, including ADP-D-ribose binding activity; NAD+ ADP-ribosyltransferase activity; and STAT family protein binding activity. Involved in several processes, including positive regulation of nitrogen compound metabolic process; regulation of defense response; and regulation of gene expression. |
| HERC6 | HERC6 belongs to the HERC family of ubiquitin ligases, all of which contain a HECT domain and at least 1 RCC1 (MIM 179710)-like domain (RLD). |
| KLF4 | The encoded protein is thought to control the G1-to-S transition of the cell cycle following DNA damage by mediating the tumor suppressor gene p53. |
| IFI6 | The encoded protein may play a critical role in the regulation of apoptosis. |
| CEACAM1 | The encoded protein was originally described in bile ducts of liver as biliary glycoprotein. Subsequently, it was found to be a cell-cell adhesion molecule detected on leukocytes, epithelia, and endothelia. |
| PMAIP1 | This gene belongs to a pro-apoptotic subfamily within the BCL-2 protein family, referred to as the BCL-2 homology domain 3 (BH3)-only subfamily, which determine whether a cell commits to apoptosis. |
| IFI16 | This gene encodes a member of the HIN-200 (hematopoietic interferon-inducible nuclear antigens with 200 amino acid repeats) family of cytokines. The encoded protein contains domains involved in DNA binding, transcriptional regulation, and protein-protein interactions. The protein localizes to the nucleoplasm and nucleoli, and interacts with p53 and retinoblastoma-1. It modulates p53 function, and inhibits cell growth in the Ras/Raf signaling pathway. Alternatively spliced transcript variants encoding different isoforms have been found for this gene |
| CFAP53 | This gene belongs to the CFAP53 family. It was found to be differentially expressed by the ciliated cells of frog epidermis and in skin fibroblasts from human. |
| LURAP1L | Predicted to be involved in positive regulation of I-kappaB kinase/NF-kappaB signaling. |
| APOL1 | This gene encodes a secreted high density lipoprotein which binds to apolipoprotein A-I. Apolipoprotein A-I is a relatively abundant plasma protein and is the major apoprotein of HDL. |
| IFI27 | Enables RNA polymerase II-specific DNA-binding transcription factor binding activity; identical protein binding activity; and lamin binding activity. |

**Supplementary Table 5. The GO-BP enrichment of DEGs in JEG-3 cells of 24h**

| **ID** | **Description** | **GeneRatio** | **pvalue** | **p.adjust** | **Count** |
| --- | --- | --- | --- | --- | --- |
| GO:0051607 | defense response to virus | 25/149 | 3.43E-20 | 4.32E-17 | 25 |
| GO:0140546 | defense response to symbiont | 25/149 | 3.43E-20 | 4.32E-17 | 25 |
| GO:0009615 | response to virus | 28/149 | 4.50E-20 | 4.32E-17 | 28 |
| GO:0045071 | negative regulation of viral genome replication | 13/149 | 2.30E-16 | 1.66E-13 | 13 |
| GO:0048525 | negative regulation of viral process | 14/149 | 9.10E-15 | 5.25E-12 | 14 |
| GO:0045069 | regulation of viral genome replication | 13/149 | 9.03E-14 | 4.06E-11 | 13 |
| GO:0019079 | viral genome replication | 15/149 | 9.84E-14 | 4.06E-11 | 15 |
| GO:1903900 | regulation of viral life cycle | 13/149 | 1.68E-10 | 6.06E-08 | 13 |
| GO:0060337 | type I interferon signaling pathway | 11/149 | 2.28E-10 | 6.71E-08 | 11 |
| GO:0071357 | cellular response to type I interferon | 11/149 | 2.56E-10 | 6.71E-08 | 11 |
| GO:0050792 | regulation of viral process | 14/149 | 2.56E-10 | 6.71E-08 | 14 |
| GO:0034340 | response to type I interferon | 11/149 | 4.45E-10 | 1.07E-07 | 11 |
| GO:0043903 | regulation of biological process involved in symbiotic interaction | 14/149 | 5.47E-10 | 1.21E-07 | 14 |
| GO:0019058 | viral life cycle | 15/149 | 1.13E-07 | 2.33E-05 | 15 |
| GO:0002697 | regulation of immune effector process | 16/149 | 8.72E-07 | 0.00016767 | 16 |
| GO:0050688 | regulation of defense response to virus | 7/149 | 1.19E-06 | 0.00021514 | 7 |
| GO:0032480 | negative regulation of type I interferon production | 6/149 | 1.81E-06 | 0.00030635 | 6 |
| GO:0001819 | positive regulation of cytokine production | 15/149 | 2.01E-06 | 0.00032277 | 15 |
| GO:0048660 | regulation of smooth muscle cell proliferation | 9/149 | 5.14E-06 | 0.00078044 | 9 |
| GO:0048659 | smooth muscle cell proliferation | 9/149 | 5.69E-06 | 0.00082048 | 9 |
| GO:0048661 | positive regulation of smooth muscle cell proliferation | 7/149 | 1.10E-05 | 0.00145698 | 7 |
| GO:0032496 | response to lipopolysaccharide | 12/149 | 1.11E-05 | 0.00145698 | 12 |
| GO:1903037 | regulation of leukocyte cell-cell adhesion | 12/149 | 1.26E-05 | 0.00157432 | 12 |
| GO:0002544 | chronic inflammatory response | 4/149 | 1.32E-05 | 0.00158832 | 4 |
| GO:0002237 | response to molecule of bacterial origin | 12/149 | 2.01E-05 | 0.00212306 | 12 |
| GO:0072574 | hepatocyte proliferation | 4/149 | 2.02E-05 | 0.00212306 | 4 |
| GO:0072575 | epithelial cell proliferation involved in liver morphogenesis | 4/149 | 2.02E-05 | 0.00212306 | 4 |
| GO:0098586 | cellular response to virus | 6/149 | 2.06E-05 | 0.00212306 | 6 |
| GO:0072576 | liver morphogenesis | 4/149 | 2.45E-05 | 0.00243541 | 4 |
| GO:0071222 | cellular response to lipopolysaccharide | 9/149 | 2.73E-05 | 0.00262299 | 9 |
| GO:0006953 | acute-phase response | 5/149 | 3.04E-05 | 0.002825 | 5 |
| GO:0007159 | leukocyte cell-cell adhesion | 12/149 | 3.48E-05 | 0.00312518 | 12 |
| GO:0001818 | negative regulation of cytokine production | 12/149 | 3.58E-05 | 0.00312518 | 12 |
| GO:0071219 | cellular response to molecule of bacterial origin | 9/149 | 4.33E-05 | 0.00367699 | 9 |
| GO:0039530 | MDA-5 signaling pathway | 3/149 | 5.57E-05 | 0.00458665 | 3 |
| GO:0043331 | response to dsRNA | 5/149 | 6.66E-05 | 0.00533472 | 5 |
| GO:0033002 | muscle cell proliferation | 9/149 | 6.91E-05 | 0.00538543 | 9 |
| GO:0032479 | regulation of type I interferon production | 7/149 | 7.10E-05 | 0.00538543 | 7 |
| GO:0032606 | type I interferon production | 7/149 | 7.45E-05 | 0.00551216 | 7 |
| GO:0071216 | cellular response to biotic stimulus | 9/149 | 0.000100072 | 0.00709539 | 9 |
| GO:0032352 | positive regulation of hormone metabolic process | 3/149 | 0.000100871 | 0.00709539 | 3 |
| GO:1903039 | positive regulation of leukocyte cell-cell adhesion | 9/149 | 0.000103384 | 0.00709902 | 9 |
| GO:0039528 | cytoplasmic pattern recognition receptor signaling pathway in response to virus | 4/149 | 0.000113218 | 0.00759348 | 4 |
| GO:0002831 | regulation of response to biotic stimulus | 12/149 | 0.000129271 | 0.00839256 | 12 |
| GO:0060759 | regulation of response to cytokine stimulus | 8/149 | 0.000130952 | 0.00839256 | 8 |
| GO:0042102 | positive regulation of T cell proliferation | 6/149 | 0.000134541 | 0.00843513 | 6 |
| GO:0022407 | regulation of cell-cell adhesion | 12/149 | 0.000186771 | 0.01146061 | 12 |
| GO:2000345 | regulation of hepatocyte proliferation | 3/149 | 0.000205019 | 0.01212852 | 3 |
| GO:0002526 | acute inflammatory response | 6/149 | 0.000206067 | 0.01212852 | 6 |
| GO:0042531 | positive regulation of tyrosine phosphorylation of STAT protein | 5/149 | 0.000215203 | 0.01241289 | 5 |
| GO:0022612 | gland morphogenesis | 6/149 | 0.000251595 | 0.01397663 | 6 |
| GO:0002221 | pattern recognition receptor signaling pathway | 8/149 | 0.000252006 | 0.01397663 | 8 |
| GO:0032675 | regulation of interleukin-6 production | 7/149 | 0.00026288 | 0.01404002 | 7 |
| GO:0002753 | cytoplasmic pattern recognition receptor signaling pathway | 5/149 | 0.000262885 | 0.01404002 | 5 |
| GO:1902895 | positive regulation of pri-miRNA transcription by RNA polymerase II | 4/149 | 0.000273971 | 0.01436604 | 4 |
| GO:0050691 | regulation of defense response to virus by host | 4/149 | 0.000301737 | 0.01548763 | 4 |
| GO:0032635 | interleukin-6 production | 7/149 | 0.000306101 | 0.01548763 | 7 |
| GO:0045765 | regulation of angiogenesis | 10/149 | 0.000337168 | 0.01650167 | 10 |
| GO:0042445 | hormone metabolic process | 8/149 | 0.000345637 | 0.01650167 | 8 |
| GO:0022409 | positive regulation of cell-cell adhesion | 9/149 | 0.000353761 | 0.01650167 | 9 |
| GO:0002699 | positive regulation of immune effector process | 8/149 | 0.000356381 | 0.01650167 | 8 |
| GO:0019216 | regulation of lipid metabolic process | 11/149 | 0.000358055 | 0.01650167 | 11 |
| GO:0071360 | cellular response to exogenous dsRNA | 3/149 | 0.000361343 | 0.01650167 | 3 |
| GO:0032652 | regulation of interleukin-1 production | 6/149 | 0.000366195 | 0.01650167 | 6 |
| GO:0045444 | fat cell differentiation | 8/149 | 0.000378687 | 0.01657616 | 8 |
| GO:0006805 | xenobiotic metabolic process | 6/149 | 0.000382992 | 0.01657616 | 6 |
| GO:1901342 | regulation of vasculature development | 10/149 | 0.000387772 | 0.01657616 | 10 |
| GO:0042110 | T cell activation | 12/149 | 0.000390839 | 0.01657616 | 12 |
| GO:0046427 | positive regulation of receptor signaling pathway via JAK-STAT | 4/149 | 0.000397118 | 0.01659837 | 4 |
| GO:0001659 | temperature homeostasis | 7/149 | 0.000424348 | 0.01748314 | 7 |
| GO:0071466 | cellular response to xenobiotic stimulus | 6/149 | 0.000476219 | 0.01934389 | 6 |
| GO:0032612 | interleukin-1 production | 6/149 | 0.000496819 | 0.01990037 | 6 |
| GO:1904707 | positive regulation of vascular associated smooth muscle cell proliferation | 4/149 | 0.000512295 | 0.02023916 | 4 |
| GO:0002718 | regulation of cytokine production involved in immune response | 5/149 | 0.000537715 | 0.02095637 | 5 |
| GO:0043330 | response to exogenous dsRNA | 4/149 | 0.000555462 | 0.02099144 | 4 |
| GO:1904894 | positive regulation of receptor signaling pathway via STAT | 4/149 | 0.000555462 | 0.02099144 | 4 |
| GO:0019915 | lipid storage | 5/149 | 0.00056773 | 0.02099144 | 5 |
| GO:0042509 | regulation of tyrosine phosphorylation of STAT protein | 5/149 | 0.00056773 | 0.02099144 | 5 |
| GO:0032755 | positive regulation of interleukin-6 production | 5/149 | 0.000598984 | 0.02186672 | 5 |
| GO:0009410 | response to xenobiotic stimulus | 6/149 | 0.00061045 | 0.02200673 | 6 |
| GO:0007260 | tyrosine phosphorylation of STAT protein | 5/149 | 0.000665347 | 0.02303372 | 5 |
| GO:0010888 | negative regulation of lipid storage | 3/149 | 0.000666336 | 0.02303372 | 3 |
| GO:0071359 | cellular response to dsRNA | 3/149 | 0.000666336 | 0.02303372 | 3 |
| GO:0070663 | regulation of leukocyte proliferation | 8/149 | 0.000670885 | 0.02303372 | 8 |
| GO:1902893 | regulation of pri-miRNA transcription by RNA polymerase II | 4/149 | 0.000700429 | 0.02349186 | 4 |
| GO:0042058 | regulation of epidermal growth factor receptor signaling pathway | 5/149 | 0.00070052 | 0.02349186 | 5 |
| GO:0050671 | positive regulation of lymphocyte proliferation | 6/149 | 0.000715088 | 0.02370474 | 6 |
| GO:0032946 | positive regulation of mononuclear cell proliferation | 6/149 | 0.000743293 | 0.02435975 | 6 |
| GO:0061614 | pri-miRNA transcription by RNA polymerase II | 4/149 | 0.00075418 | 0.02441361 | 4 |
| GO:0097066 | response to thyroid hormone | 3/149 | 0.000761867 | 0.02441361 | 3 |
| GO:0002720 | positive regulation of cytokine production involved in immune response | 4/149 | 0.000810782 | 0.02532978 | 4 |
| GO:0051702 | biological process involved in interaction with symbiont | 5/149 | 0.000814412 | 0.02532978 | 5 |
| GO:0051090 | regulation of DNA-binding transcription factor activity | 11/149 | 0.000816806 | 0.02532978 | 11 |
| GO:0070661 | leukocyte proliferation | 9/149 | 0.000852573 | 0.02596448 | 9 |
| GO:0002367 | cytokine production involved in immune response | 5/149 | 0.000855279 | 0.02596448 | 5 |
| GO:0050731 | positive regulation of peptidyl-tyrosine phosphorylation | 7/149 | 0.000869964 | 0.02613517 | 7 |
| GO:0062013 | positive regulation of small molecule metabolic process | 6/149 | 0.000897468 | 0.02641674 | 6 |
| GO:1904035 | regulation of epithelial cell apoptotic process | 5/149 | 0.000897656 | 0.02641674 | 5 |
| GO:0042098 | T cell proliferation | 7/149 | 0.000924003 | 0.02691742 | 7 |
| GO:0039529 | RIG-I signaling pathway | 3/149 | 0.000978064 | 0.0279585 | 3 |
| GO:1901184 | regulation of ERBB signaling pathway | 5/149 | 0.000987076 | 0.0279585 | 5 |
| GO:0010883 | regulation of lipid storage | 4/149 | 0.000998518 | 0.0279585 | 4 |
| GO:0032731 | positive regulation of interleukin-1 beta production | 4/149 | 0.000998518 | 0.0279585 | 4 |
| GO:0032677 | regulation of interleukin-8 production | 5/149 | 0.001034189 | 0.02867886 | 5 |
| GO:0032757 | positive regulation of interleukin-8 production | 4/149 | 0.001067349 | 0.02925336 | 4 |
| GO:0002700 | regulation of production of molecular mediator of immune response | 6/149 | 0.001075193 | 0.02925336 | 6 |
| GO:0042403 | thyroid hormone metabolic process | 3/149 | 0.001099268 | 0.02962886 | 3 |
| GO:0070665 | positive regulation of leukocyte proliferation | 6/149 | 0.001153392 | 0.03079983 | 6 |
| GO:0046890 | regulation of lipid biosynthetic process | 7/149 | 0.001167183 | 0.03088217 | 7 |
| GO:0050863 | regulation of T cell activation | 9/149 | 0.001184112 | 0.03104527 | 9 |
| GO:0032663 | regulation of interleukin-2 production | 4/149 | 0.001214885 | 0.03126557 | 4 |
| GO:0002675 | positive regulation of acute inflammatory response | 3/149 | 0.001229554 | 0.03126557 | 3 |
| GO:0008202 | steroid metabolic process | 9/149 | 0.001235271 | 0.03126557 | 9 |
| GO:0045834 | positive regulation of lipid metabolic process | 6/149 | 0.001235879 | 0.03126557 | 6 |
| GO:0032637 | interleukin-8 production | 5/149 | 0.001352755 | 0.0336323 | 5 |
| GO:0032651 | regulation of interleukin-1 beta production | 5/149 | 0.001352755 | 0.0336323 | 5 |
| GO:0010575 | positive regulation of vascular endothelial growth factor production | 3/149 | 0.001369173 | 0.03363367 | 3 |
| GO:0032623 | interleukin-2 production | 4/149 | 0.001376135 | 0.03363367 | 4 |
| GO:0010001 | glial cell differentiation | 7/149 | 0.001418821 | 0.03438554 | 7 |
| GO:0002822 | regulation of adaptive immune response based on somatic recombination of immune receptors built from immunoglobulin superfamily domains | 6/149 | 0.001461876 | 0.03456328 | 6 |
| GO:0010573 | vascular endothelial growth factor production | 4/149 | 0.001462108 | 0.03456328 | 4 |
| GO:0051851 | modulation by host of symbiont process | 4/149 | 0.001462108 | 0.03456328 | 4 |
| GO:0050870 | positive regulation of T cell activation | 7/149 | 0.00149798 | 0.03512336 | 7 |
| GO:0045940 | positive regulation of steroid metabolic process | 3/149 | 0.001518366 | 0.03527154 | 3 |
| GO:0030522 | intracellular receptor signaling pathway | 8/149 | 0.00152876 | 0.03527154 | 8 |
| GO:0032732 | positive regulation of interleukin-1 production | 4/149 | 0.001645161 | 0.03750022 | 4 |
| GO:0032611 | interleukin-1 beta production | 5/149 | 0.001669205 | 0.03750022 | 5 |
| GO:0002828 | regulation of type 2 immune response | 3/149 | 0.001677368 | 0.03750022 | 3 |
| GO:0045948 | positive regulation of translational initiation | 3/149 | 0.001677368 | 0.03750022 | 3 |
| GO:0032680 | regulation of tumor necrosis factor production | 6/149 | 0.001718106 | 0.03811552 | 6 |
| GO:0045824 | negative regulation of innate immune response | 4/149 | 0.001742403 | 0.03835946 | 4 |
| GO:0032640 | tumor necrosis factor production | 6/149 | 0.001829629 | 0.03971068 | 6 |
| GO:0046651 | lymphocyte proliferation | 8/149 | 0.001831318 | 0.03971068 | 8 |
| GO:0002230 | positive regulation of defense response to virus by host | 3/149 | 0.001846406 | 0.03973907 | 3 |
| GO:0050670 | regulation of lymphocyte proliferation | 7/149 | 0.001897798 | 0.0405426 | 7 |
| GO:1903555 | regulation of tumor necrosis factor superfamily cytokine production | 6/149 | 0.001946559 | 0.04118154 | 6 |
| GO:0032943 | mononuclear cell proliferation | 8/149 | 0.001956266 | 0.04118154 | 8 |
| GO:0032944 | regulation of mononuclear cell proliferation | 7/149 | 0.001996943 | 0.04172945 | 7 |
| GO:1901099 | negative regulation of signal transduction in absence of ligand | 3/149 | 0.002025702 | 0.04172945 | 3 |
| GO:2001240 | negative regulation of extrinsic apoptotic signaling pathway in absence of ligand | 3/149 | 0.002025702 | 0.04172945 | 3 |
| GO:0045785 | positive regulation of cell adhesion | 10/149 | 0.002056283 | 0.04202238 | 10 |
| GO:0007259 | receptor signaling pathway via JAK-STAT | 6/149 | 0.002069063 | 0.04202238 | 6 |
| GO:0071706 | tumor necrosis factor superfamily cytokine production | 6/149 | 0.002132458 | 0.04300706 | 6 |
| GO:0051098 | regulation of binding | 9/149 | 0.002158899 | 0.0430437 | 9 |
| GO:0050673 | epithelial cell proliferation | 10/149 | 0.002164125 | 0.0430437 | 10 |
| GO:0042129 | regulation of T cell proliferation | 6/149 | 0.00219731 | 0.04317167 | 6 |
| GO:0032350 | regulation of hormone metabolic process | 3/149 | 0.002215467 | 0.04317167 | 3 |
| GO:0032728 | positive regulation of interferon-beta production | 3/149 | 0.002215467 | 0.04317167 | 3 |
| GO:1904019 | epithelial cell apoptotic process | 5/149 | 0.002284085 | 0.04421007 | 5 |
| GO:0002819 | regulation of adaptive immune response | 6/149 | 0.002331469 | 0.04482637 | 6 |
| GO:0120254 | olefinic compound metabolic process | 5/149 | 0.002552416 | 0.04874946 | 5 |
| GO:0001660 | fever generation | 2/149 | 0.002675757 | 0.04948461 | 2 |
| GO:0003093 | regulation of glomerular filtration | 2/149 | 0.002675757 | 0.04948461 | 2 |
| GO:0042135 | neurotransmitter catabolic process | 2/149 | 0.002675757 | 0.04948461 | 2 |
| GO:0071313 | cellular response to caffeine | 2/149 | 0.002675757 | 0.04948461 | 2 |
| GO:0048771 | tissue remodeling | 6/149 | 0.002693857 | 0.04948461 | 6 |
| GO:0097696 | receptor signaling pathway via STAT | 6/149 | 0.002693857 | 0.04948461 | 6 |

**Supplementary Table 6. The GO-BP enrichment of up-regulated DEGs in JEG-3 cells of 24h**

| ID | Description | GeneRatio | pvalue | p.adjust | Count |
| --- | --- | --- | --- | --- | --- |
| GO:0051607 | defense response to virus | 25/122 | 1.93E-22 | 2.59E-19 | 25 |
| GO:0140546 | defense response to symbiont | 25/122 | 1.93E-22 | 2.59E-19 | 25 |
| GO:0009615 | response to virus | 27/122 | 2.26E-21 | 2.03E-18 | 27 |
| GO:0045071 | negative regulation of viral genome replication | 13/122 | 1.60E-17 | 1.07E-14 | 13 |
| GO:0048525 | negative regulation of viral process | 14/122 | 5.30E-16 | 2.85E-13 | 14 |
| GO:0019079 | viral genome replication | 15/122 | 4.84E-15 | 2.17E-12 | 15 |
| GO:0045069 | regulation of viral genome replication | 13/122 | 6.51E-15 | 2.50E-12 | 13 |
| GO:1903900 | regulation of viral life cycle | 13/122 | 1.32E-11 | 4.44E-09 | 13 |
| GO:0050792 | regulation of viral process | 14/122 | 1.70E-11 | 5.07E-09 | 14 |
| GO:0060337 | type I interferon signaling pathway | 11/122 | 2.59E-11 | 6.95E-09 | 11 |
| GO:0071357 | cellular response to type I interferon | 11/122 | 2.91E-11 | 7.10E-09 | 11 |
| GO:0043903 | regulation of biological process involved in symbiotic interaction | 14/122 | 3.68E-11 | 8.24E-09 | 14 |
| GO:0034340 | response to type I interferon | 11/122 | 5.10E-11 | 1.05E-08 | 11 |
| GO:0019058 | viral life cycle | 15/122 | 7.47E-09 | 1.43E-06 | 15 |
| GO:0050688 | regulation of defense response to virus | 7/122 | 3.08E-07 | 5.52E-05 | 7 |
| GO:0002697 | regulation of immune effector process | 15/122 | 3.32E-07 | 5.58E-05 | 15 |
| GO:0032480 | negative regulation of type I interferon production | 6/122 | 5.59E-07 | 8.84E-05 | 6 |
| GO:0001819 | positive regulation of cytokine production | 14/122 | 9.15E-07 | 0.000137 | 14 |
| GO:0048660 | regulation of smooth muscle cell proliferation | 9/122 | 9.77E-07 | 0.000138 | 9 |
| GO:0048659 | smooth muscle cell proliferation | 9/122 | 1.08E-06 | 0.000146 | 9 |
| GO:0048661 | positive regulation of smooth muscle cell proliferation | 7/122 | 2.95E-06 | 0.000377 | 7 |
| GO:0001818 | negative regulation of cytokine production | 12/122 | 4.68E-06 | 0.000571 | 12 |
| GO:0071222 | cellular response to lipopolysaccharide | 9/122 | 5.44E-06 | 0.000635 | 9 |
| GO:0002544 | chronic inflammatory response | 4/122 | 5.99E-06 | 0.00067 | 4 |
| GO:0098586 | cellular response to virus | 6/122 | 6.57E-06 | 0.000706 | 6 |
| GO:0071219 | cellular response to molecule of bacterial origin | 9/122 | 8.77E-06 | 0.000881 | 9 |
| GO:0032496 | response to lipopolysaccharide | 11/122 | 8.85E-06 | 0.000881 | 11 |
| GO:1903037 | regulation of leukocyte cell-cell adhesion | 11/122 | 9.93E-06 | 0.000952 | 11 |
| GO:0006953 | acute-phase response | 5/122 | 1.16E-05 | 0.001071 | 5 |
| GO:0033002 | muscle cell proliferation | 9/122 | 1.42E-05 | 0.001273 | 9 |
| GO:0002237 | response to molecule of bacterial origin | 11/122 | 1.54E-05 | 0.001338 | 11 |
| GO:0032479 | regulation of type I interferon production | 7/122 | 1.97E-05 | 0.001649 | 7 |
| GO:0032606 | type I interferon production | 7/122 | 2.07E-05 | 0.001649 | 7 |
| GO:0071216 | cellular response to biotic stimulus | 9/122 | 2.09E-05 | 0.001649 | 9 |
| GO:0043331 | response to dsRNA | 5/122 | 2.56E-05 | 0.001935 | 5 |
| GO:0007159 | leukocyte cell-cell adhesion | 11/122 | 2.59E-05 | 0.001935 | 11 |
| GO:0039530 | MDA-5 signaling pathway | 3/122 | 3.06E-05 | 0.002225 | 3 |
| GO:0060759 | regulation of response to cytokine stimulus | 8/122 | 3.19E-05 | 0.002252 | 8 |
| GO:0039528 | cytoplasmic pattern recognition receptor signaling pathway in response to virus | 4/122 | 5.21E-05 | 0.003586 | 4 |
| GO:0002221 | pattern recognition receptor signaling pathway | 8/122 | 6.28E-05 | 0.004217 | 8 |
| GO:0045765 | regulation of angiogenesis | 10/122 | 6.48E-05 | 0.004248 | 10 |
| GO:0002526 | acute inflammatory response | 6/122 | 6.87E-05 | 0.004391 | 6 |
| GO:1901342 | regulation of vasculature development | 10/122 | 7.51E-05 | 0.004627 | 10 |
| GO:0032675 | regulation of interleukin-6 production | 7/122 | 7.58E-05 | 0.004627 | 7 |
| GO:0042531 | positive regulation of tyrosine phosphorylation of STAT protein | 5/122 | 8.42E-05 | 0.005024 | 5 |
| GO:0032635 | interleukin-6 production | 7/122 | 8.87E-05 | 0.005038 | 7 |
| GO:0002831 | regulation of response to biotic stimulus | 11/122 | 8.97E-05 | 0.005038 | 11 |
| GO:0002699 | positive regulation of immune effector process | 8/122 | 9.00E-05 | 0.005038 | 8 |
| GO:0045444 | fat cell differentiation | 8/122 | 9.59E-05 | 0.005257 | 8 |
| GO:0002753 | cytoplasmic pattern recognition receptor signaling pathway | 5/122 | 0.000103 | 0.005543 | 5 |
| GO:0032652 | regulation of interleukin-1 production | 6/122 | 0.000124 | 0.006329 | 6 |
| GO:0001659 | temperature homeostasis | 7/122 | 0.000124 | 0.006329 | 7 |
| GO:1902895 | positive regulation of pri-miRNA transcription by RNA polymerase II | 4/122 | 0.000127 | 0.006329 | 4 |
| GO:0022407 | regulation of cell-cell adhesion | 11/122 | 0.000127 | 0.006329 | 11 |
| GO:0050691 | regulation of defense response to virus by host | 4/122 | 0.00014 | 0.006827 | 4 |
| GO:1903039 | positive regulation of leukocyte cell-cell adhesion | 8/122 | 0.000142 | 0.006827 | 8 |
| GO:0032612 | interleukin-1 production | 6/122 | 0.000169 | 0.00798 | 6 |
| GO:0046427 | positive regulation of receptor signaling pathway via JAK-STAT | 4/122 | 0.000185 | 0.008574 | 4 |
| GO:0071360 | cellular response to exogenous dsRNA | 3/122 | 0.000201 | 0.009136 | 3 |
| GO:0002718 | regulation of cytokine production involved in immune response | 5/122 | 0.000214 | 0.009582 | 5 |
| GO:0019915 | lipid storage | 5/122 | 0.000226 | 0.009802 | 5 |
| GO:0042509 | regulation of tyrosine phosphorylation of STAT protein | 5/122 | 0.000226 | 0.009802 | 5 |
| GO:0032755 | positive regulation of interleukin-6 production | 5/122 | 0.000239 | 0.010058 | 5 |
| GO:1904707 | positive regulation of vascular associated smooth muscle cell proliferation | 4/122 | 0.00024 | 0.010058 | 4 |
| GO:0042110 | T cell activation | 11/122 | 0.000257 | 0.010429 | 11 |
| GO:0043330 | response to exogenous dsRNA | 4/122 | 0.00026 | 0.010429 | 4 |
| GO:1904894 | positive regulation of receptor signaling pathway via STAT | 4/122 | 0.00026 | 0.010429 | 4 |
| GO:0007260 | tyrosine phosphorylation of STAT protein | 5/122 | 0.000266 | 0.010511 | 5 |
| GO:0042058 | regulation of epidermal growth factor receptor signaling pathway | 5/122 | 0.00028 | 0.010919 | 5 |
| GO:0019216 | regulation of lipid metabolic process | 10/122 | 0.000286 | 0.010972 | 10 |
| GO:0062013 | positive regulation of small molecule metabolic process | 6/122 | 0.000311 | 0.011783 | 6 |
| GO:0072574 | hepatocyte proliferation | 3/122 | 0.000322 | 0.011865 | 3 |
| GO:0072575 | epithelial cell proliferation involved in liver morphogenesis | 3/122 | 0.000322 | 0.011865 | 3 |
| GO:1902893 | regulation of pri-miRNA transcription by RNA polymerase II | 4/122 | 0.000329 | 0.011947 | 4 |
| GO:0002367 | cytokine production involved in immune response | 5/122 | 0.000344 | 0.012322 | 5 |
| GO:0061614 | pri-miRNA transcription by RNA polymerase II | 4/122 | 0.000355 | 0.012323 | 4 |
| GO:0046890 | regulation of lipid biosynthetic process | 7/122 | 0.000356 | 0.012323 | 7 |
| GO:1904035 | regulation of epithelial cell apoptotic process | 5/122 | 0.000362 | 0.012323 | 5 |
| GO:0010888 | negative regulation of lipid storage | 3/122 | 0.000372 | 0.012323 | 3 |
| GO:0071359 | cellular response to dsRNA | 3/122 | 0.000372 | 0.012323 | 3 |
| GO:0072576 | liver morphogenesis | 3/122 | 0.000372 | 0.012323 | 3 |
| GO:0002720 | positive regulation of cytokine production involved in immune response | 4/122 | 0.000382 | 0.012508 | 4 |
| GO:1901184 | regulation of ERBB signaling pathway | 5/122 | 0.000398 | 0.012895 | 5 |
| GO:0030522 | intracellular receptor signaling pathway | 8/122 | 0.000413 | 0.013208 | 8 |
| GO:0032677 | regulation of interleukin-8 production | 5/122 | 0.000418 | 0.013208 | 5 |
| GO:0045834 | positive regulation of lipid metabolic process | 6/122 | 0.000434 | 0.013304 | 6 |
| GO:0022409 | positive regulation of cell-cell adhesion | 8/122 | 0.000434 | 0.013304 | 8 |
| GO:0010001 | glial cell differentiation | 7/122 | 0.000436 | 0.013304 | 7 |
| GO:0042102 | positive regulation of T cell proliferation | 5/122 | 0.000459 | 0.013857 | 5 |
| GO:0010883 | regulation of lipid storage | 4/122 | 0.000472 | 0.013928 | 4 |
| GO:0032731 | positive regulation of interleukin-1 beta production | 4/122 | 0.000472 | 0.013928 | 4 |
| GO:0032757 | positive regulation of interleukin-8 production | 4/122 | 0.000505 | 0.014743 | 4 |
| GO:0002822 | regulation of adaptive immune response based on somatic recombination of immune receptors built from immunoglobulin superfamily domains | 6/122 | 0.000516 | 0.0149 | 6 |
| GO:0039529 | RIG-I signaling pathway | 3/122 | 0.000547 | 0.015404 | 3 |
| GO:0032637 | interleukin-8 production | 5/122 | 0.000551 | 0.015404 | 5 |
| GO:0032651 | regulation of interleukin-1 beta production | 5/122 | 0.000551 | 0.015404 | 5 |
| GO:0032680 | regulation of tumor necrosis factor production | 6/122 | 0.00061 | 0.01689 | 6 |
| GO:0051090 | regulation of DNA-binding transcription factor activity | 10/122 | 0.000622 | 0.017056 | 10 |
| GO:0032640 | tumor necrosis factor production | 6/122 | 0.000651 | 0.017664 | 6 |
| GO:0032611 | interleukin-1 beta production | 5/122 | 0.000683 | 0.018141 | 5 |
| GO:0002675 | positive regulation of acute inflammatory response | 3/122 | 0.000689 | 0.018141 | 3 |
| GO:1903555 | regulation of tumor necrosis factor superfamily cytokine production | 6/122 | 0.000694 | 0.018141 | 6 |
| GO:0010573 | vascular endothelial growth factor production | 4/122 | 0.000696 | 0.018141 | 4 |
| GO:0007259 | receptor signaling pathway via JAK-STAT | 6/122 | 0.00074 | 0.019106 | 6 |
| GO:0071706 | tumor necrosis factor superfamily cytokine production | 6/122 | 0.000763 | 0.019421 | 6 |
| GO:0010575 | positive regulation of vascular endothelial growth factor production | 3/122 | 0.000769 | 0.019421 | 3 |
| GO:0022612 | gland morphogenesis | 5/122 | 0.000774 | 0.019421 | 5 |
| GO:0032732 | positive regulation of interleukin-1 production | 4/122 | 0.000784 | 0.01951 | 4 |
| GO:0045824 | negative regulation of innate immune response | 4/122 | 0.000832 | 0.020452 | 4 |
| GO:0002819 | regulation of adaptive immune response | 6/122 | 0.000838 | 0.020452 | 6 |
| GO:0045940 | positive regulation of steroid metabolic process | 3/122 | 0.000853 | 0.020645 | 3 |
| GO:0002828 | regulation of type 2 immune response | 3/122 | 0.000943 | 0.022235 | 3 |
| GO:0045948 | positive regulation of translational initiation | 3/122 | 0.000943 | 0.022235 | 3 |
| GO:1904019 | epithelial cell apoptotic process | 5/122 | 0.000944 | 0.022235 | 5 |
| GO:0048771 | tissue remodeling | 6/122 | 0.000974 | 0.022542 | 6 |
| GO:0097696 | receptor signaling pathway via STAT | 6/122 | 0.000974 | 0.022542 | 6 |
| GO:0002230 | positive regulation of defense response to virus by host | 3/122 | 0.00104 | 0.023868 | 3 |
| GO:0032102 | negative regulation of response to external stimulus | 9/122 | 0.001058 | 0.024077 | 9 |
| GO:1901099 | negative regulation of signal transduction in absence of ligand | 3/122 | 0.001142 | 0.025558 | 3 |
| GO:2001240 | negative regulation of extrinsic apoptotic signaling pathway in absence of ligand | 3/122 | 0.001142 | 0.025558 | 3 |
| GO:0007173 | epidermal growth factor receptor signaling pathway | 5/122 | 0.001227 | 0.027229 | 5 |
| GO:0032728 | positive regulation of interferon-beta production | 3/122 | 0.00125 | 0.027523 | 3 |
| GO:0010565 | regulation of cellular ketone metabolic process | 6/122 | 0.001296 | 0.028239 | 6 |
| GO:0050863 | regulation of T cell activation | 8/122 | 0.001304 | 0.028239 | 8 |
| GO:0002705 | positive regulation of leukocyte mediated immunity | 5/122 | 0.001318 | 0.028318 | 5 |
| GO:0032481 | positive regulation of type I interferon production | 4/122 | 0.001567 | 0.033394 | 4 |
| GO:0050731 | positive regulation of peptidyl-tyrosine phosphorylation | 6/122 | 0.001608 | 0.033849 | 6 |
| GO:0042092 | type 2 immune response | 3/122 | 0.001613 | 0.033849 | 3 |
| GO:0042098 | T cell proliferation | 6/122 | 0.001694 | 0.035272 | 6 |
| GO:0031397 | negative regulation of protein ubiquitination | 4/122 | 0.001722 | 0.035387 | 4 |
| GO:0045727 | positive regulation of translation | 5/122 | 0.001734 | 0.035387 | 5 |
| GO:1904994 | regulation of leukocyte adhesion to vascular endothelial cell | 3/122 | 0.001747 | 0.035387 | 3 |
| GO:0045785 | positive regulation of cell adhesion | 9/122 | 0.001783 | 0.035387 | 9 |
| GO:2000117 | negative regulation of cysteine-type endopeptidase activity | 4/122 | 0.001804 | 0.035387 | 4 |
| GO:0001660 | fever generation | 2/122 | 0.001805 | 0.035387 | 2 |
| GO:0003093 | regulation of glomerular filtration | 2/122 | 0.001805 | 0.035387 | 2 |
| GO:0071313 | cellular response to caffeine | 2/122 | 0.001805 | 0.035387 | 2 |
| GO:0042542 | response to hydrogen peroxide | 5/122 | 0.001851 | 0.03577 | 5 |
| GO:0050671 | positive regulation of lymphocyte proliferation | 5/122 | 0.001851 | 0.03577 | 5 |
| GO:0050673 | epithelial cell proliferation | 9/122 | 0.00187 | 0.035878 | 9 |
| GO:0045923 | positive regulation of fatty acid metabolic process | 3/122 | 0.001888 | 0.035966 | 3 |
| GO:0032946 | positive regulation of mononuclear cell proliferation | 5/122 | 0.001912 | 0.036166 | 5 |
| GO:1904705 | regulation of vascular associated smooth muscle cell proliferation | 4/122 | 0.002065 | 0.038516 | 4 |
| GO:1990874 | vascular associated smooth muscle cell proliferation | 4/122 | 0.002065 | 0.038516 | 4 |
| GO:0035457 | cellular response to interferon-alpha | 2/122 | 0.002197 | 0.040139 | 2 |
| GO:0051798 | positive regulation of hair follicle development | 2/122 | 0.002197 | 0.040139 | 2 |
| GO:2000551 | regulation of T-helper 2 cell cytokine production | 2/122 | 0.002197 | 0.040139 | 2 |
| GO:0051098 | regulation of binding | 8/122 | 0.002259 | 0.040994 | 8 |
| GO:0034103 | regulation of tissue remodeling | 4/122 | 0.002351 | 0.042123 | 4 |
| GO:0010907 | positive regulation of glucose metabolic process | 3/122 | 0.002352 | 0.042123 | 3 |
| GO:0002703 | regulation of leukocyte mediated immunity | 6/122 | 0.0024 | 0.042692 | 6 |
| GO:0032760 | positive regulation of tumor necrosis factor production | 4/122 | 0.002452 | 0.04311 | 4 |
| GO:0042063 | gliogenesis | 7/122 | 0.002456 | 0.04311 | 7 |
| GO:0014002 | astrocyte development | 3/122 | 0.002521 | 0.043243 | 3 |
| GO:0062208 | positive regulation of pattern recognition receptor signaling pathway | 3/122 | 0.002521 | 0.043243 | 3 |
| GO:2001239 | regulation of extrinsic apoptotic signaling pathway in absence of ligand | 3/122 | 0.002521 | 0.043243 | 3 |
| GO:0038127 | ERBB signaling pathway | 5/122 | 0.002528 | 0.043243 | 5 |
| GO:0050870 | positive regulation of T cell activation | 6/122 | 0.002576 | 0.043792 | 6 |
| GO:0002700 | regulation of production of molecular mediator of immune response | 5/122 | 0.002604 | 0.043792 | 5 |
| GO:0002863 | positive regulation of inflammatory response to antigenic stimulus | 2/122 | 0.002625 | 0.043792 | 2 |
| GO:0032352 | positive regulation of hormone metabolic process | 2/122 | 0.002625 | 0.043792 | 2 |
| GO:0032642 | regulation of chemokine production | 4/122 | 0.002663 | 0.04416 | 4 |
| GO:0070665 | positive regulation of leukocyte proliferation | 5/122 | 0.002762 | 0.04515 | 5 |
| GO:0070301 | cellular response to hydrogen peroxide | 4/122 | 0.002774 | 0.04515 | 4 |
| GO:1903557 | positive regulation of tumor necrosis factor superfamily cytokine production | 4/122 | 0.002774 | 0.04515 | 4 |
| GO:0050810 | regulation of steroid biosynthetic process | 4/122 | 0.002887 | 0.046237 | 4 |
| GO:1903321 | negative regulation of protein modification by small protein conjugation or removal | 4/122 | 0.002887 | 0.046237 | 4 |
| GO:0097191 | extrinsic apoptotic signaling pathway | 6/122 | 0.002892 | 0.046237 | 6 |
| GO:0050777 | negative regulation of immune response | 5/122 | 0.002926 | 0.046505 | 5 |
| GO:0051702 | biological process involved in interaction with symbiont | 4/122 | 0.003003 | 0.046614 | 4 |
| GO:1901216 | positive regulation of neuron death | 4/122 | 0.003003 | 0.046614 | 4 |
| GO:0050678 | regulation of epithelial cell proliferation | 8/122 | 0.003004 | 0.046614 | 8 |
| GO:0006869 | lipid transport | 9/122 | 0.003076 | 0.046614 | 9 |
| GO:0016322 | neuron remodeling | 2/122 | 0.003089 | 0.046614 | 2 |
| GO:0019336 | phenol-containing compound catabolic process | 2/122 | 0.003089 | 0.046614 | 2 |
| GO:0035745 | T-helper 2 cell cytokine production | 2/122 | 0.003089 | 0.046614 | 2 |
| GO:0061043 | regulation of vascular wound healing | 2/122 | 0.003089 | 0.046614 | 2 |
| GO:0071415 | cellular response to purine-containing compound | 2/122 | 0.003089 | 0.046614 | 2 |
| GO:0048709 | oligodendrocyte differentiation | 4/122 | 0.003123 | 0.046856 | 4 |
| GO:0051348 | negative regulation of transferase activity | 7/122 | 0.003151 | 0.047012 | 7 |
| GO:0050798 | activated T cell proliferation | 3/122 | 0.003272 | 0.048083 | 3 |
| GO:0050873 | brown fat cell differentiation | 3/122 | 0.003272 | 0.048083 | 3 |
| GO:2001236 | regulation of extrinsic apoptotic signaling pathway | 5/122 | 0.003276 | 0.048083 | 5 |
| GO:0032602 | chemokine production | 4/122 | 0.003371 | 0.049092 | 4 |
| GO:0000302 | response to reactive oxygen species | 6/122 | 0.003381 | 0.049092 | 6 |

**Supplementary Table 7. The GO-BP enrichment of down-regulated DEGs in JEG-3 cells of 24h**

| ID | Description | GeneRatio | pvalue | p.adjust | Count |
| --- | --- | --- | --- | --- | --- |
| GO:0017144 | drug metabolic process | 3/27 | 3.57E-05 | 0.020289 | 3 |
| GO:0019369 | arachidonic acid metabolic process | 3/27 | 8.06E-05 | 0.022933 | 3 |
| GO:0042738 | exogenous drug catabolic process | 2/27 | 0.00049 | 0.03827 | 2 |
| GO:0042737 | drug catabolic process | 2/27 | 0.00058 | 0.03827 | 2 |
| GO:0033559 | unsaturated fatty acid metabolic process | 3/27 | 0.00058 | 0.03827 | 3 |
| GO:0120254 | olefinic compound metabolic process | 3/27 | 0.000641 | 0.03827 | 3 |
| GO:0001676 | long-chain fatty acid metabolic process | 3/27 | 0.000657 | 0.03827 | 3 |
| GO:0006805 | xenobiotic metabolic process | 3/27 | 0.000657 | 0.03827 | 3 |
| GO:0006690 | icosanoid metabolic process | 3/27 | 0.000706 | 0.03827 | 3 |
| GO:0071280 | cellular response to copper ion | 2/27 | 0.000729 | 0.03827 | 2 |
| GO:0071466 | cellular response to xenobiotic stimulus | 3/27 | 0.00074 | 0.03827 | 3 |
| GO:0009410 | response to xenobiotic stimulus | 3/27 | 0.000848 | 0.040194 | 3 |
| GO:0042759 | long-chain fatty acid biosynthetic process | 2/27 | 0.001014 | 0.044371 | 2 |

**Supplementary Table 8. The GO-BP enrichment of DEGs in U-251 MG cells of 24h**

| ID | Description | GeneRatio | pvalue | p.adjust | Count |
| --- | --- | --- | --- | --- | --- |
| GO:0051607 | defense response to virus | 67/1080 | 3.25E-26 | 8.58E-23 | 67 |
| GO:0140546 | defense response to symbiont | 67/1080 | 3.25E-26 | 8.58E-23 | 67 |
| GO:0060337 | type I interferon signaling pathway | 40/1080 | 4.88E-25 | 8.58E-22 | 40 |
| GO:0071357 | cellular response to type I interferon | 40/1080 | 7.91E-25 | 1.04E-21 | 40 |
| GO:0009615 | response to virus | 77/1080 | 2.00E-24 | 2.11E-21 | 77 |
| GO:0034340 | response to type I interferon | 40/1080 | 7.99E-24 | 7.02E-21 | 40 |
| GO:0034341 | response to interferon-gamma | 53/1080 | 6.40E-22 | 4.82E-19 | 53 |
| GO:0048525 | negative regulation of viral process | 34/1080 | 7.41E-20 | 4.89E-17 | 34 |
| GO:0060333 | interferon-gamma-mediated signaling pathway | 34/1080 | 1.70E-19 | 9.97E-17 | 34 |
| GO:0071346 | cellular response to interferon-gamma | 45/1080 | 9.21E-18 | 4.86E-15 | 45 |
| GO:0045071 | negative regulation of viral genome replication | 25/1080 | 2.29E-17 | 1.10E-14 | 25 |
| GO:1903900 | regulation of viral life cycle | 38/1080 | 2.32E-15 | 1.02E-12 | 38 |
| GO:0050792 | regulation of viral process | 40/1080 | 2.51E-13 | 1.02E-10 | 40 |
| GO:0045069 | regulation of viral genome replication | 26/1080 | 3.91E-13 | 1.47E-10 | 26 |
| GO:0043903 | regulation of biological process involved in symbiotic interaction | 40/1080 | 1.78E-12 | 6.27E-10 | 40 |
| GO:0019885 | antigen processing and presentation of endogenous peptide antigen via MHC class I | 12/1080 | 5.54E-12 | 1.83E-09 | 12 |
| GO:0002831 | regulation of response to biotic stimulus | 62/1080 | 5.91E-12 | 1.83E-09 | 62 |
| GO:0001819 | positive regulation of cytokine production | 63/1080 | 1.13E-11 | 3.30E-09 | 63 |
| GO:0002483 | antigen processing and presentation of endogenous peptide antigen | 12/1080 | 4.06E-11 | 1.13E-08 | 12 |
| GO:0002237 | response to molecule of bacterial origin | 53/1080 | 5.15E-11 | 1.36E-08 | 53 |
| GO:0032496 | response to lipopolysaccharide | 51/1080 | 5.47E-11 | 1.37E-08 | 51 |
| GO:0034612 | response to tumor necrosis factor | 49/1080 | 2.80E-10 | 6.72E-08 | 49 |
| GO:0019079 | viral genome replication | 28/1080 | 7.67E-10 | 1.76E-07 | 28 |
| GO:0002683 | negative regulation of immune system process | 54/1080 | 4.95E-09 | 1.09E-06 | 54 |
| GO:0019883 | antigen processing and presentation of endogenous antigen | 12/1080 | 5.34E-09 | 1.13E-06 | 12 |
| GO:0071356 | cellular response to tumor necrosis factor | 44/1080 | 5.80E-09 | 1.18E-06 | 44 |
| GO:0002474 | antigen processing and presentation of peptide antigen via MHC class I | 23/1080 | 6.25E-09 | 1.22E-06 | 23 |
| GO:0045088 | regulation of innate immune response | 45/1080 | 1.36E-08 | 2.56E-06 | 45 |
| GO:0051091 | positive regulation of DNA-binding transcription factor activity | 40/1080 | 2.05E-08 | 3.72E-06 | 40 |
| GO:0046596 | regulation of viral entry into host cell | 14/1080 | 3.08E-08 | 5.41E-06 | 14 |
| GO:0002697 | regulation of immune effector process | 57/1080 | 4.64E-08 | 7.90E-06 | 57 |
| GO:0035456 | response to interferon-beta | 12/1080 | 5.98E-08 | 9.85E-06 | 12 |
| GO:0042590 | antigen processing and presentation of exogenous peptide antigen via MHC class I | 19/1080 | 8.83E-08 | 1.41E-05 | 19 |
| GO:0045824 | negative regulation of innate immune response | 17/1080 | 9.23E-08 | 1.43E-05 | 17 |
| GO:0051092 | positive regulation of NF-kappaB transcription factor activity | 28/1080 | 9.64E-08 | 1.45E-05 | 28 |
| GO:0045229 | external encapsulating structure organization | 50/1080 | 1.24E-07 | 1.82E-05 | 50 |
| GO:0051090 | regulation of DNA-binding transcription factor activity | 54/1080 | 1.36E-07 | 1.94E-05 | 54 |
| GO:0002479 | antigen processing and presentation of exogenous peptide antigen via MHC class I TAP-dependent | TAP-dependent | 1.62E-07 | 2.25E-05 | 18 |
| GO:0050727 | regulation of inflammatory response | 47/1080 | 1.79E-07 | 2.39E-05 | 47 |
| GO:0002832 | negative regulation of response to biotic stimulus | 21/1080 | 1.81E-07 | 2.39E-05 | 21 |
| GO:0042110 | T cell activation | 56/1080 | 2.12E-07 | 2.72E-05 | 56 |
| GO:0042098 | T cell proliferation | 31/1080 | 2.24E-07 | 2.82E-05 | 31 |
| GO:0030198 | extracellular matrix organization | 49/1080 | 2.44E-07 | 2.99E-05 | 49 |
| GO:0043062 | extracellular structure organization | 49/1080 | 2.64E-07 | 3.13E-05 | 49 |
| GO:0019058 | viral life cycle | 45/1080 | 2.67E-07 | 3.13E-05 | 45 |
| GO:0052372 | modulation by symbiont of entry into host | 14/1080 | 2.89E-07 | 3.31E-05 | 14 |
| GO:0007249 | I-kappaB kinase/NF-kappaB signaling | 38/1080 | 8.08E-07 | 9.07E-05 | 38 |
| GO:1901342 | regulation of vasculature development | 43/1080 | 9.74E-07 | 0.000107 | 43 |
| GO:0035455 | response to interferon-alpha | 9/1080 | 1.01E-06 | 0.000107 | 9 |
| GO:1903037 | regulation of leukocyte cell-cell adhesion | 42/1080 | 1.02E-06 | 0.000107 | 42 |
| GO:0070663 | regulation of leukocyte proliferation | 34/1080 | 1.06E-06 | 0.00011 | 34 |
| GO:0050670 | regulation of lymphocyte proliferation | 32/1080 | 1.23E-06 | 0.000125 | 32 |
| GO:0032609 | interferon-gamma production | 21/1080 | 1.32E-06 | 0.000131 | 21 |
| GO:0043122 | regulation of I-kappaB kinase/NF-kappaB signaling | 34/1080 | 1.41E-06 | 0.000138 | 34 |
| GO:0032944 | regulation of mononuclear cell proliferation | 32/1080 | 1.50E-06 | 0.000139 | 32 |
| GO:0045765 | regulation of angiogenesis | 42/1080 | 1.51E-06 | 0.000139 | 42 |
| GO:0070661 | leukocyte proliferation | 40/1080 | 1.52E-06 | 0.000139 | 40 |
| GO:0019882 | antigen processing and presentation | 33/1080 | 1.53E-06 | 0.000139 | 33 |
| GO:0031349 | positive regulation of defense response | 44/1080 | 1.84E-06 | 0.000163 | 44 |
| GO:0031348 | negative regulation of defense response | 33/1080 | 1.86E-06 | 0.000163 | 33 |
| GO:0050863 | regulation of T cell activation | 41/1080 | 2.01E-06 | 0.000173 | 41 |
| GO:0046651 | lymphocyte proliferation | 37/1080 | 2.15E-06 | 0.00018 | 37 |
| GO:0033209 | tumor necrosis factor-mediated signaling pathway | 27/1080 | 2.15E-06 | 0.00018 | 27 |
| GO:0045766 | positive regulation of angiogenesis | 27/1080 | 2.41E-06 | 0.000193 | 27 |
| GO:1904018 | positive regulation of vasculature development | 27/1080 | 2.41E-06 | 0.000193 | 27 |
| GO:0022407 | regulation of cell-cell adhesion | 50/1080 | 2.41E-06 | 0.000193 | 50 |
| GO:0007159 | leukocyte cell-cell adhesion | 44/1080 | 2.66E-06 | 0.000209 | 44 |
| GO:0032943 | mononuclear cell proliferation | 37/1080 | 2.76E-06 | 0.000214 | 37 |
| GO:0042129 | regulation of T cell proliferation | 26/1080 | 3.48E-06 | 0.000266 | 26 |
| GO:0032102 | negative regulation of response to external stimulus | 46/1080 | 3.53E-06 | 0.000266 | 46 |
| GO:1901224 | positive regulation of NIK/NF-kappaB signaling | 15/1080 | 4.40E-06 | 0.000327 | 15 |
| GO:0032680 | regulation of tumor necrosis factor production | 25/1080 | 4.47E-06 | 0.000327 | 25 |
| GO:0050777 | negative regulation of immune response | 24/1080 | 4.53E-06 | 0.000327 | 24 |
| GO:0032640 | tumor necrosis factor production | 25/1080 | 5.61E-06 | 0.0004 | 25 |
| GO:0048002 | antigen processing and presentation of peptide antigen | 28/1080 | 5.95E-06 | 0.000419 | 28 |
| GO:0050729 | positive regulation of inflammatory response | 22/1080 | 6.45E-06 | 0.000441 | 22 |
| GO:0048638 | regulation of developmental growth | 39/1080 | 6.51E-06 | 0.000441 | 39 |
| GO:0002720 | positive regulation of cytokine production involved in immune response | 13/1080 | 6.52E-06 | 0.000441 | 13 |
| GO:0001818 | negative regulation of cytokine production | 43/1080 | 6.66E-06 | 0.000445 | 43 |
| GO:1903555 | regulation of tumor necrosis factor superfamily cytokine production | 25/1080 | 7.00E-06 | 0.000462 | 25 |
| GO:0001570 | vasculogenesis | 16/1080 | 7.13E-06 | 0.000464 | 16 |
| GO:0018108 | peptidyl-tyrosine phosphorylation | 43/1080 | 7.64E-06 | 0.000491 | 43 |
| GO:2000116 | regulation of cysteine-type endopeptidase activity | 31/1080 | 8.12E-06 | 0.000516 | 31 |
| GO:0050671 | positive regulation of lymphocyte proliferation | 22/1080 | 8.27E-06 | 0.000519 | 22 |
| GO:0022409 | positive regulation of cell-cell adhesion | 35/1080 | 8.61E-06 | 0.000534 | 35 |
| GO:0032946 | positive regulation of mononuclear cell proliferation | 22/1080 | 9.34E-06 | 0.000552 | 22 |
| GO:0018212 | peptidyl-tyrosine modification | 43/1080 | 9.37E-06 | 0.000552 | 43 |
| GO:0032480 | negative regulation of type I interferon production | 12/1080 | 9.44E-06 | 0.000552 | 12 |
| GO:0001666 | response to hypoxia | 41/1080 | 9.48E-06 | 0.000552 | 41 |
| GO:0002526 | acute inflammatory response | 19/1080 | 9.52E-06 | 0.000552 | 19 |
| GO:0032649 | regulation of interferon-gamma production | 19/1080 | 9.52E-06 | 0.000552 | 19 |
| GO:0071706 | tumor necrosis factor superfamily cytokine production | 25/1080 | 9.69E-06 | 0.000556 | 25 |
| GO:1903039 | positive regulation of leukocyte cell-cell adhesion | 31/1080 | 1.15E-05 | 0.000654 | 31 |
| GO:0070665 | positive regulation of leukocyte proliferation | 23/1080 | 1.17E-05 | 0.000654 | 23 |
| GO:0032479 | regulation of type I interferon production | 21/1080 | 1.18E-05 | 0.000657 | 21 |
| GO:0010038 | response to metal ion | 41/1080 | 1.25E-05 | 0.000683 | 41 |
| GO:0006898 | receptor-mediated endocytosis | 39/1080 | 1.26E-05 | 0.000683 | 39 |
| GO:0050691 | regulation of defense response to virus by host | 11/1080 | 1.33E-05 | 0.000714 | 11 |
| GO:0032606 | type I interferon production | 21/1080 | 1.34E-05 | 0.000714 | 21 |
| GO:0060560 | developmental growth involved in morphogenesis | 30/1080 | 1.40E-05 | 0.000738 | 30 |
| GO:0060326 | cell chemotaxis | 37/1080 | 1.43E-05 | 0.00074 | 37 |
| GO:0050673 | epithelial cell proliferation | 47/1080 | 1.43E-05 | 0.00074 | 47 |
| GO:0043123 | positive regulation of I-kappaB kinase/NF-kappaB signaling | 26/1080 | 1.52E-05 | 0.00078 | 26 |
| GO:0009308 | amine metabolic process | 24/1080 | 1.92E-05 | 0.000976 | 24 |
| GO:0010721 | negative regulation of cell development | 25/1080 | 2.00E-05 | 0.001003 | 25 |
| GO:0036293 | response to decreased oxygen levels | 41/1080 | 2.13E-05 | 0.001056 | 41 |
| GO:0050768 | negative regulation of neurogenesis | 21/1080 | 2.16E-05 | 0.001056 | 21 |
| GO:0034142 | toll-like receptor 4 signaling pathway | 11/1080 | 2.18E-05 | 0.001056 | 11 |
| GO:0070482 | response to oxygen levels | 43/1080 | 2.18E-05 | 0.001056 | 43 |
| GO:0071222 | cellular response to lipopolysaccharide | 27/1080 | 2.26E-05 | 0.001073 | 27 |
| GO:0001916 | positive regulation of T cell mediated cytotoxicity | 9/1080 | 2.26E-05 | 0.001073 | 9 |
| GO:0140289 | protein mono-ADP-ribosylation | 6/1080 | 2.38E-05 | 0.001121 | 6 |
| GO:0048568 | embryonic organ development | 45/1080 | 2.48E-05 | 0.001155 | 45 |
| GO:0045785 | positive regulation of cell adhesion | 46/1080 | 2.54E-05 | 0.001174 | 46 |
| GO:0007389 | pattern specification process | 46/1080 | 2.69E-05 | 0.001226 | 46 |
| GO:0009991 | response to extracellular stimulus | 50/1080 | 2.70E-05 | 0.001226 | 50 |
| GO:0071216 | cellular response to biotic stimulus | 30/1080 | 2.76E-05 | 0.001246 | 30 |
| GO:0050688 | regulation of defense response to virus | 14/1080 | 3.00E-05 | 0.001328 | 14 |
| GO:0006919 | activation of cysteine-type endopeptidase activity involved in apoptotic process | 16/1080 | 3.02E-05 | 0.001328 | 16 |
| GO:0032760 | positive regulation of tumor necrosis factor production | 16/1080 | 3.02E-05 | 0.001328 | 16 |
| GO:0050870 | positive regulation of T cell activation | 28/1080 | 3.18E-05 | 0.001388 | 28 |
| GO:1903901 | negative regulation of viral life cycle | 8/1080 | 3.63E-05 | 0.001569 | 8 |
| GO:0016049 | cell growth | 49/1080 | 3.72E-05 | 0.001595 | 49 |
| GO:0051961 | negative regulation of nervous system development | 21/1080 | 3.81E-05 | 0.001619 | 21 |
| GO:0002819 | regulation of adaptive immune response | 24/1080 | 3.89E-05 | 0.001641 | 24 |
| GO:0001914 | regulation of T cell mediated cytotoxicity | 10/1080 | 3.94E-05 | 0.001649 | 10 |
| GO:0006953 | acute-phase response | 11/1080 | 4.31E-05 | 0.001769 | 11 |
| GO:0002695 | negative regulation of leukocyte activation | 25/1080 | 4.31E-05 | 0.001769 | 25 |
| GO:0002824 | positive regulation of adaptive immune response based on somatic recombination of immune receptors built from immunoglobulin superfamily domains | 17/1080 | 4.33E-05 | 0.001769 | 17 |
| GO:0043281 | regulation of cysteine-type endopeptidase activity involved in apoptotic process | 27/1080 | 4.60E-05 | 0.001865 | 27 |
| GO:1903557 | positive regulation of tumor necrosis factor superfamily cytokine production | 16/1080 | 4.66E-05 | 0.001875 | 16 |
| GO:1905521 | regulation of macrophage migration | 10/1080 | 5.03E-05 | 0.002009 | 10 |
| GO:0009310 | amine catabolic process | 9/1080 | 5.42E-05 | 0.002134 | 9 |
| GO:0002544 | chronic inflammatory response | 7/1080 | 5.42E-05 | 0.002134 | 7 |
| GO:0052548 | regulation of endopeptidase activity | 45/1080 | 5.58E-05 | 0.002181 | 45 |
| GO:0003002 | regionalization | 37/1080 | 5.70E-05 | 0.002206 | 37 |
| GO:0002699 | positive regulation of immune effector process | 28/1080 | 5.73E-05 | 0.002206 | 28 |
| GO:1990138 | neuron projection extension | 23/1080 | 6.21E-05 | 0.002373 | 23 |
| GO:0032735 | positive regulation of interleukin-12 production | 10/1080 | 6.36E-05 | 0.002412 | 10 |
| GO:0002703 | regulation of leukocyte mediated immunity | 27/1080 | 6.45E-05 | 0.002412 | 27 |
| GO:0071219 | cellular response to molecule of bacterial origin | 27/1080 | 6.45E-05 | 0.002412 | 27 |
| GO:0001913 | T cell mediated cytotoxicity | 11/1080 | 6.57E-05 | 0.002439 | 11 |
| GO:0032729 | positive regulation of interferon-gamma production | 13/1080 | 6.71E-05 | 0.002475 | 13 |
| GO:0002475 | antigen processing and presentation via MHC class Ib | 6/1080 | 7.01E-05 | 0.002566 | 6 |
| GO:0002822 | regulation of adaptive immune response based on somatic recombination of immune receptors built from immunoglobulin superfamily domains | 22/1080 | 7.42E-05 | 0.002697 | 22 |
| GO:0002718 | regulation of cytokine production involved in immune response | 15/1080 | 7.56E-05 | 0.002731 | 15 |
| GO:0001558 | regulation of cell growth | 43/1080 | 7.63E-05 | 0.002738 | 43 |
| GO:0050866 | negative regulation of cell activation | 26/1080 | 7.87E-05 | 0.002804 | 26 |
| GO:0046597 | negative regulation of viral entry into host cell | 7/1080 | 7.93E-05 | 0.002807 | 7 |
| GO:0060759 | regulation of response to cytokine stimulus | 25/1080 | 8.08E-05 | 0.002839 | 25 |
| GO:0002456 | T cell mediated immunity | 17/1080 | 8.22E-05 | 0.002853 | 17 |
| GO:0002821 | positive regulation of adaptive immune response | 17/1080 | 8.22E-05 | 0.002853 | 17 |
| GO:0043112 | receptor metabolic process | 23/1080 | 9.05E-05 | 0.003118 | 23 |
| GO:0051960 | regulation of nervous system development | 44/1080 | 9.11E-05 | 0.003118 | 44 |
| GO:0009612 | response to mechanical stimulus | 26/1080 | 9.30E-05 | 0.003163 | 26 |
| GO:0010758 | regulation of macrophage chemotaxis | 8/1080 | 9.40E-05 | 0.003177 | 8 |
| GO:0044106 | cellular amine metabolic process | 22/1080 | 9.91E-05 | 0.003328 | 22 |
| GO:0050730 | regulation of peptidyl-tyrosine phosphorylation | 31/1080 | 0.000104 | 0.003457 | 31 |
| GO:0032663 | regulation of interleukin-2 production | 12/1080 | 0.000107 | 0.003538 | 12 |
| GO:0031667 | response to nutrient levels | 46/1080 | 0.000107 | 0.003538 | 46 |
| GO:0050678 | regulation of epithelial cell proliferation | 40/1080 | 0.000109 | 0.003559 | 40 |
| GO:0046631 | alpha-beta T cell activation | 21/1080 | 0.000119 | 0.003863 | 21 |
| GO:0001660 | fever generation | 5/1080 | 0.000121 | 0.003902 | 5 |
| GO:0071456 | cellular response to hypoxia | 26/1080 | 0.000129 | 0.004138 | 26 |
| GO:1904062 | regulation of cation transmembrane transport | 37/1080 | 0.000137 | 0.004365 | 37 |
| GO:0032623 | interleukin-2 production | 12/1080 | 0.00015 | 0.00472 | 12 |
| GO:0038061 | NIK/NF-kappaB signaling | 24/1080 | 0.000151 | 0.00472 | 24 |
| GO:0002221 | pattern recognition receptor signaling pathway | 26/1080 | 0.000151 | 0.00472 | 26 |
| GO:0042102 | positive regulation of T cell proliferation | 16/1080 | 0.000151 | 0.00472 | 16 |
| GO:0070997 | neuron death | 37/1080 | 0.000154 | 0.004773 | 37 |
| GO:0031649 | heat generation | 6/1080 | 0.000169 | 0.005181 | 6 |
| GO:0002704 | negative regulation of leukocyte mediated immunity | 11/1080 | 0.00017 | 0.005181 | 11 |
| GO:1905517 | macrophage migration | 11/1080 | 0.00017 | 0.005181 | 11 |
| GO:0019722 | calcium-mediated signaling | 25/1080 | 0.000171 | 0.005181 | 25 |
| GO:0048511 | rhythmic process | 33/1080 | 0.000173 | 0.00522 | 33 |
| GO:0010001 | glial cell differentiation | 26/1080 | 0.000176 | 0.005279 | 26 |
| GO:0048588 | developmental cell growth | 27/1080 | 0.00018 | 0.005353 | 27 |
| GO:0002478 | antigen processing and presentation of exogenous peptide antigen | 23/1080 | 0.0002 | 0.005896 | 23 |
| GO:2001257 | regulation of cation channel activity | 23/1080 | 0.0002 | 0.005896 | 23 |
| GO:0050920 | regulation of chemotaxis | 27/1080 | 0.000208 | 0.006104 | 27 |
| GO:0071248 | cellular response to metal ion | 24/1080 | 0.00021 | 0.006107 | 24 |
| GO:0042402 | cellular biogenic amine catabolic process | 8/1080 | 0.000213 | 0.006157 | 8 |
| GO:0046135 | pyrimidine nucleoside catabolic process | 7/1080 | 0.000216 | 0.006212 | 7 |
| GO:0048705 | skeletal system morphogenesis | 26/1080 | 0.000221 | 0.006346 | 26 |
| GO:0099637 | neurotransmitter receptor transport | 10/1080 | 0.000224 | 0.00638 | 10 |
| GO:0036294 | cellular response to decreased oxygen levels | 26/1080 | 0.000239 | 0.006766 | 26 |
| GO:0030595 | leukocyte chemotaxis | 27/1080 | 0.000241 | 0.006796 | 27 |
| GO:0002367 | cytokine production involved in immune response | 15/1080 | 0.000246 | 0.006911 | 15 |
| GO:0060538 | skeletal muscle organ development | 21/1080 | 0.000249 | 0.00695 | 21 |
| GO:0043280 | positive regulation of cysteine-type endopeptidase activity involved in apoptotic process | 19/1080 | 0.00025 | 0.00695 | 19 |
| GO:0052547 | regulation of peptidase activity | 45/1080 | 0.000255 | 0.007033 | 45 |
| GO:0071260 | cellular response to mechanical stimulus | 13/1080 | 0.000265 | 0.007285 | 13 |
| GO:0002705 | positive regulation of leukocyte mediated immunity | 18/1080 | 0.000271 | 0.007395 | 18 |
| GO:0002230 | positive regulation of defense response to virus by host | 8/1080 | 0.000272 | 0.007398 | 8 |
| GO:0071241 | cellular response to inorganic substance | 26/1080 | 0.000277 | 0.007482 | 26 |
| GO:0070265 | necrotic cell death | 12/1080 | 0.000281 | 0.007551 | 12 |
| GO:0050731 | positive regulation of peptidyl-tyrosine phosphorylation | 24/1080 | 0.000288 | 0.007709 | 24 |
| GO:0010950 | positive regulation of endopeptidase activity | 23/1080 | 0.000302 | 0.007967 | 23 |
| GO:0071496 | cellular response to external stimulus | 33/1080 | 0.000303 | 0.007967 | 33 |
| GO:0003158 | endothelium development | 19/1080 | 0.000303 | 0.007967 | 19 |
| GO:0062207 | regulation of pattern recognition receptor signaling pathway | 16/1080 | 0.000304 | 0.007967 | 16 |
| GO:0002460 | adaptive immune response based on somatic recombination of immune receptors built from immunoglobulin superfamily domains | 38/1080 | 0.000307 | 0.008022 | 38 |
| GO:0042180 | cellular ketone metabolic process | 29/1080 | 0.000314 | 0.008164 | 29 |
| GO:0002715 | regulation of natural killer cell mediated immunity | 10/1080 | 0.000324 | 0.008366 | 10 |
| GO:0050771 | negative regulation of axonogenesis | 12/1080 | 0.000325 | 0.008371 | 12 |
| GO:0071453 | cellular response to oxygen levels | 27/1080 | 0.000343 | 0.008725 | 27 |
| GO:0002730 | regulation of dendritic cell cytokine production | 5/1080 | 0.000344 | 0.008725 | 5 |
| GO:2001214 | positive regulation of vasculogenesis | 5/1080 | 0.000344 | 0.008725 | 5 |
| GO:0007626 | locomotory behavior | 21/1080 | 0.000353 | 0.008913 | 21 |
| GO:0045953 | negative regulation of natural killer cell mediated cytotoxicity | 6/1080 | 0.000355 | 0.008923 | 6 |
| GO:0048640 | negative regulation of developmental growth | 16/1080 | 0.000378 | 0.009442 | 16 |
| GO:0099590 | neurotransmitter receptor internalization | 7/1080 | 0.000382 | 0.009482 | 7 |
| GO:0019884 | antigen processing and presentation of exogenous antigen | 23/1080 | 0.000383 | 0.009482 | 23 |
| GO:0062012 | regulation of small molecule metabolic process | 43/1080 | 0.000386 | 0.009499 | 43 |
| GO:0045926 | negative regulation of growth | 28/1080 | 0.000389 | 0.009548 | 28 |
| GO:2001056 | positive regulation of cysteine-type endopeptidase activity | 20/1080 | 0.000395 | 0.009611 | 20 |
| GO:0045444 | fat cell differentiation | 26/1080 | 0.000396 | 0.009611 | 26 |
| GO:0034121 | regulation of toll-like receptor signaling pathway | 13/1080 | 0.000397 | 0.009611 | 13 |
| GO:0046718 | viral entry into host cell | 19/1080 | 0.000401 | 0.009655 | 19 |
| GO:0042063 | gliogenesis | 31/1080 | 0.000412 | 0.009864 | 31 |
| GO:0050867 | positive regulation of cell activation | 41/1080 | 0.000415 | 0.009896 | 41 |
| GO:0033003 | regulation of mast cell activation | 9/1080 | 0.000425 | 0.010093 | 9 |
| GO:0050868 | negative regulation of T cell activation | 17/1080 | 0.000436 | 0.010258 | 17 |
| GO:0048732 | gland development | 41/1080 | 0.000436 | 0.010258 | 41 |
| GO:0031341 | regulation of cell killing | 15/1080 | 0.000441 | 0.010343 | 15 |
| GO:0055074 | calcium ion homeostasis | 44/1080 | 0.000458 | 0.010695 | 44 |
| GO:0051250 | negative regulation of lymphocyte activation | 20/1080 | 0.00047 | 0.010909 | 20 |
| GO:0051403 | stress-activated MAPK cascade | 29/1080 | 0.000494 | 0.011334 | 29 |
| GO:0002716 | negative regulation of natural killer cell mediated immunity | 6/1080 | 0.000494 | 0.011334 | 6 |
| GO:0010893 | positive regulation of steroid biosynthetic process | 6/1080 | 0.000494 | 0.011334 | 6 |
| GO:0021675 | nerve development | 12/1080 | 0.000498 | 0.011359 | 12 |
| GO:0001959 | regulation of cytokine-mediated signaling pathway | 22/1080 | 0.00051 | 0.011583 | 22 |
| GO:0001910 | regulation of leukocyte mediated cytotoxicity | 13/1080 | 0.000513 | 0.011619 | 13 |
| GO:0032615 | interleukin-12 production | 11/1080 | 0.000531 | 0.011808 | 11 |
| GO:0032655 | regulation of interleukin-12 production | 11/1080 | 0.000531 | 0.011808 | 11 |
| GO:0045576 | mast cell activation | 11/1080 | 0.000531 | 0.011808 | 11 |
| GO:0002371 | dendritic cell cytokine production | 5/1080 | 0.000533 | 0.011808 | 5 |
| GO:0031650 | regulation of heat generation | 5/1080 | 0.000533 | 0.011808 | 5 |
| GO:0006471 | protein ADP-ribosylation | 8/1080 | 0.000538 | 0.011812 | 8 |
| GO:0009164 | nucleoside catabolic process | 8/1080 | 0.000538 | 0.011812 | 8 |
| GO:0097300 | programmed necrotic cell death | 10/1080 | 0.000541 | 0.011837 | 10 |
| GO:0072507 | divalent inorganic cation homeostasis | 47/1080 | 0.000561 | 0.012216 | 47 |
| GO:0031623 | receptor internalization | 16/1080 | 0.000574 | 0.012452 | 16 |
| GO:0048562 | embryonic organ morphogenesis | 30/1080 | 0.000611 | 0.013182 | 30 |
| GO:0062014 | negative regulation of small molecule metabolic process | 15/1080 | 0.000612 | 0.013182 | 15 |
| GO:0030517 | negative regulation of axon extension | 9/1080 | 0.000617 | 0.013226 | 9 |
| GO:0001764 | neuron migration | 19/1080 | 0.000625 | 0.013282 | 19 |
| GO:1903169 | regulation of calcium ion transmembrane transport | 19/1080 | 0.000625 | 0.013282 | 19 |
| GO:0002711 | positive regulation of T cell mediated immunity | 10/1080 | 0.000636 | 0.013467 | 10 |
| GO:0002224 | toll-like receptor signaling pathway | 20/1080 | 0.000656 | 0.013787 | 20 |
| GO:0002706 | regulation of lymphocyte mediated immunity | 20/1080 | 0.000656 | 0.013787 | 20 |
| GO:0070588 | calcium ion transmembrane transport | 32/1080 | 0.00067 | 0.014011 | 32 |
| GO:0032816 | positive regulation of natural killer cell activation | 6/1080 | 0.000672 | 0.014011 | 6 |
| GO:0007517 | muscle organ development | 33/1080 | 0.000676 | 0.014042 | 33 |
| GO:0002685 | regulation of leukocyte migration | 24/1080 | 0.000698 | 0.014436 | 24 |
| GO:0001909 | leukocyte mediated cytotoxicity | 16/1080 | 0.000701 | 0.014436 | 16 |
| GO:0008344 | adult locomotory behavior | 11/1080 | 0.000709 | 0.01455 | 11 |
| GO:0042269 | regulation of natural killer cell mediated cytotoxicity | 9/1080 | 0.000737 | 0.015024 | 9 |
| GO:0007519 | skeletal muscle tissue development | 19/1080 | 0.00074 | 0.015024 | 19 |
| GO:0097529 | myeloid leukocyte migration | 25/1080 | 0.000741 | 0.015024 | 25 |
| GO:1903131 | mononuclear cell differentiation | 40/1080 | 0.000751 | 0.015173 | 40 |
| GO:0048675 | axon extension | 16/1080 | 0.000773 | 0.015515 | 16 |
| GO:0002698 | negative regulation of immune effector process | 17/1080 | 0.000774 | 0.015515 | 17 |
| GO:1902187 | negative regulation of viral release from host cell | 5/1080 | 0.00079 | 0.015703 | 5 |
| GO:0001659 | temperature homeostasis | 21/1080 | 0.000792 | 0.015703 | 21 |
| GO:0019932 | second-messenger-mediated signaling | 32/1080 | 0.000792 | 0.015703 | 32 |
| GO:0032703 | negative regulation of interleukin-2 production | 7/1080 | 0.00081 | 0.015994 | 7 |
| GO:0031343 | positive regulation of cell killing | 11/1080 | 0.000815 | 0.016044 | 11 |
| GO:1901019 | regulation of calcium ion transmembrane transporter activity | 13/1080 | 0.000833 | 0.016269 | 13 |
| GO:0032412 | regulation of ion transmembrane transporter activity | 28/1080 | 0.000833 | 0.016269 | 28 |
| GO:0061387 | regulation of extent of cell growth | 15/1080 | 0.000837 | 0.016288 | 15 |
| GO:0002707 | negative regulation of lymphocyte mediated immunity | 9/1080 | 0.000875 | 0.016945 | 9 |
| GO:0002696 | positive regulation of leukocyte activation | 39/1080 | 0.00088 | 0.016945 | 39 |
| GO:0050767 | regulation of neurogenesis | 35/1080 | 0.000881 | 0.016945 | 35 |
| GO:0002053 | positive regulation of mesenchymal cell proliferation | 6/1080 | 0.000896 | 0.017179 | 6 |
| GO:0006874 | cellular calcium ion homeostasis | 42/1080 | 0.000906 | 0.017248 | 42 |
| GO:1903038 | negative regulation of leukocyte cell-cell adhesion | 18/1080 | 0.000906 | 0.017248 | 18 |
| GO:0010565 | regulation of cellular ketone metabolic process | 22/1080 | 0.000929 | 0.017554 | 22 |
| GO:0030308 | negative regulation of cell growth | 22/1080 | 0.000929 | 0.017554 | 22 |
| GO:0042267 | natural killer cell mediated cytotoxicity | 11/1080 | 0.000935 | 0.017562 | 11 |
| GO:0032874 | positive regulation of stress-activated MAPK cascade | 16/1080 | 0.000936 | 0.017562 | 16 |
| GO:0015711 | organic anion transport | 37/1080 | 0.000953 | 0.017814 | 37 |
| GO:0046633 | alpha-beta T cell proliferation | 8/1080 | 0.000981 | 0.018286 | 8 |
| GO:0001912 | positive regulation of leukocyte mediated cytotoxicity | 10/1080 | 0.001006 | 0.018686 | 10 |
| GO:0045940 | positive regulation of steroid metabolic process | 7/1080 | 0.001015 | 0.018785 | 7 |
| GO:1901222 | regulation of NIK/NF-kappaB signaling | 15/1080 | 0.001023 | 0.018856 | 15 |
| GO:0007409 | axonogenesis | 43/1080 | 0.001098 | 0.020168 | 43 |
| GO:0007623 | circadian rhythm | 24/1080 | 0.001121 | 0.020399 | 24 |
| GO:0030178 | negative regulation of Wnt signaling pathway | 24/1080 | 0.001121 | 0.020399 | 24 |
| GO:0070304 | positive regulation of stress-activated protein kinase signaling cascade | 16/1080 | 0.001127 | 0.020399 | 16 |
| GO:0071871 | response to epinephrine | 5/1080 | 0.00113 | 0.020399 | 5 |
| GO:0072075 | metanephric mesenchyme development | 5/1080 | 0.00113 | 0.020399 | 5 |
| GO:0010952 | positive regulation of peptidase activity | 23/1080 | 0.001139 | 0.020503 | 23 |
| GO:0001911 | negative regulation of leukocyte mediated cytotoxicity | 6/1080 | 0.001173 | 0.020908 | 6 |
| GO:0035458 | cellular response to interferon-beta | 6/1080 | 0.001173 | 0.020908 | 6 |
| GO:0010463 | mesenchymal cell proliferation | 8/1080 | 0.001182 | 0.020908 | 8 |
| GO:0045214 | sarcomere organization | 8/1080 | 0.001182 | 0.020908 | 8 |
| GO:0048246 | macrophage chemotaxis | 8/1080 | 0.001182 | 0.020908 | 8 |
| GO:0031098 | stress-activated protein kinase signaling cascade | 29/1080 | 0.001204 | 0.021135 | 29 |
| GO:0044409 | entry into host | 19/1080 | 0.001205 | 0.021135 | 19 |
| GO:0051924 | regulation of calcium ion transport | 26/1080 | 0.001206 | 0.021135 | 26 |
| GO:1901214 | regulation of neuron death | 31/1080 | 0.001217 | 0.021254 | 31 |
| GO:0002688 | regulation of leukocyte chemotaxis | 16/1080 | 0.001235 | 0.021416 | 16 |
| GO:0045446 | endothelial cell differentiation | 16/1080 | 0.001235 | 0.021416 | 16 |
| GO:0002708 | positive regulation of lymphocyte mediated immunity | 15/1080 | 0.001242 | 0.021472 | 15 |
| GO:0051968 | positive regulation of synaptic transmission glutamatergic | glutamatergic | 0.00126 | 0.021706 | 7 |
| GO:0006816 | calcium ion transport | 39/1080 | 0.001273 | 0.021857 | 39 |
| GO:0015718 | monocarboxylic acid transport | 21/1080 | 0.001328 | 0.02273 | 21 |
| GO:0032757 | positive regulation of interleukin-8 production | 10/1080 | 0.00134 | 0.022863 | 10 |
| GO:0007568 | aging | 31/1080 | 0.001353 | 0.023022 | 31 |
| GO:0033238 | regulation of cellular amine metabolic process | 12/1080 | 0.001363 | 0.023108 | 12 |
| GO:0002228 | natural killer cell mediated immunity | 11/1080 | 0.001381 | 0.023196 | 11 |
| GO:0019226 | transmission of nerve impulse | 11/1080 | 0.001381 | 0.023196 | 11 |
| GO:0032922 | circadian regulation of gene expression | 11/1080 | 0.001381 | 0.023196 | 11 |
| GO:0050679 | positive regulation of epithelial cell proliferation | 23/1080 | 0.001392 | 0.023294 | 23 |
| GO:0001906 | cell killing | 21/1080 | 0.001425 | 0.023733 | 21 |
| GO:0072503 | cellular divalent inorganic cation homeostasis | 44/1080 | 0.001427 | 0.023733 | 44 |
| GO:0050806 | positive regulation of synaptic transmission | 18/1080 | 0.001486 | 0.024636 | 18 |
| GO:1901863 | positive regulation of muscle tissue development | 6/1080 | 0.001511 | 0.024969 | 6 |
| GO:0002709 | regulation of T cell mediated immunity | 12/1080 | 0.001528 | 0.02503 | 12 |
| GO:0140029 | exocytic process | 12/1080 | 0.001528 | 0.02503 | 12 |
| GO:0071347 | cellular response to interleukin-1 | 21/1080 | 0.001529 | 0.02503 | 21 |
| GO:0032196 | transposition | 7/1080 | 0.001548 | 0.025266 | 7 |
| GO:2001212 | regulation of vasculogenesis | 5/1080 | 0.001566 | 0.025489 | 5 |
| GO:0022898 | regulation of transmembrane transporter activity | 28/1080 | 0.001577 | 0.025585 | 28 |
| GO:0010975 | regulation of neuron projection development | 40/1080 | 0.001615 | 0.026114 | 40 |
| GO:0001503 | ossification | 38/1080 | 0.00163 | 0.026291 | 38 |
| GO:0071622 | regulation of granulocyte chemotaxis | 9/1080 | 0.001652 | 0.02656 | 9 |
| GO:0070555 | response to interleukin-1 | 23/1080 | 0.00169 | 0.026982 | 23 |
| GO:0032817 | regulation of natural killer cell proliferation | 4/1080 | 0.001699 | 0.026982 | 4 |
| GO:0043301 | negative regulation of leukocyte degranulation | 4/1080 | 0.001699 | 0.026982 | 4 |
| GO:0070383 | DNA cytosine deamination | 4/1080 | 0.001699 | 0.026982 | 4 |
| GO:0099003 | vesicle-mediated transport in synapse | 22/1080 | 0.001728 | 0.027363 | 22 |
| GO:0030239 | myofibril assembly | 10/1080 | 0.001758 | 0.027752 | 10 |
| GO:0032675 | regulation of interleukin-6 production | 19/1080 | 0.001763 | 0.027753 | 19 |
| GO:0035249 | synaptic transmission glutamatergic | glutamatergic | 0.001789 | 0.028083 | 13 |
| GO:0030001 | metal ion transport | 44/1080 | 0.001817 | 0.028426 | 44 |
| GO:0042391 | regulation of membrane potential | 40/1080 | 0.001835 | 0.028621 | 40 |
| GO:0046942 | carboxylic acid transport | 29/1080 | 0.001862 | 0.028964 | 29 |
| GO:0007616 | long-term memory | 7/1080 | 0.001884 | 0.029148 | 7 |
| GO:0035725 | sodium ion transmembrane transport | 20/1080 | 0.001897 | 0.029148 | 20 |
| GO:0071621 | granulocyte chemotaxis | 16/1080 | 0.001911 | 0.029148 | 16 |
| GO:0034656 | nucleobase-containing small molecule catabolic process | 9/1080 | 0.001914 | 0.029148 | 9 |
| GO:0045912 | negative regulation of carbohydrate metabolic process | 9/1080 | 0.001914 | 0.029148 | 9 |
| GO:0061436 | establishment of skin barrier | 6/1080 | 0.001918 | 0.029148 | 6 |
| GO:1905523 | positive regulation of macrophage migration | 6/1080 | 0.001918 | 0.029148 | 6 |
| GO:0015849 | organic acid transport | 32/1080 | 0.001918 | 0.029148 | 32 |
| GO:0030217 | T cell differentiation | 26/1080 | 0.001934 | 0.029312 | 26 |
| GO:0050804 | modulation of chemical synaptic transmission | 38/1080 | 0.001944 | 0.029367 | 38 |
| GO:0051145 | smooth muscle cell differentiation | 11/1080 | 0.00199 | 0.029976 | 11 |
| GO:0097530 | granulocyte migration | 18/1080 | 0.002026 | 0.030406 | 18 |
| GO:0099177 | regulation of trans-synaptic signaling | 38/1080 | 0.00203 | 0.030406 | 38 |
| GO:0010959 | regulation of metal ion transport | 26/1080 | 0.002048 | 0.030587 | 26 |
| GO:0010759 | positive regulation of macrophage chemotaxis | 5/1080 | 0.002115 | 0.031416 | 5 |
| GO:0071850 | mitotic cell cycle arrest | 5/1080 | 0.002115 | 0.031416 | 5 |
| GO:0030516 | regulation of axon extension | 13/1080 | 0.002189 | 0.032179 | 13 |
| GO:0006576 | cellular biogenic amine metabolic process | 14/1080 | 0.002191 | 0.032179 | 14 |
| GO:0030593 | neutrophil chemotaxis | 14/1080 | 0.002191 | 0.032179 | 14 |
| GO:0055002 | striated muscle cell development | 14/1080 | 0.002191 | 0.032179 | 14 |
| GO:0043900 | regulation of multi-organism process | 9/1080 | 0.002208 | 0.032258 | 9 |
| GO:1903307 | positive regulation of regulated secretory pathway | 9/1080 | 0.002208 | 0.032258 | 9 |
| GO:0045089 | positive regulation of innate immune response | 24/1080 | 0.002228 | 0.032448 | 24 |
| GO:0032814 | regulation of natural killer cell activation | 7/1080 | 0.002275 | 0.032964 | 7 |
| GO:1905330 | regulation of morphogenesis of an epithelium | 10/1080 | 0.002276 | 0.032964 | 10 |
| GO:0071900 | regulation of protein serine/threonine kinase activity | 44/1080 | 0.002297 | 0.033178 | 44 |
| GO:0072006 | nephron development | 17/1080 | 0.002322 | 0.03345 | 17 |
| GO:0062208 | positive regulation of pattern recognition receptor signaling pathway | 8/1080 | 0.002329 | 0.033458 | 8 |
| GO:0032635 | interleukin-6 production | 19/1080 | 0.002355 | 0.033739 | 19 |
| GO:0001562 | response to protozoan | 6/1080 | 0.002402 | 0.034054 | 6 |
| GO:0031664 | regulation of lipopolysaccharide-mediated signaling pathway | 6/1080 | 0.002402 | 0.034054 | 6 |
| GO:0042537 | benzene-containing compound metabolic process | 6/1080 | 0.002402 | 0.034054 | 6 |
| GO:0042832 | defense response to protozoan | 6/1080 | 0.002402 | 0.034054 | 6 |
| GO:0051251 | positive regulation of lymphocyte activation | 34/1080 | 0.002457 | 0.03473 | 34 |
| GO:0052126 | movement in host environment | 20/1080 | 0.002496 | 0.035197 | 20 |
| GO:0050921 | positive regulation of chemotaxis | 17/1080 | 0.002508 | 0.035264 | 17 |
| GO:0001787 | natural killer cell proliferation | 4/1080 | 0.002549 | 0.035467 | 4 |
| GO:0070189 | kynurenine metabolic process | 4/1080 | 0.002549 | 0.035467 | 4 |
| GO:0070213 | protein auto-ADP-ribosylation | 4/1080 | 0.002549 | 0.035467 | 4 |
| GO:2000644 | regulation of receptor catabolic process | 4/1080 | 0.002549 | 0.035467 | 4 |
| GO:0007411 | axon guidance | 28/1080 | 0.002558 | 0.035489 | 28 |
| GO:0050808 | synapse organization | 38/1080 | 0.002619 | 0.036241 | 38 |
| GO:0097485 | neuron projection guidance | 28/1080 | 0.002693 | 0.037141 | 28 |
| GO:0015701 | bicarbonate transport | 8/1080 | 0.00272 | 0.037141 | 8 |
| GO:0032620 | interleukin-17 production | 8/1080 | 0.00272 | 0.037141 | 8 |
| GO:0070266 | necroptotic process | 8/1080 | 0.00272 | 0.037141 | 8 |
| GO:0046640 | regulation of alpha-beta T cell proliferation | 7/1080 | 0.002726 | 0.037141 | 7 |
| GO:1901385 | regulation of voltage-gated calcium channel activity | 7/1080 | 0.002726 | 0.037141 | 7 |
| GO:0022408 | negative regulation of cell-cell adhesion | 21/1080 | 0.002783 | 0.037651 | 21 |
| GO:0032740 | positive regulation of interleukin-17 production | 5/1080 | 0.002792 | 0.037651 | 5 |
| GO:0098884 | postsynaptic neurotransmitter receptor internalization | 5/1080 | 0.002792 | 0.037651 | 5 |
| GO:0140239 | postsynaptic endocytosis | 5/1080 | 0.002792 | 0.037651 | 5 |
| GO:0009166 | nucleotide catabolic process | 11/1080 | 0.0028 | 0.037662 | 11 |
| GO:1902105 | regulation of leukocyte differentiation | 28/1080 | 0.002835 | 0.038039 | 28 |
| GO:0051235 | maintenance of location | 31/1080 | 0.002866 | 0.038352 | 31 |
| GO:0007584 | response to nutrient | 19/1080 | 0.002901 | 0.03872 | 19 |
| GO:0062013 | positive regulation of small molecule metabolic process | 17/1080 | 0.002917 | 0.03872 | 17 |
| GO:1904064 | positive regulation of cation transmembrane transport | 17/1080 | 0.002917 | 0.03872 | 17 |
| GO:0010464 | regulation of mesenchymal cell proliferation | 6/1080 | 0.002974 | 0.03872 | 6 |
| GO:0010528 | regulation of transposition | 6/1080 | 0.002974 | 0.03872 | 6 |
| GO:0010529 | negative regulation of transposition | 6/1080 | 0.002974 | 0.03872 | 6 |
| GO:0021602 | cranial nerve morphogenesis | 6/1080 | 0.002974 | 0.03872 | 6 |
| GO:0021952 | central nervous system projection neuron axonogenesis | 6/1080 | 0.002974 | 0.03872 | 6 |
| GO:0031342 | negative regulation of cell killing | 6/1080 | 0.002974 | 0.03872 | 6 |
| GO:0033561 | regulation of water loss via skin | 6/1080 | 0.002974 | 0.03872 | 6 |
| GO:2000311 | regulation of AMPA receptor activity | 6/1080 | 0.002974 | 0.03872 | 6 |
| GO:0060284 | regulation of cell development | 43/1080 | 0.003001 | 0.038975 | 43 |
| GO:0046632 | alpha-beta T cell differentiation | 14/1080 | 0.003135 | 0.040473 | 14 |
| GO:0030509 | BMP signaling pathway | 18/1080 | 0.003139 | 0.040473 | 18 |
| GO:0046434 | organophosphate catabolic process | 18/1080 | 0.003139 | 0.040473 | 18 |
| GO:0010677 | negative regulation of cellular carbohydrate metabolic process | 8/1080 | 0.003161 | 0.040549 | 8 |
| GO:1901658 | glycosyl compound catabolic process | 8/1080 | 0.003161 | 0.040549 | 8 |
| GO:0042692 | muscle cell differentiation | 34/1080 | 0.003211 | 0.040997 | 34 |
| GO:0046889 | positive regulation of lipid biosynthetic process | 12/1080 | 0.003211 | 0.040997 | 12 |
| GO:0002573 | myeloid leukocyte differentiation | 22/1080 | 0.003241 | 0.041176 | 22 |
| GO:0044706 | multi-multicellular organism process | 22/1080 | 0.003241 | 0.041176 | 22 |
| GO:0007162 | negative regulation of cell adhesion | 29/1080 | 0.003258 | 0.041296 | 29 |
| GO:0035914 | skeletal muscle cell differentiation | 10/1080 | 0.003275 | 0.041322 | 10 |
| GO:0120254 | olefinic compound metabolic process | 15/1080 | 0.003276 | 0.041322 | 15 |
| GO:0071772 | response to BMP | 19/1080 | 0.00332 | 0.041685 | 19 |
| GO:0071773 | cellular response to BMP stimulus | 19/1080 | 0.00332 | 0.041685 | 19 |
| GO:0098657 | import into cell | 24/1080 | 0.003334 | 0.041758 | 24 |
| GO:0032409 | regulation of transporter activity | 28/1080 | 0.003468 | 0.043227 | 28 |
| GO:0097193 | intrinsic apoptotic signaling pathway | 28/1080 | 0.003468 | 0.043227 | 28 |
| GO:0055001 | muscle cell development | 19/1080 | 0.003548 | 0.043817 | 19 |
| GO:0048643 | positive regulation of skeletal muscle tissue development | 5/1080 | 0.003612 | 0.043817 | 5 |
| GO:0062099 | negative regulation of programmed necrotic cell death | 5/1080 | 0.003612 | 0.043817 | 5 |
| GO:0072074 | kidney mesenchyme development | 5/1080 | 0.003612 | 0.043817 | 5 |
| GO:0098543 | detection of other organism | 5/1080 | 0.003612 | 0.043817 | 5 |
| GO:0071624 | positive regulation of granulocyte chemotaxis | 6/1080 | 0.003641 | 0.043817 | 6 |
| GO:0090200 | positive regulation of release of cytochrome c from mitochondria | 6/1080 | 0.003641 | 0.043817 | 6 |
| GO:0006109 | regulation of carbohydrate metabolic process | 22/1080 | 0.003649 | 0.043817 | 22 |
| GO:0002863 | positive regulation of inflammatory response to antigenic stimulus | 4/1080 | 0.003652 | 0.043817 | 4 |
| GO:0006216 | cytidine catabolic process | 4/1080 | 0.003652 | 0.043817 | 4 |
| GO:0009972 | cytidine deamination | 4/1080 | 0.003652 | 0.043817 | 4 |
| GO:0016554 | cytidine to uridine editing | 4/1080 | 0.003652 | 0.043817 | 4 |
| GO:0033004 | negative regulation of mast cell activation | 4/1080 | 0.003652 | 0.043817 | 4 |
| GO:0046087 | cytidine metabolic process | 4/1080 | 0.003652 | 0.043817 | 4 |
| GO:0071872 | cellular response to epinephrine stimulus | 4/1080 | 0.003652 | 0.043817 | 4 |
| GO:0003009 | skeletal muscle contraction | 8/1080 | 0.003655 | 0.043817 | 8 |
| GO:0030111 | regulation of Wnt signaling pathway | 34/1080 | 0.003656 | 0.043817 | 34 |
| GO:0032872 | regulation of stress-activated MAPK cascade | 20/1080 | 0.00369 | 0.04412 | 20 |
| GO:0051701 | biological process involved in interaction with host | 23/1080 | 0.003699 | 0.044124 | 23 |
| GO:0002833 | positive regulation of response to biotic stimulus | 26/1080 | 0.003715 | 0.044221 | 26 |
| GO:0006213 | pyrimidine nucleoside metabolic process | 7/1080 | 0.003829 | 0.04537 | 7 |
| GO:0097484 | dendrite extension | 7/1080 | 0.003829 | 0.04537 | 7 |
| GO:0050830 | defense response to Gram-positive bacterium | 13/1080 | 0.003844 | 0.045447 | 13 |
| GO:0051279 | regulation of release of sequestered calcium ion into cytosol | 11/1080 | 0.003857 | 0.045497 | 11 |
| GO:0061458 | reproductive system development | 37/1080 | 0.003914 | 0.046067 | 37 |
| GO:0009896 | positive regulation of catabolic process | 40/1080 | 0.003932 | 0.046177 | 40 |
| GO:0048639 | positive regulation of developmental growth | 19/1080 | 0.004044 | 0.047384 | 19 |
| GO:0042130 | negative regulation of T cell proliferation | 10/1080 | 0.004115 | 0.048009 | 10 |
| GO:0046635 | positive regulation of alpha-beta T cell activation | 10/1080 | 0.004115 | 0.048009 | 10 |
| GO:0035270 | endocrine system development | 15/1080 | 0.004157 | 0.048393 | 15 |
| GO:0002700 | regulation of production of molecular mediator of immune response | 17/1080 | 0.004189 | 0.048432 | 17 |
| GO:0002702 | positive regulation of production of molecular mediator of immune response | 13/1080 | 0.004199 | 0.048432 | 13 |
| GO:0097553 | calcium ion transmembrane import into cytosol | 16/1080 | 0.004201 | 0.048432 | 16 |
| GO:0006195 | purine nucleotide catabolic process | 8/1080 | 0.004207 | 0.048432 | 8 |
| GO:0050873 | brown fat cell differentiation | 8/1080 | 0.004207 | 0.048432 | 8 |
| GO:0050879 | multicellular organismal movement | 9/1080 | 0.004261 | 0.048629 | 9 |
| GO:0050881 | musculoskeletal movement | 9/1080 | 0.004261 | 0.048629 | 9 |
| GO:0060135 | maternal process involved in female pregnancy | 9/1080 | 0.004261 | 0.048629 | 9 |
| GO:0060760 | positive regulation of response to cytokine stimulus | 9/1080 | 0.004261 | 0.048629 | 9 |
| GO:0070098 | chemokine-mediated signaling pathway | 12/1080 | 0.004286 | 0.048816 | 12 |
| GO:0006836 | neurotransmitter transport | 22/1080 | 0.004341 | 0.049228 | 22 |
| GO:0051651 | maintenance of location in cell | 22/1080 | 0.004341 | 0.049228 | 22 |
| GO:0002507 | tolerance induction | 6/1080 | 0.004413 | 0.049725 | 6 |
| GO:0007271 | synaptic transmissioncholinergic | cholinergic | 0.004413 | 0.049725 | 6 |
| GO:0010575 | positive regulation of vascular endothelial growth factor production | 6/1080 | 0.004413 | 0.049725 | 6 |
| GO:0070302 | regulation of stress-activated protein kinase signaling cascade | 20/1080 | 0.004445 | 0.049979 | 20 |

**Supplementary Table 9. The GO-BP enrichment of up-regulated DEGs in U-251 MG cells of 24h**

| ID | Description | GeneRatio | pvalue | p.adjust | Count |
| --- | --- | --- | --- | --- | --- |
| GO:0051607 | defense response to virus | 67/955 | 2.46E-29 | 6.34E-26 | 67 |
| GO:0140546 | defense response to symbiont | 67/955 | 2.46E-29 | 6.34E-26 | 67 |
| GO:0009615 | response to virus | 76/955 | 4.12E-27 | 6.06E-24 | 76 |
| GO:0060337 | type I interferon signaling pathway | 40/955 | 4.70E-27 | 6.06E-24 | 40 |
| GO:0071357 | cellular response to type I interferon | 40/955 | 7.68E-27 | 7.92E-24 | 40 |
| GO:0034340 | response to type I interferon | 40/955 | 8.02E-26 | 6.89E-23 | 40 |
| GO:0034341 | response to interferon-gamma | 53/955 | 2.14E-24 | 1.58E-21 | 53 |
| GO:0048525 | negative regulation of viral process | 34/955 | 1.53E-21 | 9.88E-19 | 34 |
| GO:0060333 | interferon-gamma-mediated signaling pathway | 34/955 | 3.57E-21 | 2.05E-18 | 34 |
| GO:0071346 | cellular response to interferon-gamma | 45/955 | 7.93E-20 | 4.09E-17 | 45 |
| GO:0045071 | negative regulation of viral genome replication | 25/955 | 1.24E-18 | 5.82E-16 | 25 |
| GO:1903900 | regulation of viral life cycle | 38/955 | 4.20E-17 | 1.80E-14 | 38 |
| GO:0050792 | regulation of viral process | 40/955 | 4.50E-15 | 1.78E-12 | 40 |
| GO:0045069 | regulation of viral genome replication | 26/955 | 2.25E-14 | 8.29E-12 | 26 |
| GO:0002831 | regulation of response to biotic stimulus | 62/955 | 2.69E-14 | 9.24E-12 | 62 |
| GO:0043903 | regulation of biological process involved in symbiotic interaction | 40/955 | 3.44E-14 | 1.11E-11 | 40 |
| GO:0001819 | positive regulation of cytokine production | 62/955 | 1.67E-13 | 5.08E-11 | 62 |
| GO:0019885 | antigen processing and presentation of endogenous peptide antigen via MHC class I | 12/955 | 1.30E-12 | 3.72E-10 | 12 |
| GO:0002483 | antigen processing and presentation of endogenous peptide antigen | 12/955 | 9.62E-12 | 2.61E-09 | 12 |
| GO:0034612 | response to tumor necrosis factor | 48/955 | 1.32E-11 | 3.41E-09 | 48 |
| GO:0002237 | response to molecule of bacterial origin | 50/955 | 1.99E-11 | 4.89E-09 | 50 |
| GO:0032496 | response to lipopolysaccharide | 48/955 | 2.59E-11 | 6.06E-09 | 48 |
| GO:0019079 | viral genome replication | 28/955 | 4.60E-11 | 1.03E-08 | 28 |
| GO:0002683 | negative regulation of immune system process | 53/955 | 1.80E-10 | 3.87E-08 | 53 |
| GO:0045088 | regulation of innate immune response | 45/955 | 2.99E-10 | 6.16E-08 | 45 |
| GO:0071356 | cellular response to tumor necrosis factor | 43/955 | 4.37E-10 | 8.66E-08 | 43 |
| GO:0002697 | regulation of immune effector process | 57/955 | 5.68E-10 | 1.08E-07 | 57 |
| GO:0002474 | antigen processing and presentation of peptide antigen via MHC class I | 23/955 | 5.93E-10 | 1.09E-07 | 23 |
| GO:0019883 | antigen processing and presentation of endogenous antigen | 12/955 | 1.33E-09 | 2.36E-07 | 12 |
| GO:0046596 | regulation of viral entry into host cell | 14/955 | 6.47E-09 | 1.11E-06 | 14 |
| GO:0051091 | positive regulation of DNA-binding transcription factor activity | 38/955 | 7.24E-09 | 1.20E-06 | 38 |
| GO:0042590 | antigen processing and presentation of exogenous peptide antigen via MHC class I | 19/955 | 1.25E-08 | 2.01E-06 | 19 |
| GO:0042098 | T cell proliferation | 31/955 | 1.41E-08 | 2.20E-06 | 31 |
| GO:0035456 | response to interferon-beta | 12/955 | 1.53E-08 | 2.26E-06 | 12 |
| GO:0045824 | negative regulation of innate immune response | 17/955 | 1.54E-08 | 2.26E-06 | 17 |
| GO:0019058 | viral life cycle | 44/955 | 2.13E-08 | 2.97E-06 | 44 |
| GO:0042110 | T cell activation | 54/955 | 2.13E-08 | 2.97E-06 | 54 |
| GO:0002832 | negative regulation of response to biotic stimulus | 21/955 | 2.25E-08 | 3.06E-06 | 21 |
| GO:0002479 | antigen processing and presentation of exogenous peptide antigen via MHC class I TAP-dependent | TAP-dependent | 2.53E-08 | 3.34E-06 | 18 |
| GO:0051092 | positive regulation of NF-kappaB transcription factor activity | 27/955 | 2.91E-08 | 3.75E-06 | 27 |
| GO:0007249 | I-kappaB kinase/NF-kappaB signaling | 38/955 | 3.61E-08 | 4.53E-06 | 38 |
| GO:1903037 | regulation of leukocyte cell-cell adhesion | 42/955 | 3.69E-08 | 4.53E-06 | 42 |
| GO:0070663 | regulation of leukocyte proliferation | 34/955 | 6.09E-08 | 7.30E-06 | 34 |
| GO:0052372 | modulation by symbiont of entry into host | 14/955 | 6.36E-08 | 7.30E-06 | 14 |
| GO:0070661 | leukocyte proliferation | 40/955 | 6.37E-08 | 7.30E-06 | 40 |
| GO:0050670 | regulation of lymphocyte proliferation | 32/955 | 8.04E-08 | 9.01E-06 | 32 |
| GO:0043122 | regulation of I-kappaB kinase/NF-kappaB signaling | 34/955 | 8.28E-08 | 9.08E-06 | 34 |
| GO:0007159 | leukocyte cell-cell adhesion | 44/955 | 9.43E-08 | 1.01E-05 | 44 |
| GO:0032944 | regulation of mononuclear cell proliferation | 32/955 | 9.95E-08 | 1.05E-05 | 32 |
| GO:0051090 | regulation of DNA-binding transcription factor activity | 50/955 | 1.03E-07 | 1.06E-05 | 50 |
| GO:0046651 | lymphocyte proliferation | 37/955 | 1.09E-07 | 1.10E-05 | 37 |
| GO:0032943 | mononuclear cell proliferation | 37/955 | 1.43E-07 | 1.42E-05 | 37 |
| GO:0032609 | interferon-gamma production | 21/955 | 1.77E-07 | 1.72E-05 | 21 |
| GO:0050863 | regulation of T cell activation | 40/955 | 2.27E-07 | 2.17E-05 | 40 |
| GO:0050727 | regulation of inflammatory response | 43/955 | 2.52E-07 | 2.36E-05 | 43 |
| GO:0001818 | negative regulation of cytokine production | 43/955 | 2.71E-07 | 2.50E-05 | 43 |
| GO:0019882 | antigen processing and presentation | 32/955 | 3.05E-07 | 2.76E-05 | 32 |
| GO:0042129 | regulation of T cell proliferation | 26/955 | 3.51E-07 | 3.12E-05 | 26 |
| GO:0035455 | response to interferon-alpha | 9/955 | 3.57E-07 | 3.12E-05 | 9 |
| GO:0031348 | negative regulation of defense response | 32/955 | 3.71E-07 | 3.19E-05 | 32 |
| GO:0022407 | regulation of cell-cell adhesion | 48/955 | 3.89E-07 | 3.29E-05 | 48 |
| GO:0031349 | positive regulation of defense response | 42/955 | 4.52E-07 | 3.76E-05 | 42 |
| GO:0032680 | regulation of tumor necrosis factor production | 25/955 | 4.88E-07 | 4.00E-05 | 25 |
| GO:0050777 | negative regulation of immune response | 24/955 | 5.28E-07 | 4.25E-05 | 24 |
| GO:0048002 | antigen processing and presentation of peptide antigen | 28/955 | 5.50E-07 | 4.36E-05 | 28 |
| GO:0032640 | tumor necrosis factor production | 25/955 | 6.20E-07 | 4.85E-05 | 25 |
| GO:0033209 | tumor necrosis factor-mediated signaling pathway | 26/955 | 7.04E-07 | 5.42E-05 | 26 |
| GO:1903555 | regulation of tumor necrosis factor superfamily cytokine production | 25/955 | 7.85E-07 | 5.95E-05 | 25 |
| GO:1903039 | positive regulation of leukocyte cell-cell adhesion | 31/955 | 9.38E-07 | 7.01E-05 | 31 |
| GO:0071706 | tumor necrosis factor superfamily cytokine production | 25/955 | 1.11E-06 | 8.16E-05 | 25 |
| GO:0050671 | positive regulation of lymphocyte proliferation | 22/955 | 1.13E-06 | 8.22E-05 | 22 |
| GO:0032946 | positive regulation of mononuclear cell proliferation | 22/955 | 1.29E-06 | 9.22E-05 | 22 |
| GO:0070665 | positive regulation of leukocyte proliferation | 23/955 | 1.53E-06 | 0.000108 | 23 |
| GO:0022409 | positive regulation of cell-cell adhesion | 34/955 | 1.54E-06 | 0.000108 | 34 |
| GO:0032649 | regulation of interferon-gamma production | 19/955 | 1.60E-06 | 0.00011 | 19 |
| GO:0002720 | positive regulation of cytokine production involved in immune response | 13/955 | 1.69E-06 | 0.000113 | 13 |
| GO:0043123 | positive regulation of I-kappaB kinase/NF-kappaB signaling | 26/955 | 1.69E-06 | 0.000113 | 26 |
| GO:0032479 | regulation of type I interferon production | 21/955 | 1.76E-06 | 0.000117 | 21 |
| GO:0032102 | negative regulation of response to external stimulus | 43/955 | 1.85E-06 | 0.000121 | 43 |
| GO:0032606 | type I interferon production | 21/955 | 2.01E-06 | 0.000129 | 21 |
| GO:0071222 | cellular response to lipopolysaccharide | 27/955 | 2.42E-06 | 0.000154 | 27 |
| GO:0071216 | cellular response to biotic stimulus | 30/955 | 2.54E-06 | 0.00016 | 30 |
| GO:0032480 | negative regulation of type I interferon production | 12/955 | 2.67E-06 | 0.000166 | 12 |
| GO:0001666 | response to hypoxia | 39/955 | 2.92E-06 | 0.000179 | 39 |
| GO:0050870 | positive regulation of T cell activation | 28/955 | 3.31E-06 | 0.000201 | 28 |
| GO:0050691 | regulation of defense response to virus by host | 11/955 | 4.12E-06 | 0.000247 | 11 |
| GO:0045229 | external encapsulating structure organization | 42/955 | 5.03E-06 | 0.000298 | 42 |
| GO:0002819 | regulation of adaptive immune response | 24/955 | 5.16E-06 | 0.000302 | 24 |
| GO:1901224 | positive regulation of NIK/NF-kappaB signaling | 14/955 | 5.25E-06 | 0.000304 | 14 |
| GO:2000116 | regulation of cysteine-type endopeptidase activity | 29/955 | 5.64E-06 | 0.000323 | 29 |
| GO:0070482 | response to oxygen levels | 41/955 | 5.88E-06 | 0.000333 | 41 |
| GO:0002699 | positive regulation of immune effector process | 28/955 | 6.23E-06 | 0.000349 | 28 |
| GO:0032760 | positive regulation of tumor necrosis factor production | 16/955 | 6.59E-06 | 0.000362 | 16 |
| GO:0036293 | response to decreased oxygen levels | 39/955 | 6.60E-06 | 0.000362 | 39 |
| GO:0034142 | toll-like receptor 4 signaling pathway | 11/955 | 6.83E-06 | 0.000371 | 11 |
| GO:0002703 | regulation of leukocyte mediated immunity | 27/955 | 7.48E-06 | 0.000397 | 27 |
| GO:0071219 | cellular response to molecule of bacterial origin | 27/955 | 7.48E-06 | 0.000397 | 27 |
| GO:0050688 | regulation of defense response to virus | 14/955 | 7.55E-06 | 0.000397 | 14 |
| GO:0009308 | amine metabolic process | 23/955 | 8.13E-06 | 0.000424 | 23 |
| GO:0001916 | positive regulation of T cell mediated cytotoxicity | 9/955 | 8.42E-06 | 0.000434 | 9 |
| GO:0002824 | positive regulation of adaptive immune response based on somatic recombination of immune receptors built from immunoglobulin superfamily domains | 17/955 | 8.96E-06 | 0.000457 | 17 |
| GO:0030198 | extracellular matrix organization | 41/955 | 9.71E-06 | 0.000491 | 41 |
| GO:0043062 | extracellular structure organization | 41/955 | 1.03E-05 | 0.000514 | 41 |
| GO:1903557 | positive regulation of tumor necrosis factor superfamily cytokine production | 16/955 | 1.04E-05 | 0.000514 | 16 |
| GO:0002822 | regulation of adaptive immune response based on somatic recombination of immune receptors built from immunoglobulin superfamily domains | 22/955 | 1.16E-05 | 0.000568 | 22 |
| GO:0140289 | protein mono-ADP-ribosylation | 6/955 | 1.18E-05 | 0.000573 | 6 |
| GO:0050729 | positive regulation of inflammatory response | 20/955 | 1.20E-05 | 0.000578 | 20 |
| GO:0001914 | regulation of T cell mediated cytotoxicity | 10/955 | 1.36E-05 | 0.000652 | 10 |
| GO:1903901 | negative regulation of viral life cycle | 8/955 | 1.49E-05 | 0.0007 | 8 |
| GO:0060326 | cell chemotaxis | 34/955 | 1.49E-05 | 0.0007 | 34 |
| GO:0002695 | negative regulation of leukocyte activation | 24/955 | 1.67E-05 | 0.000777 | 24 |
| GO:1905521 | regulation of macrophage migration | 10/955 | 1.75E-05 | 0.000795 | 10 |
| GO:0002456 | T cell mediated immunity | 17/955 | 1.76E-05 | 0.000795 | 17 |
| GO:0002821 | positive regulation of adaptive immune response | 17/955 | 1.76E-05 | 0.000795 | 17 |
| GO:0002718 | regulation of cytokine production involved in immune response | 15/955 | 1.84E-05 | 0.000825 | 15 |
| GO:0032729 | positive regulation of interferon-gamma production | 13/955 | 1.87E-05 | 0.000832 | 13 |
| GO:0046631 | alpha-beta T cell activation | 21/955 | 2.02E-05 | 0.000892 | 21 |
| GO:0001913 | T cell mediated cytotoxicity | 11/955 | 2.13E-05 | 0.00093 | 11 |
| GO:0009991 | response to extracellular stimulus | 46/955 | 2.19E-05 | 0.00095 | 46 |
| GO:0010038 | response to metal ion | 37/955 | 2.21E-05 | 0.00095 | 37 |
| GO:0032735 | positive regulation of interleukin-12 production | 10/955 | 2.23E-05 | 0.000951 | 10 |
| GO:0002460 | adaptive immune response based on somatic recombination of immune receptors built from immunoglobulin superfamily domains | 38/955 | 2.41E-05 | 0.001018 | 38 |
| GO:0002526 | acute inflammatory response | 17/955 | 2.57E-05 | 0.001079 | 17 |
| GO:0050730 | regulation of peptidyl-tyrosine phosphorylation | 30/955 | 2.64E-05 | 0.001097 | 30 |
| GO:0006898 | receptor-mediated endocytosis | 35/955 | 2.67E-05 | 0.0011 | 35 |
| GO:0018108 | peptidyl-tyrosine phosphorylation | 38/955 | 2.72E-05 | 0.001112 | 38 |
| GO:0045785 | positive regulation of cell adhesion | 42/955 | 2.80E-05 | 0.001134 | 42 |
| GO:0050866 | negative regulation of cell activation | 25/955 | 2.84E-05 | 0.001134 | 25 |
| GO:0006919 | activation of cysteine-type endopeptidase activity involved in apoptotic process | 15/955 | 2.84E-05 | 0.001134 | 15 |
| GO:0050867 | positive regulation of cell activation | 41/955 | 2.96E-05 | 0.001174 | 41 |
| GO:0030595 | leukocyte chemotaxis | 27/955 | 3.12E-05 | 0.001228 | 27 |
| GO:0002478 | antigen processing and presentation of exogenous peptide antigen | 23/955 | 3.16E-05 | 0.001234 | 23 |
| GO:0018212 | peptidyl-tyrosine modification | 38/955 | 3.25E-05 | 0.001258 | 38 |
| GO:0032663 | regulation of interleukin-2 production | 12/955 | 3.27E-05 | 0.001258 | 12 |
| GO:0002475 | antigen processing and presentation via MHC class Ib | 6/955 | 3.51E-05 | 0.00134 | 6 |
| GO:0048638 | regulation of developmental growth | 34/955 | 3.54E-05 | 0.00134 | 34 |
| GO:0042102 | positive regulation of T cell proliferation | 16/955 | 3.56E-05 | 0.00134 | 16 |
| GO:0046597 | negative regulation of viral entry into host cell | 7/955 | 3.62E-05 | 0.001352 | 7 |
| GO:0010758 | regulation of macrophage chemotaxis | 8/955 | 3.93E-05 | 0.001459 | 8 |
| GO:0045765 | regulation of angiogenesis | 35/955 | 4.14E-05 | 0.001526 | 35 |
| GO:0043281 | regulation of cysteine-type endopeptidase activity involved in apoptotic process | 25/955 | 4.30E-05 | 0.001573 | 25 |
| GO:0050731 | positive regulation of peptidyl-tyrosine phosphorylation | 24/955 | 4.43E-05 | 0.001609 | 24 |
| GO:0032623 | interleukin-2 production | 12/955 | 4.63E-05 | 0.001671 | 12 |
| GO:0071456 | cellular response to hypoxia | 25/955 | 4.66E-05 | 0.001671 | 25 |
| GO:0044106 | cellular amine metabolic process | 21/955 | 4.92E-05 | 0.00175 | 21 |
| GO:0002221 | pattern recognition receptor signaling pathway | 25/955 | 5.48E-05 | 0.001935 | 25 |
| GO:1904062 | regulation of cation transmembrane transport | 35/955 | 5.61E-05 | 0.00197 | 35 |
| GO:0002704 | negative regulation of leukocyte mediated immunity | 11/955 | 5.69E-05 | 0.001971 | 11 |
| GO:1905517 | macrophage migration | 11/955 | 5.69E-05 | 0.001971 | 11 |
| GO:0002705 | positive regulation of leukocyte mediated immunity | 18/955 | 5.81E-05 | 0.001999 | 18 |
| GO:1901342 | regulation of vasculature development | 35/955 | 5.96E-05 | 0.002036 | 35 |
| GO:0052548 | regulation of endopeptidase activity | 41/955 | 6.32E-05 | 0.002117 | 41 |
| GO:0002367 | cytokine production involved in immune response | 15/955 | 6.34E-05 | 0.002117 | 15 |
| GO:0019884 | antigen processing and presentation of exogenous antigen | 23/955 | 6.36E-05 | 0.002117 | 23 |
| GO:0038061 | NIK/NF-kappaB signaling | 23/955 | 6.36E-05 | 0.002117 | 23 |
| GO:0050673 | epithelial cell proliferation | 41/955 | 7.02E-05 | 0.002322 | 41 |
| GO:0062207 | regulation of pattern recognition receptor signaling pathway | 16/955 | 7.42E-05 | 0.002437 | 16 |
| GO:0002696 | positive regulation of leukocyte activation | 39/955 | 7.57E-05 | 0.002471 | 39 |
| GO:0036294 | cellular response to decreased oxygen levels | 25/955 | 8.73E-05 | 0.002831 | 25 |
| GO:0060759 | regulation of response to cytokine stimulus | 23/955 | 8.86E-05 | 0.002832 | 23 |
| GO:0071248 | cellular response to metal ion | 23/955 | 8.86E-05 | 0.002832 | 23 |
| GO:0070265 | necrotic cell death | 12/955 | 8.89E-05 | 0.002832 | 12 |
| GO:0042402 | cellular biogenic amine catabolic process | 8/955 | 9.05E-05 | 0.002865 | 8 |
| GO:2001257 | regulation of cation channel activity | 22/955 | 9.14E-05 | 0.002874 | 22 |
| GO:0009612 | response to mechanical stimulus | 24/955 | 9.20E-05 | 0.002875 | 24 |
| GO:0051250 | negative regulation of lymphocyte activation | 20/955 | 9.33E-05 | 0.002899 | 20 |
| GO:0046135 | pyrimidine nucleoside catabolic process | 7/955 | 0.0001 | 0.003091 | 7 |
| GO:0071241 | cellular response to inorganic substance | 25/955 | 0.000101 | 0.003115 | 25 |
| GO:0050868 | negative regulation of T cell activation | 17/955 | 0.000102 | 0.003122 | 17 |
| GO:0031667 | response to nutrient levels | 42/955 | 0.000108 | 0.003291 | 42 |
| GO:0002685 | regulation of leukocyte migration | 24/955 | 0.000116 | 0.00348 | 24 |
| GO:0002230 | positive regulation of defense response to virus by host | 8/955 | 0.000117 | 0.00348 | 8 |
| GO:0031341 | regulation of cell killing | 15/955 | 0.000117 | 0.00348 | 15 |
| GO:0097529 | myeloid leukocyte migration | 25/955 | 0.000118 | 0.00348 | 25 |
| GO:0071453 | cellular response to oxygen levels | 26/955 | 0.000118 | 0.00348 | 26 |
| GO:0002715 | regulation of natural killer cell mediated immunity | 10/955 | 0.000119 | 0.00348 | 10 |
| GO:0034121 | regulation of toll-like receptor signaling pathway | 13/955 | 0.000119 | 0.00348 | 13 |
| GO:0002224 | toll-like receptor signaling pathway | 20/955 | 0.000134 | 0.003832 | 20 |
| GO:0002706 | regulation of lymphocyte mediated immunity | 20/955 | 0.000134 | 0.003832 | 20 |
| GO:0060538 | skeletal muscle organ development | 20/955 | 0.000134 | 0.003832 | 20 |
| GO:0010950 | positive regulation of endopeptidase activity | 22/955 | 0.000138 | 0.003933 | 22 |
| GO:0070997 | neuron death | 34/955 | 0.000141 | 0.003985 | 34 |
| GO:0048732 | gland development | 39/955 | 0.000141 | 0.003985 | 39 |
| GO:0045444 | fat cell differentiation | 25/955 | 0.000146 | 0.0041 | 25 |
| GO:0009310 | amine catabolic process | 8/955 | 0.000149 | 0.004146 | 8 |
| GO:0001910 | regulation of leukocyte mediated cytotoxicity | 13/955 | 0.000156 | 0.004332 | 13 |
| GO:0043280 | positive regulation of cysteine-type endopeptidase activity involved in apoptotic process | 18/955 | 0.00016 | 0.004401 | 18 |
| GO:0071496 | cellular response to external stimulus | 31/955 | 0.000163 | 0.004483 | 31 |
| GO:0099590 | neurotransmitter receptor internalization | 7/955 | 0.00018 | 0.004887 | 7 |
| GO:0001909 | leukocyte mediated cytotoxicity | 16/955 | 0.00018 | 0.004887 | 16 |
| GO:0045953 | negative regulation of natural killer cell mediated cytotoxicity | 6/955 | 0.000182 | 0.004915 | 6 |
| GO:0032615 | interleukin-12 production | 11/955 | 0.000185 | 0.00495 | 11 |
| GO:0032655 | regulation of interleukin-12 production | 11/955 | 0.000185 | 0.00495 | 11 |
| GO:0002698 | negative regulation of immune effector process | 17/955 | 0.000189 | 0.005017 | 17 |
| GO:0002730 | regulation of dendritic cell cytokine production | 5/955 | 0.000193 | 0.005117 | 5 |
| GO:0097300 | programmed necrotic cell death | 10/955 | 0.000203 | 0.005339 | 10 |
| GO:0048705 | skeletal system morphogenesis | 24/955 | 0.000209 | 0.005474 | 24 |
| GO:1903038 | negative regulation of leukocyte cell-cell adhesion | 18/955 | 0.000211 | 0.0055 | 18 |
| GO:0045766 | positive regulation of angiogenesis | 21/955 | 0.000215 | 0.005541 | 21 |
| GO:1904018 | positive regulation of vasculature development | 21/955 | 0.000215 | 0.005541 | 21 |
| GO:2001056 | positive regulation of cysteine-type endopeptidase activity | 19/955 | 0.000229 | 0.005877 | 19 |
| GO:0006471 | protein ADP-ribosylation | 8/955 | 0.000235 | 0.00596 | 8 |
| GO:0009164 | nucleoside catabolic process | 8/955 | 0.000235 | 0.00596 | 8 |
| GO:0002711 | positive regulation of T cell mediated immunity | 10/955 | 0.00024 | 0.006066 | 10 |
| GO:0046718 | viral entry into host cell | 18/955 | 0.000253 | 0.006315 | 18 |
| GO:0002544 | chronic inflammatory response | 6/955 | 0.000255 | 0.006315 | 6 |
| GO:0002716 | negative regulation of natural killer cell mediated immunity | 6/955 | 0.000255 | 0.006315 | 6 |
| GO:0010893 | positive regulation of steroid biosynthetic process | 6/955 | 0.000255 | 0.006315 | 6 |
| GO:1903131 | mononuclear cell differentiation | 38/955 | 0.000262 | 0.006443 | 38 |
| GO:0052547 | regulation of peptidase activity | 41/955 | 0.000262 | 0.006443 | 41 |
| GO:0032412 | regulation of ion transmembrane transporter activity | 27/955 | 0.000276 | 0.006742 | 27 |
| GO:0031343 | positive regulation of cell killing | 11/955 | 0.00029 | 0.00705 | 11 |
| GO:0001906 | cell killing | 21/955 | 0.000294 | 0.007114 | 21 |
| GO:0051251 | positive regulation of lymphocyte activation | 34/955 | 0.0003 | 0.007195 | 34 |
| GO:0042269 | regulation of natural killer cell mediated cytotoxicity | 9/955 | 0.000301 | 0.007195 | 9 |
| GO:0002371 | dendritic cell cytokine production | 5/955 | 0.000301 | 0.007195 | 5 |
| GO:0071347 | cellular response to interleukin-1 | 21/955 | 0.000317 | 0.007537 | 21 |
| GO:0070555 | response to interleukin-1 | 23/955 | 0.000321 | 0.007588 | 23 |
| GO:0002688 | regulation of leukocyte chemotaxis | 16/955 | 0.000329 | 0.007753 | 16 |
| GO:0042267 | natural killer cell mediated cytotoxicity | 11/955 | 0.000334 | 0.007803 | 11 |
| GO:0043112 | receptor metabolic process | 20/955 | 0.000334 | 0.007803 | 20 |
| GO:0032816 | positive regulation of natural killer cell activation | 6/955 | 0.000348 | 0.008092 | 6 |
| GO:0002708 | positive regulation of lymphocyte mediated immunity | 15/955 | 0.000351 | 0.008117 | 15 |
| GO:0002707 | negative regulation of lymphocyte mediated immunity | 9/955 | 0.000359 | 0.00827 | 9 |
| GO:0070588 | calcium ion transmembrane transport | 30/955 | 0.000384 | 0.008797 | 30 |
| GO:0001912 | positive regulation of leukocyte mediated cytotoxicity | 10/955 | 0.000387 | 0.008797 | 10 |
| GO:0032703 | negative regulation of interleukin-2 production | 7/955 | 0.000387 | 0.008797 | 7 |
| GO:1903169 | regulation of calcium ion transmembrane transport | 18/955 | 0.000391 | 0.008854 | 18 |
| GO:0032675 | regulation of interleukin-6 production | 19/955 | 0.00041 | 0.009241 | 19 |
| GO:0045089 | positive regulation of innate immune response | 24/955 | 0.000415 | 0.009296 | 24 |
| GO:0006953 | acute-phase response | 9/955 | 0.000427 | 0.009487 | 9 |
| GO:0099637 | neurotransmitter receptor transport | 9/955 | 0.000427 | 0.009487 | 9 |
| GO:0046633 | alpha-beta T cell proliferation | 8/955 | 0.000436 | 0.00965 | 8 |
| GO:0051960 | regulation of nervous system development | 38/955 | 0.00044 | 0.009693 | 38 |
| GO:0050920 | regulation of chemotaxis | 24/955 | 0.000443 | 0.009714 | 24 |
| GO:1902187 | negative regulation of viral release from host cell | 5/955 | 0.000449 | 0.009817 | 5 |
| GO:0051924 | regulation of calcium ion transport | 25/955 | 0.000458 | 0.009965 | 25 |
| GO:0007519 | skeletal muscle tissue development | 18/955 | 0.000463 | 0.010029 | 18 |
| GO:0002053 | positive regulation of mesenchymal cell proliferation | 6/955 | 0.000467 | 0.010075 | 6 |
| GO:0060560 | developmental growth involved in morphogenesis | 24/955 | 0.000472 | 0.010149 | 24 |
| GO:0006816 | calcium ion transport | 37/955 | 0.000476 | 0.010198 | 37 |
| GO:0048511 | rhythmic process | 29/955 | 0.000481 | 0.010244 | 29 |
| GO:0030178 | negative regulation of Wnt signaling pathway | 23/955 | 0.000484 | 0.010266 | 23 |
| GO:0045940 | positive regulation of steroid metabolic process | 7/955 | 0.000488 | 0.010321 | 7 |
| GO:0097530 | granulocyte migration | 18/955 | 0.000502 | 0.010502 | 18 |
| GO:0002228 | natural killer cell mediated immunity | 11/955 | 0.000503 | 0.010502 | 11 |
| GO:0019226 | transmission of nerve impulse | 11/955 | 0.000503 | 0.010502 | 11 |
| GO:0032757 | positive regulation of interleukin-8 production | 10/955 | 0.000521 | 0.01074 | 10 |
| GO:0001570 | vasculogenesis | 12/955 | 0.000524 | 0.01074 | 12 |
| GO:0002709 | regulation of T cell mediated immunity | 12/955 | 0.000524 | 0.01074 | 12 |
| GO:0071621 | granulocyte chemotaxis | 16/955 | 0.000525 | 0.01074 | 16 |
| GO:0010952 | positive regulation of peptidase activity | 22/955 | 0.000526 | 0.01074 | 22 |
| GO:0048246 | macrophage chemotaxis | 8/955 | 0.000528 | 0.01074 | 8 |
| GO:0010721 | negative regulation of cell development | 20/955 | 0.000529 | 0.01074 | 20 |
| GO:0022898 | regulation of transmembrane transporter activity | 27/955 | 0.000535 | 0.010826 | 27 |
| GO:0050808 | synapse organization | 37/955 | 0.000547 | 0.011019 | 37 |
| GO:0032635 | interleukin-6 production | 19/955 | 0.000562 | 0.011274 | 19 |
| GO:0003002 | regionalization | 31/955 | 0.000584 | 0.011678 | 31 |
| GO:0001911 | negative regulation of leukocyte mediated cytotoxicity | 6/955 | 0.000615 | 0.012193 | 6 |
| GO:0035458 | cellular response to interferon-beta | 6/955 | 0.000615 | 0.012193 | 6 |
| GO:0072075 | metanephric mesenchyme development | 5/955 | 0.000646 | 0.012763 | 5 |
| GO:0001959 | regulation of cytokine-mediated signaling pathway | 20/955 | 0.000659 | 0.012965 | 20 |
| GO:0002833 | positive regulation of response to biotic stimulus | 26/955 | 0.00067 | 0.013132 | 26 |
| GO:0006576 | cellular biogenic amine metabolic process | 14/955 | 0.000681 | 0.013252 | 14 |
| GO:0030593 | neutrophil chemotaxis | 14/955 | 0.000681 | 0.013252 | 14 |
| GO:0002573 | myeloid leukocyte differentiation | 22/955 | 0.000689 | 0.013355 | 22 |
| GO:0071622 | regulation of granulocyte chemotaxis | 9/955 | 0.000695 | 0.013418 | 9 |
| GO:0050678 | regulation of epithelial cell proliferation | 34/955 | 0.000728 | 0.014016 | 34 |
| GO:0044409 | entry into host | 18/955 | 0.000748 | 0.014346 | 18 |
| GO:0032196 | transposition | 7/955 | 0.000753 | 0.014385 | 7 |
| GO:0032874 | positive regulation of stress-activated MAPK cascade | 15/955 | 0.000758 | 0.014426 | 15 |
| GO:1904064 | positive regulation of cation transmembrane transport | 17/955 | 0.000786 | 0.014899 | 17 |
| GO:0010959 | regulation of metal ion transport | 25/955 | 0.00079 | 0.014919 | 25 |
| GO:0045576 | mast cell activation | 10/955 | 0.000793 | 0.014919 | 10 |
| GO:0051403 | stress-activated MAPK cascade | 26/955 | 0.000795 | 0.014919 | 26 |
| GO:0034656 | nucleobase-containing small molecule catabolic process | 9/955 | 0.00081 | 0.015129 | 9 |
| GO:0001558 | regulation of cell growth | 36/955 | 0.000814 | 0.01515 | 36 |
| GO:0007568 | aging | 29/955 | 0.000823 | 0.015262 | 29 |
| GO:0033003 | regulation of mast cell activation | 8/955 | 0.000902 | 0.016672 | 8 |
| GO:0070304 | positive regulation of stress-activated protein kinase signaling cascade | 15/955 | 0.000907 | 0.01669 | 15 |
| GO:1901222 | regulation of NIK/NF-kappaB signaling | 14/955 | 0.000909 | 0.01669 | 14 |
| GO:0055074 | calcium ion homeostasis | 39/955 | 0.000923 | 0.016881 | 39 |
| GO:1901019 | regulation of calcium ion transmembrane transporter activity | 12/955 | 0.000928 | 0.016915 | 12 |
| GO:0016049 | cell growth | 40/955 | 0.000954 | 0.017329 | 40 |
| GO:0030001 | metal ion transport | 41/955 | 0.000983 | 0.017786 | 41 |
| GO:1902105 | regulation of leukocyte differentiation | 27/955 | 0.000988 | 0.017821 | 27 |
| GO:0046632 | alpha-beta T cell differentiation | 14/955 | 0.000998 | 0.017882 | 14 |
| GO:0048640 | negative regulation of developmental growth | 14/955 | 0.000998 | 0.017882 | 14 |
| GO:0007389 | pattern specification process | 37/955 | 0.001012 | 0.018018 | 37 |
| GO:0010001 | glial cell differentiation | 22/955 | 0.001014 | 0.018018 | 22 |
| GO:1905523 | positive regulation of macrophage migration | 6/955 | 0.001017 | 0.018018 | 6 |
| GO:0006874 | cellular calcium ion homeostasis | 38/955 | 0.001051 | 0.018565 | 38 |
| GO:0048568 | embryonic organ development | 36/955 | 0.001057 | 0.018615 | 36 |
| GO:0062208 | positive regulation of pattern recognition receptor signaling pathway | 8/955 | 0.001065 | 0.018629 | 8 |
| GO:0032817 | regulation of natural killer cell proliferation | 4/955 | 0.001073 | 0.018629 | 4 |
| GO:0043301 | negative regulation of leukocyte degranulation | 4/955 | 0.001073 | 0.018629 | 4 |
| GO:0070383 | DNA cytosine deamination | 4/955 | 0.001073 | 0.018629 | 4 |
| GO:0032814 | regulation of natural killer cell activation | 7/955 | 0.00112 | 0.019385 | 7 |
| GO:0050768 | negative regulation of neurogenesis | 16/955 | 0.001134 | 0.019557 | 16 |
| GO:0007623 | circadian rhythm | 22/955 | 0.001148 | 0.019697 | 22 |
| GO:0046889 | positive regulation of lipid biosynthetic process | 12/955 | 0.001149 | 0.019697 | 12 |
| GO:0002700 | regulation of production of molecular mediator of immune response | 17/955 | 0.001163 | 0.01987 | 17 |
| GO:0071260 | cellular response to mechanical stimulus | 11/955 | 0.001186 | 0.020184 | 11 |
| GO:1903706 | regulation of hemopoiesis | 36/955 | 0.001202 | 0.02039 | 36 |
| GO:0032409 | regulation of transporter activity | 27/955 | 0.001221 | 0.020544 | 27 |
| GO:0010759 | positive regulation of macrophage chemotaxis | 5/955 | 0.001223 | 0.020544 | 5 |
| GO:0071850 | mitotic cell cycle arrest | 5/955 | 0.001223 | 0.020544 | 5 |
| GO:0032620 | interleukin-17 production | 8/955 | 0.001252 | 0.020891 | 8 |
| GO:0070266 | necroptotic process | 8/955 | 0.001252 | 0.020891 | 8 |
| GO:0035270 | endocrine system development | 15/955 | 0.00128 | 0.02104 | 15 |
| GO:0001562 | response to protozoan | 6/955 | 0.001281 | 0.02104 | 6 |
| GO:0031664 | regulation of lipopolysaccharide-mediated signaling pathway | 6/955 | 0.001281 | 0.02104 | 6 |
| GO:0042537 | benzene-containing compound metabolic process | 6/955 | 0.001281 | 0.02104 | 6 |
| GO:0042832 | defense response to protozoan | 6/955 | 0.001281 | 0.02104 | 6 |
| GO:0050830 | defense response to Gram-positive bacterium | 13/955 | 0.001313 | 0.021507 | 13 |
| GO:0046640 | regulation of alpha-beta T cell proliferation | 7/955 | 0.001349 | 0.022027 | 7 |
| GO:1990266 | neutrophil migration | 15/955 | 0.001391 | 0.022636 | 15 |
| GO:0072503 | cellular divalent inorganic cation homeostasis | 40/955 | 0.001416 | 0.022961 | 40 |
| GO:0052126 | movement in host environment | 19/955 | 0.001431 | 0.023087 | 19 |
| GO:0031623 | receptor internalization | 14/955 | 0.001432 | 0.023087 | 14 |
| GO:0002702 | positive regulation of production of molecular mediator of immune response | 13/955 | 0.001443 | 0.023194 | 13 |
| GO:1901658 | glycosyl compound catabolic process | 8/955 | 0.001463 | 0.023433 | 8 |
| GO:0051279 | regulation of release of sequestered calcium ion into cytosol | 11/955 | 0.001482 | 0.02367 | 11 |
| GO:0022408 | negative regulation of cell-cell adhesion | 20/955 | 0.001489 | 0.023705 | 20 |
| GO:1901214 | regulation of neuron death | 28/955 | 0.00154 | 0.024447 | 28 |
| GO:0045926 | negative regulation of growth | 24/955 | 0.00156 | 0.024623 | 24 |
| GO:0062012 | regulation of small molecule metabolic process | 37/955 | 0.001592 | 0.024623 | 37 |
| GO:0010464 | regulation of mesenchymal cell proliferation | 6/955 | 0.001595 | 0.024623 | 6 |
| GO:0010528 | regulation of transposition | 6/955 | 0.001595 | 0.024623 | 6 |
| GO:0010529 | negative regulation of transposition | 6/955 | 0.001595 | 0.024623 | 6 |
| GO:0031342 | negative regulation of cell killing | 6/955 | 0.001595 | 0.024623 | 6 |
| GO:2000311 | regulation of AMPA receptor activity | 6/955 | 0.001595 | 0.024623 | 6 |
| GO:0001787 | natural killer cell proliferation | 4/955 | 0.001618 | 0.024623 | 4 |
| GO:0070189 | kynurenine metabolic process | 4/955 | 0.001618 | 0.024623 | 4 |
| GO:0070213 | protein auto-ADP-ribosylation | 4/955 | 0.001618 | 0.024623 | 4 |
| GO:2000644 | regulation of receptor catabolic process | 4/955 | 0.001618 | 0.024623 | 4 |
| GO:0072507 | divalent inorganic cation homeostasis | 41/955 | 0.001619 | 0.024623 | 41 |
| GO:0032740 | positive regulation of interleukin-17 production | 5/955 | 0.001623 | 0.024623 | 5 |
| GO:0098884 | postsynaptic neurotransmitter receptor internalization | 5/955 | 0.001623 | 0.024623 | 5 |
| GO:0140239 | postsynaptic endocytosis | 5/955 | 0.001623 | 0.024623 | 5 |
| GO:0030217 | T cell differentiation | 24/955 | 0.001648 | 0.024912 | 24 |
| GO:0033238 | regulation of cellular amine metabolic process | 11/955 | 0.001652 | 0.024912 | 11 |
| GO:1990138 | neuron projection extension | 18/955 | 0.00167 | 0.025107 | 18 |
| GO:0051961 | negative regulation of nervous system development | 16/955 | 0.001677 | 0.025139 | 16 |
| GO:0042130 | negative regulation of T cell proliferation | 10/955 | 0.001688 | 0.025169 | 10 |
| GO:0046635 | positive regulation of alpha-beta T cell activation | 10/955 | 0.001688 | 0.025169 | 10 |
| GO:0003009 | skeletal muscle contraction | 8/955 | 0.001701 | 0.025292 | 8 |
| GO:0046634 | regulation of alpha-beta T cell activation | 13/955 | 0.001736 | 0.025726 | 13 |
| GO:0032612 | interleukin-1 production | 15/955 | 0.001774 | 0.026224 | 15 |
| GO:0031098 | stress-activated protein kinase signaling cascade | 26/955 | 0.001782 | 0.026266 | 26 |
| GO:0140029 | exocytic process | 11/955 | 0.001837 | 0.026993 | 11 |
| GO:0090090 | negative regulation of canonical Wnt signaling pathway | 19/955 | 0.001863 | 0.027279 | 19 |
| GO:0060760 | positive regulation of response to cytokine stimulus | 9/955 | 0.001867 | 0.027279 | 9 |
| GO:0032922 | circadian regulation of gene expression | 10/955 | 0.001896 | 0.027631 | 10 |
| GO:0006213 | pyrimidine nucleoside metabolic process | 7/955 | 0.001918 | 0.027783 | 7 |
| GO:0097484 | dendrite extension | 7/955 | 0.001918 | 0.027783 | 7 |
| GO:0034764 | positive regulation of transmembrane transport | 21/955 | 0.001946 | 0.028035 | 21 |
| GO:0034767 | positive regulation of ion transmembrane transport | 21/955 | 0.001946 | 0.028035 | 21 |
| GO:0071624 | positive regulation of granulocyte chemotaxis | 6/955 | 0.001963 | 0.028132 | 6 |
| GO:0090200 | positive regulation of release of cytochrome c from mitochondria | 6/955 | 0.001963 | 0.028132 | 6 |
| GO:0046434 | organophosphate catabolic process | 17/955 | 0.002084 | 0.029762 | 17 |
| GO:0002690 | positive regulation of leukocyte chemotaxis | 12/955 | 0.00209 | 0.029762 | 12 |
| GO:0062013 | positive regulation of small molecule metabolic process | 16/955 | 0.002096 | 0.029762 | 16 |
| GO:0062099 | negative regulation of programmed necrotic cell death | 5/955 | 0.002112 | 0.029762 | 5 |
| GO:0072074 | kidney mesenchyme development | 5/955 | 0.002112 | 0.029762 | 5 |
| GO:0098543 | detection of other organism | 5/955 | 0.002112 | 0.029762 | 5 |
| GO:0051651 | maintenance of location in cell | 21/955 | 0.002188 | 0.030755 | 21 |
| GO:0032088 | negative regulation of NF-kappaB transcription factor activity | 12/955 | 0.002296 | 0.032176 | 12 |
| GO:0006216 | cytidine catabolic process | 4/955 | 0.002331 | 0.03223 | 4 |
| GO:0009972 | cytidine deamination | 4/955 | 0.002331 | 0.03223 | 4 |
| GO:0016554 | cytidine to uridine editing | 4/955 | 0.002331 | 0.03223 | 4 |
| GO:0046087 | cytidine metabolic process | 4/955 | 0.002331 | 0.03223 | 4 |
| GO:2001214 | positive regulation of vasculogenesis | 4/955 | 0.002331 | 0.03223 | 4 |
| GO:0001503 | ossification | 34/955 | 0.002357 | 0.032511 | 34 |
| GO:0042531 | positive regulation of tyrosine phosphorylation of STAT protein | 10/955 | 0.002374 | 0.032637 | 10 |
| GO:0002507 | tolerance induction | 6/955 | 0.002393 | 0.032637 | 6 |
| GO:0007271 | synaptic transmission cholinergic | cholinergic | 0.002393 | 0.032637 | 6 |
| GO:0010575 | positive regulation of vascular endothelial growth factor production | 6/955 | 0.002393 | 0.032637 | 6 |
| GO:0031663 | lipopolysaccharide-mediated signaling pathway | 9/955 | 0.002398 | 0.032637 | 9 |
| GO:0099565 | chemical synaptic transmission postsynaptic | postsynaptic | 0.002467 | 0.033492 | 13 |
| GO:0042180 | cellular ketone metabolic process | 24/955 | 0.002519 | 0.034065 | 24 |
| GO:0042063 | gliogenesis | 26/955 | 0.002523 | 0.034065 | 26 |
| GO:0019722 | calcium-mediated signaling | 20/955 | 0.002589 | 0.034869 | 20 |
| GO:0030099 | myeloid cell differentiation | 35/955 | 0.002622 | 0.035206 | 35 |
| GO:0009896 | positive regulation of catabolic process | 37/955 | 0.002634 | 0.035206 | 37 |
| GO:0051145 | smooth muscle cell differentiation | 10/955 | 0.002647 | 0.035206 | 10 |
| GO:1904427 | positive regulation of calcium ion transmembrane transport | 10/955 | 0.002647 | 0.035206 | 10 |
| GO:0009595 | detection of biotic stimulus | 7/955 | 0.002655 | 0.035206 | 7 |
| GO:0010463 | mesenchymal cell proliferation | 7/955 | 0.002655 | 0.035206 | 7 |
| GO:0002577 | regulation of antigen processing and presentation | 5/955 | 0.0027 | 0.035274 | 5 |
| GO:0010226 | response to lithium ion | 5/955 | 0.0027 | 0.035274 | 5 |
| GO:0016553 | base conversion or substitution editing | 5/955 | 0.0027 | 0.035274 | 5 |
| GO:0043901 | negative regulation of multi-organism process | 5/955 | 0.0027 | 0.035274 | 5 |
| GO:0044342 | type B pancreatic cell proliferation | 5/955 | 0.0027 | 0.035274 | 5 |
| GO:1902041 | regulation of extrinsic apoptotic signaling pathway via death domain receptors | 9/955 | 0.002705 | 0.035274 | 9 |
| GO:0009749 | response to glucose | 19/955 | 0.002715 | 0.035274 | 19 |
| GO:0010565 | regulation of cellular ketone metabolic process | 19/955 | 0.002715 | 0.035274 | 19 |
| GO:0050804 | modulation of chemical synaptic transmission | 34/955 | 0.002765 | 0.035831 | 34 |
| GO:0032652 | regulation of interleukin-1 production | 14/955 | 0.002777 | 0.035899 | 14 |
| GO:0001764 | neuron migration | 16/955 | 0.00279 | 0.035971 | 16 |
| GO:0099177 | regulation of trans-synaptic signaling | 34/955 | 0.002876 | 0.036988 | 34 |
| GO:0098586 | cellular response to virus | 10/955 | 0.002944 | 0.037778 | 10 |
| GO:0038066 | p38MAPK cascade | 8/955 | 0.002979 | 0.038126 | 8 |
| GO:0010522 | regulation of calcium ion transport into cytosol | 12/955 | 0.003012 | 0.038461 | 12 |
| GO:0002687 | positive regulation of leukocyte migration | 15/955 | 0.00302 | 0.038462 | 15 |
| GO:0099072 | regulation of postsynaptic membrane neurotransmitter receptor levels | 9/955 | 0.003043 | 0.038656 | 9 |
| GO:0032660 | regulation of interleukin-17 production | 7/955 | 0.003097 | 0.039249 | 7 |
| GO:0007517 | muscle organ development | 28/955 | 0.003106 | 0.039269 | 28 |
| GO:0001501 | skeletal system development | 39/955 | 0.003134 | 0.039518 | 39 |
| GO:0002923 | regulation of humoral immune response mediated by circulating immunoglobulin | 4/955 | 0.003233 | 0.04057 | 4 |
| GO:0031665 | negative regulation of lipopolysaccharide-mediated signaling pathway | 4/955 | 0.003233 | 0.04057 | 4 |
| GO:0097553 | calcium ion transmembrane import into cytosol | 15/955 | 0.003246 | 0.040636 | 15 |
| GO:0008016 | regulation of heart contraction | 23/955 | 0.00332 | 0.041462 | 23 |
| GO:0051235 | maintenance of location | 28/955 | 0.003393 | 0.041809 | 28 |
| GO:0031629 | synaptic vesicle fusion to presynaptic active zone membrane | 5/955 | 0.003397 | 0.041809 | 5 |
| GO:0046641 | positive regulation of alpha-beta T cell proliferation | 5/955 | 0.003397 | 0.041809 | 5 |
| GO:0072574 | hepatocyte proliferation | 5/955 | 0.003397 | 0.041809 | 5 |
| GO:0072575 | epithelial cell proliferation involved in liver morphogenesis | 5/955 | 0.003397 | 0.041809 | 5 |
| GO:0099500 | vesicle fusion to plasma membrane | 5/955 | 0.003397 | 0.041809 | 5 |
| GO:0070059 | intrinsic apoptotic signaling pathway in response to endoplasmic reticulum stress | 9/955 | 0.003412 | 0.041809 | 9 |
| GO:1905330 | regulation of morphogenesis of an epithelium | 9/955 | 0.003412 | 0.041809 | 9 |
| GO:0043618 | regulation of transcription from RNA polymerase II promoter in response to stress | 13/955 | 0.003435 | 0.041982 | 13 |
| GO:0035637 | multicellular organismal signaling | 20/955 | 0.003452 | 0.042095 | 20 |
| GO:0051968 | positive regulation of synaptic transmission glutamatergic | glutamatergic | 0.003461 | 0.042099 | 6 |
| GO:0030111 | regulation of Wnt signaling pathway | 31/955 | 0.003494 | 0.042401 | 31 |
| GO:0051281 | positive regulation of release of sequestered calcium ion into cytosol | 7/955 | 0.003593 | 0.043402 | 7 |
| GO:0072529 | pyrimidine-containing compound catabolic process | 7/955 | 0.003593 | 0.043402 | 7 |
| GO:0009166 | nucleotide catabolic process | 10/955 | 0.003619 | 0.043545 | 10 |
| GO:2000027 | regulation of animal organ morphogenesis | 18/955 | 0.003622 | 0.043545 | 18 |
| GO:0050852 | T cell receptor signaling pathway | 20/955 | 0.003651 | 0.04368 | 20 |
| GO:0009746 | response to hexose | 19/955 | 0.003654 | 0.04368 | 19 |
| GO:0042509 | regulation of tyrosine phosphorylation of STAT protein | 11/955 | 0.003658 | 0.04368 | 11 |
| GO:0060078 | regulation of postsynaptic membrane potential | 15/955 | 0.003739 | 0.044542 | 15 |
| GO:0032613 | interleukin-10 production | 9/955 | 0.003817 | 0.045274 | 9 |
| GO:0051701 | biological process involved in interaction with host | 21/955 | 0.003818 | 0.045274 | 21 |
| GO:0043900 | regulation of multi-organism process | 8/955 | 0.003848 | 0.045527 | 8 |
| GO:0032755 | positive regulation of interleukin-6 production | 11/955 | 0.004008 | 0.047305 | 11 |
| GO:0042391 | regulation of membrane potential | 35/955 | 0.004121 | 0.048526 | 35 |
| GO:0010939 | regulation of necrotic cell death | 7/955 | 0.004147 | 0.048725 | 7 |
| GO:0030878 | thyroid gland development | 5/955 | 0.004216 | 0.048976 | 5 |
| GO:0043457 | regulation of cellular respiration | 5/955 | 0.004216 | 0.048976 | 5 |
| GO:0060575 | intestinal epithelial cell differentiation | 5/955 | 0.004216 | 0.048976 | 5 |
| GO:0072576 | liver morphogenesis | 5/955 | 0.004216 | 0.048976 | 5 |
| GO:1900543 | negative regulation of purine nucleotide metabolic process | 5/955 | 0.004216 | 0.048976 | 5 |
| GO:0007584 | response to nutrient | 17/955 | 0.004281 | 0.049537 | 17 |
| GO:0072006 | nephron development | 15/955 | 0.004292 | 0.049537 | 15 |
| GO:0002467 | germinal center formation | 4/955 | 0.004346 | 0.049537 | 4 |
| GO:0030213 | hyaluronan biosynthetic process | 4/955 | 0.004346 | 0.049537 | 4 |
| GO:0045006 | DNA deamination | 4/955 | 0.004346 | 0.049537 | 4 |
| GO:0045820 | negative regulation of glycolytic process | 4/955 | 0.004346 | 0.049537 | 4 |
| GO:0046131 | pyrimidine ribonucleoside metabolic process | 4/955 | 0.004346 | 0.049537 | 4 |
| GO:0046133 | pyrimidine ribonucleoside catabolic process | 4/955 | 0.004346 | 0.049537 | 4 |
| GO:0050819 | negative regulation of coagulation | 8/955 | 0.004351 | 0.049537 | 8 |

**Supplementary Table 10. The GO-BP enrichment of down-regulated DEGs in U-251 MG cells of 24h**

| ID | Description | GeneRatio | pvalue | p.adjust | Count |
| --- | --- | --- | --- | --- | --- |
| GO:0070268 | cornification | 7/125 | 1.02E-05 | 0.020041 | 7 |

**Supplementary Table 11. The GO-BP enrichment of DEGs in HK-2 cells of 24h**

| ID | Description | GeneRatio | pvalue | p.adjust | Count |
| --- | --- | --- | --- | --- | --- |
| GO:0009615 | response to virus | 74/1207 | 1.34E-19 | 7.35E-16 | 74 |
| GO:0060337 | type I interferon signaling pathway | 35/1207 | 2.92E-18 | 5.68E-15 | 35 |
| GO:0034340 | response to type I interferon | 36/1207 | 3.40E-18 | 5.68E-15 | 36 |
| GO:0071357 | cellular response to type I interferon | 35/1207 | 4.31E-18 | 5.68E-15 | 35 |
| GO:0051607 | defense response to virus | 59/1207 | 6.22E-18 | 5.68E-15 | 59 |
| GO:0140546 | defense response to symbiont | 59/1207 | 6.22E-18 | 5.68E-15 | 59 |
| GO:0034341 | response to interferon-gamma | 44/1207 | 1.88E-13 | 1.47E-10 | 44 |
| GO:0048525 | negative regulation of viral process | 28/1207 | 6.62E-13 | 4.54E-10 | 28 |
| GO:0003018 | vascular process in circulatory system | 48/1207 | 2.60E-12 | 1.59E-09 | 48 |
| GO:0071346 | cellular response to interferon-gamma | 38/1207 | 2.94E-11 | 1.61E-08 | 38 |
| GO:0050804 | modulation of chemical synaptic transmission | 63/1207 | 4.46E-11 | 2.05E-08 | 63 |
| GO:0045071 | negative regulation of viral genome replication | 20/1207 | 4.50E-11 | 2.05E-08 | 20 |
| GO:0099177 | regulation of trans-synaptic signaling | 63/1207 | 4.96E-11 | 2.09E-08 | 63 |
| GO:1903900 | regulation of viral life cycle | 33/1207 | 2.04E-10 | 7.99E-08 | 33 |
| GO:0060333 | interferon-gamma-mediated signaling pathway | 25/1207 | 2.99E-10 | 1.09E-07 | 25 |
| GO:0010232 | vascular transport | 24/1207 | 7.96E-10 | 2.73E-07 | 24 |
| GO:0150104 | transport across blood-brain barrier | 23/1207 | 3.47E-09 | 1.12E-06 | 23 |
| GO:0045069 | regulation of viral genome replication | 22/1207 | 7.19E-09 | 2.19E-06 | 22 |
| GO:0060326 | cell chemotaxis | 47/1207 | 1.98E-08 | 5.73E-06 | 47 |
| GO:0050792 | regulation of viral process | 34/1207 | 2.49E-08 | 6.84E-06 | 34 |
| GO:0034612 | response to tumor necrosis factor | 48/1207 | 3.01E-08 | 7.86E-06 | 48 |
| GO:0043903 | regulation of biological process involved in symbiotic interaction | 35/1207 | 3.31E-08 | 8.23E-06 | 35 |
| GO:0001819 | positive regulation of cytokine production | 59/1207 | 4.17E-08 | 9.94E-06 | 59 |
| GO:0035633 | maintenance of blood-brain barrier | 13/1207 | 4.86E-08 | 1.11E-05 | 13 |
| GO:0002237 | response to molecule of bacterial origin | 50/1207 | 5.29E-08 | 1.14E-05 | 50 |
| GO:0032496 | response to lipopolysaccharide | 48/1207 | 5.43E-08 | 1.14E-05 | 48 |
| GO:0009612 | response to mechanical stimulus | 35/1207 | 6.34E-08 | 1.29E-05 | 35 |
| GO:0072507 | divalent inorganic cation homeostasis | 64/1207 | 7.88E-08 | 1.54E-05 | 64 |
| GO:0097191 | extrinsic apoptotic signaling pathway | 36/1207 | 1.28E-07 | 2.42E-05 | 36 |
| GO:0007411 | axon guidance | 42/1207 | 1.61E-07 | 2.94E-05 | 42 |
| GO:0097485 | neuron projection guidance | 42/1207 | 1.78E-07 | 3.15E-05 | 42 |
| GO:0035455 | response to interferon-alpha | 10/1207 | 2.04E-07 | 3.50E-05 | 10 |
| GO:0072503 | cellular divalent inorganic cation homeostasis | 61/1207 | 2.20E-07 | 3.65E-05 | 61 |
| GO:0015849 | organic acid transport | 46/1207 | 3.03E-07 | 4.88E-05 | 46 |
| GO:0016126 | sterol biosynthetic process | 19/1207 | 3.23E-07 | 5.05E-05 | 19 |
| GO:0050806 | positive regulation of synaptic transmission | 27/1207 | 3.96E-07 | 6.02E-05 | 27 |
| GO:0019079 | viral genome replication | 25/1207 | 5.53E-07 | 8.20E-05 | 25 |
| GO:0051480 | regulation of cytosolic calcium ion concentration | 47/1207 | 6.99E-07 | 0.000101 | 47 |
| GO:0070588 | calcium ion transmembrane transport | 43/1207 | 8.10E-07 | 0.000112 | 43 |
| GO:0055074 | calcium ion homeostasis | 57/1207 | 8.15E-07 | 0.000112 | 57 |
| GO:0050777 | negative regulation of immune response | 27/1207 | 9.18E-07 | 0.000123 | 27 |
| GO:0071621 | granulocyte chemotaxis | 24/1207 | 9.50E-07 | 0.000124 | 24 |
| GO:0071356 | cellular response to tumor necrosis factor | 42/1207 | 9.97E-07 | 0.000127 | 42 |
| GO:0048660 | regulation of smooth muscle cell proliferation | 28/1207 | 1.05E-06 | 0.00013 | 28 |
| GO:1901342 | regulation of vasculature development | 46/1207 | 1.31E-06 | 0.00016 | 46 |
| GO:0048659 | smooth muscle cell proliferation | 28/1207 | 1.35E-06 | 0.000161 | 28 |
| GO:0043123 | positive regulation of I-kappaB kinase/NF-kappaB signaling | 30/1207 | 1.61E-06 | 0.000188 | 30 |
| GO:0006874 | cellular calcium ion homeostasis | 55/1207 | 1.68E-06 | 0.000191 | 55 |
| GO:0006695 | cholesterol biosynthetic process | 17/1207 | 2.13E-06 | 0.000233 | 17 |
| GO:1902653 | secondary alcohol biosynthetic process | 17/1207 | 2.13E-06 | 0.000233 | 17 |
| GO:0046942 | carboxylic acid transport | 40/1207 | 2.17E-06 | 0.000233 | 40 |
| GO:0043122 | regulation of I-kappaB kinase/NF-kappaB signaling | 36/1207 | 2.33E-06 | 0.000243 | 36 |
| GO:0045824 | negative regulation of innate immune response | 16/1207 | 2.35E-06 | 0.000243 | 16 |
| GO:0140353 | lipid export from cell | 18/1207 | 2.75E-06 | 0.000277 | 18 |
| GO:0046849 | bone remodeling | 19/1207 | 2.78E-06 | 0.000277 | 19 |
| GO:0050673 | epithelial cell proliferation | 53/1207 | 2.98E-06 | 0.000292 | 53 |
| GO:0007204 | positive regulation of cytosolic calcium ion concentration | 42/1207 | 3.35E-06 | 0.000321 | 42 |
| GO:0048265 | response to pain | 10/1207 | 3.43E-06 | 0.000321 | 10 |
| GO:0097529 | myeloid leukocyte migration | 33/1207 | 3.46E-06 | 0.000321 | 33 |
| GO:0033555 | multicellular organismal response to stress | 16/1207 | 3.60E-06 | 0.000329 | 16 |
| GO:0019885 | antigen processing and presentation of endogenous peptide antigen via MHC class I | 8/1207 | 3.97E-06 | 0.000356 | 8 |
| GO:0001935 | endothelial cell proliferation | 30/1207 | 4.45E-06 | 0.000388 | 30 |
| GO:0045765 | regulation of angiogenesis | 44/1207 | 4.51E-06 | 0.000388 | 44 |
| GO:1901617 | organic hydroxy compound biosynthetic process | 36/1207 | 4.53E-06 | 0.000388 | 36 |
| GO:0002683 | negative regulation of immune system process | 50/1207 | 5.35E-06 | 0.000451 | 50 |
| GO:0007409 | axonogenesis | 55/1207 | 5.64E-06 | 0.000469 | 55 |
| GO:0036293 | response to decreased oxygen levels | 46/1207 | 5.80E-06 | 0.000474 | 46 |
| GO:0016125 | sterol metabolic process | 27/1207 | 6.06E-06 | 0.000488 | 27 |
| GO:0045540 | regulation of cholesterol biosynthetic process | 13/1207 | 6.64E-06 | 0.00052 | 13 |
| GO:0106118 | regulation of sterol biosynthetic process | 13/1207 | 6.64E-06 | 0.00052 | 13 |
| GO:0030595 | leukocyte chemotaxis | 33/1207 | 7.60E-06 | 0.000587 | 33 |
| GO:0002831 | regulation of response to biotic stimulus | 51/1207 | 7.96E-06 | 0.000591 | 51 |
| GO:0001936 | regulation of endothelial cell proliferation | 28/1207 | 7.98E-06 | 0.000591 | 28 |
| GO:0008217 | regulation of blood pressure | 28/1207 | 7.98E-06 | 0.000591 | 28 |
| GO:0006816 | calcium ion transport | 50/1207 | 8.09E-06 | 0.000591 | 50 |
| GO:0015711 | organic anion transport | 47/1207 | 8.33E-06 | 0.000601 | 47 |
| GO:1903522 | regulation of blood circulation | 39/1207 | 8.87E-06 | 0.000631 | 39 |
| GO:0032753 | positive regulation of interleukin-4 production | 9/1207 | 9.49E-06 | 0.000659 | 9 |
| GO:1905523 | positive regulation of macrophage migration | 9/1207 | 9.49E-06 | 0.000659 | 9 |
| GO:0002483 | antigen processing and presentation of endogenous peptide antigen | 8/1207 | 1.10E-05 | 0.000749 | 8 |
| GO:0098657 | import into cell | 33/1207 | 1.11E-05 | 0.000749 | 33 |
| GO:0070663 | regulation of leukocyte proliferation | 34/1207 | 1.18E-05 | 0.000791 | 34 |
| GO:0046850 | regulation of bone remodeling | 13/1207 | 1.37E-05 | 0.000904 | 13 |
| GO:0035456 | response to interferon-beta | 10/1207 | 1.42E-05 | 0.000929 | 10 |
| GO:0023061 | signal release | 55/1207 | 1.44E-05 | 0.000931 | 55 |
| GO:2001236 | regulation of extrinsic apoptotic signaling pathway | 25/1207 | 1.52E-05 | 0.00097 | 25 |
| GO:0070482 | response to oxygen levels | 47/1207 | 1.56E-05 | 0.00097 | 47 |
| GO:1902041 | regulation of extrinsic apoptotic signaling pathway via death domain receptors | 14/1207 | 1.57E-05 | 0.00097 | 14 |
| GO:0050678 | regulation of epithelial cell proliferation | 46/1207 | 1.58E-05 | 0.00097 | 46 |
| GO:0002685 | regulation of leukocyte migration | 30/1207 | 1.86E-05 | 0.001132 | 30 |
| GO:0046165 | alcohol biosynthetic process | 25/1207 | 1.91E-05 | 0.001152 | 25 |
| GO:0032673 | regulation of interleukin-4 production | 10/1207 | 1.95E-05 | 0.001163 | 10 |
| GO:0048167 | regulation of synaptic plasticity | 27/1207 | 2.04E-05 | 0.0012 | 27 |
| GO:0043434 | response to peptide hormone | 51/1207 | 2.10E-05 | 0.001222 | 51 |
| GO:0070661 | leukocyte proliferation | 40/1207 | 2.14E-05 | 0.001225 | 40 |
| GO:1905517 | macrophage migration | 13/1207 | 2.15E-05 | 0.001225 | 13 |
| GO:1905521 | regulation of macrophage migration | 11/1207 | 2.23E-05 | 0.001246 | 11 |
| GO:0099504 | synaptic vesicle cycle | 27/1207 | 2.26E-05 | 0.001246 | 27 |
| GO:0071675 | regulation of mononuclear cell migration | 20/1207 | 2.27E-05 | 0.001246 | 20 |
| GO:0097530 | granulocyte migration | 24/1207 | 2.28E-05 | 0.001246 | 24 |
| GO:0034329 | cell junction assembly | 50/1207 | 2.30E-05 | 0.001246 | 50 |
| GO:0032675 | regulation of interleukin-6 production | 25/1207 | 2.39E-05 | 0.001271 | 25 |
| GO:1902652 | secondary alcohol metabolic process | 25/1207 | 2.39E-05 | 0.001271 | 25 |
| GO:0071496 | cellular response to external stimulus | 39/1207 | 2.46E-05 | 0.001299 | 39 |
| GO:0006869 | lipid transport | 53/1207 | 2.50E-05 | 0.001307 | 53 |
| GO:0008203 | cholesterol metabolic process | 24/1207 | 2.56E-05 | 0.001322 | 24 |
| GO:0007249 | I-kappaB kinase/NF-kappaB signaling | 37/1207 | 2.59E-05 | 0.001326 | 37 |
| GO:0001666 | response to hypoxia | 43/1207 | 2.64E-05 | 0.001328 | 43 |
| GO:0032633 | interleukin-4 production | 10/1207 | 2.64E-05 | 0.001328 | 10 |
| GO:0098742 | cell-cell adhesion via plasma-membrane adhesion molecules | 36/1207 | 2.97E-05 | 0.00148 | 36 |
| GO:0007162 | negative regulation of cell adhesion | 38/1207 | 3.06E-05 | 0.001499 | 38 |
| GO:0071674 | mononuclear cell migration | 28/1207 | 3.06E-05 | 0.001499 | 28 |
| GO:0006066 | alcohol metabolic process | 45/1207 | 3.14E-05 | 0.001524 | 45 |
| GO:0048661 | positive regulation of smooth muscle cell proliferation | 18/1207 | 3.31E-05 | 0.00159 | 18 |
| GO:0033002 | muscle cell proliferation | 31/1207 | 3.46E-05 | 0.001648 | 31 |
| GO:0006865 | amino acid transport | 24/1207 | 3.57E-05 | 0.001677 | 24 |
| GO:0002687 | positive regulation of leukocyte migration | 22/1207 | 3.61E-05 | 0.001677 | 22 |
| GO:0052372 | modulation by symbiont of entry into host | 12/1207 | 3.61E-05 | 0.001677 | 12 |
| GO:0032635 | interleukin-6 production | 25/1207 | 3.68E-05 | 0.001694 | 25 |
| GO:0046596 | regulation of viral entry into host cell | 11/1207 | 3.74E-05 | 0.001707 | 11 |
| GO:0042590 | antigen processing and presentation of exogenous peptide antigen via MHC class I | 16/1207 | 3.93E-05 | 0.001782 | 16 |
| GO:0001778 | plasma membrane repair | 6/1207 | 4.48E-05 | 0.001997 | 6 |
| GO:0048266 | behavioral response to pain | 6/1207 | 4.48E-05 | 0.001997 | 6 |
| GO:0002040 | sprouting angiogenesis | 27/1207 | 4.55E-05 | 0.00201 | 27 |
| GO:0010759 | positive regulation of macrophage chemotaxis | 7/1207 | 4.75E-05 | 0.002083 | 7 |
| GO:0035249 | synaptic transmission glutamatergic glutamatergic | 17/1207 | 4.92E-05 | 0.002141 | 17 |
| GO:0048771 | tissue remodeling | 26/1207 | 5.04E-05 | 0.002174 | 26 |
| GO:0001938 | positive regulation of endothelial cell proliferation | 19/1207 | 5.08E-05 | 0.002174 | 19 |
| GO:0042493 | response to drug | 43/1207 | 5.53E-05 | 0.002351 | 43 |
| GO:0019058 | viral life cycle | 42/1207 | 5.65E-05 | 0.002369 | 42 |
| GO:0016338 | calcium-independent cell-cell adhesion via plasma membrane cell-adhesion molecules | 8/1207 | 5.66E-05 | 0.002369 | 8 |
| GO:0071260 | cellular response to mechanical stimulus | 15/1207 | 5.78E-05 | 0.002373 | 15 |
| GO:0001894 | tissue homeostasis | 34/1207 | 5.80E-05 | 0.002373 | 34 |
| GO:0032102 | negative regulation of response to external stimulus | 46/1207 | 5.80E-05 | 0.002373 | 46 |
| GO:0045088 | regulation of innate immune response | 39/1207 | 5.89E-05 | 0.00239 | 39 |
| GO:1902930 | regulation of alcohol biosynthetic process | 15/1207 | 6.80E-05 | 0.002742 | 15 |
| GO:0006836 | neurotransmitter transport | 29/1207 | 6.86E-05 | 0.002745 | 29 |
| GO:0060759 | regulation of response to cytokine stimulus | 27/1207 | 7.29E-05 | 0.002852 | 27 |
| GO:0008625 | extrinsic apoptotic signaling pathway via death domain receptors | 16/1207 | 7.32E-05 | 0.002852 | 16 |
| GO:0006936 | muscle contraction | 42/1207 | 7.34E-05 | 0.002852 | 42 |
| GO:0010038 | response to metal ion | 42/1207 | 7.34E-05 | 0.002852 | 42 |
| GO:0050866 | negative regulation of cell activation | 28/1207 | 7.76E-05 | 0.002995 | 28 |
| GO:1903131 | mononuclear cell differentiation | 47/1207 | 8.05E-05 | 0.003087 | 47 |
| GO:0050808 | synapse organization | 47/1207 | 8.54E-05 | 0.003252 | 47 |
| GO:0030593 | neutrophil chemotaxis | 18/1207 | 8.68E-05 | 0.003281 | 18 |
| GO:0032944 | regulation of mononuclear cell proliferation | 30/1207 | 9.22E-05 | 0.003461 | 30 |
| GO:0008016 | regulation of heart contraction | 32/1207 | 9.67E-05 | 0.003606 | 32 |
| GO:0002695 | negative regulation of leukocyte activation | 26/1207 | 9.84E-05 | 0.003621 | 26 |
| GO:0003015 | heart process | 36/1207 | 9.84E-05 | 0.003621 | 36 |
| GO:0051966 | regulation of synaptic transmission glutamatergic | 14/1207 | 0.0001 | 0.003659 | 14 |
| GO:0050679 | positive regulation of epithelial cell proliferation | 28/1207 | 0.000101 | 0.003663 | 28 |
| GO:0030001 | metal ion transport | 53/1207 | 0.000102 | 0.003677 | 53 |
| GO:0046686 | response to cadmium ion | 13/1207 | 0.000104 | 0.003677 | 13 |
| GO:0090181 | regulation of cholesterol metabolic process | 13/1207 | 0.000104 | 0.003677 | 13 |
| GO:0044272 | sulfur compound biosynthetic process | 27/1207 | 0.000105 | 0.003677 | 27 |
| GO:1901654 | response to ketone | 27/1207 | 0.000105 | 0.003677 | 27 |
| GO:0050729 | positive regulation of inflammatory response | 21/1207 | 0.000107 | 0.003753 | 21 |
| GO:0010975 | regulation of neuron projection development | 48/1207 | 0.000109 | 0.003796 | 48 |
| GO:0060401 | cytosolic calcium ion transport | 25/1207 | 0.00011 | 0.003798 | 25 |
| GO:0099003 | vesicle-mediated transport in synapse | 27/1207 | 0.000114 | 0.003916 | 27 |
| GO:0003012 | muscle system process | 50/1207 | 0.000118 | 0.004022 | 50 |
| GO:0033209 | tumor necrosis factor-mediated signaling pathway | 25/1207 | 0.000121 | 0.004094 | 25 |
| GO:0051282 | regulation of sequestering of calcium ion | 19/1207 | 0.000122 | 0.004098 | 19 |
| GO:0014074 | response to purine-containing compound | 22/1207 | 0.000123 | 0.004098 | 22 |
| GO:1905039 | carboxylic acid transmembrane transport | 23/1207 | 0.000124 | 0.004104 | 23 |
| GO:1901888 | regulation of cell junction assembly | 27/1207 | 0.000125 | 0.00412 | 27 |
| GO:0045766 | positive regulation of angiogenesis | 25/1207 | 0.000133 | 0.004332 | 25 |
| GO:1904018 | positive regulation of vasculature development | 25/1207 | 0.000133 | 0.004332 | 25 |
| GO:1903825 | organic acid transmembrane transport | 23/1207 | 0.000136 | 0.004425 | 23 |
| GO:0017157 | regulation of exocytosis | 27/1207 | 0.000148 | 0.004765 | 27 |
| GO:1903305 | regulation of regulated secretory pathway | 21/1207 | 0.000149 | 0.004765 | 21 |
| GO:0019883 | antigen processing and presentation of endogenous antigen | 8/1207 | 0.000152 | 0.00481 | 8 |
| GO:2000311 | regulation of AMPA receptor activity | 8/1207 | 0.000152 | 0.00481 | 8 |
| GO:0042908 | xenobiotic transport | 10/1207 | 0.00016 | 0.004972 | 10 |
| GO:0035296 | regulation of tube diameter | 20/1207 | 0.000161 | 0.004972 | 20 |
| GO:0097746 | blood vessel diameter maintenance | 20/1207 | 0.000161 | 0.004972 | 20 |
| GO:2000116 | regulation of cysteine-type endopeptidase activity | 30/1207 | 0.000161 | 0.004972 | 30 |
| GO:1901136 | carbohydrate derivative catabolic process | 27/1207 | 0.000161 | 0.004972 | 27 |
| GO:0048638 | regulation of developmental growth | 38/1207 | 0.000164 | 0.004995 | 38 |
| GO:0051235 | maintenance of location | 38/1207 | 0.000164 | 0.004995 | 38 |
| GO:0030168 | platelet activation | 23/1207 | 0.000166 | 0.005024 | 23 |
| GO:0003014 | renal system process | 19/1207 | 0.000173 | 0.005208 | 19 |
| GO:0015718 | monocarboxylic acid transport | 25/1207 | 0.000174 | 0.005215 | 25 |
| GO:0007159 | leukocyte cell-cell adhesion | 42/1207 | 0.000175 | 0.005215 | 42 |
| GO:0045785 | positive regulation of cell adhesion | 47/1207 | 0.000179 | 0.005246 | 47 |
| GO:0006790 | sulfur compound metabolic process | 43/1207 | 0.000179 | 0.005246 | 43 |
| GO:0035150 | regulation of tube size | 20/1207 | 0.00018 | 0.005246 | 20 |
| GO:0022407 | regulation of cell-cell adhesion | 48/1207 | 0.00018 | 0.005246 | 48 |
| GO:0045444 | fat cell differentiation | 29/1207 | 0.000186 | 0.005354 | 29 |
| GO:0050670 | regulation of lymphocyte proliferation | 29/1207 | 0.000186 | 0.005354 | 29 |
| GO:0002832 | negative regulation of response to biotic stimulus | 17/1207 | 0.000191 | 0.005489 | 17 |
| GO:0051208 | sequestering of calcium ion | 19/1207 | 0.000194 | 0.005529 | 19 |
| GO:0002690 | positive regulation of leukocyte chemotaxis | 16/1207 | 0.000196 | 0.005531 | 16 |
| GO:0050810 | regulation of steroid biosynthetic process | 16/1207 | 0.000196 | 0.005531 | 16 |
| GO:0033003 | regulation of mast cell activation | 10/1207 | 0.0002 | 0.005627 | 10 |
| GO:0050921 | positive regulation of chemotaxis | 21/1207 | 0.000203 | 0.005639 | 21 |
| GO:0051091 | positive regulation of DNA-binding transcription factor activity | 33/1207 | 0.000204 | 0.005639 | 33 |
| GO:0010758 | regulation of macrophage chemotaxis | 8/1207 | 0.000204 | 0.005639 | 8 |
| GO:0010043 | response to zinc ion | 12/1207 | 0.000215 | 0.00588 | 12 |
| GO:0002819 | regulation of adaptive immune response | 24/1207 | 0.000215 | 0.00588 | 24 |
| GO:0030198 | extracellular matrix organization | 44/1207 | 0.000216 | 0.00588 | 44 |
| GO:0009914 | hormone transport | 37/1207 | 0.000217 | 0.00588 | 37 |
| GO:0031032 | actomyosin structure organization | 26/1207 | 0.000218 | 0.00588 | 26 |
| GO:0046683 | response to organophosphorus | 20/1207 | 0.000223 | 0.00598 | 20 |
| GO:0015908 | fatty acid transport | 22/1207 | 0.000224 | 0.005991 | 22 |
| GO:0016048 | detection of temperature stimulus | 7/1207 | 0.000226 | 0.006026 | 7 |
| GO:0043062 | extracellular structure organization | 44/1207 | 0.000229 | 0.006048 | 44 |
| GO:0031589 | cell-substrate adhesion | 41/1207 | 0.00023 | 0.006048 | 41 |
| GO:0060047 | heart contraction | 34/1207 | 0.000231 | 0.006048 | 34 |
| GO:0022612 | gland morphogenesis | 18/1207 | 0.000232 | 0.006048 | 18 |
| GO:0007611 | learning or memory | 30/1207 | 0.000236 | 0.006139 | 30 |
| GO:0001822 | kidney development | 34/1207 | 0.000247 | 0.006392 | 34 |
| GO:0002479 | antigen processing and presentation of exogenous peptide antigen via MHC class I TAP-dependent | 14/1207 | 0.000254 | 0.006515 | 14 |
| GO:0031348 | negative regulation of defense response | 30/1207 | 0.000255 | 0.006515 | 30 |
| GO:0045229 | external encapsulating structure organization | 44/1207 | 0.000256 | 0.006515 | 44 |
| GO:0031349 | positive regulation of defense response | 41/1207 | 0.000259 | 0.006571 | 41 |
| GO:0051090 | regulation of DNA-binding transcription factor activity | 48/1207 | 0.000261 | 0.006587 | 48 |
| GO:0044706 | multi-multicellular organism process | 27/1207 | 0.000265 | 0.006657 | 27 |
| GO:0090066 | regulation of anatomical structure size | 52/1207 | 0.00027 | 0.006756 | 52 |
| GO:0060402 | calcium ion transport into cytosol | 22/1207 | 0.000271 | 0.006763 | 22 |
| GO:1903532 | positive regulation of secretion by cell | 33/1207 | 0.000288 | 0.007128 | 33 |
| GO:0051960 | regulation of nervous system development | 46/1207 | 0.000289 | 0.007128 | 46 |
| GO:0051283 | negative regulation of sequestering of calcium ion | 18/1207 | 0.000291 | 0.007152 | 18 |
| GO:0046928 | regulation of neurotransmitter secretion | 15/1207 | 0.000294 | 0.007195 | 15 |
| GO:0048511 | rhythmic process | 35/1207 | 0.000295 | 0.007195 | 35 |
| GO:0030239 | myofibril assembly | 12/1207 | 0.000302 | 0.007325 | 12 |
| GO:0042220 | response to cocaine | 10/1207 | 0.000305 | 0.007356 | 10 |
| GO:0070555 | response to interleukin-1 | 27/1207 | 0.00031 | 0.007429 | 27 |
| GO:0016079 | synaptic vesicle exocytosis | 17/1207 | 0.00031 | 0.007429 | 17 |
| GO:0009636 | response to toxic substance | 30/1207 | 0.000317 | 0.007555 | 30 |
| GO:0006023 | aminoglycan biosynthetic process | 18/1207 | 0.000325 | 0.007697 | 18 |
| GO:1903037 | regulation of leukocyte cell-cell adhesion | 38/1207 | 0.000326 | 0.007697 | 38 |
| GO:0043491 | protein kinase B signaling | 33/1207 | 0.000329 | 0.007739 | 33 |
| GO:0007596 | blood coagulation | 39/1207 | 0.000333 | 0.00781 | 39 |
| GO:0034103 | regulation of tissue remodeling | 15/1207 | 0.000336 | 0.007833 | 15 |
| GO:0048732 | gland development | 45/1207 | 0.000337 | 0.007837 | 45 |
| GO:0010810 | regulation of cell-substrate adhesion | 28/1207 | 0.00034 | 0.007837 | 28 |
| GO:0030224 | monocyte differentiation | 9/1207 | 0.00034 | 0.007837 | 9 |
| GO:0032943 | mononuclear cell proliferation | 34/1207 | 0.000344 | 0.007882 | 34 |
| GO:0050727 | regulation of inflammatory response | 41/1207 | 0.000345 | 0.007882 | 41 |
| GO:2001269 | positive regulation of cysteine-type endopeptidase activity involved in apoptotic signaling pathway | 5/1207 | 0.000355 | 0.00806 | 5 |
| GO:1903670 | regulation of sprouting angiogenesis | 12/1207 | 0.000356 | 0.00806 | 12 |
| GO:0007269 | neurotransmitter secretion | 22/1207 | 0.000359 | 0.008064 | 22 |
| GO:0099643 | signal release from synapse | 22/1207 | 0.000359 | 0.008064 | 22 |
| GO:0007160 | cell-matrix adhesion | 29/1207 | 0.000367 | 0.008213 | 29 |
| GO:0045124 | regulation of bone resorption | 10/1207 | 0.000372 | 0.008258 | 10 |
| GO:1901653 | cellular response to peptide | 43/1207 | 0.000372 | 0.008258 | 43 |
| GO:0071229 | cellular response to acid chemical | 13/1207 | 0.00038 | 0.00839 | 13 |
| GO:0032760 | positive regulation of tumor necrosis factor production | 15/1207 | 0.000382 | 0.008414 | 15 |
| GO:0001959 | regulation of cytokine-mediated signaling pathway | 24/1207 | 0.000397 | 0.008693 | 24 |
| GO:0050890 | cognition | 33/1207 | 0.000401 | 0.00876 | 33 |
| GO:0045471 | response to ethanol | 19/1207 | 0.00041 | 0.008872 | 19 |
| GO:2001235 | positive regulation of apoptotic signaling pathway | 19/1207 | 0.00041 | 0.008872 | 19 |
| GO:0007156 | homophilic cell adhesion via plasma membrane adhesion molecules | 23/1207 | 0.000415 | 0.008872 | 23 |
| GO:1990868 | response to chemokine | 16/1207 | 0.000415 | 0.008872 | 16 |
| GO:1990869 | cellular response to chemokine | 16/1207 | 0.000415 | 0.008872 | 16 |
| GO:0072001 | renal system development | 34/1207 | 0.000418 | 0.008872 | 34 |
| GO:1905330 | regulation of morphogenesis of an epithelium | 12/1207 | 0.000418 | 0.008872 | 12 |
| GO:0007599 | hemostasis | 39/1207 | 0.000421 | 0.008905 | 39 |
| GO:0070098 | chemokine-mediated signaling pathway | 15/1207 | 0.000434 | 0.009149 | 15 |
| GO:0001558 | regulation of cell growth | 44/1207 | 0.000438 | 0.009207 | 44 |
| GO:0010524 | positive regulation of calcium ion transport into cytosol | 11/1207 | 0.000445 | 0.009216 | 11 |
| GO:0050817 | coagulation | 39/1207 | 0.000446 | 0.009216 | 39 |
| GO:0046685 | response to arsenic-containing substance | 8/1207 | 0.000452 | 0.009216 | 8 |
| GO:0051968 | positive regulation of synaptic transmission glutamatergic | 8/1207 | 0.000452 | 0.009216 | 8 |
| GO:0070633 | transepithelial transport | 8/1207 | 0.000452 | 0.009216 | 8 |
| GO:0007158 | neuron cell-cell adhesion | 6/1207 | 0.000455 | 0.009216 | 6 |
| GO:0050961 | detection of temperature stimulus involved in sensory perception | 6/1207 | 0.000455 | 0.009216 | 6 |
| GO:0061687 | detoxification of inorganic compound | 6/1207 | 0.000455 | 0.009216 | 6 |
| GO:0071216 | cellular response to biotic stimulus | 29/1207 | 0.000456 | 0.009216 | 29 |
| GO:0097305 | response to alcohol | 29/1207 | 0.000456 | 0.009216 | 29 |
| GO:2001233 | regulation of apoptotic signaling pathway | 39/1207 | 0.000472 | 0.009506 | 39 |
| GO:0006694 | steroid biosynthetic process | 25/1207 | 0.00048 | 0.00964 | 25 |
| GO:0045453 | bone resorption | 12/1207 | 0.000488 | 0.009763 | 12 |
| GO:0032642 | regulation of chemokine production | 15/1207 | 0.000492 | 0.009763 | 15 |
| GO:0033273 | response to vitamin | 15/1207 | 0.000492 | 0.009763 | 15 |
| GO:0032479 | regulation of type I interferon production | 19/1207 | 0.000502 | 0.009929 | 19 |
| GO:0048246 | macrophage chemotaxis | 9/1207 | 0.000525 | 0.010161 | 9 |
| GO:0071276 | cellular response to cadmium ion | 9/1207 | 0.000525 | 0.010161 | 9 |
| GO:0071548 | response to dexamethasone | 9/1207 | 0.000525 | 0.010161 | 9 |
| GO:0002474 | antigen processing and presentation of peptide antigen via MHC class I | 16/1207 | 0.000525 | 0.010161 | 16 |
| GO:0019233 | sensory perception of pain | 16/1207 | 0.000525 | 0.010161 | 16 |
| GO:0043279 | response to alkaloid | 16/1207 | 0.000525 | 0.010161 | 16 |
| GO:0097479 | synaptic vesicle localization | 11/1207 | 0.000526 | 0.010161 | 11 |
| GO:1903531 | negative regulation of secretion by cell | 20/1207 | 0.000546 | 0.010498 | 20 |
| GO:0032606 | type I interferon production | 19/1207 | 0.000554 | 0.010538 | 19 |
| GO:0051896 | regulation of protein kinase B signaling | 30/1207 | 0.000555 | 0.010538 | 30 |
| GO:0003073 | regulation of systemic arterial blood pressure | 15/1207 | 0.000556 | 0.010538 | 15 |
| GO:1903557 | positive regulation of tumor necrosis factor superfamily cytokine production | 15/1207 | 0.000556 | 0.010538 | 15 |
| GO:0051048 | negative regulation of secretion | 22/1207 | 0.00056 | 0.010586 | 22 |
| GO:0048015 | phosphatidylinositol-mediated signaling | 25/1207 | 0.000562 | 0.010586 | 25 |
| GO:0048662 | negative regulation of smooth muscle cell proliferation | 12/1207 | 0.000568 | 0.010604 | 12 |
| GO:0036003 | positive regulation of transcription from RNA polymerase II promoter in response to stress | 7/1207 | 0.000569 | 0.010604 | 7 |
| GO:0097421 | liver regeneration | 8/1207 | 0.000576 | 0.010604 | 8 |
| GO:0032429 | regulation of phospholipase A2 activity | 5/1207 | 0.000577 | 0.010604 | 5 |
| GO:0033004 | negative regulation of mast cell activation | 5/1207 | 0.000577 | 0.010604 | 5 |
| GO:0035672 | oligopeptide transmembrane transport | 5/1207 | 0.000577 | 0.010604 | 5 |
| GO:0140289 | protein mono-ADP-ribosylation | 5/1207 | 0.000577 | 0.010604 | 5 |
| GO:0046651 | lymphocyte proliferation | 33/1207 | 0.000589 | 0.010757 | 33 |
| GO:0006939 | smooth muscle contraction | 16/1207 | 0.000589 | 0.010757 | 16 |
| GO:0098739 | import across plasma membrane | 22/1207 | 0.000611 | 0.011119 | 22 |
| GO:0040013 | negative regulation of locomotion | 41/1207 | 0.000631 | 0.011443 | 41 |
| GO:0048872 | homeostasis of number of cells | 30/1207 | 0.000634 | 0.011477 | 30 |
| GO:0045953 | negative regulation of natural killer cell mediated cytotoxicity | 6/1207 | 0.000645 | 0.011487 | 6 |
| GO:0046851 | negative regulation of bone remodeling | 6/1207 | 0.000645 | 0.011487 | 6 |
| GO:0071243 | cellular response to arsenic-containing substance | 6/1207 | 0.000645 | 0.011487 | 6 |
| GO:0097501 | stress response to metal ion | 6/1207 | 0.000645 | 0.011487 | 6 |
| GO:0051924 | regulation of calcium ion transport | 29/1207 | 0.000646 | 0.011487 | 29 |
| GO:2000379 | positive regulation of reactive oxygen species metabolic process | 16/1207 | 0.000659 | 0.011586 | 16 |
| GO:0051893 | regulation of focal adhesion assembly | 12/1207 | 0.000659 | 0.011586 | 12 |
| GO:0090109 | regulation of cell-substrate junction assembly | 12/1207 | 0.000659 | 0.011586 | 12 |
| GO:0043281 | regulation of cysteine-type endopeptidase activity involved in apoptotic process | 26/1207 | 0.00066 | 0.011586 | 26 |
| GO:0031623 | receptor internalization | 17/1207 | 0.000675 | 0.011818 | 17 |
| GO:0031667 | response to nutrient levels | 47/1207 | 0.000677 | 0.011818 | 47 |
| GO:0001818 | negative regulation of cytokine production | 40/1207 | 0.000699 | 0.012165 | 40 |
| GO:1901655 | cellular response to ketone | 15/1207 | 0.000705 | 0.012234 | 15 |
| GO:0001655 | urogenital system development | 36/1207 | 0.000712 | 0.012318 | 36 |
| GO:0009308 | amine metabolic process | 22/1207 | 0.000723 | 0.012458 | 22 |
| GO:0048011 | neurotrophin TRK receptor signaling pathway | 8/1207 | 0.000725 | 0.012458 | 8 |
| GO:0060760 | positive regulation of response to cytokine stimulus | 11/1207 | 0.000728 | 0.012475 | 11 |
| GO:0002092 | positive regulation of receptor internalization | 7/1207 | 0.000747 | 0.012648 | 7 |
| GO:0035902 | response to immobilization stress | 7/1207 | 0.000747 | 0.012648 | 7 |
| GO:0030278 | regulation of ossification | 17/1207 | 0.00075 | 0.012648 | 17 |
| GO:0051209 | release of sequestered calcium ion into cytosol | 17/1207 | 0.00075 | 0.012648 | 17 |
| GO:0019218 | regulation of steroid metabolic process | 18/1207 | 0.00075 | 0.012648 | 18 |
| GO:0051962 | positive regulation of nervous system development | 31/1207 | 0.000753 | 0.012665 | 31 |
| GO:0051250 | negative regulation of lymphocyte activation | 21/1207 | 0.000758 | 0.012701 | 21 |
| GO:0048017 | inositol lipid-mediated signaling | 25/1207 | 0.000763 | 0.01275 | 25 |
| GO:0045778 | positive regulation of ossification | 10/1207 | 0.000777 | 0.01291 | 10 |
| GO:0018108 | peptidyl-tyrosine phosphorylation | 40/1207 | 0.000777 | 0.01291 | 40 |
| GO:1903034 | regulation of response to wounding | 22/1207 | 0.000786 | 0.013014 | 22 |
| GO:0051591 | response to cAMP | 15/1207 | 0.000792 | 0.013078 | 15 |
| GO:0052547 | regulation of peptidase activity | 47/1207 | 0.000818 | 0.013426 | 47 |
| GO:0055002 | striated muscle cell development | 16/1207 | 0.000821 | 0.013426 | 16 |
| GO:2001237 | negative regulation of extrinsic apoptotic signaling pathway | 16/1207 | 0.000821 | 0.013426 | 16 |
| GO:1990266 | neutrophil migration | 18/1207 | 0.000827 | 0.013492 | 18 |
| GO:0002718 | regulation of cytokine production involved in immune response | 14/1207 | 0.000839 | 0.013652 | 14 |
| GO:0062197 | cellular response to chemical stress | 38/1207 | 0.000859 | 0.013883 | 38 |
| GO:0106106 | cold-induced thermogenesis | 20/1207 | 0.000861 | 0.013883 | 20 |
| GO:0120161 | regulation of cold-induced thermogenesis | 20/1207 | 0.000861 | 0.013883 | 20 |
| GO:0006979 | response to oxidative stress | 46/1207 | 0.000869 | 0.013966 | 46 |
| GO:0051651 | maintenance of location in cell | 26/1207 | 0.000882 | 0.014014 | 26 |
| GO:0006857 | oligopeptide transport | 5/1207 | 0.000888 | 0.014014 | 5 |
| GO:0032905 | transforming growth factor beta1 production | 5/1207 | 0.000888 | 0.014014 | 5 |
| GO:0045741 | positive regulation of epidermal growth factor-activated receptor activity | 5/1207 | 0.000888 | 0.014014 | 5 |
| GO:0097091 | synaptic vesicle clustering | 5/1207 | 0.000888 | 0.014014 | 5 |
| GO:0097553 | calcium ion transmembrane import into cytosol | 19/1207 | 0.00089 | 0.014014 | 19 |
| GO:0002544 | chronic inflammatory response | 6/1207 | 0.000892 | 0.014014 | 6 |
| GO:0002716 | negative regulation of natural killer cell mediated immunity | 6/1207 | 0.000892 | 0.014014 | 6 |
| GO:0002724 | regulation of T cell cytokine production | 8/1207 | 0.000904 | 0.014077 | 8 |
| GO:0014046 | dopamine secretion | 8/1207 | 0.000904 | 0.014077 | 8 |
| GO:0014059 | regulation of dopamine secretion | 8/1207 | 0.000904 | 0.014077 | 8 |
| GO:0018212 | peptidyl-tyrosine modification | 40/1207 | 0.000909 | 0.014114 | 40 |
| GO:0045661 | regulation of myoblast differentiation | 10/1207 | 0.00092 | 0.014212 | 10 |
| GO:0071622 | regulation of granulocyte chemotaxis | 10/1207 | 0.00092 | 0.014212 | 10 |
| GO:0016049 | cell growth | 48/1207 | 0.000925 | 0.014246 | 48 |
| GO:0002262 | myeloid cell homeostasis | 20/1207 | 0.00094 | 0.014433 | 20 |
| GO:0007565 | female pregnancy | 23/1207 | 0.000943 | 0.014433 | 23 |
| GO:0032689 | negative regulation of interferon-gamma production | 9/1207 | 0.000949 | 0.014445 | 9 |
| GO:1902042 | negative regulation of extrinsic apoptotic signaling pathway via death domain receptors | 9/1207 | 0.000949 | 0.014445 | 9 |
| GO:0046879 | hormone secretion | 34/1207 | 0.000975 | 0.01477 | 34 |
| GO:0043524 | negative regulation of neuron apoptotic process | 19/1207 | 0.000976 | 0.01477 | 19 |
| GO:0032602 | chemokine production | 15/1207 | 0.000993 | 0.014997 | 15 |
| GO:0001952 | regulation of cell-matrix adhesion | 18/1207 | 0.001002 | 0.015087 | 18 |
| GO:1901224 | positive regulation of NIK/NF-kappaB signaling | 12/1207 | 0.001009 | 0.015113 | 12 |
| GO:0042692 | muscle cell differentiation | 39/1207 | 0.001009 | 0.015113 | 39 |
| GO:0001505 | regulation of neurotransmitter levels | 26/1207 | 0.001016 | 0.015137 | 26 |
| GO:1903510 | mucopolysaccharide metabolic process | 17/1207 | 0.001016 | 0.015137 | 17 |
| GO:0022408 | negative regulation of cell-cell adhesion | 24/1207 | 0.001025 | 0.015204 | 24 |
| GO:0045926 | negative regulation of growth | 29/1207 | 0.001026 | 0.015204 | 29 |
| GO:0001667 | ameboidal-type cell migration | 48/1207 | 0.001059 | 0.015652 | 48 |
| GO:0032755 | positive regulation of interleukin-6 production | 14/1207 | 0.001066 | 0.015686 | 14 |
| GO:0032946 | positive regulation of mononuclear cell proliferation | 19/1207 | 0.001067 | 0.015686 | 19 |
| GO:0051146 | striated muscle cell differentiation | 31/1207 | 0.001094 | 0.016029 | 31 |
| GO:0003333 | amino acid transmembrane transport | 15/1207 | 0.001109 | 0.016209 | 15 |
| GO:0033280 | response to vitamin D | 8/1207 | 0.001117 | 0.016243 | 8 |
| GO:0007043 | cell-cell junction assembly | 20/1207 | 0.001117 | 0.016243 | 20 |
| GO:0051271 | negative regulation of cellular component movement | 38/1207 | 0.00112 | 0.016244 | 38 |
| GO:0089718 | amino acid import across plasma membrane | 9/1207 | 0.00114 | 0.016442 | 9 |
| GO:2001239 | regulation of extrinsic apoptotic signaling pathway in absence of ligand | 9/1207 | 0.00114 | 0.016442 | 9 |
| GO:0001503 | ossification | 42/1207 | 0.001155 | 0.016589 | 42 |
| GO:0022604 | regulation of cell morphogenesis | 34/1207 | 0.001156 | 0.016589 | 34 |
| GO:0010959 | regulation of metal ion transport | 29/1207 | 0.001166 | 0.016687 | 29 |
| GO:0071347 | cellular response to interleukin-1 | 23/1207 | 0.001186 | 0.016935 | 23 |
| GO:0034104 | negative regulation of tissue remodeling | 6/1207 | 0.001206 | 0.017129 | 6 |
| GO:0046597 | negative regulation of viral entry into host cell | 6/1207 | 0.001206 | 0.017129 | 6 |
| GO:0070665 | positive regulation of leukocyte proliferation | 20/1207 | 0.001216 | 0.017221 | 20 |
| GO:0015850 | organic hydroxy compound transport | 31/1207 | 0.001234 | 0.017413 | 31 |
| GO:0120162 | positive regulation of cold-induced thermogenesis | 15/1207 | 0.001236 | 0.017413 | 15 |
| GO:0006024 | glycosaminoglycan biosynthetic process | 16/1207 | 0.001247 | 0.017428 | 16 |
| GO:0007613 | memory | 16/1207 | 0.001247 | 0.017428 | 16 |
| GO:0032649 | regulation of interferon-gamma production | 16/1207 | 0.001247 | 0.017428 | 16 |
| GO:0050803 | regulation of synapse structure or activity | 26/1207 | 0.00125 | 0.017428 | 26 |
| GO:0009991 | response to extracellular stimulus | 48/1207 | 0.001265 | 0.017562 | 48 |
| GO:0002709 | regulation of T cell mediated immunity | 13/1207 | 0.00127 | 0.017562 | 13 |
| GO:0048639 | positive regulation of developmental growth | 22/1207 | 0.00127 | 0.017562 | 22 |
| GO:1903038 | negative regulation of leukocyte cell-cell adhesion | 19/1207 | 0.001274 | 0.017562 | 19 |
| GO:0031099 | regeneration | 24/1207 | 0.001277 | 0.017562 | 24 |
| GO:0051047 | positive regulation of secretion | 33/1207 | 0.001278 | 0.017562 | 33 |
| GO:0150116 | regulation of cell-substrate junction organization | 12/1207 | 0.001318 | 0.018066 | 12 |
| GO:0032615 | interleukin-12 production | 11/1207 | 0.001326 | 0.018083 | 11 |
| GO:0032655 | regulation of interleukin-12 production | 11/1207 | 0.001326 | 0.018083 | 11 |
| GO:0070664 | negative regulation of leukocyte proliferation | 14/1207 | 0.001341 | 0.018238 | 14 |
| GO:0032680 | regulation of tumor necrosis factor production | 21/1207 | 0.001354 | 0.018371 | 21 |
| GO:0035094 | response to nicotine | 9/1207 | 0.001361 | 0.018419 | 9 |
| GO:0001659 | temperature homeostasis | 22/1207 | 0.001372 | 0.018521 | 22 |
| GO:0055076 | transition metal ion homeostasis | 19/1207 | 0.001389 | 0.018704 | 19 |
| GO:0042310 | vasoconstriction | 13/1207 | 0.001433 | 0.019252 | 13 |
| GO:0006814 | sodium ion transport | 28/1207 | 0.001451 | 0.019447 | 28 |
| GO:0006937 | regulation of muscle contraction | 21/1207 | 0.001466 | 0.019593 | 21 |
| GO:0006022 | aminoglycan metabolic process | 22/1207 | 0.00148 | 0.019743 | 22 |
| GO:0050433 | regulation of catecholamine secretion | 10/1207 | 0.001489 | 0.019772 | 10 |
| GO:0001101 | response to acid chemical | 17/1207 | 0.001493 | 0.019772 | 17 |
| GO:0002688 | regulation of leukocyte chemotaxis | 17/1207 | 0.001493 | 0.019772 | 17 |
| GO:0046718 | viral entry into host cell | 19/1207 | 0.001513 | 0.01996 | 19 |
| GO:0030099 | myeloid cell differentiation | 43/1207 | 0.001515 | 0.01996 | 43 |
| GO:0070059 | intrinsic apoptotic signaling pathway in response to endoplasmic reticulum stress | 11/1207 | 0.001526 | 0.019966 | 11 |
| GO:0071230 | cellular response to amino acid stimulus | 11/1207 | 0.001526 | 0.019966 | 11 |
| GO:0051588 | regulation of neurotransmitter transport | 15/1207 | 0.001526 | 0.019966 | 15 |
| GO:0008299 | isoprenoid biosynthetic process | 7/1207 | 0.001555 | 0.020187 | 7 |
| GO:0032607 | interferon-alpha production | 7/1207 | 0.001555 | 0.020187 | 7 |
| GO:0032647 | regulation of interferon-alpha production | 7/1207 | 0.001555 | 0.020187 | 7 |
| GO:2001056 | positive regulation of cysteine-type endopeptidase activity | 20/1207 | 0.001558 | 0.020187 | 20 |
| GO:0032640 | tumor necrosis factor production | 21/1207 | 0.001585 | 0.020467 | 21 |
| GO:0035929 | steroid hormone secretion | 6/1207 | 0.001598 | 0.020467 | 6 |
| GO:0071636 | positive regulation of transforming growth factor beta production | 6/1207 | 0.001598 | 0.020467 | 6 |
| GO:0072574 | hepatocyte proliferation | 6/1207 | 0.001598 | 0.020467 | 6 |
| GO:0072575 | epithelial cell proliferation involved in liver morphogenesis | 6/1207 | 0.001598 | 0.020467 | 6 |
| GO:0015804 | neutral amino acid transport | 9/1207 | 0.001615 | 0.020639 | 9 |
| GO:0002369 | T cell cytokine production | 8/1207 | 0.001661 | 0.021171 | 8 |
| GO:0010927 | cellular component assembly involved in morphogenesis | 15/1207 | 0.001692 | 0.021514 | 15 |
| GO:0098586 | cellular response to virus | 12/1207 | 0.001702 | 0.021599 | 12 |
| GO:0061041 | regulation of wound healing | 18/1207 | 0.001727 | 0.021817 | 18 |
| GO:0002704 | negative regulation of leukocyte mediated immunity | 10/1207 | 0.001731 | 0.021817 | 10 |
| GO:0050432 | catecholamine secretion | 10/1207 | 0.001731 | 0.021817 | 10 |
| GO:0051701 | biological process involved in interaction with host | 26/1207 | 0.001743 | 0.021909 | 26 |
| GO:0070527 | platelet aggregation | 11/1207 | 0.00175 | 0.02195 | 11 |
| GO:0010811 | positive regulation of cell-substrate adhesion | 17/1207 | 0.001795 | 0.022468 | 17 |
| GO:0071222 | cellular response to lipopolysaccharide | 24/1207 | 0.001816 | 0.022678 | 24 |
| GO:0044409 | entry into host | 20/1207 | 0.001829 | 0.022728 | 20 |
| GO:1990845 | adaptive thermogenesis | 20/1207 | 0.001829 | 0.022728 | 20 |
| GO:1903555 | regulation of tumor necrosis factor superfamily cytokine production | 21/1207 | 0.001848 | 0.022921 | 21 |
| GO:0043542 | endothelial cell migration | 31/1207 | 0.001854 | 0.022937 | 31 |
| GO:0050965 | detection of temperature stimulus involved in sensory perception of pain | 5/1207 | 0.00186 | 0.02296 | 5 |
| GO:0045216 | cell-cell junction organization | 25/1207 | 0.001906 | 0.023429 | 25 |
| GO:0002707 | negative regulation of lymphocyte mediated immunity | 9/1207 | 0.001906 | 0.023429 | 9 |
| GO:0015909 | long-chain fatty acid transport | 12/1207 | 0.001926 | 0.023567 | 12 |
| GO:0036465 | synaptic vesicle recycling | 12/1207 | 0.001926 | 0.023567 | 12 |
| GO:0071692 | protein localization to extracellular region | 39/1207 | 0.001931 | 0.023567 | 39 |
| GO:0071549 | cellular response to dexamethasone stimulus | 7/1207 | 0.001938 | 0.023601 | 7 |
| GO:1902105 | regulation of leukocyte differentiation | 31/1207 | 0.001961 | 0.023837 | 31 |
| GO:0007584 | response to nutrient | 21/1207 | 0.001993 | 0.024104 | 21 |
| GO:0048846 | axon extension involved in axon guidance | 8/1207 | 0.002002 | 0.024104 | 8 |
| GO:1902284 | neuron projection extension involved in neuron projection guidance | 8/1207 | 0.002002 | 0.024104 | 8 |
| GO:0002043 | blood vessel endothelial cell proliferation involved in sprouting angiogenesis | 10/1207 | 0.002005 | 0.024104 | 10 |
| GO:0043331 | response to dsRNA | 10/1207 | 0.002005 | 0.024104 | 10 |
| GO:0006970 | response to osmotic stress | 13/1207 | 0.002029 | 0.024281 | 13 |
| GO:0032609 | interferon-gamma production | 16/1207 | 0.002029 | 0.024281 | 16 |
| GO:0001911 | negative regulation of leukocyte mediated cytotoxicity | 6/1207 | 0.00208 | 0.024571 | 6 |
| GO:0032069 | regulation of nuclease activity | 6/1207 | 0.00208 | 0.024571 | 6 |
| GO:0032727 | positive regulation of interferon-alpha production | 6/1207 | 0.00208 | 0.024571 | 6 |
| GO:0035458 | cellular response to interferon-beta | 6/1207 | 0.00208 | 0.024571 | 6 |
| GO:0072576 | liver morphogenesis | 6/1207 | 0.00208 | 0.024571 | 6 |
| GO:0052548 | regulation of endopeptidase activity | 43/1207 | 0.00208 | 0.024571 | 43 |
| GO:0002822 | regulation of adaptive immune response based on somatic recombination of immune receptors built from immunoglobulin superfamily domains | 20/1207 | 0.002138 | 0.025205 | 20 |
| GO:0090130 | tissue migration | 38/1207 | 0.00215 | 0.025283 | 38 |
| GO:0008202 | steroid metabolic process | 35/1207 | 0.002203 | 0.025855 | 35 |
| GO:0002697 | regulation of immune effector process | 46/1207 | 0.002216 | 0.025912 | 46 |
| GO:1903706 | regulation of hemopoiesis | 42/1207 | 0.002217 | 0.025912 | 42 |
| GO:0010952 | positive regulation of peptidase activity | 24/1207 | 0.002225 | 0.025949 | 24 |
| GO:0006953 | acute-phase response | 9/1207 | 0.002238 | 0.026043 | 9 |
| GO:0071248 | cellular response to metal ion | 23/1207 | 0.002268 | 0.026244 | 23 |
| GO:1901292 | nucleoside phosphate catabolic process | 13/1207 | 0.002268 | 0.026244 | 13 |
| GO:0042267 | natural killer cell mediated cytotoxicity | 11/1207 | 0.002278 | 0.026244 | 11 |
| GO:0031532 | actin cytoskeleton reorganization | 15/1207 | 0.00228 | 0.026244 | 15 |
| GO:1903169 | regulation of calcium ion transmembrane transport | 19/1207 | 0.002283 | 0.026244 | 19 |
| GO:0002367 | cytokine production involved in immune response | 14/1207 | 0.002298 | 0.026244 | 14 |
| GO:0007631 | feeding behavior | 14/1207 | 0.002298 | 0.026244 | 14 |
| GO:0071706 | tumor necrosis factor superfamily cytokine production | 21/1207 | 0.002313 | 0.026244 | 21 |
| GO:0001755 | neural crest cell migration | 10/1207 | 0.002313 | 0.026244 | 10 |
| GO:0045620 | negative regulation of lymphocyte differentiation | 10/1207 | 0.002313 | 0.026244 | 10 |
| GO:2000300 | regulation of synaptic vesicle exocytosis | 10/1207 | 0.002313 | 0.026244 | 10 |
| GO:0030336 | negative regulation of cell migration | 35/1207 | 0.002317 | 0.026244 | 35 |
| GO:0007623 | circadian rhythm | 25/1207 | 0.002317 | 0.026244 | 25 |
| GO:0009306 | protein secretion | 38/1207 | 0.002365 | 0.026727 | 38 |
| GO:0050920 | regulation of chemotaxis | 26/1207 | 0.002394 | 0.026855 | 26 |
| GO:0038179 | neurotrophin signaling pathway | 8/1207 | 0.002396 | 0.026855 | 8 |
| GO:0045214 | sarcomere organization | 8/1207 | 0.002396 | 0.026855 | 8 |
| GO:2000249 | regulation of actin cytoskeleton reorganization | 8/1207 | 0.002396 | 0.026855 | 8 |
| GO:0043280 | positive regulation of cysteine-type endopeptidase activity involved in apoptotic process | 18/1207 | 0.002423 | 0.027053 | 18 |
| GO:0050671 | positive regulation of lymphocyte proliferation | 18/1207 | 0.002423 | 0.027053 | 18 |
| GO:0019932 | second-messenger-mediated signaling | 33/1207 | 0.002456 | 0.027363 | 33 |
| GO:0035592 | establishment of protein localization to extracellular region | 38/1207 | 0.002479 | 0.027566 | 38 |
| GO:0046883 | regulation of hormone secretion | 29/1207 | 0.002543 | 0.028166 | 29 |
| GO:0045779 | negative regulation of bone resorption | 5/1207 | 0.002564 | 0.028166 | 5 |
| GO:0051709 | regulation of killing of cells of other organism | 5/1207 | 0.002564 | 0.028166 | 5 |
| GO:0035860 | glial cell-derived neurotrophic factor receptor signaling pathway | 4/1207 | 0.002564 | 0.028166 | 4 |
| GO:0039530 | MDA-5 signaling pathway | 4/1207 | 0.002564 | 0.028166 | 4 |
| GO:0043301 | negative regulation of leukocyte degranulation | 4/1207 | 0.002564 | 0.028166 | 4 |
| GO:0009409 | response to cold | 9/1207 | 0.002614 | 0.02848 | 9 |
| GO:0032480 | negative regulation of type I interferon production | 9/1207 | 0.002614 | 0.02848 | 9 |
| GO:0070169 | positive regulation of biomineral tissue development | 9/1207 | 0.002614 | 0.02848 | 9 |
| GO:1903053 | regulation of extracellular matrix organization | 9/1207 | 0.002614 | 0.02848 | 9 |
| GO:0021544 | subpallium development | 6/1207 | 0.002663 | 0.028751 | 6 |
| GO:0036499 | PERK-mediated unfolded protein response | 6/1207 | 0.002663 | 0.028751 | 6 |
| GO:0046135 | pyrimidine nucleoside catabolic process | 6/1207 | 0.002663 | 0.028751 | 6 |
| GO:0050951 | sensory perception of temperature stimulus | 6/1207 | 0.002663 | 0.028751 | 6 |
| GO:2000146 | negative regulation of cell motility | 36/1207 | 0.002665 | 0.028751 | 36 |
| GO:0048593 | camera-type eye morphogenesis | 16/1207 | 0.00267 | 0.028751 | 16 |
| GO:0030203 | glycosaminoglycan metabolic process | 20/1207 | 0.002685 | 0.0288 | 20 |
| GO:0044106 | cellular amine metabolic process | 20/1207 | 0.002685 | 0.0288 | 20 |
| GO:0046890 | regulation of lipid biosynthetic process | 24/1207 | 0.00271 | 0.029006 | 24 |
| GO:1903707 | negative regulation of hemopoiesis | 15/1207 | 0.00276 | 0.02949 | 15 |
| GO:0010522 | regulation of calcium ion transport into cytosol | 14/1207 | 0.002813 | 0.02996 | 14 |
| GO:0048041 | focal adhesion assembly | 13/1207 | 0.002815 | 0.02996 | 13 |
| GO:0030501 | positive regulation of bone mineralization | 8/1207 | 0.002848 | 0.030233 | 8 |
| GO:0003158 | endothelium development | 18/1207 | 0.002852 | 0.030233 | 18 |
| GO:0002573 | myeloid leukocyte differentiation | 24/1207 | 0.00289 | 0.030537 | 24 |
| GO:0051092 | positive regulation of NF-kappaB transcription factor activity | 20/1207 | 0.002892 | 0.030537 | 20 |
| GO:0002230 | positive regulation of defense response to virus by host | 7/1207 | 0.002919 | 0.030648 | 7 |
| GO:0032228 | regulation of synaptic transmission GABAergic | 7/1207 | 0.002919 | 0.030648 | 7 |
| GO:0034405 | response to fluid shear stress | 7/1207 | 0.002919 | 0.030648 | 7 |
| GO:0050731 | positive regulation of peptidyl-tyrosine phosphorylation | 23/1207 | 0.002968 | 0.0311 | 23 |
| GO:0043090 | amino acid import | 9/1207 | 0.003038 | 0.031593 | 9 |
| GO:0043330 | response to exogenous dsRNA | 9/1207 | 0.003038 | 0.031593 | 9 |
| GO:0110151 | positive regulation of biomineralization | 9/1207 | 0.003038 | 0.031593 | 9 |
| GO:2001238 | positive regulation of extrinsic apoptotic signaling pathway | 9/1207 | 0.003038 | 0.031593 | 9 |
| GO:0001889 | liver development | 18/1207 | 0.003088 | 0.031999 | 18 |
| GO:0098754 | detoxification | 18/1207 | 0.003088 | 0.031999 | 18 |
| GO:1901890 | positive regulation of cell junction assembly | 14/1207 | 0.003104 | 0.032105 | 14 |
| GO:0002576 | platelet degranulation | 17/1207 | 0.003286 | 0.033867 | 17 |
| GO:0071375 | cellular response to peptide hormone stimulus | 34/1207 | 0.003305 | 0.033867 | 34 |
| GO:0002228 | natural killer cell mediated immunity | 11/1207 | 0.003306 | 0.033867 | 11 |
| GO:0038034 | signal transduction in absence of ligand | 11/1207 | 0.003306 | 0.033867 | 11 |
| GO:0097192 | extrinsic apoptotic signaling pathway in absence of ligand | 11/1207 | 0.003306 | 0.033867 | 11 |
| GO:0045927 | positive regulation of growth | 28/1207 | 0.003335 | 0.033956 | 28 |
| GO:0003094 | glomerular filtration | 6/1207 | 0.003361 | 0.033956 | 6 |
| GO:0045662 | negative regulation of myoblast differentiation | 6/1207 | 0.003361 | 0.033956 | 6 |
| GO:0062149 | detection of stimulus involved in sensory perception of pain | 6/1207 | 0.003361 | 0.033956 | 6 |
| GO:1903901 | negative regulation of viral life cycle | 6/1207 | 0.003361 | 0.033956 | 6 |
| GO:0014047 | glutamate secretion | 8/1207 | 0.003364 | 0.033956 | 8 |
| GO:0051281 | positive regulation of release of sequestered calcium ion into cytosol | 8/1207 | 0.003364 | 0.033956 | 8 |
| GO:0072529 | pyrimidine-containing compound catabolic process | 8/1207 | 0.003364 | 0.033956 | 8 |
| GO:0042098 | T cell proliferation | 23/1207 | 0.003381 | 0.034001 | 23 |
| GO:1901215 | negative regulation of neuron death | 23/1207 | 0.003381 | 0.034001 | 23 |
| GO:0060191 | regulation of lipase activity | 14/1207 | 0.00342 | 0.034062 | 14 |
| GO:0032481 | positive regulation of type I interferon production | 12/1207 | 0.003437 | 0.034062 | 12 |
| GO:0048678 | response to axon injury | 12/1207 | 0.003437 | 0.034062 | 12 |
| GO:0006577 | amino-acid betaine metabolic process | 5/1207 | 0.003443 | 0.034062 | 5 |
| GO:0010715 | regulation of extracellular matrix disassembly | 5/1207 | 0.003443 | 0.034062 | 5 |
| GO:0021756 | striatum development | 5/1207 | 0.003443 | 0.034062 | 5 |
| GO:0048791 | calcium ion-regulated exocytosis of neurotransmitter | 5/1207 | 0.003443 | 0.034062 | 5 |
| GO:0060192 | negative regulation of lipase activity | 5/1207 | 0.003443 | 0.034062 | 5 |
| GO:2001267 | regulation of cysteine-type endopeptidase activity involved in apoptotic signaling pathway | 5/1207 | 0.003443 | 0.034062 | 5 |
| GO:0051928 | positive regulation of calcium ion transport | 16/1207 | 0.003471 | 0.034275 | 16 |
| GO:0001961 | positive regulation of cytokine-mediated signaling pathway | 9/1207 | 0.003514 | 0.034581 | 9 |
| GO:0019359 | nicotinamide nucleotide biosynthetic process | 7/1207 | 0.003533 | 0.034581 | 7 |
| GO:0019363 | pyridine nucleotide biosynthetic process | 7/1207 | 0.003533 | 0.034581 | 7 |
| GO:0035767 | endothelial cell chemotaxis | 7/1207 | 0.003533 | 0.034581 | 7 |
| GO:0098801 | regulation of renal system process | 7/1207 | 0.003533 | 0.034581 | 7 |
| GO:0061008 | hepaticobiliary system development | 18/1207 | 0.003611 | 0.03528 | 18 |
| GO:0048762 | mesenchymal cell differentiation | 26/1207 | 0.003647 | 0.035571 | 26 |
| GO:0090257 | regulation of muscle system process | 27/1207 | 0.003695 | 0.035975 | 27 |
| GO:0038061 | NIK/NF-kappaB signaling | 22/1207 | 0.003706 | 0.036015 | 22 |
| GO:0051937 | catecholamine transport | 11/1207 | 0.003721 | 0.036095 | 11 |
| GO:0019216 | regulation of lipid metabolic process | 40/1207 | 0.003746 | 0.036273 | 40 |
| GO:0032736 | positive regulation of interleukin-13 production | 4/1207 | 0.003827 | 0.036732 | 4 |
| GO:0035457 | cellular response to interferon-alpha | 4/1207 | 0.003827 | 0.036732 | 4 |
| GO:0070142 | synaptic vesicle budding | 4/1207 | 0.003827 | 0.036732 | 4 |
| GO:0070673 | response to interleukin-18 | 4/1207 | 0.003827 | 0.036732 | 4 |
| GO:1990440 | positive regulation of transcription from RNA polymerase II promoter in response to endoplasmic reticulum stress | 4/1207 | 0.003827 | 0.036732 | 4 |
| GO:1904064 | positive regulation of cation transmembrane transport | 18/1207 | 0.003899 | 0.037356 | 18 |
| GO:0050691 | regulation of defense response to virus by host | 8/1207 | 0.003949 | 0.037647 | 8 |
| GO:0072524 | pyridine-containing compound metabolic process | 8/1207 | 0.003949 | 0.037647 | 8 |
| GO:0071219 | cellular response to molecule of bacterial origin | 24/1207 | 0.003949 | 0.037647 | 24 |
| GO:0032963 | collagen metabolic process | 15/1207 | 0.00397 | 0.037715 | 15 |
| GO:1902414 | protein localization to cell junction | 15/1207 | 0.00397 | 0.037715 | 15 |
| GO:0051897 | positive regulation of protein kinase B signaling | 21/1207 | 0.004056 | 0.038391 | 21 |
| GO:0052126 | movement in host environment | 21/1207 | 0.004056 | 0.038391 | 21 |
| GO:0019722 | calcium-mediated signaling | 23/1207 | 0.004091 | 0.038656 | 23 |
| GO:1990138 | neuron projection extension | 20/1207 | 0.004134 | 0.039001 | 20 |
| GO:0021675 | nerve development | 11/1207 | 0.004176 | 0.03916 | 11 |
| GO:0050688 | regulation of defense response to virus | 11/1207 | 0.004176 | 0.03916 | 11 |
| GO:0030206 | chondroitin sulfate biosynthetic process | 6/1207 | 0.004187 | 0.03916 | 6 |
| GO:0060544 | regulation of necroptotic process | 6/1207 | 0.004187 | 0.03916 | 6 |
| GO:0097205 | renal filtration | 6/1207 | 0.004187 | 0.03916 | 6 |
| GO:0071214 | cellular response to abiotic stimulus | 34/1207 | 0.004201 | 0.03916 | 34 |
| GO:0104004 | cellular response to environmental stimulus | 34/1207 | 0.004201 | 0.03916 | 34 |
| GO:0032728 | positive regulation of interferon-beta production | 7/1207 | 0.004241 | 0.039398 | 7 |
| GO:1905332 | positive regulation of morphogenesis of an epithelium | 7/1207 | 0.004241 | 0.039398 | 7 |
| GO:0048608 | reproductive structure development | 40/1207 | 0.004256 | 0.039441 | 40 |
| GO:0015844 | monoamine transport | 12/1207 | 0.00426 | 0.039441 | 12 |
| GO:0042391 | regulation of membrane potential | 42/1207 | 0.004387 | 0.040548 | 42 |
| GO:0034101 | erythrocyte homeostasis | 16/1207 | 0.004459 | 0.040928 | 16 |
| GO:0032722 | positive regulation of chemokine production | 10/1207 | 0.004466 | 0.040928 | 10 |
| GO:0045576 | mast cell activation | 10/1207 | 0.004466 | 0.040928 | 10 |
| GO:0061098 | positive regulation of protein tyrosine kinase activity | 10/1207 | 0.004466 | 0.040928 | 10 |
| GO:0014065 | phosphatidylinositol 3-kinase signaling | 19/1207 | 0.004497 | 0.040928 | 19 |
| GO:0046434 | organophosphate catabolic process | 19/1207 | 0.004497 | 0.040928 | 19 |
| GO:0060541 | respiratory system development | 22/1207 | 0.004499 | 0.040928 | 22 |
| GO:0002281 | macrophage activation involved in immune response | 5/1207 | 0.004519 | 0.040928 | 5 |
| GO:0060546 | negative regulation of necroptotic process | 5/1207 | 0.004519 | 0.040928 | 5 |
| GO:0071360 | cellular response to exogenous dsRNA | 5/1207 | 0.004519 | 0.040928 | 5 |
| GO:2000831 | regulation of steroid hormone secretion | 5/1207 | 0.004519 | 0.040928 | 5 |
| GO:0007044 | cell-substrate junction assembly | 14/1207 | 0.004525 | 0.040928 | 14 |
| GO:0060079 | excitatory postsynaptic potential | 14/1207 | 0.004525 | 0.040928 | 14 |
| GO:0031346 | positive regulation of cell projection organization | 35/1207 | 0.004536 | 0.040959 | 35 |
| GO:0071604 | transforming growth factor beta production | 8/1207 | 0.004611 | 0.041563 | 8 |
| GO:0032964 | collagen biosynthetic process | 9/1207 | 0.004641 | 0.041703 | 9 |
| GO:1903307 | positive regulation of regulated secretory pathway | 9/1207 | 0.004641 | 0.041703 | 9 |
| GO:0010631 | epithelial cell migration | 36/1207 | 0.004657 | 0.041775 | 36 |
| GO:1903035 | negative regulation of response to wounding | 13/1207 | 0.004665 | 0.041783 | 13 |
| GO:1904427 | positive regulation of calcium ion transmembrane transport | 11/1207 | 0.004675 | 0.041804 | 11 |
| GO:1905954 | positive regulation of lipid localization | 15/1207 | 0.004721 | 0.042142 | 15 |
| GO:0061458 | reproductive system development | 40/1207 | 0.004824 | 0.042993 | 40 |
| GO:1902106 | negative regulation of leukocyte differentiation | 14/1207 | 0.004952 | 0.044062 | 14 |
| GO:0044060 | regulation of endocrine process | 7/1207 | 0.005051 | 0.044797 | 7 |
| GO:0046640 | regulation of alpha-beta T cell proliferation | 7/1207 | 0.005051 | 0.044797 | 7 |
| GO:0060560 | developmental growth involved in morphogenesis | 25/1207 | 0.005083 | 0.045005 | 25 |
| GO:0045664 | regulation of neuron differentiation | 22/1207 | 0.005103 | 0.045114 | 22 |
| GO:0001823 | mesonephros development | 13/1207 | 0.005132 | 0.04512 | 13 |
| GO:0007015 | actin filament organization | 42/1207 | 0.005152 | 0.04512 | 42 |
| GO:0006972 | hyperosmotic response | 6/1207 | 0.005153 | 0.04512 | 6 |
| GO:0009651 | response to salt stress | 6/1207 | 0.005153 | 0.04512 | 6 |
| GO:0010460 | positive regulation of heart rate | 6/1207 | 0.005153 | 0.04512 | 6 |
| GO:0031342 | negative regulation of cell killing | 6/1207 | 0.005153 | 0.04512 | 6 |
| GO:1903409 | reactive oxygen species biosynthetic process | 16/1207 | 0.005238 | 0.045785 | 16 |
| GO:0002711 | positive regulation of T cell mediated immunity | 9/1207 | 0.005301 | 0.046119 | 9 |
| GO:0002820 | negative regulation of adaptive immune response | 9/1207 | 0.005301 | 0.046119 | 9 |
| GO:0002886 | regulation of myeloid leukocyte mediated immunity | 9/1207 | 0.005301 | 0.046119 | 9 |
| GO:0090132 | epithelium migration | 36/1207 | 0.005317 | 0.046184 | 36 |
| GO:0046677 | response to antibiotic | 8/1207 | 0.005353 | 0.046327 | 8 |
| GO:2000273 | positive regulation of signaling receptor activity | 8/1207 | 0.005353 | 0.046327 | 8 |
| GO:0050730 | regulation of peptidyl-tyrosine phosphorylation | 28/1207 | 0.005415 | 0.046327 | 28 |
| GO:0042129 | regulation of T cell proliferation | 20/1207 | 0.005422 | 0.046327 | 20 |
| GO:0050767 | regulation of neurogenesis | 35/1207 | 0.005432 | 0.046327 | 35 |
| GO:0014831 | gastro-intestinal system smooth muscle contraction | 4/1207 | 0.005452 | 0.046327 | 4 |
| GO:0030208 | dermatan sulfate biosynthetic process | 4/1207 | 0.005452 | 0.046327 | 4 |
| GO:0032230 | positive regulation of synaptic transmissionGABAergic | 4/1207 | 0.005452 | 0.046327 | 4 |
| GO:0032494 | response to peptidoglycan | 4/1207 | 0.005452 | 0.046327 | 4 |
| GO:0032908 | regulation of transforming growth factor beta1 production | 4/1207 | 0.005452 | 0.046327 | 4 |
| GO:0036462 | TRAIL-activated apoptotic signaling pathway | 4/1207 | 0.005452 | 0.046327 | 4 |
| GO:0038180 | nerve growth factor signaling pathway | 4/1207 | 0.005452 | 0.046327 | 4 |
| GO:0042178 | xenobiotic catabolic process | 4/1207 | 0.005452 | 0.046327 | 4 |
| GO:0061517 | macrophage proliferation | 4/1207 | 0.005452 | 0.046327 | 4 |
| GO:0009267 | cellular response to starvation | 19/1207 | 0.005545 | 0.047023 | 19 |
| GO:0034599 | cellular response to oxidative stress | 31/1207 | 0.005551 | 0.047023 | 31 |
| GO:0062012 | regulation of small molecule metabolic process | 42/1207 | 0.005574 | 0.04715 | 42 |
| GO:0046887 | positive regulation of hormone secretion | 16/1207 | 0.005666 | 0.047715 | 16 |
| GO:0032613 | interleukin-10 production | 10/1207 | 0.005676 | 0.047715 | 10 |
| GO:0032835 | glomerulus development | 10/1207 | 0.005676 | 0.047715 | 10 |
| GO:0042698 | ovulation cycle | 10/1207 | 0.005676 | 0.047715 | 10 |
| GO:0007413 | axonal fasciculation | 5/1207 | 0.005814 | 0.048578 | 5 |
| GO:0007620 | copulation | 5/1207 | 0.005814 | 0.048578 | 5 |
| GO:0062099 | negative regulation of programmed necrotic cell death | 5/1207 | 0.005814 | 0.048578 | 5 |
| GO:0106030 | neuron projection fasciculation | 5/1207 | 0.005814 | 0.048578 | 5 |
| GO:0050807 | regulation of synapse organization | 23/1207 | 0.005886 | 0.049106 | 23 |
| GO:0007200 | phospholipase C-activating G protein-coupled receptor signaling pathway | 14/1207 | 0.005903 | 0.049174 | 14 |
| GO:0022409 | positive regulation of cell-cell adhesion | 29/1207 | 0.005956 | 0.049444 | 29 |
| GO:0019362 | pyridine nucleotide metabolic process | 7/1207 | 0.005972 | 0.049444 | 7 |
| GO:0046496 | nicotinamide nucleotide metabolic process | 7/1207 | 0.005972 | 0.049444 | 7 |
| GO:0072525 | pyridine-containing compound biosynthetic process | 7/1207 | 0.005972 | 0.049444 | 7 |
| GO:0009266 | response to temperature stimulus | 25/1207 | 0.006012 | 0.049698 | 25 |

**Supplementary Table 12. The GO-BP enrichment of up-regulated DEGs in HK-2 cells of 24h**

| ID | Description | GeneRatio | pvalue | p.adjust | Count |
| --- | --- | --- | --- | --- | --- |
| GO:0009615 | response to virus | 68/707 | 4.97E-29 | 1.40E-25 | 68 |
| GO:0051607 | defense response to virus | 58/707 | 8.75E-29 | 1.40E-25 | 58 |
| GO:0140546 | defense response to symbiont | 58/707 | 8.75E-29 | 1.40E-25 | 58 |
| GO:0060337 | type I interferon signaling pathway | 35/707 | 7.67E-26 | 9.19E-23 | 35 |
| GO:0071357 | cellular response to type I interferon | 35/707 | 1.17E-25 | 1.12E-22 | 35 |
| GO:0034340 | response to type I interferon | 35/707 | 8.66E-25 | 6.91E-22 | 35 |
| GO:0034341 | response to interferon-gamma | 39/707 | 8.59E-18 | 5.88E-15 | 39 |
| GO:0048525 | negative regulation of viral process | 26/707 | 1.18E-16 | 7.04E-14 | 26 |
| GO:0045071 | negative regulation of viral genome replication | 20/707 | 2.23E-15 | 1.19E-12 | 20 |
| GO:0071346 | cellular response to interferon-gamma | 34/707 | 2.91E-15 | 1.39E-12 | 34 |
| GO:0060333 | interferon-gamma-mediated signaling pathway | 24/707 | 2.25E-14 | 9.81E-12 | 24 |
| GO:1903900 | regulation of viral life cycle | 30/707 | 2.83E-14 | 1.13E-11 | 30 |
| GO:0045069 | regulation of viral genome replication | 22/707 | 2.45E-13 | 9.03E-11 | 22 |
| GO:0002237 | response to molecule of bacterial origin | 45/707 | 3.19E-13 | 1.09E-10 | 45 |
| GO:0002831 | regulation of response to biotic stimulus | 50/707 | 4.71E-13 | 1.50E-10 | 50 |
| GO:0032496 | response to lipopolysaccharide | 43/707 | 6.70E-13 | 2.01E-10 | 43 |
| GO:0001819 | positive regulation of cytokine production | 50/707 | 2.13E-12 | 5.99E-10 | 50 |
| GO:0043903 | regulation of biological process involved in symbiotic interaction | 32/707 | 2.36E-12 | 6.28E-10 | 32 |
| GO:0050792 | regulation of viral process | 31/707 | 2.60E-12 | 6.55E-10 | 31 |
| GO:0019079 | viral genome replication | 24/707 | 7.29E-11 | 1.75E-08 | 24 |
| GO:0045088 | regulation of innate immune response | 38/707 | 2.17E-10 | 4.94E-08 | 38 |
| GO:0060326 | cell chemotaxis | 37/707 | 3.52E-10 | 7.66E-08 | 37 |
| GO:0071621 | granulocyte chemotaxis | 22/707 | 1.16E-09 | 2.42E-07 | 22 |
| GO:0034612 | response to tumor necrosis factor | 37/707 | 1.24E-09 | 2.48E-07 | 37 |
| GO:0045824 | negative regulation of innate immune response | 16/707 | 1.50E-09 | 2.87E-07 | 16 |
| GO:0050777 | negative regulation of immune response | 24/707 | 1.82E-09 | 3.35E-07 | 24 |
| GO:0043122 | regulation of I-kappaB kinase/NF-kappaB signaling | 31/707 | 2.89E-09 | 5.14E-07 | 31 |
| GO:0043123 | positive regulation of I-kappaB kinase/NF-kappaB signaling | 25/707 | 2.06E-08 | 3.52E-06 | 25 |
| GO:0071356 | cellular response to tumor necrosis factor | 33/707 | 2.41E-08 | 3.99E-06 | 33 |
| GO:0007249 | I-kappaB kinase/NF-kappaB signaling | 32/707 | 2.58E-08 | 4.12E-06 | 32 |
| GO:0030595 | leukocyte chemotaxis | 28/707 | 2.93E-08 | 4.53E-06 | 28 |
| GO:0097530 | granulocyte migration | 22/707 | 3.42E-08 | 5.12E-06 | 22 |
| GO:0097191 | extrinsic apoptotic signaling pathway | 27/707 | 4.73E-08 | 6.87E-06 | 27 |
| GO:0002697 | regulation of immune effector process | 43/707 | 5.12E-08 | 7.21E-06 | 43 |
| GO:0002683 | negative regulation of immune system process | 39/707 | 6.52E-08 | 8.93E-06 | 39 |
| GO:0019885 | antigen processing and presentation of endogenous peptide antigen via MHC class I | 8/707 | 6.75E-08 | 8.98E-06 | 8 |
| GO:0031349 | positive regulation of defense response | 36/707 | 9.95E-08 | 1.26E-05 | 36 |
| GO:0002819 | regulation of adaptive immune response | 23/707 | 1.00E-07 | 1.26E-05 | 23 |
| GO:0035456 | response to interferon-beta | 10/707 | 1.12E-07 | 1.37E-05 | 10 |
| GO:0019058 | viral life cycle | 35/707 | 1.22E-07 | 1.46E-05 | 35 |
| GO:0003018 | vascular process in circulatory system | 28/707 | 1.65E-07 | 1.93E-05 | 28 |
| GO:0002832 | negative regulation of response to biotic stimulus | 17/707 | 1.71E-07 | 1.95E-05 | 17 |
| GO:0060759 | regulation of response to cytokine stimulus | 24/707 | 1.79E-07 | 1.99E-05 | 24 |
| GO:0097529 | myeloid leukocyte migration | 26/707 | 1.96E-07 | 2.09E-05 | 26 |
| GO:0002483 | antigen processing and presentation of endogenous peptide antigen | 8/707 | 1.96E-07 | 2.09E-05 | 8 |
| GO:0042590 | antigen processing and presentation of exogenous peptide antigen via MHC class I | 15/707 | 2.41E-07 | 2.51E-05 | 15 |
| GO:0030593 | neutrophil chemotaxis | 17/707 | 2.66E-07 | 2.71E-05 | 17 |
| GO:0032479 | regulation of type I interferon production | 19/707 | 2.99E-07 | 2.99E-05 | 19 |
| GO:0032606 | type I interferon production | 19/707 | 3.39E-07 | 3.31E-05 | 19 |
| GO:0070663 | regulation of leukocyte proliferation | 27/707 | 4.09E-07 | 3.92E-05 | 27 |
| GO:0032675 | regulation of interleukin-6 production | 21/707 | 4.84E-07 | 4.55E-05 | 21 |
| GO:0035455 | response to interferon-alpha | 8/707 | 4.94E-07 | 4.55E-05 | 8 |
| GO:0002479 | antigen processing and presentation of exogenous peptide antigen via MHC class I TAP-dependent | 14/707 | 6.41E-07 | 5.79E-05 | 14 |
| GO:0071216 | cellular response to biotic stimulus | 26/707 | 7.23E-07 | 6.41E-05 | 26 |
| GO:0032635 | interleukin-6 production | 21/707 | 7.37E-07 | 6.42E-05 | 21 |
| GO:0071674 | mononuclear cell migration | 23/707 | 7.58E-07 | 6.49E-05 | 23 |
| GO:0051090 | regulation of DNA-binding transcription factor activity | 39/707 | 7.99E-07 | 6.72E-05 | 39 |
| GO:0009308 | amine metabolic process | 21/707 | 8.17E-07 | 6.75E-05 | 21 |
| GO:0070661 | leukocyte proliferation | 31/707 | 8.32E-07 | 6.76E-05 | 31 |
| GO:0051091 | positive regulation of DNA-binding transcription factor activity | 28/707 | 8.96E-07 | 7.16E-05 | 28 |
| GO:0072507 | divalent inorganic cation homeostasis | 42/707 | 9.33E-07 | 7.33E-05 | 42 |
| GO:0032944 | regulation of mononuclear cell proliferation | 25/707 | 1.08E-06 | 8.35E-05 | 25 |
| GO:0007159 | leukocyte cell-cell adhesion | 34/707 | 1.17E-06 | 8.88E-05 | 34 |
| GO:2001236 | regulation of extrinsic apoptotic signaling pathway | 20/707 | 1.31E-06 | 9.80E-05 | 20 |
| GO:0071496 | cellular response to external stimulus | 30/707 | 1.34E-06 | 9.91E-05 | 30 |
| GO:1902041 | regulation of extrinsic apoptotic signaling pathway via death domain receptors | 12/707 | 1.56E-06 | 0.000113 | 12 |
| GO:1905523 | positive regulation of macrophage migration | 8/707 | 1.62E-06 | 0.000116 | 8 |
| GO:0007204 | positive regulation of cytosolic calcium ion concentration | 30/707 | 2.15E-06 | 0.000152 | 30 |
| GO:0009612 | response to mechanical stimulus | 23/707 | 2.21E-06 | 0.000154 | 23 |
| GO:0051480 | regulation of cytosolic calcium ion concentration | 32/707 | 2.33E-06 | 0.000159 | 32 |
| GO:0033209 | tumor necrosis factor-mediated signaling pathway | 21/707 | 2.39E-06 | 0.000159 | 21 |
| GO:0002687 | positive regulation of leukocyte migration | 18/707 | 2.42E-06 | 0.000159 | 18 |
| GO:0050729 | positive regulation of inflammatory response | 18/707 | 2.42E-06 | 0.000159 | 18 |
| GO:0048872 | homeostasis of number of cells | 26/707 | 2.54E-06 | 0.000165 | 26 |
| GO:0002718 | regulation of cytokine production involved in immune response | 14/707 | 2.66E-06 | 0.00017 | 14 |
| GO:1903037 | regulation of leukocyte cell-cell adhesion | 31/707 | 2.73E-06 | 0.000172 | 31 |
| GO:0031348 | negative regulation of defense response | 25/707 | 3.05E-06 | 0.000188 | 25 |
| GO:0070555 | response to interleukin-1 | 23/707 | 3.10E-06 | 0.000188 | 23 |
| GO:0050670 | regulation of lymphocyte proliferation | 24/707 | 3.10E-06 | 0.000188 | 24 |
| GO:0001959 | regulation of cytokine-mediated signaling pathway | 21/707 | 3.15E-06 | 0.000189 | 21 |
| GO:0019883 | antigen processing and presentation of endogenous antigen | 8/707 | 3.21E-06 | 0.00019 | 8 |
| GO:1990266 | neutrophil migration | 17/707 | 3.43E-06 | 0.0002 | 17 |
| GO:0032943 | mononuclear cell proliferation | 28/707 | 3.50E-06 | 0.000202 | 28 |
| GO:0002703 | regulation of leukocyte mediated immunity | 23/707 | 3.96E-06 | 0.000226 | 23 |
| GO:0002474 | antigen processing and presentation of peptide antigen via MHC class I | 15/707 | 4.04E-06 | 0.000227 | 15 |
| GO:0150104 | transport across blood-brain barrier | 14/707 | 4.08E-06 | 0.000227 | 14 |
| GO:0048265 | response to pain | 8/707 | 4.41E-06 | 0.000243 | 8 |
| GO:0010232 | vascular transport | 14/707 | 4.69E-06 | 0.000255 | 14 |
| GO:0071222 | cellular response to lipopolysaccharide | 22/707 | 5.04E-06 | 0.000271 | 22 |
| GO:0072503 | cellular divalent inorganic cation homeostasis | 39/707 | 5.32E-06 | 0.000283 | 39 |
| GO:0002822 | regulation of adaptive immune response based on somatic recombination of immune receptors built from immunoglobulin superfamily domains | 19/707 | 5.69E-06 | 0.0003 | 19 |
| GO:0002709 | regulation of T cell mediated immunity | 13/707 | 6.02E-06 | 0.000314 | 13 |
| GO:2000116 | regulation of cysteine-type endopeptidase activity | 24/707 | 6.21E-06 | 0.00032 | 24 |
| GO:0010043 | response to zinc ion | 11/707 | 7.26E-06 | 0.00037 | 11 |
| GO:0044106 | cellular amine metabolic process | 19/707 | 7.55E-06 | 0.000381 | 19 |
| GO:0051092 | positive regulation of NF-kappaB transcription factor activity | 19/707 | 8.28E-06 | 0.000413 | 19 |
| GO:0046651 | lymphocyte proliferation | 27/707 | 8.40E-06 | 0.000415 | 27 |
| GO:0050727 | regulation of inflammatory response | 32/707 | 8.53E-06 | 0.000417 | 32 |
| GO:0055074 | calcium ion homeostasis | 37/707 | 8.65E-06 | 0.000419 | 37 |
| GO:0002367 | cytokine production involved in immune response | 14/707 | 9.09E-06 | 0.000436 | 14 |
| GO:0052372 | modulation by symbiont of entry into host | 10/707 | 9.24E-06 | 0.000438 | 10 |
| GO:0002685 | regulation of leukocyte migration | 22/707 | 9.59E-06 | 0.000451 | 22 |
| GO:0070665 | positive regulation of leukocyte proliferation | 18/707 | 1.11E-05 | 0.000518 | 18 |
| GO:0098586 | cellular response to virus | 12/707 | 1.18E-05 | 0.000543 | 12 |
| GO:1901654 | response to ketone | 21/707 | 1.23E-05 | 0.000561 | 21 |
| GO:0071219 | cellular response to molecule of bacterial origin | 22/707 | 1.30E-05 | 0.00059 | 22 |
| GO:0032946 | positive regulation of mononuclear cell proliferation | 17/707 | 1.35E-05 | 0.000604 | 17 |
| GO:0097421 | liver regeneration | 8/707 | 1.37E-05 | 0.000609 | 8 |
| GO:0008625 | extrinsic apoptotic signaling pathway via death domain receptors | 13/707 | 1.39E-05 | 0.000611 | 13 |
| GO:0071347 | cellular response to interleukin-1 | 20/707 | 1.45E-05 | 0.00063 | 20 |
| GO:0016338 | calcium-independent cell-cell adhesion via plasma membrane cell-adhesion molecules | 7/707 | 1.46E-05 | 0.000632 | 7 |
| GO:1990868 | response to chemokine | 14/707 | 1.49E-05 | 0.000633 | 14 |
| GO:1990869 | cellular response to chemokine | 14/707 | 1.49E-05 | 0.000633 | 14 |
| GO:0046850 | regulation of bone remodeling | 10/707 | 1.63E-05 | 0.000678 | 10 |
| GO:0033003 | regulation of mast cell activation | 9/707 | 1.66E-05 | 0.000678 | 9 |
| GO:0046596 | regulation of viral entry into host cell | 9/707 | 1.66E-05 | 0.000678 | 9 |
| GO:1902042 | negative regulation of extrinsic apoptotic signaling pathway via death domain receptors | 9/707 | 1.66E-05 | 0.000678 | 9 |
| GO:0071675 | regulation of mononuclear cell migration | 15/707 | 1.68E-05 | 0.000683 | 15 |
| GO:0002695 | negative regulation of leukocyte activation | 20/707 | 1.70E-05 | 0.000686 | 20 |
| GO:0032673 | regulation of interleukin-4 production | 8/707 | 1.77E-05 | 0.000707 | 8 |
| GO:0031667 | response to nutrient levels | 36/707 | 1.79E-05 | 0.000709 | 36 |
| GO:0032755 | positive regulation of interleukin-6 production | 13/707 | 1.80E-05 | 0.000709 | 13 |
| GO:0071260 | cellular response to mechanical stimulus | 12/707 | 1.82E-05 | 0.00071 | 12 |
| GO:0007162 | negative regulation of cell adhesion | 27/707 | 1.90E-05 | 0.000734 | 27 |
| GO:0032753 | positive regulation of interleukin-4 production | 7/707 | 2.00E-05 | 0.00076 | 7 |
| GO:0036003 | positive regulation of transcription from RNA polymerase II promoter in response to stress | 7/707 | 2.00E-05 | 0.00076 | 7 |
| GO:0050866 | negative regulation of cell activation | 21/707 | 2.12E-05 | 0.000799 | 21 |
| GO:0038061 | NIK/NF-kappaB signaling | 20/707 | 2.17E-05 | 0.000809 | 20 |
| GO:0022407 | regulation of cell-cell adhesion | 35/707 | 2.18E-05 | 0.000809 | 35 |
| GO:0002724 | regulation of T cell cytokine production | 8/707 | 2.26E-05 | 0.000825 | 8 |
| GO:0032633 | interleukin-4 production | 8/707 | 2.26E-05 | 0.000825 | 8 |
| GO:0042267 | natural killer cell mediated cytotoxicity | 11/707 | 2.31E-05 | 0.000825 | 11 |
| GO:0001818 | negative regulation of cytokine production | 31/707 | 2.31E-05 | 0.000825 | 31 |
| GO:0070098 | chemokine-mediated signaling pathway | 13/707 | 2.32E-05 | 0.000825 | 13 |
| GO:0002704 | negative regulation of leukocyte mediated immunity | 10/707 | 2.33E-05 | 0.000825 | 10 |
| GO:0010759 | positive regulation of macrophage chemotaxis | 6/707 | 2.36E-05 | 0.000825 | 6 |
| GO:0061687 | detoxification of inorganic compound | 6/707 | 2.36E-05 | 0.000825 | 6 |
| GO:0009991 | response to extracellular stimulus | 37/707 | 2.57E-05 | 0.000891 | 37 |
| GO:0032642 | regulation of chemokine production | 13/707 | 2.63E-05 | 0.000896 | 13 |
| GO:0046849 | bone remodeling | 13/707 | 2.63E-05 | 0.000896 | 13 |
| GO:0010038 | response to metal ion | 30/707 | 2.64E-05 | 0.000896 | 30 |
| GO:0046942 | carboxylic acid transport | 26/707 | 2.69E-05 | 0.000901 | 26 |
| GO:0045765 | regulation of angiogenesis | 29/707 | 2.69E-05 | 0.000901 | 29 |
| GO:0032481 | positive regulation of type I interferon production | 12/707 | 2.76E-05 | 0.00091 | 12 |
| GO:0006874 | cellular calcium ion homeostasis | 35/707 | 2.76E-05 | 0.00091 | 35 |
| GO:0060401 | cytosolic calcium ion transport | 19/707 | 2.77E-05 | 0.00091 | 19 |
| GO:2001237 | negative regulation of extrinsic apoptotic signaling pathway | 14/707 | 2.99E-05 | 0.000974 | 14 |
| GO:0048771 | tissue remodeling | 19/707 | 3.25E-05 | 0.001054 | 19 |
| GO:0002690 | positive regulation of leukocyte chemotaxis | 13/707 | 3.35E-05 | 0.001077 | 13 |
| GO:0045953 | negative regulation of natural killer cell mediated cytotoxicity | 6/707 | 3.42E-05 | 0.00108 | 6 |
| GO:0071243 | cellular response to arsenic-containing substance | 6/707 | 3.42E-05 | 0.00108 | 6 |
| GO:0097501 | stress response to metal ion | 6/707 | 3.42E-05 | 0.00108 | 6 |
| GO:0009636 | response to toxic substance | 23/707 | 3.55E-05 | 0.001113 | 23 |
| GO:0002228 | natural killer cell mediated immunity | 11/707 | 3.60E-05 | 0.001113 | 11 |
| GO:1901224 | positive regulation of NIK/NF-kappaB signaling | 11/707 | 3.60E-05 | 0.001113 | 11 |
| GO:0002707 | negative regulation of lymphocyte mediated immunity | 9/707 | 3.66E-05 | 0.001125 | 9 |
| GO:0032102 | negative regulation of response to external stimulus | 32/707 | 3.71E-05 | 0.001129 | 32 |
| GO:1901342 | regulation of vasculature development | 29/707 | 3.72E-05 | 0.001129 | 29 |
| GO:0015849 | organic acid transport | 28/707 | 3.80E-05 | 0.001144 | 28 |
| GO:0060760 | positive regulation of response to cytokine stimulus | 10/707 | 3.84E-05 | 0.00115 | 10 |
| GO:0006953 | acute-phase response | 9/707 | 4.40E-05 | 0.001311 | 9 |
| GO:0001906 | cell killing | 19/707 | 4.45E-05 | 0.001312 | 19 |
| GO:0002369 | T cell cytokine production | 8/707 | 4.46E-05 | 0.001312 | 8 |
| GO:0002526 | acute inflammatory response | 14/707 | 4.60E-05 | 0.001344 | 14 |
| GO:0140289 | protein mono-ADP-ribosylation | 5/707 | 4.64E-05 | 0.001345 | 5 |
| GO:0050671 | positive regulation of lymphocyte proliferation | 16/707 | 4.66E-05 | 0.001345 | 16 |
| GO:0050688 | regulation of defense response to virus | 11/707 | 4.77E-05 | 0.001369 | 11 |
| GO:0002716 | negative regulation of natural killer cell mediated immunity | 6/707 | 4.85E-05 | 0.001383 | 6 |
| GO:0062197 | cellular response to chemical stress | 29/707 | 5.10E-05 | 0.001447 | 29 |
| GO:0032480 | negative regulation of type I interferon production | 9/707 | 5.27E-05 | 0.00148 | 9 |
| GO:2001056 | positive regulation of cysteine-type endopeptidase activity | 17/707 | 5.28E-05 | 0.00148 | 17 |
| GO:0032602 | chemokine production | 13/707 | 5.31E-05 | 0.00148 | 13 |
| GO:0043618 | regulation of transcription from RNA polymerase II promoter in response to stress | 14/707 | 5.66E-05 | 0.001568 | 14 |
| GO:0060402 | calcium ion transport into cytosol | 17/707 | 5.74E-05 | 0.001581 | 17 |
| GO:0032607 | interferon-alpha production | 7/707 | 6.00E-05 | 0.001633 | 7 |
| GO:0032647 | regulation of interferon-alpha production | 7/707 | 6.00E-05 | 0.001633 | 7 |
| GO:1903038 | negative regulation of leukocyte cell-cell adhesion | 16/707 | 6.09E-05 | 0.00165 | 16 |
| GO:0034599 | cellular response to oxidative stress | 26/707 | 6.42E-05 | 0.001728 | 26 |
| GO:0010952 | positive regulation of peptidase activity | 20/707 | 6.62E-05 | 0.001772 | 20 |
| GO:0071548 | response to dexamethasone | 8/707 | 6.75E-05 | 0.001797 | 8 |
| GO:0032615 | interleukin-12 production | 10/707 | 7.10E-05 | 0.00187 | 10 |
| GO:0032655 | regulation of interleukin-12 production | 10/707 | 7.10E-05 | 0.00187 | 10 |
| GO:0045741 | positive regulation of epidermal growth factor-activated receptor activity | 5/707 | 7.31E-05 | 0.001913 | 5 |
| GO:0031341 | regulation of cell killing | 13/707 | 7.38E-05 | 0.001922 | 13 |
| GO:0071622 | regulation of granulocyte chemotaxis | 9/707 | 7.43E-05 | 0.001924 | 9 |
| GO:0002699 | positive regulation of immune effector process | 21/707 | 8.08E-05 | 0.00208 | 21 |
| GO:0046686 | response to cadmium ion | 10/707 | 8.21E-05 | 0.002083 | 10 |
| GO:0070059 | intrinsic apoptotic signaling pathway in response to endoplasmic reticulum stress | 10/707 | 8.21E-05 | 0.002083 | 10 |
| GO:1905521 | regulation of macrophage migration | 8/707 | 8.22E-05 | 0.002083 | 8 |
| GO:1903131 | mononuclear cell differentiation | 32/707 | 8.31E-05 | 0.002096 | 32 |
| GO:0034103 | regulation of tissue remodeling | 12/707 | 8.43E-05 | 0.002116 | 12 |
| GO:0072574 | hepatocyte proliferation | 6/707 | 9.09E-05 | 0.002257 | 6 |
| GO:0072575 | epithelial cell proliferation involved in liver morphogenesis | 6/707 | 9.09E-05 | 0.002257 | 6 |
| GO:0022408 | negative regulation of cell-cell adhesion | 19/707 | 9.31E-05 | 0.00228 | 19 |
| GO:0043281 | regulation of cysteine-type endopeptidase activity involved in apoptotic process | 20/707 | 9.34E-05 | 0.00228 | 20 |
| GO:0042493 | response to drug | 29/707 | 9.34E-05 | 0.00228 | 29 |
| GO:0032760 | positive regulation of tumor necrosis factor production | 12/707 | 9.46E-05 | 0.00228 | 12 |
| GO:0032613 | interleukin-10 production | 10/707 | 9.47E-05 | 0.00228 | 10 |
| GO:0045453 | bone resorption | 10/707 | 9.47E-05 | 0.00228 | 10 |
| GO:0046685 | response to arsenic-containing substance | 7/707 | 9.66E-05 | 0.002315 | 7 |
| GO:0043620 | regulation of DNA-templated transcription in response to stress | 14/707 | 0.000102 | 0.00243 | 14 |
| GO:0043491 | protein kinase B signaling | 24/707 | 0.000102 | 0.00243 | 24 |
| GO:0045089 | positive regulation of innate immune response | 21/707 | 0.000105 | 0.002469 | 21 |
| GO:0032680 | regulation of tumor necrosis factor production | 17/707 | 0.000109 | 0.002564 | 17 |
| GO:0002262 | myeloid cell homeostasis | 16/707 | 0.00011 | 0.002582 | 16 |
| GO:0052547 | regulation of peptidase activity | 34/707 | 0.000112 | 0.002613 | 34 |
| GO:0002221 | pattern recognition receptor signaling pathway | 20/707 | 0.000114 | 0.00264 | 20 |
| GO:0002833 | positive regulation of response to biotic stimulus | 23/707 | 0.000115 | 0.002653 | 23 |
| GO:0050691 | regulation of defense response to virus by host | 8/707 | 0.000119 | 0.002686 | 8 |
| GO:0002711 | positive regulation of T cell mediated immunity | 9/707 | 0.00012 | 0.002686 | 9 |
| GO:0002886 | regulation of myeloid leukocyte mediated immunity | 9/707 | 0.00012 | 0.002686 | 9 |
| GO:0002230 | positive regulation of defense response to virus by host | 7/707 | 0.000121 | 0.002686 | 7 |
| GO:0001911 | negative regulation of leukocyte mediated cytotoxicity | 6/707 | 0.000121 | 0.002686 | 6 |
| GO:0032727 | positive regulation of interferon-alpha production | 6/707 | 0.000121 | 0.002686 | 6 |
| GO:0035458 | cellular response to interferon-beta | 6/707 | 0.000121 | 0.002686 | 6 |
| GO:0072576 | liver morphogenesis | 6/707 | 0.000121 | 0.002686 | 6 |
| GO:0050731 | positive regulation of peptidyl-tyrosine phosphorylation | 19/707 | 0.000123 | 0.002717 | 19 |
| GO:0032640 | tumor necrosis factor production | 17/707 | 0.000127 | 0.002796 | 17 |
| GO:0001894 | tissue homeostasis | 23/707 | 0.000129 | 0.002828 | 23 |
| GO:0015718 | monocarboxylic acid transport | 18/707 | 0.000131 | 0.002844 | 18 |
| GO:2001233 | regulation of apoptotic signaling pathway | 28/707 | 0.000131 | 0.002844 | 28 |
| GO:1903557 | positive regulation of tumor necrosis factor superfamily cytokine production | 12/707 | 0.000132 | 0.002845 | 12 |
| GO:0002456 | T cell mediated immunity | 13/707 | 0.000137 | 0.002927 | 13 |
| GO:0002821 | positive regulation of adaptive immune response | 13/707 | 0.000137 | 0.002927 | 13 |
| GO:1905517 | macrophage migration | 9/707 | 0.00014 | 0.002986 | 9 |
| GO:0042098 | T cell proliferation | 19/707 | 0.000141 | 0.002989 | 19 |
| GO:0006869 | lipid transport | 34/707 | 0.000144 | 0.003045 | 34 |
| GO:1903555 | regulation of tumor necrosis factor superfamily cytokine production | 17/707 | 0.000148 | 0.003107 | 17 |
| GO:0018108 | peptidyl-tyrosine phosphorylation | 29/707 | 0.00015 | 0.003147 | 29 |
| GO:0006979 | response to oxidative stress | 33/707 | 0.000156 | 0.003248 | 33 |
| GO:0046135 | pyrimidine nucleoside catabolic process | 6/707 | 0.000159 | 0.003291 | 6 |
| GO:0052548 | regulation of endopeptidase activity | 32/707 | 0.000161 | 0.003327 | 32 |
| GO:0043331 | response to dsRNA | 9/707 | 0.000163 | 0.00334 | 9 |
| GO:1901655 | cellular response to ketone | 12/707 | 0.000163 | 0.00334 | 12 |
| GO:0043280 | positive regulation of cysteine-type endopeptidase activity involved in apoptotic process | 15/707 | 0.000166 | 0.003385 | 15 |
| GO:0035094 | response to nicotine | 8/707 | 0.00017 | 0.003445 | 8 |
| GO:0018212 | peptidyl-tyrosine modification | 29/707 | 0.000173 | 0.003492 | 29 |
| GO:0010950 | positive regulation of endopeptidase activity | 18/707 | 0.000174 | 0.003494 | 18 |
| GO:0032649 | regulation of interferon-gamma production | 13/707 | 0.000183 | 0.00364 | 13 |
| GO:0032728 | positive regulation of interferon-beta production | 7/707 | 0.000184 | 0.00364 | 7 |
| GO:0035633 | maintenance of blood-brain barrier | 7/707 | 0.000184 | 0.00364 | 7 |
| GO:0071706 | tumor necrosis factor superfamily cytokine production | 17/707 | 0.000184 | 0.00364 | 17 |
| GO:0033555 | multicellular organismal response to stress | 10/707 | 0.000185 | 0.00364 | 10 |
| GO:0042129 | regulation of T cell proliferation | 17/707 | 0.000198 | 0.003888 | 17 |
| GO:0045124 | regulation of bone resorption | 8/707 | 0.000201 | 0.003925 | 8 |
| GO:1903039 | positive regulation of leukocyte cell-cell adhesion | 21/707 | 0.000205 | 0.003987 | 21 |
| GO:0048732 | gland development | 31/707 | 0.000206 | 0.003987 | 31 |
| GO:0015711 | organic anion transport | 29/707 | 0.000207 | 0.003987 | 29 |
| GO:0051250 | negative regulation of lymphocyte activation | 16/707 | 0.000207 | 0.003987 | 16 |
| GO:0070588 | calcium ion transmembrane transport | 25/707 | 0.000212 | 0.004057 | 25 |
| GO:1903706 | regulation of hemopoiesis | 31/707 | 0.000224 | 0.004262 | 31 |
| GO:0046640 | regulation of alpha-beta T cell proliferation | 7/707 | 0.000224 | 0.004262 | 7 |
| GO:0051709 | regulation of killing of cells of other organism | 5/707 | 0.000226 | 0.004276 | 5 |
| GO:0050921 | positive regulation of chemotaxis | 15/707 | 0.00023 | 0.004314 | 15 |
| GO:0055076 | transition metal ion homeostasis | 15/707 | 0.00023 | 0.004314 | 15 |
| GO:0071229 | cellular response to acid chemical | 10/707 | 0.000237 | 0.00443 | 10 |
| GO:0002698 | negative regulation of immune effector process | 14/707 | 0.00025 | 0.004653 | 14 |
| GO:1903522 | regulation of blood circulation | 24/707 | 0.000256 | 0.004755 | 24 |
| GO:0050863 | regulation of T cell activation | 26/707 | 0.000271 | 0.005017 | 26 |
| GO:0002706 | regulation of lymphocyte mediated immunity | 16/707 | 0.000279 | 0.005135 | 16 |
| GO:0032609 | interferon-gamma production | 13/707 | 0.000289 | 0.005286 | 13 |
| GO:0051209 | release of sequestered calcium ion into cytosol | 13/707 | 0.000289 | 0.005286 | 13 |
| GO:0007189 | adenylate cyclase-activating G protein-coupled receptor signaling pathway | 15/707 | 0.000313 | 0.005686 | 15 |
| GO:0048511 | rhythmic process | 24/707 | 0.000313 | 0.005686 | 24 |
| GO:0001909 | leukocyte mediated cytotoxicity | 13/707 | 0.000316 | 0.005686 | 13 |
| GO:0051283 | negative regulation of sequestering of calcium ion | 13/707 | 0.000316 | 0.005686 | 13 |
| GO:0045766 | positive regulation of angiogenesis | 17/707 | 0.000322 | 0.005756 | 17 |
| GO:1904018 | positive regulation of vasculature development | 17/707 | 0.000322 | 0.005756 | 17 |
| GO:0007411 | axon guidance | 23/707 | 0.000326 | 0.005803 | 23 |
| GO:0002824 | positive regulation of adaptive immune response based on somatic recombination of immune receptors built from immunoglobulin superfamily domains | 12/707 | 0.000327 | 0.005812 | 12 |
| GO:0031342 | negative regulation of cell killing | 6/707 | 0.000329 | 0.005812 | 6 |
| GO:0045785 | positive regulation of cell adhesion | 31/707 | 0.000338 | 0.00596 | 31 |
| GO:0097485 | neuron projection guidance | 23/707 | 0.000343 | 0.005978 | 23 |
| GO:0039530 | MDA-5 signaling pathway | 4/707 | 0.000343 | 0.005978 | 4 |
| GO:0043301 | negative regulation of leukocyte degranulation | 4/707 | 0.000343 | 0.005978 | 4 |
| GO:1902105 | regulation of leukocyte differentiation | 23/707 | 0.000361 | 0.006265 | 23 |
| GO:0002478 | antigen processing and presentation of exogenous peptide antigen | 17/707 | 0.000368 | 0.006308 | 17 |
| GO:0032653 | regulation of interleukin-10 production | 9/707 | 0.00037 | 0.006308 | 9 |
| GO:0032722 | positive regulation of chemokine production | 9/707 | 0.00037 | 0.006308 | 9 |
| GO:0045576 | mast cell activation | 9/707 | 0.00037 | 0.006308 | 9 |
| GO:1903670 | regulation of sprouting angiogenesis | 9/707 | 0.00037 | 0.006308 | 9 |
| GO:0050730 | regulation of peptidyl-tyrosine phosphorylation | 22/707 | 0.000373 | 0.006308 | 22 |
| GO:0043330 | response to exogenous dsRNA | 8/707 | 0.000374 | 0.006308 | 8 |
| GO:0051282 | regulation of sequestering of calcium ion | 13/707 | 0.000375 | 0.006308 | 13 |
| GO:0046683 | response to organophosphorus | 14/707 | 0.000375 | 0.006308 | 14 |
| GO:0008016 | regulation of heart contraction | 21/707 | 0.000383 | 0.006411 | 21 |
| GO:0046633 | alpha-beta T cell proliferation | 7/707 | 0.000389 | 0.006496 | 7 |
| GO:0002700 | regulation of production of molecular mediator of immune response | 15/707 | 0.000392 | 0.006522 | 15 |
| GO:0032874 | positive regulation of stress-activated MAPK cascade | 13/707 | 0.000408 | 0.00674 | 13 |
| GO:0010758 | regulation of macrophage chemotaxis | 6/707 | 0.000409 | 0.00674 | 6 |
| GO:0071624 | positive regulation of granulocyte chemotaxis | 6/707 | 0.000409 | 0.00674 | 6 |
| GO:0071360 | cellular response to exogenous dsRNA | 5/707 | 0.000416 | 0.006828 | 5 |
| GO:0051896 | regulation of protein kinase B signaling | 21/707 | 0.000426 | 0.006974 | 21 |
| GO:0001961 | positive regulation of cytokine-mediated signaling pathway | 8/707 | 0.000433 | 0.007034 | 8 |
| GO:0045661 | regulation of myoblast differentiation | 8/707 | 0.000433 | 0.007034 | 8 |
| GO:0051235 | maintenance of location | 25/707 | 0.000438 | 0.007087 | 25 |
| GO:1903034 | regulation of response to wounding | 16/707 | 0.000455 | 0.007346 | 16 |
| GO:0042401 | cellular biogenic amine biosynthetic process | 7/707 | 0.000462 | 0.007375 | 7 |
| GO:0048246 | macrophage chemotaxis | 7/707 | 0.000462 | 0.007375 | 7 |
| GO:0071276 | cellular response to cadmium ion | 7/707 | 0.000462 | 0.007375 | 7 |
| GO:0006576 | cellular biogenic amine metabolic process | 12/707 | 0.000473 | 0.007529 | 12 |
| GO:0070304 | positive regulation of stress-activated protein kinase signaling cascade | 13/707 | 0.000482 | 0.007651 | 13 |
| GO:0032648 | regulation of interferon-beta production | 8/707 | 0.000499 | 0.007889 | 8 |
| GO:0002507 | tolerance induction | 6/707 | 0.000505 | 0.007954 | 6 |
| GO:0097553 | calcium ion transmembrane import into cytosol | 14/707 | 0.000511 | 0.00801 | 14 |
| GO:0070673 | response to interleukin-18 | 4/707 | 0.000523 | 0.00801 | 4 |
| GO:1990440 | positive regulation of transcription from RNA polymerase II promoter in response to endoplasmic reticulum stress | 4/707 | 0.000523 | 0.00801 | 4 |
| GO:2001269 | positive regulation of cysteine-type endopeptidase activity involved in apoptotic signaling pathway | 4/707 | 0.000523 | 0.00801 | 4 |
| GO:0002688 | regulation of leukocyte chemotaxis | 13/707 | 0.000523 | 0.00801 | 13 |
| GO:0032652 | regulation of interleukin-1 production | 13/707 | 0.000523 | 0.00801 | 13 |
| GO:0050868 | negative regulation of T cell activation | 13/707 | 0.000523 | 0.00801 | 13 |
| GO:0051208 | sequestering of calcium ion | 13/707 | 0.000523 | 0.00801 | 13 |
| GO:0036293 | response to decreased oxygen levels | 27/707 | 0.000523 | 0.00801 | 27 |
| GO:0009309 | amine biosynthetic process | 7/707 | 0.000545 | 0.008313 | 7 |
| GO:0062207 | regulation of pattern recognition receptor signaling pathway | 12/707 | 0.000564 | 0.00858 | 12 |
| GO:0019932 | second-messenger-mediated signaling | 24/707 | 0.000585 | 0.008876 | 24 |
| GO:0031346 | positive regulation of cell projection organization | 26/707 | 0.000587 | 0.008876 | 26 |
| GO:0003015 | heart process | 23/707 | 0.000591 | 0.008905 | 23 |
| GO:1903305 | regulation of regulated secretory pathway | 14/707 | 0.000594 | 0.008921 | 14 |
| GO:0019884 | antigen processing and presentation of exogenous antigen | 17/707 | 0.000613 | 0.009171 | 17 |
| GO:1901222 | regulation of NIK/NF-kappaB signaling | 12/707 | 0.000615 | 0.009171 | 12 |
| GO:0071549 | cellular response to dexamethasone stimulus | 6/707 | 0.000616 | 0.009171 | 6 |
| GO:0032735 | positive regulation of interleukin-12 production | 7/707 | 0.000639 | 0.009483 | 7 |
| GO:0032088 | negative regulation of NF-kappaB transcription factor activity | 11/707 | 0.000646 | 0.00955 | 11 |
| GO:0002820 | negative regulation of adaptive immune response | 8/707 | 0.000655 | 0.009623 | 8 |
| GO:0032608 | interferon-beta production | 8/707 | 0.000655 | 0.009623 | 8 |
| GO:0048639 | positive regulation of developmental growth | 16/707 | 0.000676 | 0.00991 | 16 |
| GO:0001889 | liver development | 14/707 | 0.000687 | 0.010012 | 14 |
| GO:0098754 | detoxification | 14/707 | 0.000687 | 0.010012 | 14 |
| GO:1905039 | carboxylic acid transmembrane transport | 15/707 | 0.00069 | 0.010016 | 15 |
| GO:0001666 | response to hypoxia | 26/707 | 0.000697 | 0.010071 | 26 |
| GO:0050804 | modulation of chemical synaptic transmission | 29/707 | 0.000698 | 0.010071 | 29 |
| GO:0046597 | negative regulation of viral entry into host cell | 5/707 | 0.000707 | 0.01018 | 5 |
| GO:0001776 | leukocyte homeostasis | 10/707 | 0.000717 | 0.010286 | 10 |
| GO:0001659 | temperature homeostasis | 16/707 | 0.000721 | 0.010311 | 16 |
| GO:0099177 | regulation of trans-synaptic signaling | 29/707 | 0.000725 | 0.010346 | 29 |
| GO:0009914 | hormone transport | 24/707 | 0.000735 | 0.010456 | 24 |
| GO:1903825 | organic acid transmembrane transport | 15/707 | 0.000738 | 0.010461 | 15 |
| GO:0002720 | positive regulation of cytokine production involved in immune response | 8/707 | 0.000746 | 0.010516 | 8 |
| GO:0010524 | positive regulation of calcium ion transport into cytosol | 8/707 | 0.000746 | 0.010516 | 8 |
| GO:0022409 | positive regulation of cell-cell adhesion | 22/707 | 0.000754 | 0.010546 | 22 |
| GO:0032494 | response to peptidoglycan | 4/707 | 0.000761 | 0.010546 | 4 |
| GO:0033004 | negative regulation of mast cell activation | 4/707 | 0.000761 | 0.010546 | 4 |
| GO:0035672 | oligopeptide transmembrane transport | 4/707 | 0.000761 | 0.010546 | 4 |
| GO:0036462 | TRAIL-activated apoptotic signaling pathway | 4/707 | 0.000761 | 0.010546 | 4 |
| GO:0048266 | behavioral response to pain | 4/707 | 0.000761 | 0.010546 | 4 |
| GO:0061008 | hepaticobiliary system development | 14/707 | 0.000793 | 0.010953 | 14 |
| GO:0010975 | regulation of neuron projection development | 30/707 | 0.000803 | 0.011054 | 30 |
| GO:0006816 | calcium ion transport | 29/707 | 0.000814 | 0.011182 | 29 |
| GO:0002224 | toll-like receptor signaling pathway | 15/707 | 0.000843 | 0.011538 | 15 |
| GO:0061756 | leukocyte adhesion to vascular endothelial cell | 8/707 | 0.000848 | 0.011572 | 8 |
| GO:0062208 | positive regulation of pattern recognition receptor signaling pathway | 7/707 | 0.000867 | 0.011767 | 7 |
| GO:2001239 | regulation of extrinsic apoptotic signaling pathway in absence of ligand | 7/707 | 0.000867 | 0.011767 | 7 |
| GO:0060047 | heart contraction | 22/707 | 0.00087 | 0.011779 | 22 |
| GO:0034405 | response to fluid shear stress | 6/707 | 0.000896 | 0.012098 | 6 |
| GO:0032612 | interleukin-1 production | 13/707 | 0.000901 | 0.012128 | 13 |
| GO:0022612 | gland morphogenesis | 12/707 | 0.00093 | 0.012423 | 12 |
| GO:0031099 | regeneration | 17/707 | 0.000931 | 0.012423 | 17 |
| GO:0048015 | phosphatidylinositol-mediated signaling | 17/707 | 0.000931 | 0.012423 | 17 |
| GO:0051100 | negative regulation of binding | 15/707 | 0.00096 | 0.012777 | 15 |
| GO:0051962 | positive regulation of nervous system development | 21/707 | 0.000963 | 0.012789 | 21 |
| GO:0043300 | regulation of leukocyte degranulation | 7/707 | 0.001002 | 0.013196 | 7 |
| GO:0046677 | response to antibiotic | 7/707 | 0.001002 | 0.013196 | 7 |
| GO:2000273 | positive regulation of signaling receptor activity | 7/707 | 0.001002 | 0.013196 | 7 |
| GO:0120162 | positive regulation of cold-induced thermogenesis | 11/707 | 0.00101 | 0.013255 | 11 |
| GO:0048660 | regulation of smooth muscle cell proliferation | 15/707 | 0.001023 | 0.0134 | 15 |
| GO:0048002 | antigen processing and presentation of peptide antigen | 17/707 | 0.001044 | 0.0135 | 17 |
| GO:0008217 | regulation of blood pressure | 16/707 | 0.001044 | 0.0135 | 16 |
| GO:0014074 | response to purine-containing compound | 14/707 | 0.001046 | 0.0135 | 14 |
| GO:0106106 | cold-induced thermogenesis | 14/707 | 0.001046 | 0.0135 | 14 |
| GO:0120161 | regulation of cold-induced thermogenesis | 14/707 | 0.001046 | 0.0135 | 14 |
| GO:0097193 | intrinsic apoptotic signaling pathway | 22/707 | 0.001048 | 0.0135 | 22 |
| GO:0006857 | oligopeptide transport | 4/707 | 0.001067 | 0.013582 | 4 |
| GO:0034616 | response to laminar fluid shear stress | 4/707 | 0.001067 | 0.013582 | 4 |
| GO:0039528 | cytoplasmic pattern recognition receptor signaling pathway in response to virus | 6/707 | 0.001069 | 0.013582 | 6 |
| GO:1901099 | negative regulation of signal transduction in absence of ligand | 6/707 | 0.001069 | 0.013582 | 6 |
| GO:2001240 | negative regulation of extrinsic apoptotic signaling pathway in absence of ligand | 6/707 | 0.001069 | 0.013582 | 6 |
| GO:0050870 | positive regulation of T cell activation | 18/707 | 0.001085 | 0.013753 | 18 |
| GO:1901215 | negative regulation of neuron death | 17/707 | 0.001105 | 0.01397 | 17 |
| GO:0002710 | negative regulation of T cell mediated immunity | 5/707 | 0.001129 | 0.014166 | 5 |
| GO:0032069 | regulation of nuclease activity | 5/707 | 0.001129 | 0.014166 | 5 |
| GO:0071359 | cellular response to dsRNA | 5/707 | 0.001129 | 0.014166 | 5 |
| GO:0042269 | regulation of natural killer cell mediated cytotoxicity | 7/707 | 0.001154 | 0.01444 | 7 |
| GO:0048659 | smooth muscle cell proliferation | 15/707 | 0.001161 | 0.014492 | 15 |
| GO:0048017 | inositol lipid-mediated signaling | 17/707 | 0.001169 | 0.014548 | 17 |
| GO:0030099 | myeloid cell differentiation | 29/707 | 0.001184 | 0.014693 | 29 |
| GO:0042102 | positive regulation of T cell proliferation | 11/707 | 0.001196 | 0.014812 | 11 |
| GO:0019722 | calcium-mediated signaling | 17/707 | 0.001306 | 0.01613 | 17 |
| GO:0051781 | positive regulation of cell division | 10/707 | 0.001394 | 0.017144 | 10 |
| GO:0010996 | response to auditory stimulus | 5/707 | 0.001399 | 0.017144 | 5 |
| GO:0036499 | PERK-mediated unfolded protein response | 5/707 | 0.001399 | 0.017144 | 5 |
| GO:0046634 | regulation of alpha-beta T cell activation | 11/707 | 0.00141 | 0.017186 | 11 |
| GO:2000379 | positive regulation of reactive oxygen species metabolic process | 11/707 | 0.00141 | 0.017186 | 11 |
| GO:0050769 | positive regulation of neurogenesis | 18/707 | 0.001413 | 0.017186 | 18 |
| GO:0045927 | positive regulation of growth | 20/707 | 0.001421 | 0.017196 | 20 |
| GO:0070482 | response to oxygen levels | 27/707 | 0.001425 | 0.017196 | 27 |
| GO:0031668 | cellular response to extracellular stimulus | 19/707 | 0.001426 | 0.017196 | 19 |
| GO:0002753 | cytoplasmic pattern recognition receptor signaling pathway | 9/707 | 0.001432 | 0.017196 | 9 |
| GO:0015909 | long-chain fatty acid transport | 9/707 | 0.001432 | 0.017196 | 9 |
| GO:0002475 | antigen processing and presentation via MHC class Ib | 4/707 | 0.00145 | 0.017332 | 4 |
| GO:0002864 | regulation of acute inflammatory response to antigenic stimulus | 4/707 | 0.00145 | 0.017332 | 4 |
| GO:0046631 | alpha-beta T cell activation | 14/707 | 0.001455 | 0.017342 | 14 |
| GO:0009164 | nucleoside catabolic process | 6/707 | 0.001488 | 0.017652 | 6 |
| GO:0033280 | response to vitamin D | 6/707 | 0.001488 | 0.017652 | 6 |
| GO:0045862 | positive regulation of proteolysis | 26/707 | 0.001501 | 0.01776 | 26 |
| GO:0051701 | biological process involved in interaction with host | 18/707 | 0.001566 | 0.018472 | 18 |
| GO:0002040 | sprouting angiogenesis | 16/707 | 0.001569 | 0.018472 | 16 |
| GO:0031100 | animal organ regeneration | 9/707 | 0.00158 | 0.018557 | 9 |
| GO:0072676 | lymphocyte migration | 12/707 | 0.001587 | 0.018598 | 12 |
| GO:0051924 | regulation of calcium ion transport | 19/707 | 0.001651 | 0.019244 | 19 |
| GO:0032651 | regulation of interleukin-1 beta production | 11/707 | 0.001655 | 0.019244 | 11 |
| GO:0070498 | interleukin-1-mediated signaling pathway | 11/707 | 0.001655 | 0.019244 | 11 |
| GO:0042542 | response to hydrogen peroxide | 13/707 | 0.0017 | 0.019474 | 13 |
| GO:0043524 | negative regulation of neuron apoptotic process | 13/707 | 0.0017 | 0.019474 | 13 |
| GO:0051384 | response to glucocorticoid | 13/707 | 0.0017 | 0.019474 | 13 |
| GO:0001101 | response to acid chemical | 12/707 | 0.001707 | 0.019474 | 12 |
| GO:0061098 | positive regulation of protein tyrosine kinase activity | 8/707 | 0.001712 | 0.019474 | 8 |
| GO:0071294 | cellular response to zinc ion | 5/707 | 0.001713 | 0.019474 | 5 |
| GO:0090023 | positive regulation of neutrophil chemotaxis | 5/707 | 0.001713 | 0.019474 | 5 |
| GO:1903901 | negative regulation of viral life cycle | 5/707 | 0.001713 | 0.019474 | 5 |
| GO:0002823 | negative regulation of adaptive immune response based on somatic recombination of immune receptors built from immunoglobulin superfamily domains | 7/707 | 0.001719 | 0.019474 | 7 |
| GO:0009409 | response to cold | 7/707 | 0.001719 | 0.019474 | 7 |
| GO:0045581 | negative regulation of T cell differentiation | 7/707 | 0.001719 | 0.019474 | 7 |
| GO:0006814 | sodium ion transport | 19/707 | 0.001733 | 0.019539 | 19 |
| GO:0045444 | fat cell differentiation | 18/707 | 0.001733 | 0.019539 | 18 |
| GO:0031960 | response to corticosteroid | 14/707 | 0.001758 | 0.019781 | 14 |
| GO:1901653 | cellular response to peptide | 27/707 | 0.001778 | 0.019959 | 27 |
| GO:0044706 | multi-multicellular organism process | 17/707 | 0.001802 | 0.02018 | 17 |
| GO:0044409 | entry into host | 14/707 | 0.001871 | 0.020847 | 14 |
| GO:1990845 | adaptive thermogenesis | 14/707 | 0.001871 | 0.020847 | 14 |
| GO:0071230 | cellular response to amino acid stimulus | 8/707 | 0.001907 | 0.021146 | 8 |
| GO:0090066 | regulation of anatomical structure size | 32/707 | 0.001919 | 0.021146 | 32 |
| GO:0010273 | detoxification of copper ion | 4/707 | 0.00192 | 0.021146 | 4 |
| GO:1990169 | stress response to copper ion | 4/707 | 0.00192 | 0.021146 | 4 |
| GO:2000345 | regulation of hepatocyte proliferation | 4/707 | 0.00192 | 0.021146 | 4 |
| GO:0002715 | regulation of natural killer cell mediated immunity | 7/707 | 0.001949 | 0.021319 | 7 |
| GO:0042149 | cellular response to glucose starvation | 7/707 | 0.001949 | 0.021319 | 7 |
| GO:0045778 | positive regulation of ossification | 7/707 | 0.001949 | 0.021319 | 7 |
| GO:0034101 | erythrocyte homeostasis | 12/707 | 0.001968 | 0.021478 | 12 |
| GO:0014065 | phosphatidylinositol 3-kinase signaling | 14/707 | 0.001989 | 0.021632 | 14 |
| GO:0051302 | regulation of cell division | 15/707 | 0.001991 | 0.021632 | 15 |
| GO:0000302 | response to reactive oxygen species | 18/707 | 0.002011 | 0.021804 | 18 |
| GO:0006213 | pyrimidine nucleoside metabolic process | 6/707 | 0.002023 | 0.021877 | 6 |
| GO:0071692 | protein localization to extracellular region | 26/707 | 0.002027 | 0.021877 | 26 |
| GO:0035902 | response to immobilization stress | 5/707 | 0.002076 | 0.022259 | 5 |
| GO:0060544 | regulation of necroptotic process | 5/707 | 0.002076 | 0.022259 | 5 |
| GO:0043434 | response to peptide hormone | 29/707 | 0.002076 | 0.022259 | 29 |
| GO:0000209 | protein polyubiquitination | 24/707 | 0.002091 | 0.022368 | 24 |
| GO:0030073 | insulin secretion | 17/707 | 0.002105 | 0.022463 | 17 |
| GO:0006521 | regulation of cellular amino acid metabolic process | 8/707 | 0.002119 | 0.022568 | 8 |
| GO:0072593 | reactive oxygen species metabolic process | 21/707 | 0.002183 | 0.0232 | 21 |
| GO:1903409 | reactive oxygen species biosynthetic process | 12/707 | 0.00226 | 0.023963 | 12 |
| GO:0034121 | regulation of toll-like receptor signaling pathway | 9/707 | 0.0023 | 0.024333 | 9 |
| GO:0051651 | maintenance of location in cell | 17/707 | 0.002329 | 0.024583 | 17 |
| GO:0046718 | viral entry into host cell | 13/707 | 0.002353 | 0.024783 | 13 |
| GO:0009267 | cellular response to starvation | 14/707 | 0.002381 | 0.02489 | 14 |
| GO:0030168 | platelet activation | 14/707 | 0.002381 | 0.02489 | 14 |
| GO:0046879 | hormone secretion | 22/707 | 0.002384 | 0.02489 | 22 |
| GO:1901214 | regulation of neuron death | 22/707 | 0.002384 | 0.02489 | 22 |
| GO:0002708 | positive regulation of lymphocyte mediated immunity | 11/707 | 0.00242 | 0.025152 | 11 |
| GO:0032611 | interleukin-1 beta production | 11/707 | 0.00242 | 0.025152 | 11 |
| GO:0007409 | axonogenesis | 30/707 | 0.002441 | 0.025316 | 30 |
| GO:0031669 | cellular response to nutrient levels | 17/707 | 0.002448 | 0.025341 | 17 |
| GO:0002931 | response to ischemia | 7/707 | 0.002478 | 0.025572 | 7 |
| GO:0045779 | negative regulation of bone resorption | 4/707 | 0.002484 | 0.025572 | 4 |
| GO:0002719 | negative regulation of cytokine production involved in immune response | 5/707 | 0.002492 | 0.025572 | 5 |
| GO:0071880 | adenylate cyclase-activating adrenergic receptor signaling pathway | 5/707 | 0.002492 | 0.025572 | 5 |
| GO:0010959 | regulation of metal ion transport | 19/707 | 0.002517 | 0.025631 | 19 |
| GO:0030072 | peptide hormone secretion | 19/707 | 0.002517 | 0.025631 | 19 |
| GO:0007631 | feeding behavior | 10/707 | 0.002519 | 0.025631 | 10 |
| GO:0051591 | response to cAMP | 10/707 | 0.002519 | 0.025631 | 10 |
| GO:0007596 | blood coagulation | 24/707 | 0.002533 | 0.025658 | 24 |
| GO:0070997 | neuron death | 24/707 | 0.002533 | 0.025658 | 24 |
| GO:0002705 | positive regulation of leukocyte mediated immunity | 12/707 | 0.002588 | 0.02616 | 12 |
| GO:0032732 | positive regulation of interleukin-1 production | 8/707 | 0.002599 | 0.026218 | 8 |
| GO:0051960 | regulation of nervous system development | 28/707 | 0.002629 | 0.026463 | 28 |
| GO:0098657 | import into cell | 18/707 | 0.002681 | 0.026753 | 18 |
| GO:0022604 | regulation of cell morphogenesis | 22/707 | 0.002691 | 0.026753 | 22 |
| GO:0001914 | regulation of T cell mediated cytotoxicity | 6/707 | 0.002691 | 0.026753 | 6 |
| GO:0007618 | mating | 6/707 | 0.002691 | 0.026753 | 6 |
| GO:0032733 | positive regulation of interleukin-10 production | 6/707 | 0.002691 | 0.026753 | 6 |
| GO:0045429 | positive regulation of nitric oxide biosynthetic process | 6/707 | 0.002691 | 0.026753 | 6 |
| GO:0007623 | circadian rhythm | 17/707 | 0.002703 | 0.026814 | 17 |
| GO:0001910 | regulation of leukocyte mediated cytotoxicity | 9/707 | 0.002747 | 0.027199 | 9 |
| GO:0045471 | response to ethanol | 12/707 | 0.002765 | 0.027258 | 12 |
| GO:0043433 | negative regulation of DNA-binding transcription factor activity | 15/707 | 0.002781 | 0.027258 | 15 |
| GO:0071715 | icosanoid transport | 7/707 | 0.002782 | 0.027258 | 7 |
| GO:0097300 | programmed necrotic cell death | 7/707 | 0.002782 | 0.027258 | 7 |
| GO:1903307 | positive regulation of regulated secretory pathway | 7/707 | 0.002782 | 0.027258 | 7 |
| GO:0090501 | RNA phosphodiester bond hydrolysis | 14/707 | 0.002834 | 0.027717 | 14 |
| GO:0007599 | hemostasis | 24/707 | 0.002941 | 0.028699 | 24 |
| GO:0010522 | regulation of calcium ion transport into cytosol | 10/707 | 0.002948 | 0.028713 | 10 |
| GO:0002675 | positive regulation of acute inflammatory response | 5/707 | 0.002965 | 0.028766 | 5 |
| GO:0140467 | integrated stress response signaling | 5/707 | 0.002965 | 0.028766 | 5 |
| GO:0045445 | myoblast differentiation | 9/707 | 0.002995 | 0.028993 | 9 |
| GO:1905954 | positive regulation of lipid localization | 11/707 | 0.003002 | 0.029 | 11 |
| GO:0050817 | coagulation | 24/707 | 0.003051 | 0.029415 | 24 |
| GO:1904407 | positive regulation of nitric oxide metabolic process | 6/707 | 0.003081 | 0.029649 | 6 |
| GO:0009306 | protein secretion | 25/707 | 0.003088 | 0.029658 | 25 |
| GO:0043392 | negative regulation of DNA binding | 7/707 | 0.003112 | 0.029829 | 7 |
| GO:0017157 | regulation of exocytosis | 16/707 | 0.003133 | 0.029919 | 16 |
| GO:0006577 | amino-acid betaine metabolic process | 4/707 | 0.003153 | 0.029919 | 4 |
| GO:0015732 | prostaglandin transport | 4/707 | 0.003153 | 0.029919 | 4 |
| GO:0061158 | 3'-UTR-mediated mRNA destabilization | 4/707 | 0.003153 | 0.029919 | 4 |
| GO:2001267 | regulation of cysteine-type endopeptidase activity involved in apoptotic signaling pathway | 4/707 | 0.003153 | 0.029919 | 4 |
| GO:0046635 | positive regulation of alpha-beta T cell activation | 8/707 | 0.003159 | 0.029919 | 8 |
| GO:0048661 | positive regulation of smooth muscle cell proliferation | 10/707 | 0.003183 | 0.030086 | 10 |
| GO:0035592 | establishment of protein localization to extracellular region | 25/707 | 0.0032 | 0.030183 | 25 |
| GO:1901136 | carbohydrate derivative catabolic process | 16/707 | 0.003294 | 0.03101 | 16 |
| GO:0048545 | response to steroid hormone | 23/707 | 0.003351 | 0.031489 | 23 |
| GO:0046328 | regulation of JNK cascade | 12/707 | 0.003358 | 0.031489 | 12 |
| GO:0032872 | regulation of stress-activated MAPK cascade | 15/707 | 0.003439 | 0.032186 | 15 |
| GO:0038034 | signal transduction in absence of ligand | 8/707 | 0.003472 | 0.032366 | 8 |
| GO:0097192 | extrinsic apoptotic signaling pathway in absence of ligand | 8/707 | 0.003472 | 0.032366 | 8 |
| GO:0010575 | positive regulation of vascular endothelial growth factor production | 5/707 | 0.0035 | 0.032366 | 5 |
| GO:0062098 | regulation of programmed necrotic cell death | 5/707 | 0.0035 | 0.032366 | 5 |
| GO:1903672 | positive regulation of sprouting angiogenesis | 5/707 | 0.0035 | 0.032366 | 5 |
| GO:0045776 | negative regulation of blood pressure | 6/707 | 0.003512 | 0.032366 | 6 |
| GO:0072529 | pyrimidine-containing compound catabolic process | 6/707 | 0.003512 | 0.032366 | 6 |
| GO:1902895 | positive regulation of pri-miRNA transcription by RNA polymerase II | 6/707 | 0.003512 | 0.032366 | 6 |
| GO:0006970 | response to osmotic stress | 9/707 | 0.003543 | 0.032589 | 9 |
| GO:1903320 | regulation of protein modification by small protein conjugation or removal | 18/707 | 0.003689 | 0.033866 | 18 |
| GO:0007584 | response to nutrient | 14/707 | 0.003746 | 0.034325 | 14 |
| GO:0042110 | T cell activation | 30/707 | 0.003779 | 0.034559 | 30 |
| GO:0061041 | regulation of wound healing | 12/707 | 0.003807 | 0.03475 | 12 |
| GO:0097479 | synaptic vesicle localization | 7/707 | 0.003863 | 0.035196 | 7 |
| GO:0002281 | macrophage activation involved in immune response | 4/707 | 0.003935 | 0.035512 | 4 |
| GO:0035743 | CD4-positive alpha-beta T cell cytokine production | 4/707 | 0.003935 | 0.035512 | 4 |
| GO:0046851 | negative regulation of bone remodeling | 4/707 | 0.003935 | 0.035512 | 4 |
| GO:0060546 | negative regulation of necroptotic process | 4/707 | 0.003935 | 0.035512 | 4 |
| GO:0071498 | cellular response to fluid shear stress | 4/707 | 0.003935 | 0.035512 | 4 |
| GO:0030218 | erythrocyte differentiation | 11/707 | 0.003947 | 0.035556 | 11 |
| GO:0019233 | sensory perception of pain | 10/707 | 0.003979 | 0.035705 | 10 |
| GO:1903426 | regulation of reactive oxygen species biosynthetic process | 10/707 | 0.003979 | 0.035705 | 10 |
| GO:0032689 | negative regulation of interferon-gamma production | 6/707 | 0.003986 | 0.035705 | 6 |
| GO:0070302 | regulation of stress-activated protein kinase signaling cascade | 15/707 | 0.004012 | 0.035871 | 15 |
| GO:0034976 | response to endoplasmic reticulum stress | 21/707 | 0.004024 | 0.03591 | 21 |
| GO:0001916 | positive regulation of T cell mediated cytotoxicity | 5/707 | 0.004101 | 0.036328 | 5 |
| GO:0043304 | regulation of mast cell degranulation | 5/707 | 0.004101 | 0.036328 | 5 |
| GO:0060055 | angiogenesis involved in wound healing | 5/707 | 0.004101 | 0.036328 | 5 |
| GO:1902624 | positive regulation of neutrophil migration | 5/707 | 0.004101 | 0.036328 | 5 |
| GO:0046330 | positive regulation of JNK cascade | 9/707 | 0.004167 | 0.036845 | 9 |
| GO:0015908 | fatty acid transport | 13/707 | 0.004278 | 0.037623 | 13 |
| GO:0045620 | negative regulation of lymphocyte differentiation | 7/707 | 0.004287 | 0.037623 | 7 |
| GO:0071385 | cellular response to glucocorticoid stimulus | 7/707 | 0.004287 | 0.037623 | 7 |
| GO:1903428 | positive regulation of reactive oxygen species biosynthetic process | 7/707 | 0.004287 | 0.037623 | 7 |
| GO:0033002 | muscle cell proliferation | 17/707 | 0.004326 | 0.037899 | 17 |
| GO:0051098 | regulation of binding | 24/707 | 0.004359 | 0.038114 | 24 |
| GO:0050852 | T cell receptor signaling pathway | 16/707 | 0.004406 | 0.038462 | 16 |
| GO:0048638 | regulation of developmental growth | 22/707 | 0.004598 | 0.040064 | 22 |
| GO:2001234 | negative regulation of apoptotic signaling pathway | 17/707 | 0.004732 | 0.041037 | 17 |
| GO:0032731 | positive regulation of interleukin-1 beta production | 7/707 | 0.004744 | 0.041037 | 7 |
| GO:0050879 | multicellular organismal movement | 7/707 | 0.004744 | 0.041037 | 7 |
| GO:0050881 | musculoskeletal movement | 7/707 | 0.004744 | 0.041037 | 7 |
| GO:0007176 | regulation of epidermal growth factor-activated receptor activity | 5/707 | 0.004772 | 0.041037 | 5 |
| GO:0033006 | regulation of mast cell activation involved in immune response | 5/707 | 0.004772 | 0.041037 | 5 |
| GO:0070633 | transepithelial transport | 5/707 | 0.004772 | 0.041037 | 5 |
| GO:0046916 | cellular transition metal ion homeostasis | 11/707 | 0.004802 | 0.041037 | 11 |
| GO:0051928 | positive regulation of calcium ion transport | 11/707 | 0.004802 | 0.041037 | 11 |
| GO:0002544 | chronic inflammatory response | 4/707 | 0.004838 | 0.041037 | 4 |
| GO:0007620 | copulation | 4/707 | 0.004838 | 0.041037 | 4 |
| GO:0034138 | toll-like receptor 3 signaling pathway | 4/707 | 0.004838 | 0.041037 | 4 |
| GO:0045780 | positive regulation of bone resorption | 4/707 | 0.004838 | 0.041037 | 4 |
| GO:0062099 | negative regulation of programmed necrotic cell death | 4/707 | 0.004838 | 0.041037 | 4 |
| GO:2001185 | regulation of CD8-positive alpha-beta T cell activation | 4/707 | 0.004838 | 0.041037 | 4 |
| GO:0055072 | iron ion homeostasis | 9/707 | 0.004874 | 0.041266 | 9 |
| GO:0042116 | macrophage activation | 10/707 | 0.004925 | 0.041623 | 10 |
| GO:0010976 | positive regulation of neuron projection development | 13/707 | 0.005054 | 0.042586 | 13 |
| GO:0034142 | toll-like receptor 4 signaling pathway | 6/707 | 0.005074 | 0.042586 | 6 |
| GO:0046189 | phenol-containing compound biosynthetic process | 6/707 | 0.005074 | 0.042586 | 6 |
| GO:0070266 | necroptotic process | 6/707 | 0.005074 | 0.042586 | 6 |
| GO:0071248 | cellular response to metal ion | 15/707 | 0.00514 | 0.043045 | 15 |
| GO:0002524 | hypersensitivity | 3/707 | 0.005165 | 0.043045 | 3 |
| GO:0002725 | negative regulation of T cell cytokine production | 3/707 | 0.005165 | 0.043045 | 3 |
| GO:0035907 | dorsal aorta development | 3/707 | 0.005165 | 0.043045 | 3 |
| GO:0032757 | positive regulation of interleukin-8 production | 7/707 | 0.005238 | 0.043576 | 7 |
| GO:0006919 | activation of cysteine-type endopeptidase activity involved in apoptotic process | 9/707 | 0.005261 | 0.04369 | 9 |
| GO:1902107 | positive regulation of leukocyte differentiation | 13/707 | 0.005337 | 0.04417 | 13 |
| GO:1903708 | positive regulation of hemopoiesis | 13/707 | 0.005337 | 0.04417 | 13 |
| GO:0030001 | metal ion transport | 30/707 | 0.005378 | 0.044436 | 30 |
| GO:1903036 | positive regulation of response to wounding | 8/707 | 0.005415 | 0.044663 | 8 |
| GO:0060249 | anatomical structure homeostasis | 29/707 | 0.005502 | 0.045298 | 29 |
| GO:0071875 | adrenergic receptor signaling pathway | 5/707 | 0.005518 | 0.045353 | 5 |
| GO:0070664 | negative regulation of leukocyte proliferation | 9/707 | 0.005671 | 0.04653 | 9 |
| GO:0015804 | neutral amino acid transport | 6/707 | 0.005693 | 0.046558 | 6 |
| GO:1901658 | glycosyl compound catabolic process | 6/707 | 0.005693 | 0.046558 | 6 |
| GO:0002726 | positive regulation of T cell cytokine production | 4/707 | 0.005871 | 0.047734 | 4 |
| GO:0010042 | response to manganese ion | 4/707 | 0.005871 | 0.047734 | 4 |
| GO:0034104 | negative regulation of tissue remodeling | 4/707 | 0.005871 | 0.047734 | 4 |
| GO:0006809 | nitric oxide biosynthetic process | 8/707 | 0.005887 | 0.047734 | 8 |
| GO:0009166 | nucleotide catabolic process | 8/707 | 0.005887 | 0.047734 | 8 |
| GO:1903707 | negative regulation of hemopoiesis | 10/707 | 0.006039 | 0.04888 | 10 |
| GO:0050673 | epithelial cell proliferation | 27/707 | 0.006072 | 0.049066 | 27 |
| GO:0033273 | response to vitamin | 9/707 | 0.006105 | 0.049166 | 9 |
| GO:0042058 | regulation of epidermal growth factor receptor signaling pathway | 9/707 | 0.006105 | 0.049166 | 9 |
| GO:0007160 | cell-matrix adhesion | 17/707 | 0.006138 | 0.049353 | 17 |

**Supplementary Table 13. The GO-BP enrichment of down-regulated DEGs in HK-2 cells of 24h**

| ID | Description | GeneRatio | pvalue | p.adjust | Count |
| --- | --- | --- | --- | --- | --- |
| GO:0016126 | sterol biosynthetic process | 18/500 | 1.51E-12 | 5.62E-09 | 18 |
| GO:0006695 | cholesterol biosynthetic process | 17/500 | 4.25E-12 | 5.62E-09 | 17 |
| GO:1902653 | secondary alcohol biosynthetic process | 17/500 | 4.25E-12 | 5.62E-09 | 17 |
| GO:1902652 | secondary alcohol metabolic process | 22/500 | 2.08E-10 | 1.49E-07 | 22 |
| GO:0045540 | regulation of cholesterol biosynthetic process | 13/500 | 2.26E-10 | 1.49E-07 | 13 |
| GO:0106118 | regulation of sterol biosynthetic process | 13/500 | 2.26E-10 | 1.49E-07 | 13 |
| GO:0008203 | cholesterol metabolic process | 21/500 | 4.29E-10 | 2.42E-07 | 21 |
| GO:0016125 | sterol metabolic process | 22/500 | 4.88E-10 | 2.42E-07 | 22 |
| GO:1902930 | regulation of alcohol biosynthetic process | 15/500 | 9.63E-10 | 4.24E-07 | 15 |
| GO:0050804 | modulation of chemical synaptic transmission | 34/500 | 3.41E-09 | 1.31E-06 | 34 |
| GO:0099177 | regulation of trans-synaptic signaling | 34/500 | 3.63E-09 | 1.31E-06 | 34 |
| GO:0090181 | regulation of cholesterol metabolic process | 13/500 | 5.62E-09 | 1.80E-06 | 13 |
| GO:0006066 | alcohol metabolic process | 32/500 | 5.91E-09 | 1.80E-06 | 32 |
| GO:0006694 | steroid biosynthetic process | 22/500 | 7.19E-09 | 2.03E-06 | 22 |
| GO:0050810 | regulation of steroid biosynthetic process | 15/500 | 1.57E-08 | 4.15E-06 | 15 |
| GO:0046165 | alcohol biosynthetic process | 19/500 | 3.37E-08 | 8.34E-06 | 19 |
| GO:0006790 | sulfur compound metabolic process | 30/500 | 1.01E-07 | 2.36E-05 | 30 |
| GO:0019218 | regulation of steroid metabolic process | 16/500 | 1.45E-07 | 3.20E-05 | 16 |
| GO:1901617 | organic hydroxy compound biosynthetic process | 23/500 | 2.61E-07 | 5.44E-05 | 23 |
| GO:0006023 | aminoglycan biosynthetic process | 15/500 | 3.41E-07 | 6.76E-05 | 15 |
| GO:0008202 | steroid metabolic process | 26/500 | 7.88E-07 | 0.000149 | 26 |
| GO:0044272 | sulfur compound biosynthetic process | 19/500 | 9.75E-07 | 0.000176 | 19 |
| GO:1902414 | protein localization to cell junction | 14/500 | 1.13E-06 | 0.000195 | 14 |
| GO:0050806 | positive regulation of synaptic transmission | 16/500 | 1.42E-06 | 0.000235 | 16 |
| GO:0006024 | glycosaminoglycan biosynthetic process | 13/500 | 5.11E-06 | 0.00081 | 13 |
| GO:0001822 | kidney development | 22/500 | 6.09E-06 | 0.000928 | 22 |
| GO:0008299 | isoprenoid biosynthetic process | 7/500 | 6.44E-06 | 0.000945 | 7 |
| GO:0030198 | extracellular matrix organization | 27/500 | 6.91E-06 | 0.000977 | 27 |
| GO:0043062 | extracellular structure organization | 27/500 | 7.24E-06 | 0.000989 | 27 |
| GO:0045229 | external encapsulating structure organization | 27/500 | 7.94E-06 | 0.001048 | 27 |
| GO:0001778 | plasma membrane repair | 5/500 | 8.71E-06 | 0.001113 | 5 |
| GO:0003018 | vascular process in circulatory system | 20/500 | 9.13E-06 | 0.001131 | 20 |
| GO:0072001 | renal system development | 22/500 | 9.53E-06 | 0.001144 | 22 |
| GO:0007156 | homophilic cell adhesion via plasma membrane adhesion molecules | 16/500 | 9.85E-06 | 0.001148 | 16 |
| GO:0001935 | endothelial cell proliferation | 17/500 | 1.39E-05 | 0.001532 | 17 |
| GO:0031032 | actomyosin structure organization | 17/500 | 1.39E-05 | 0.001532 | 17 |
| GO:0006022 | aminoglycan metabolic process | 16/500 | 1.43E-05 | 0.001532 | 16 |
| GO:0001655 | urogenital system development | 23/500 | 1.61E-05 | 0.001682 | 23 |
| GO:0001936 | regulation of endothelial cell proliferation | 16/500 | 2.05E-05 | 0.00208 | 16 |
| GO:0001938 | positive regulation of endothelial cell proliferation | 12/500 | 2.93E-05 | 0.002902 | 12 |
| GO:0003094 | glomerular filtration | 6/500 | 3.02E-05 | 0.002916 | 6 |
| GO:0032835 | glomerulus development | 9/500 | 3.50E-05 | 0.003302 | 9 |
| GO:0097205 | renal filtration | 6/500 | 3.88E-05 | 0.003577 | 6 |
| GO:0048846 | axon extension involved in axon guidance | 7/500 | 4.55E-05 | 0.00401 | 7 |
| GO:1902284 | neuron projection extension involved in neuron projection guidance | 7/500 | 4.55E-05 | 0.00401 | 7 |
| GO:0035418 | protein localization to synapse | 10/500 | 4.89E-05 | 0.004213 | 10 |
| GO:0006865 | amino acid transport | 14/500 | 5.43E-05 | 0.004431 | 14 |
| GO:0140353 | lipid export from cell | 10/500 | 5.45E-05 | 0.004431 | 10 |
| GO:1903510 | mucopolysaccharide metabolic process | 12/500 | 5.48E-05 | 0.004431 | 12 |
| GO:0007158 | neuron cell-cell adhesion | 5/500 | 6.10E-05 | 0.004831 | 5 |
| GO:1901888 | regulation of cell junction assembly | 16/500 | 6.66E-05 | 0.005176 | 16 |
| GO:0003014 | renal system process | 12/500 | 7.06E-05 | 0.005193 | 12 |
| GO:0006936 | muscle contraction | 23/500 | 7.12E-05 | 0.005193 | 23 |
| GO:0097120 | receptor localization to synapse | 8/500 | 7.16E-05 | 0.005193 | 8 |
| GO:0048167 | regulation of synaptic plasticity | 15/500 | 7.28E-05 | 0.005193 | 15 |
| GO:0034329 | cell junction assembly | 26/500 | 7.34E-05 | 0.005193 | 26 |
| GO:0040013 | negative regulation of locomotion | 24/500 | 7.49E-05 | 0.005206 | 24 |
| GO:0050673 | epithelial cell proliferation | 26/500 | 8.23E-05 | 0.00556 | 26 |
| GO:0030203 | glycosaminoglycan metabolic process | 14/500 | 8.28E-05 | 0.00556 | 14 |
| GO:0001755 | neural crest cell migration | 8/500 | 9.39E-05 | 0.006201 | 8 |
| GO:0007015 | actin filament organization | 26/500 | 0.000107 | 0.006849 | 26 |
| GO:0046890 | regulation of lipid biosynthetic process | 16/500 | 0.000107 | 0.006849 | 16 |
| GO:0001501 | skeletal system development | 28/500 | 0.00011 | 0.006874 | 28 |
| GO:0010232 | vascular transport | 10/500 | 0.000111 | 0.006874 | 10 |
| GO:0050808 | synapse organization | 25/500 | 0.000114 | 0.00697 | 25 |
| GO:0042220 | response to cocaine | 7/500 | 0.000124 | 0.00746 | 7 |
| GO:0098742 | cell-cell adhesion via plasma-membrane adhesion molecules | 19/500 | 0.000133 | 0.007845 | 19 |
| GO:0032963 | collagen metabolic process | 11/500 | 0.00015 | 0.008763 | 11 |
| GO:0007411 | axon guidance | 19/500 | 0.00016 | 0.009198 | 19 |
| GO:0097485 | neuron projection guidance | 19/500 | 0.000168 | 0.009498 | 19 |
| GO:0023061 | signal release | 27/500 | 0.000181 | 0.009967 | 27 |
| GO:0099633 | protein localization to postsynaptic specialization membrane | 5/500 | 0.000184 | 0.009967 | 5 |
| GO:0099645 | neurotransmitter receptor localization to postsynaptic specialization membrane | 5/500 | 0.000184 | 0.009967 | 5 |
| GO:0051271 | negative regulation of cellular component movement | 22/500 | 0.000192 | 0.010281 | 22 |
| GO:0035633 | maintenance of blood-brain barrier | 6/500 | 0.000203 | 0.010492 | 6 |
| GO:0032429 | regulation of phospholipase A2 activity | 4/500 | 0.000204 | 0.010492 | 4 |
| GO:0003012 | muscle system process | 26/500 | 0.000204 | 0.010492 | 26 |
| GO:0007269 | neurotransmitter secretion | 13/500 | 0.000254 | 0.012762 | 13 |
| GO:0099643 | signal release from synapse | 13/500 | 0.000254 | 0.012762 | 13 |
| GO:0099504 | synaptic vesicle cycle | 14/500 | 0.000275 | 0.01359 | 14 |
| GO:0061448 | connective tissue development | 17/500 | 0.000278 | 0.01359 | 17 |
| GO:0042692 | muscle cell differentiation | 22/500 | 0.000284 | 0.01371 | 22 |
| GO:0048172 | regulation of short-term neuronal synaptic plasticity | 4/500 | 0.000288 | 0.01371 | 4 |
| GO:0003071 | renal system process involved in regulation of systemic arterial blood pressure | 5/500 | 0.000291 | 0.01371 | 5 |
| GO:0072006 | nephron development | 12/500 | 0.000313 | 0.014578 | 12 |
| GO:0051146 | striated muscle cell differentiation | 18/500 | 0.00032 | 0.014729 | 18 |
| GO:0048660 | regulation of smooth muscle cell proliferation | 13/500 | 0.000347 | 0.015543 | 13 |
| GO:0050679 | positive regulation of epithelial cell proliferation | 15/500 | 0.000351 | 0.015543 | 15 |
| GO:0033865 | nucleoside bisphosphate metabolic process | 12/500 | 0.000357 | 0.015543 | 12 |
| GO:0033875 | ribonucleoside bisphosphate metabolic process | 12/500 | 0.000357 | 0.015543 | 12 |
| GO:0034032 | purine nucleoside bisphosphate metabolic process | 12/500 | 0.000357 | 0.015543 | 12 |
| GO:0032964 | collagen biosynthetic process | 7/500 | 0.000372 | 0.01602 | 7 |
| GO:0048659 | smooth muscle cell proliferation | 13/500 | 0.000391 | 0.01666 | 13 |
| GO:1903729 | regulation of plasma membrane organization | 4/500 | 0.000395 | 0.01666 | 4 |
| GO:0046928 | regulation of neurotransmitter secretion | 9/500 | 0.000415 | 0.017309 | 9 |
| GO:0030206 | chondroitin sulfate biosynthetic process | 5/500 | 0.000439 | 0.017753 | 5 |
| GO:0072012 | glomerulus vasculature development | 5/500 | 0.000439 | 0.017753 | 5 |
| GO:0016079 | synaptic vesicle exocytosis | 10/500 | 0.000441 | 0.017753 | 10 |
| GO:0050678 | regulation of epithelial cell proliferation | 22/500 | 0.000444 | 0.017753 | 22 |
| GO:0030204 | chondroitin sulfate metabolic process | 6/500 | 0.000452 | 0.01791 | 6 |
| GO:0048762 | mesenchymal cell differentiation | 16/500 | 0.000465 | 0.018224 | 16 |
| GO:0006836 | neurotransmitter transport | 15/500 | 0.000478 | 0.018567 | 15 |
| GO:0150104 | transport across blood-brain barrier | 9/500 | 0.000493 | 0.018983 | 9 |
| GO:1903539 | protein localization to postsynaptic membrane | 6/500 | 0.000522 | 0.019523 | 6 |
| GO:0001505 | regulation of neurotransmitter levels | 15/500 | 0.000528 | 0.019523 | 15 |
| GO:0061437 | renal system vasculature development | 5/500 | 0.000532 | 0.019523 | 5 |
| GO:0061440 | kidney vasculature development | 5/500 | 0.000532 | 0.019523 | 5 |
| GO:2000311 | regulation of AMPA receptor activity | 5/500 | 0.000532 | 0.019523 | 5 |
| GO:0014047 | glutamate secretion | 6/500 | 0.000601 | 0.021649 | 6 |
| GO:0007409 | axonogenesis | 25/500 | 0.000601 | 0.021649 | 25 |
| GO:0003073 | regulation of systemic arterial blood pressure | 9/500 | 0.000634 | 0.022218 | 9 |
| GO:0014033 | neural crest cell differentiation | 9/500 | 0.000634 | 0.022218 | 9 |
| GO:0035249 | synaptic transmission glutamatergic | 9/500 | 0.000634 | 0.022218 | 9 |
| GO:0001823 | mesonephros development | 9/500 | 0.000687 | 0.023422 | 9 |
| GO:0060485 | mesenchyme development | 18/500 | 0.000689 | 0.023422 | 18 |
| GO:0072109 | glomerular mesangium development | 4/500 | 0.000689 | 0.023422 | 4 |
| GO:0099003 | vesicle-mediated transport in synapse | 14/500 | 0.000692 | 0.023422 | 14 |
| GO:0010810 | regulation of cell-substrate adhesion | 15/500 | 0.000741 | 0.024869 | 15 |
| GO:0032970 | regulation of actin filament-based process | 22/500 | 0.000749 | 0.02495 | 22 |
| GO:0018146 | keratan sulfate biosynthetic process | 5/500 | 0.000761 | 0.025131 | 5 |
| GO:0016049 | cell growth | 25/500 | 0.000818 | 0.02677 | 25 |
| GO:0045926 | negative regulation of growth | 16/500 | 0.000877 | 0.028418 | 16 |
| GO:0050961 | detection of temperature stimulus involved in sensory perception | 4/500 | 0.000882 | 0.028418 | 4 |
| GO:0050654 | chondroitin sulfate proteoglycan metabolic process | 6/500 | 0.000893 | 0.028525 | 6 |
| GO:0030239 | myofibril assembly | 7/500 | 0.000914 | 0.028964 | 7 |
| GO:2000146 | negative regulation of cell motility | 20/500 | 0.000951 | 0.029904 | 20 |
| GO:0062237 | protein localization to postsynapse | 6/500 | 0.001011 | 0.03106 | 6 |
| GO:0098815 | modulation of excitatory postsynaptic potential | 6/500 | 0.001011 | 0.03106 | 6 |
| GO:0099072 | regulation of postsynaptic membrane neurotransmitter receptor levels | 7/500 | 0.001011 | 0.03106 | 7 |
| GO:0050650 | chondroitin sulfate proteoglycan biosynthetic process | 5/500 | 0.001057 | 0.031957 | 5 |
| GO:0051968 | positive regulation of synaptic transmission glutamatergic | 5/500 | 0.001057 | 0.031957 | 5 |
| GO:0070527 | platelet aggregation | 7/500 | 0.001231 | 0.036939 | 7 |
| GO:0014032 | neural crest cell development | 8/500 | 0.001256 | 0.036982 | 8 |
| GO:0043279 | response to alkaloid | 9/500 | 0.00126 | 0.036982 | 9 |
| GO:0051588 | regulation of neurotransmitter transport | 9/500 | 0.00126 | 0.036982 | 9 |
| GO:0098657 | import into cell | 15/500 | 0.001274 | 0.037101 | 15 |
| GO:0050919 | negative chemotaxis | 6/500 | 0.001283 | 0.037101 | 6 |
| GO:0001558 | regulation of cell growth | 22/500 | 0.001303 | 0.037403 | 22 |
| GO:0070588 | calcium ion transmembrane transport | 18/500 | 0.001327 | 0.037814 | 18 |
| GO:0030308 | negative regulation of cell growth | 13/500 | 0.001353 | 0.038018 | 13 |
| GO:0006939 | smooth muscle contraction | 9/500 | 0.001353 | 0.038018 | 9 |
| GO:0030336 | negative regulation of cell migration | 19/500 | 0.001367 | 0.03814 | 19 |
| GO:0048841 | regulation of axon extension involved in axon guidance | 5/500 | 0.00143 | 0.039572 | 5 |
| GO:0010712 | regulation of collagen metabolic process | 6/500 | 0.001438 | 0.039572 | 6 |
| GO:0051893 | regulation of focal adhesion assembly | 7/500 | 0.001486 | 0.040316 | 7 |
| GO:0090109 | regulation of cell-substrate junction assembly | 7/500 | 0.001486 | 0.040316 | 7 |
| GO:0006720 | isoprenoid metabolic process | 11/500 | 0.001516 | 0.040864 | 11 |
| GO:0048864 | stem cell development | 8/500 | 0.001595 | 0.042705 | 8 |
| GO:0042339 | keratan sulfate metabolic process | 5/500 | 0.001649 | 0.043837 | 5 |
| GO:0055002 | striated muscle cell development | 9/500 | 0.001665 | 0.043982 | 9 |
| GO:0097503 | sialylation | 4/500 | 0.001686 | 0.044247 | 4 |
| GO:0014031 | mesenchymal cell development | 8/500 | 0.001723 | 0.044916 | 8 |
| GO:0050803 | regulation of synapse structure or activity | 14/500 | 0.001774 | 0.045926 | 14 |
| GO:0034109 | homotypic cell-cell adhesion | 8/500 | 0.001859 | 0.047521 | 8 |
| GO:0048041 | focal adhesion assembly | 8/500 | 0.001859 | 0.047521 | 8 |
| GO:0006637 | acyl-CoA metabolic process | 9/500 | 0.001904 | 0.04782 | 9 |
| GO:0035383 | thioester metabolic process | 9/500 | 0.001904 | 0.04782 | 9 |
| GO:0003093 | regulation of glomerular filtration | 3/500 | 0.001933 | 0.04782 | 3 |
| GO:0072124 | regulation of glomerular mesangial cell proliferation | 3/500 | 0.001933 | 0.04782 | 3 |
| GO:0001667 | ameboidal-type cell migration | 24/500 | 0.001938 | 0.04782 | 24 |
| GO:0015800 | acidic amino acid transport | 7/500 | 0.001943 | 0.04782 | 7 |
| GO:0016048 | detection of temperature stimulus | 4/500 | 0.00204 | 0.049278 | 4 |
| GO:0071636 | positive regulation of transforming growth factor beta production | 4/500 | 0.00204 | 0.049278 | 4 |
| GO:0150105 | protein localization to cell-cell junction | 4/500 | 0.00204 | 0.049278 | 4 |
| GO:0009100 | glycoprotein metabolic process | 22/500 | 0.002052 | 0.049278 | 22 |

**Supplementary Table 14. The pathway enrichment of DEGs in JEG-3 cells of 24h**

| ID | Description | GeneRatio | pvalue | p.adjust | Count |
| --- | --- | --- | --- | --- | --- |
| hsa05164 | Influenza A | 9/78 | 3.51E-05 | 0.005431 | 9 |
| hsa05162 | Measles | 8/78 | 5.22E-05 | 0.005431 | 8 |
| hsa05133 | Pertussis | 6/78 | 8.38E-05 | 0.00581 | 6 |
| hsa05171 | Coronavirus disease - COVID-19 | 9/78 | 0.000361 | 0.012583 | 9 |
| hsa04621 | NOD-like receptor signaling pathway | 8/78 | 0.000366 | 0.012583 | 8 |
| hsa04933 | AGE-RAGE signaling pathway in diabetic complications | 6/78 | 0.000381 | 0.012583 | 6 |
| hsa05142 | Chagas disease | 6/78 | 0.000423 | 0.012583 | 6 |
| hsa04622 | RIG-I-like receptor signaling pathway | 5/78 | 0.000539 | 0.014015 | 5 |
| hsa05169 | Epstein-Barr virus infection | 8/78 | 0.000682 | 0.015755 | 8 |
| hsa04912 | GnRH signaling pathway | 5/78 | 0.001949 | 0.036861 | 5 |
| hsa05323 | Rheumatoid arthritis | 5/78 | 0.001949 | 0.036861 | 5 |

**Supplementary Table 15. The pathway enrichment of DEGs in U-251 MG cells of 24h**

| ID | Description | GeneRatio | pvalue | p.adjust | Count |
| --- | --- | --- | --- | --- | --- |
| hsa04060 | Cytokine-cytokine receptor interaction | 45/533 | 7.26E-08 | 2.16E-05 | 45 |
| hsa04668 | TNF signaling pathway | 21/533 | 1.03E-05 | 0.001118 | 21 |
| hsa04064 | NF-kappa B signaling pathway | 20/533 | 1.13E-05 | 0.001118 | 20 |
| hsa05169 | Epstein-Barr virus infection | 30/533 | 1.98E-05 | 0.001477 | 30 |
| hsa05164 | Influenza A | 26/533 | 4.70E-05 | 0.002801 | 26 |
| hsa04621 | NOD-like receptor signaling pathway | 27/533 | 6.36E-05 | 0.003161 | 27 |
| hsa05133 | Pertussis | 15/533 | 0.000103 | 0.004375 | 15 |
| hsa04145 | Phagosome | 23/533 | 0.000137 | 0.00512 | 23 |
| hsa05167 | Kaposi sarcoma-associated herpesvirus infection | 27/533 | 0.000159 | 0.005262 | 27 |
| hsa04940 | Type I diabetes mellitus | 10/533 | 0.000369 | 0.011008 | 10 |
| hsa04010 | MAPK signaling pathway | 35/533 | 0.000421 | 0.011404 | 35 |
| hsa05150 | Staphylococcus aureus infection | 16/533 | 0.000473 | 0.011747 | 16 |
| hsa05171 | Coronavirus disease - COVID-19 | 29/533 | 0.000588 | 0.013489 | 29 |
| hsa05162 | Measles | 20/533 | 0.000719 | 0.015306 | 20 |
| hsa05323 | Rheumatoid arthritis | 15/533 | 0.001002 | 0.019307 | 15 |
| hsa05134 | Legionellosis | 11/533 | 0.001037 | 0.019307 | 11 |
| hsa05168 | Herpes simplex virus 1 infection | 50/533 | 0.001371 | 0.02304 | 50 |
| hsa05140 | Leishmaniasis | 13/533 | 0.001392 | 0.02304 | 13 |
| hsa04612 | Antigen processing and presentation | 13/533 | 0.001572 | 0.024482 | 13 |
| hsa04380 | Osteoclast differentiation | 18/533 | 0.001703 | 0.024482 | 18 |
| hsa04514 | Cell adhesion molecules | 20/533 | 0.001725 | 0.024482 | 20 |
| hsa04630 | JAK-STAT signaling pathway | 21/533 | 0.002077 | 0.027524 | 21 |
| hsa04061 | Viral protein interaction with cytokine and cytokine receptor | 15/533 | 0.002124 | 0.027524 | 15 |
| hsa05143 | African trypanosomiasis | 8/533 | 0.002342 | 0.029082 | 8 |
| hsa05330 | Allograft rejection | 8/533 | 0.0028 | 0.033371 | 8 |
| hsa04610 | Complement and coagulation cascades | 13/533 | 0.003458 | 0.03963 | 13 |
| hsa05412 | Arrhythmogenic right ventricular cardiomyopathy | 12/533 | 0.004175 | 0.046079 | 12 |

**Supplementary Table 16. The pathway enrichment of DEGs in HK-2 cells of 24h**

| ID | Description | GeneRatio | pvalue | p.adjust | Count |
| --- | --- | --- | --- | --- | --- |
| hsa04061 | Viral protein interaction with cytokine and cytokine receptor | 24/619 | 3.12E-07 | 9.93E-05 | 24 |
| hsa04060 | Cytokine-cytokine receptor interaction | 47/619 | 8.50E-07 | 0.000135 | 47 |
| hsa00100 | Steroid biosynthesis | 9/619 | 6.52E-06 | 0.000691 | 9 |
| hsa05164 | Influenza A | 29/619 | 3.43E-05 | 0.002729 | 29 |
| hsa04514 | Cell adhesion molecules | 26/619 | 5.28E-05 | 0.003355 | 26 |
| hsa04621 | NOD-like receptor signaling pathway | 29/619 | 0.000134 | 0.006099 | 29 |
| hsa03320 | PPAR signaling pathway | 16/619 | 0.000134 | 0.006099 | 16 |
| hsa04668 | TNF signaling pathway | 19/619 | 0.000753 | 0.027176 | 19 |
| hsa05160 | Hepatitis C | 24/619 | 0.000782 | 0.027176 | 24 |
| hsa00900 | Terpenoid backbone biosynthesis | 7/619 | 0.000905 | 0.027176 | 7 |
| hsa04670 | Leukocyte transendothelial migration | 19/619 | 0.00094 | 0.027176 | 19 |
| hsa04145 | Phagosome | 23/619 | 0.00115 | 0.029511 | 23 |
| hsa00350 | Tyrosine metabolism | 9/619 | 0.001206 | 0.029511 | 9 |
| hsa04360 | Axon guidance | 26/619 | 0.001347 | 0.030599 | 26 |
| hsa05167 | Kaposi sarcoma-associated herpesvirus infection | 27/619 | 0.001627 | 0.030876 | 27 |
| hsa05323 | Rheumatoid arthritis | 16/619 | 0.001649 | 0.030876 | 16 |
| hsa05416 | Viral myocarditis | 12/619 | 0.001651 | 0.030876 | 12 |
| hsa05130 | Pathogenic Escherichia coli infection | 27/619 | 0.002041 | 0.035022 | 27 |
| hsa04064 | NF-kappa B signaling pathway | 17/619 | 0.002126 | 0.035022 | 17 |
| hsa04530 | Tight junction | 24/619 | 0.002203 | 0.035022 | 24 |
| hsa04540 | Gap junction | 15/619 | 0.002503 | 0.037899 | 15 |
| hsa04270 | Vascular smooth muscle contraction | 20/619 | 0.002779 | 0.040174 | 20 |
| hsa05169 | Epstein-Barr virus infection | 27/619 | 0.002932 | 0.040535 | 27 |

**Supplementary Table 17. DEGs in JEG-3 cells of 3h**

| gene | pvalue | FC | gene | pvalue | FC |
| --- | --- | --- | --- | --- | --- |
| ANKRD1 | 0.035469 | 20.74134 | MNX1 | 0.034601 | 0.477705 |
| BTBD19 | 0.001038 | 16.30727 | XRRA1 | 0.048867 | 0.477204 |
| ART4 | 0.014417 | 13.37362 | CTTNBP2NL | 0.010071 | 0.475926 |
| NPIPA2 | 0.028256 | 13.26624 | ADCY6 | 0.036675 | 0.474377 |
| EDN1 | 0.048564 | 12.44762 | TSC22D4 | 0.000878 | 0.473181 |
| THBS1 | 0.009214 | 11.65298 | ST8SIA1 | 0.032353 | 0.47184 |
| EGR1 | 0.002676 | 11.5255 | COL16A1 | 0.034696 | 0.47152 |
| IQUB | 0.038182 | 11.20818 | ABCB9 | 0.02536 | 0.47018 |
| IRF8 | 0.002109 | 10.69036 | MT2A | 0.011436 | 0.470027 |
| ZAR1L | 0.000287 | 9.366862 | WNT7B | 0.001709 | 0.469855 |
| TNF | 0.013591 | 9.253025 | ATG9A | 0.016973 | 0.469159 |
| AXDND1 | 0.020264 | 8.852855 | PBX3 | 0.02632 | 0.466542 |
| PSG5 | 0.003585 | 8.104296 | HLX | 0.040615 | 0.465733 |
| BDKRB2 | 0.018612 | 7.55527 | UBN2 | 0.022944 | 0.463181 |
| MAOA | 0.000865 | 7.44447 | FOXD1 | 0.039813 | 0.462059 |
| IL10RA | 0.042733 | 7.159235 | MEX3D | 0.019165 | 0.460474 |
| MMP24 | 0.044694 | 6.972686 | PRRT3 | 0.035918 | 0.459978 |
| TCIM | 0.007222 | 6.831038 | PDE4A | 0.026638 | 0.459701 |
| SGPP2 | 4.08E-05 | 6.745252 | INTS6 | 0.012413 | 0.458791 |
| KLF7 | 0.024226 | 6.729926 | NOTCH1 | 0.015408 | 0.457698 |
| RHOH | 0.021482 | 6.51171 | HSPA8 | 0.000784 | 0.457483 |
| HTRA3 | 0.016444 | 6.300618 | MYPOP | 0.031288 | 0.455839 |
| APOD | 0.030734 | 6.151869 | NXPH4 | 0.027712 | 0.455181 |
| CLIP4 | 0.014037 | 5.862212 | ANKZF1 | 0.002023 | 0.453507 |
| CDC42 | 0.038198 | 5.64921 | GAL3ST1 | 0.026362 | 0.450016 |
| SPRY4 | 0.009184 | 5.574445 | FAM189B | 0.00479 | 0.448874 |
| ROPN1L | 0.030045 | 5.442002 | TERT | 0.000382 | 0.446552 |
| JUN | 0.013432 | 5.153899 | SPOP | 0.000281 | 0.44507 |
| SYT16 | 0.01245 | 5.038223 | ZNF865 | 0.032857 | 0.44448 |
| RADIL | 0.000176 | 4.878073 | CHST12 | 0.009843 | 0.444064 |
| TNFSF14 | 0.048801 | 4.658015 | TTBK2 | 0.007528 | 0.441628 |
| DLX2 | 0.042821 | 4.499941 | UPF2 | 0.003446 | 0.440573 |
| NPPB | 0.008772 | 4.328724 | IER5L | 0.023113 | 0.438472 |
| FGF1 | 0.014916 | 4.306765 | MAPRE3 | 0.026511 | 0.437944 |
| PP2D1 | 0.034886 | 4.276731 | PKD1 | 0.042134 | 0.436879 |
| SULT2A1 | 0.033555 | 4.252207 | ARVCF | 0.002561 | 0.436423 |
| DENND2C | 0.035216 | 4.086132 | LMTK3 | 0.035469 | 0.435418 |
| RGS2 | 0.032101 | 4.069835 | SIX5 | 0.004704 | 0.434435 |
| PLA2G10 | 0.047097 | 4.061125 | RIMKLA | 0.031482 | 0.434034 |
| CDYL2 | 0.023661 | 3.881945 | DPYSL5 | 0.006089 | 0.434012 |
| LMOD1 | 0.003032 | 3.873958 | MYCT1 | 0.001642 | 0.432486 |
| HEG1 | 2.91E-06 | 3.79554 | SLC6A9 | 0.005689 | 0.432305 |
| SPANXB1 | 0.022518 | 3.783086 | FOXG1 | 0.015666 | 0.430074 |
| FOXC1 | 0.012335 | 3.781016 | ZCCHC14 | 0.004443 | 0.429772 |
| RASGEF1B | 0.015515 | 3.635893 | SUFU | 0.004707 | 0.429664 |
| CTGF | 0.014935 | 3.514656 | PARP10 | 0.046526 | 0.42898 |
| C4BPB | 0.037207 | 3.492694 | CARD11 | 0.038473 | 0.428104 |
| MAGI2 | 0.01547 | 3.449164 | PRICKLE3 | 0.031902 | 0.423643 |
| ADHFE1 | 0.019845 | 3.44183 | RANGRF | 0.034297 | 0.421375 |
| GLIPR1 | 0.019168 | 3.441007 | BMT2 | 0.00133 | 0.420029 |
| NAALADL2 | 0.041362 | 3.429819 | SOBP | 0.043986 | 0.417841 |
| HPGDS | 0.045021 | 3.366958 | DDIT4 | 0.014301 | 0.417086 |
| CCDC178 | 0.027916 | 3.310982 | ZBTB4 | 0.005302 | 0.416242 |
| LMCD1 | 0.002307 | 3.206412 | TRIM9 | 0.003866 | 0.414368 |
| KCNJ15 | 0.042715 | 3.182563 | HELZ2 | 0.026551 | 0.413328 |
| RUBCNL | 0.031629 | 3.167552 | LRRC37A3 | 0.047862 | 0.412601 |
| TMEM52B | 0.045414 | 2.989405 | ZNF668 | 0.016348 | 0.41221 |
| GIP | 0.006376 | 2.979519 | EPN3 | 0.011578 | 0.411473 |
| TRIL | 0.03059 | 2.932469 | AL136454.1 | 0.037056 | 0.409412 |
| SMAD3 | 0.044342 | 2.918135 | ZNF512 | 0.008814 | 0.409324 |
| CCIN | 0.004206 | 2.89776 | RBBP8NL | 0.013332 | 0.407532 |
| ID3 | 0.010569 | 2.809991 | CCDC142 | 0.011756 | 0.407063 |
| TFF1 | 0.030561 | 2.778466 | LCA5 | 0.037359 | 0.40495 |
| FA2H | 0.006257 | 2.731835 | DISP1 | 0.026941 | 0.404038 |
| GBP1 | 0.007417 | 2.717087 | RHOBTB2 | 0.005012 | 0.40048 |
| CACNA1S | 0.049316 | 2.614642 | ZNF594 | 0.015352 | 0.399897 |
| CYR61 | 0.000295 | 2.611136 | PHF19 | 0.040058 | 0.398933 |
| POMZP3 | 0.023553 | 2.592195 | YEATS2 | 0.005798 | 0.397729 |
| RELB | 0.039729 | 2.574754 | ADAM22 | 0.000766 | 0.397544 |
| NOCT | 0.00334 | 2.518715 | APAF1 | 0.001031 | 0.397431 |
| FAM111A | 0.025195 | 2.48951 | HSPA1A | 0.004168 | 0.394516 |
| GREM2 | 0.00165 | 2.43992 | IRF2 | 0.009302 | 0.394444 |
| ZSCAN31 | 0.024802 | 2.315264 | AHDC1 | 0.005245 | 0.384886 |
| HOXB3 | 0.034139 | 2.314525 | ZNF382 | 0.037835 | 0.382364 |
| TUBB2B | 0.013922 | 2.299664 | FP565260.3 | 0.046502 | 0.379869 |
| GATD3B | 0.031015 | 2.29262 | GLIS2 | 0.013295 | 0.379149 |
| TRIM16L | 0.005901 | 2.291505 | RCOR2 | 0.043996 | 0.375488 |
| PIK3IP1 | 0.028694 | 2.257797 | NPR2 | 0.011977 | 0.37459 |
| PAM16 | 0.006089 | 2.249531 | NXPH3 | 0.043405 | 0.37146 |
| NR1D2 | 0.004748 | 2.245928 | RIN1 | 0.013686 | 0.370514 |
| LILRB3 | 0.049382 | 2.245323 | APPBP2 | 0.000391 | 0.370299 |
| CTAGE1 | 0.00871 | 2.238709 | CLDN15 | 0.01114 | 0.36838 |
| CEACAM1 | 0.020009 | 2.230569 | NKX3-2 | 0.025413 | 0.367352 |
| DNAJC15 | 0.022749 | 2.228038 | CUL9 | 0.007531 | 0.366042 |
| DUSP4 | 0.044105 | 2.226485 | SOX12 | 0.028101 | 0.359455 |
| PTPMT1 | 0.039207 | 2.212671 | FCHO1 | 0.028161 | 0.354265 |
| TIGAR | 0.01341 | 2.212269 | EPHB3 | 0.021017 | 0.353612 |
| EPHA2 | 0.007886 | 2.211161 | PPP3R1 | 0.004452 | 0.350189 |
| ZNF19 | 0.044679 | 2.202909 | STON1 | 0.035526 | 0.349293 |
| TAGLN | 0.011304 | 2.187588 | KIF26A | 0.039449 | 0.346808 |
| KCNK1 | 0.001222 | 2.183941 | CAMSAP3 | 0.014948 | 0.336451 |
| FAM161B | 0.038619 | 2.183779 | ANGPTL2 | 0.034044 | 0.335734 |
| ACTG2 | 0.002809 | 2.157664 | KREMEN2 | 0.019026 | 0.333169 |
| BAMBI | 0.001395 | 2.155008 | CALML6 | 0.045309 | 0.322152 |
| TFPI2 | 0.021265 | 2.139619 | HSPA1B | 5.51E-05 | 0.317685 |
| TIMM23B | 0.0461 | 2.120946 | ZAP70 | 0.002093 | 0.316606 |
| ZNF18 | 0.041287 | 2.114857 | TNFRSF25 | 0.019418 | 0.315869 |
| ADGRF4 | 0.046023 | 2.113023 | CACNB3 | 0.008738 | 0.315731 |
| RND3 | 0.019251 | 2.086975 | SLC25A45 | 0.04166 | 0.314637 |
| C6orf226 | 0.022126 | 2.07893 | PROB1 | 0.006483 | 0.314378 |
| STK32C | 0.032309 | 2.066757 | EPO | 0.044556 | 0.314372 |
| FICD | 0.035306 | 2.050542 | TMEM159 | 0.011408 | 0.308009 |
| TVP23C | 0.004621 | 2.041927 | SLC4A11 | 0.032793 | 0.307916 |
| HAGHL | 0.0404 | 2.027923 | LRRC63 | 0.0425 | 0.301807 |
| FOXO1 | 0.000317 | 2.026486 | MYOD1 | 0.039574 | 0.299249 |
| SUMF2 | 0.0048 | 2.014491 | FAM78A | 0.000328 | 0.294777 |
| HIVEP2 | 0.011402 | 2.011182 | ZNF835 | 0.004448 | 0.292395 |
| ID2 | 0.009571 | 2.004288 | LIPH | 0.005298 | 0.288925 |
| THRA | 0.040967 | 0.499641 | TTLL7 | 0.032242 | 0.284129 |
| ZFC3H1 | 0.027323 | 0.499578 | RAB3IL1 | 0.00692 | 0.276819 |
| GIT1 | 0.004323 | 0.499488 | RBM14-RBM4 | 0.032115 | 0.270835 |
| FOXP4 | 0.014538 | 0.499365 | IP6K3 | 0.007729 | 0.270779 |
| RABEP2 | 0.004874 | 0.498425 | ZIC2 | 0.005404 | 0.268292 |
| NEURL4 | 0.047735 | 0.498189 | FAM83F | 0.041188 | 0.265475 |
| ARHGEF40 | 0.015624 | 0.497121 | PPP1R18 | 0.000351 | 0.265267 |
| CFAP44 | 0.040483 | 0.496518 | 3-Mar | 0.001607 | 0.263041 |
| CIPC | 0.033758 | 0.495775 | THBS2 | 0.008881 | 0.260469 |
| DENND4C | 0.006865 | 0.495731 | HSPA1L | 0.000316 | 0.244051 |
| PPP2R5B | 0.014688 | 0.495473 | SATB2 | 0.002375 | 0.234084 |
| EGLN1 | 0.004279 | 0.495204 | CBARP | 0.039314 | 0.231575 |
| ZFYVE1 | 0.005234 | 0.494311 | MEIOC | 0.042686 | 0.230203 |
| PHETA1 | 0.00802 | 0.493502 | HS3ST3A1 | 0.004693 | 0.229475 |
| GPSM1 | 0.027769 | 0.493332 | TMEM91 | 0.032354 | 0.225325 |
| OR8A1 | 0.043648 | 0.491981 | RNF223 | 0.043857 | 0.224284 |
| BLOC1S5 | 0.000996 | 0.491456 | BST2 | 0.031922 | 0.220459 |
| THSD7A | 0.014715 | 0.490912 | AURKC | 0.021974 | 0.210895 |
| DEDD2 | 0.001793 | 0.490069 | GPR161 | 0.044044 | 0.20126 |
| HS6ST1 | 0.013535 | 0.48783 | ZNF343 | 0.022001 | 0.196585 |
| MAP3K10 | 0.004157 | 0.487737 | GRIN1 | 0.015566 | 0.180469 |
| ZNF629 | 0.022479 | 0.487261 | ADGRG7 | 0.006832 | 0.177251 |
| IFNLR1 | 0.01485 | 0.48457 | EFNA3 | 0.028392 | 0.176031 |
| ZBTB46 | 0.002297 | 0.484428 | DLG4 | 0.00642 | 0.172046 |
| EPHX2 | 0.015222 | 0.484419 | CCDC60 | 0.039158 | 0.161536 |
| FIGNL2 | 0.014191 | 0.483986 | BBS1 | 0.041931 | 0.145844 |
| C1orf216 | 0.041336 | 0.483856 | C11orf52 | 0.019423 | 0.1413 |
| ATF6B | 0.01374 | 0.481683 | ARHGEF25 | 0.008625 | 0.127986 |
| CFAP157 | 0.027295 | 0.481009 | ALKAL2 | 0.008834 | 0.112708 |
| ARHGAP39 | 0.03772 | 0.47955 | HOXA5 | 0.003979 | 0.111383 |
| DBP | 0.034164 | 0.478889 | HIC1 | 0.0233 | 0.104789 |
| HERC4 | 0.025572 | 0.478683 | TIMD4 | 0.021638 | 0.10047 |
| KLF15 | 0.025114 | 0.478661 | ITGA10 | 0.001734 | 0.090647 |
| IRF2BP1 | 0.03468 | 0.478606 | INO80B | 0.036505 | 0.069381 |
| EFNB1 | 0.044742 | 0.478482 | REPS2 | 0.048019 | 0.065177 |
| ZNF221 | 0.005943 | 0.060226 |  |  |  |

**Supplementary Table 18. DEGs in JEG-3 cells of 12h**

| gene | pvalue | FC | gene | pvalue | FC |
| --- | --- | --- | --- | --- | --- |
| CD22 | 0.01601 | 0.12113 | DYRK4 | 0.015723 | 2.479947 |
| REPS2 | 0.046443 | 0.152912 | GATD3B | 0.04252 | 2.568896 |
| ASPHD1 | 0.035667 | 0.18595 | IPO4 | 0.00385 | 2.609821 |
| DPPA4 | 0.035667 | 0.18595 | KIF26B | 0.030389 | 2.664499 |
| COLQ | 0.045826 | 0.197333 | CES1 | 0.02404 | 2.750241 |
| DLG4 | 0.009784 | 0.201331 | AL031708.1 | 0.014752 | 2.938905 |
| HSF2BP | 0.018797 | 0.223766 | DUSP27 | 0.014506 | 2.965422 |
| NLRP1 | 0.021659 | 0.226244 | IFITM1 | 0.016521 | 2.978418 |
| CATSPERE | 0.034152 | 0.229009 | OR3A1 | 0.039595 | 3.033557 |
| CLEC18A | 0.046888 | 0.256998 | TMEM37 | 0.027462 | 3.065707 |
| GAL3ST1 | 0.007864 | 0.258477 | PLSCR4 | 0.027822 | 3.080483 |
| ZNF490 | 0.023873 | 0.281743 | FAM186A | 0.033414 | 3.554331 |
| UNC5CL | 0.031289 | 0.29971 | MYEF2 | 0.036215 | 3.628588 |
| RSPH14 | 0.046115 | 0.338555 | PCDHGA9 | 0.019052 | 3.671424 |
| CHRM5 | 0.025813 | 0.349244 | SERF1B | 0.047031 | 3.719575 |
| HSPA1L | 0.041017 | 0.362808 | KHDC1L | 0.036251 | 3.785564 |
| ANK2 | 0.033746 | 0.366815 | MMRN2 | 0.037256 | 4.058356 |
| TSPAN7 | 0.026525 | 0.37241 | CCDC146 | 0.016131 | 4.114757 |
| KIAA1549L | 0.046583 | 0.37921 | ARSD | 0.043703 | 4.282606 |
| ISL2 | 0.024367 | 0.392979 | GDPD4 | 0.015164 | 4.377979 |
| NMB | 0.009338 | 0.394532 | YY2 | 0.011457 | 4.484178 |
| PCDHB12 | 0.024664 | 0.403659 | DENND2C | 0.026472 | 4.909268 |
| NXPH4 | 0.013967 | 0.429632 | KCNV2 | 0.039798 | 4.982064 |
| PPM1J | 0.037362 | 0.429633 | TACR3 | 0.022028 | 5.121121 |
| KREMEN2 | 0.032661 | 0.431012 | CACNA1S | 0.035675 | 5.136471 |
| GLDN | 0.049645 | 0.486926 | VILL | 0.017042 | 5.258481 |
| SLCO3A1 | 0.046353 | 0.493785 | PP2D1 | 0.019849 | 5.40133 |
| LPP | 0.023036 | 2.005425 | AC136612.1 | 0.030468 | 5.695306 |
| NBPF10 | 0.048924 | 2.007286 | VWA7 | 0.03603 | 5.742656 |
| TIRAP | 0.027655 | 2.012943 | IL1R2 | 0.009573 | 6.163137 |
| KRT17 | 0.024966 | 2.08614 | ERAP2 | 0.033202 | 6.283055 |
| TMEM52B | 0.035741 | 2.169161 | NRIP2 | 0.022246 | 6.851899 |
| FAP | 0.042769 | 2.2403 | ITPKB | 0.011426 | 6.941346 |
| POTEE | 0.047127 | 2.257453 | ROPN1L | 0.011804 | 7.342824 |
| TMEM236 | 0.029331 | 2.307891 | ZEB2 | 0.013137 | 7.366499 |
| SPNS2 | 0.002551 | 2.314365 | APOD | 0.003974 | 9.315829 |
| ZNF155 | 0.024471 | 2.340119 | FITM1 | 0.042618 | 10.2877 |
| ANKRD1 | 0.049031 | 2.379619 | ZBED6 | 0.040282 | 10.35284 |
| CRACR2A | 0.013351 | 2.393168 | FCGR1A | 0.027826 | 13.21449 |
| EPB41L4A | 0.024129 | 2.444902 | EHD2 | 0.03016 | 13.42105 |
| ADAM11 | 0.001105 | 17.18062 | ANKDD1B | 0.021157 | 13.93293 |

**Supplementary Table 19. The GO-BP enrichment of DEGs in JEG-3 cells of 3h**

| ID | Description | GeneRatio | pvalue | p.adjust | Count |
| --- | --- | --- | --- | --- | --- |
| GO:0048568 | embryonic organ development | 21/257 | 2.45E-07 | 0.000855 | 21 |
| GO:0048663 | neuron fate commitment | 8/257 | 1.30E-06 | 0.002258 | 8 |
| GO:0055123 | digestive system development | 11/257 | 2.28E-06 | 0.002646 | 11 |
| GO:0048732 | gland development | 19/257 | 4.21E-06 | 0.003393 | 19 |
| GO:0045668 | negative regulation of osteoblast differentiation | 7/257 | 4.87E-06 | 0.003393 | 7 |
| GO:0061448 | connective tissue development | 14/257 | 6.55E-06 | 0.003805 | 14 |
| GO:0048565 | digestive tract development | 10/257 | 8.32E-06 | 0.004141 | 10 |
| GO:0019216 | regulation of lipid metabolic process | 18/257 | 1.09E-05 | 0.004561 | 18 |
| GO:0051090 | regulation of DNA-binding transcription factor activity | 19/257 | 1.18E-05 | 0.004561 | 19 |
| GO:0048562 | embryonic organ morphogenesis | 14/257 | 2.85E-05 | 0.009942 | 14 |
| GO:0051051 | negative regulation of transport | 18/257 | 3.42E-05 | 0.010839 | 18 |
| GO:0072073 | kidney epithelium development | 9/257 | 7.84E-05 | 0.021747 | 9 |
| GO:0007389 | pattern specification process | 17/257 | 8.11E-05 | 0.021747 | 17 |
| GO:0060428 | lung epithelium development | 5/257 | 9.10E-05 | 0.022646 | 5 |
| GO:0072009 | nephron epithelium development | 8/257 | 0.000102 | 0.02362 | 8 |
| GO:0072006 | nephron development | 9/257 | 0.000117 | 0.024604 | 9 |
| GO:0001822 | kidney development | 13/257 | 0.00013 | 0.024604 | 13 |
| GO:0001503 | ossification | 16/257 | 0.000132 | 0.024604 | 16 |
| GO:0001655 | urogenital system development | 14/257 | 0.000134 | 0.024604 | 14 |
| GO:0003002 | regionalization | 14/257 | 0.000163 | 0.025744 | 14 |
| GO:0072001 | renal system development | 13/257 | 0.000171 | 0.025744 | 13 |
| GO:0048013 | ephrin receptor signaling pathway | 7/257 | 0.000173 | 0.025744 | 7 |
| GO:0043620 | regulation of DNA-templated transcription in response to stress | 8/257 | 0.00018 | 0.025744 | 8 |
| GO:0009187 | cyclic nucleotide metabolic process | 5/257 | 0.000201 | 0.025744 | 5 |
| GO:1902895 | positive regulation of pri-miRNA transcription by RNA polymerase II | 5/257 | 0.000201 | 0.025744 | 5 |
| GO:0030878 | thyroid gland development | 4/257 | 0.000203 | 0.025744 | 4 |
| GO:1903306 | negative regulation of regulated secretory pathway | 4/257 | 0.000203 | 0.025744 | 4 |
| GO:0043500 | muscle adaptation | 8/257 | 0.000214 | 0.025744 | 8 |
| GO:0051216 | cartilage development | 10/257 | 0.000232 | 0.025744 | 10 |
| GO:0034605 | cellular response to heat | 8/257 | 0.000241 | 0.025744 | 8 |
| GO:0034763 | negative regulation of transmembrane transport | 8/257 | 0.000241 | 0.025744 | 8 |
| GO:0042026 | protein refolding | 4/257 | 0.000243 | 0.025744 | 4 |
| GO:0033002 | muscle cell proliferation | 11/257 | 0.000244 | 0.025744 | 11 |
| GO:0060541 | respiratory system development | 10/257 | 0.000265 | 0.027119 | 10 |
| GO:0070482 | response to oxygen levels | 15/257 | 0.000275 | 0.027364 | 15 |
| GO:0009266 | response to temperature stimulus | 11/257 | 0.000306 | 0.029114 | 11 |
| GO:0048546 | digestive tract morphogenesis | 5/257 | 0.000318 | 0.029114 | 5 |
| GO:0019932 | second-messenger-mediated signaling | 13/257 | 0.000318 | 0.029114 | 13 |
| GO:0010611 | regulation of cardiac muscle hypertrophy | 6/257 | 0.000326 | 0.029114 | 6 |
| GO:0003300 | cardiac muscle hypertrophy | 7/257 | 0.000341 | 0.029114 | 7 |
| GO:0071453 | cellular response to oxygen levels | 11/257 | 0.000343 | 0.029114 | 11 |
| GO:0045667 | regulation of osteoblast differentiation | 8/257 | 0.000373 | 0.030946 | 8 |
| GO:0001945 | lymph vessel development | 4/257 | 0.000397 | 0.031228 | 4 |
| GO:0030324 | lung development | 9/257 | 0.000407 | 0.031228 | 9 |
| GO:0014897 | striated muscle hypertrophy | 7/257 | 0.000412 | 0.031228 | 7 |
| GO:0014743 | regulation of muscle hypertrophy | 6/257 | 0.000412 | 0.031228 | 6 |
| GO:0031589 | cell-substrate adhesion | 14/257 | 0.000433 | 0.031863 | 14 |
| GO:0007411 | axon guidance | 12/257 | 0.000439 | 0.031863 | 12 |
| GO:0097485 | neuron projection guidance | 12/257 | 0.000453 | 0.032234 | 12 |
| GO:0014896 | muscle hypertrophy | 7/257 | 0.000465 | 0.032424 | 7 |
| GO:0030323 | respiratory tube development | 9/257 | 0.000486 | 0.033179 | 9 |
| GO:0031113 | regulation of microtubule polymerization | 5/257 | 0.00058 | 0.038884 | 5 |
| GO:1903706 | regulation of hemopoiesis | 15/257 | 0.000602 | 0.039039 | 15 |
| GO:1903579 | negative regulation of ATP metabolic process | 4/257 | 0.000612 | 0.039039 | 4 |
| GO:0001889 | liver development | 8/257 | 0.000616 | 0.039039 | 8 |
| GO:1902893 | regulation of pri-miRNA transcription by RNA polymerase II | 5/257 | 0.000636 | 0.039514 | 5 |
| GO:0060576 | intestinal epithelial cell development | 3/257 | 0.000646 | 0.039514 | 3 |
| GO:0030099 | myeloid cell differentiation | 15/257 | 0.000664 | 0.039771 | 15 |
| GO:0061008 | hepaticobiliary system development | 8/257 | 0.000677 | 0.039771 | 8 |
| GO:0045165 | cell fate commitment | 11/257 | 0.000687 | 0.039771 | 11 |
| GO:0061614 | pri-miRNA transcription by RNA polymerase II | 5/257 | 0.000696 | 0.039771 | 5 |
| GO:0043618 | regulation of transcription from RNA polymerase II promoter in response to stress | 7/257 | 0.000735 | 0.040827 | 7 |
| GO:0043433 | negative regulation of DNA-binding transcription factor activity | 9/257 | 0.000738 | 0.040827 | 9 |
| GO:0048754 | branching morphogenesis of an epithelial tube | 8/257 | 0.000779 | 0.041877 | 8 |
| GO:0045879 | negative regulation of smoothened signaling pathway | 4/257 | 0.000793 | 0.041877 | 4 |
| GO:0045920 | negative regulation of exocytosis | 4/257 | 0.000793 | 0.041877 | 4 |
| GO:0045820 | negative regulation of glycolytic process | 3/257 | 0.000814 | 0.041972 | 3 |
| GO:1903959 | regulation of anion transmembrane transport | 7/257 | 0.000819 | 0.041972 | 7 |
| GO:0031110 | regulation of microtubule polymerization or depolymerization | 6/257 | 0.000834 | 0.042025 | 6 |
| GO:0010810 | regulation of cell-substrate adhesion | 10/257 | 0.000844 | 0.042025 | 10 |
| GO:0016525 | negative regulation of angiogenesis | 8/257 | 0.000891 | 0.042811 | 8 |
| GO:1901099 | negative regulation of signal transduction in absence of ligand | 4/257 | 0.000897 | 0.042811 | 4 |
| GO:2001240 | negative regulation of extrinsic apoptotic signaling pathway in absence of ligand | 4/257 | 0.000897 | 0.042811 | 4 |
| GO:2000181 | negative regulation of blood vessel morphogenesis | 8/257 | 0.000974 | 0.045861 | 8 |
| GO:0001649 | osteoblast differentiation | 10/257 | 0.001003 | 0.046611 | 10 |
| GO:1901343 | negative regulation of vasculature development | 8/257 | 0.001017 | 0.046643 | 8 |
| GO:0001763 | morphogenesis of a branching structure | 9/257 | 0.001049 | 0.047457 | 9 |
| GO:0014706 | striated muscle tissue development | 13/257 | 0.001107 | 0.049318 | 13 |
| GO:0010614 | negative regulation of cardiac muscle hypertrophy | 4/257 | 0.001132 | 0.049318 | 4 |
| GO:0051085 | chaperone cofactor-dependent protein refolding | 4/257 | 0.001132 | 0.049318 | 4 |

**Supplementary Table 20. The GO-BP enrichment of up-regulated DEGs in JEG-3 cells of 3h**

| ID | Description | GeneRatio | pvalue | p.adjust | Count |
| --- | --- | --- | --- | --- | --- |
| GO:0019216 | regulation of lipid metabolic process | 15/98 | 2.39E-09 | 5.80E-06 | 15 |
| GO:0045668 | negative regulation of osteoblast differentiation | 5/98 | 6.01E-06 | 0.007283 | 5 |
| GO:0032922 | circadian regulation of gene expression | 5/98 | 2.56E-05 | 0.01386 | 5 |
| GO:0010611 | regulation of cardiac muscle hypertrophy | 5/98 | 2.75E-05 | 0.01386 | 5 |
| GO:0014743 | regulation of muscle hypertrophy | 5/98 | 3.39E-05 | 0.01386 | 5 |
| GO:0061045 | negative regulation of wound healing | 5/98 | 4.14E-05 | 0.01386 | 5 |
| GO:0042326 | negative regulation of phosphorylation | 10/98 | 5.06E-05 | 0.01386 | 10 |
| GO:1902895 | positive regulation of pri-miRNA transcription by RNA polymerase II | 4/98 | 5.42E-05 | 0.01386 | 4 |
| GO:0048568 | embryonic organ development | 10/98 | 5.61E-05 | 0.01386 | 10 |
| GO:0048732 | gland development | 10/98 | 5.72E-05 | 0.01386 | 10 |
| GO:0014706 | striated muscle tissue development | 9/98 | 8.87E-05 | 0.017489 | 9 |
| GO:0002573 | myeloid leukocyte differentiation | 7/98 | 9.40E-05 | 0.017489 | 7 |
| GO:1903035 | negative regulation of response to wounding | 5/98 | 0.000106 | 0.017489 | 5 |
| GO:0007623 | circadian rhythm | 7/98 | 0.00012 | 0.017489 | 7 |
| GO:0060537 | muscle tissue development | 9/98 | 0.000135 | 0.017489 | 9 |
| GO:1902893 | regulation of pri-miRNA transcription by RNA polymerase II | 4/98 | 0.000142 | 0.017489 | 4 |
| GO:0003300 | cardiac muscle hypertrophy | 5/98 | 0.000144 | 0.017489 | 5 |
| GO:0048661 | positive regulation of smooth muscle cell proliferation | 5/98 | 0.000144 | 0.017489 | 5 |
| GO:0030150 | protein import into mitochondrial matrix | 3/98 | 0.000145 | 0.017489 | 3 |
| GO:0043502 | regulation of muscle adaptation | 5/98 | 0.000151 | 0.017489 | 5 |
| GO:0061614 | pri-miRNA transcription by RNA polymerase II | 4/98 | 0.000153 | 0.017489 | 4 |
| GO:0033002 | muscle cell proliferation | 7/98 | 0.000159 | 0.017489 | 7 |
| GO:0014897 | striated muscle hypertrophy | 5/98 | 0.000166 | 0.017489 | 5 |
| GO:0014896 | muscle hypertrophy | 5/98 | 0.000182 | 0.018005 | 5 |
| GO:0048660 | regulation of smooth muscle cell proliferation | 6/98 | 0.000188 | 0.018005 | 6 |
| GO:0019932 | second-messenger-mediated signaling | 8/98 | 0.000199 | 0.018005 | 8 |
| GO:0048659 | smooth muscle cell proliferation | 6/98 | 0.000201 | 0.018005 | 6 |
| GO:0007517 | muscle organ development | 8/98 | 0.000247 | 0.021379 | 8 |
| GO:0032890 | regulation of organic acid transport | 4/98 | 0.000267 | 0.021448 | 4 |
| GO:0090288 | negative regulation of cellular response to growth factor stimulus | 5/98 | 0.000272 | 0.021448 | 5 |
| GO:0022612 | gland morphogenesis | 5/98 | 0.000283 | 0.021448 | 5 |
| GO:1903959 | regulation of anion transmembrane transport | 5/98 | 0.000283 | 0.021448 | 5 |
| GO:0001945 | lymph vessel development | 3/98 | 0.000324 | 0.023251 | 3 |
| GO:0030099 | myeloid cell differentiation | 9/98 | 0.000332 | 0.023251 | 9 |
| GO:0071560 | cellular response to transforming growth factor beta stimulus | 7/98 | 0.000336 | 0.023251 | 7 |
| GO:0043500 | muscle adaptation | 5/98 | 0.000376 | 0.024735 | 5 |
| GO:0071559 | response to transforming growth factor beta | 7/98 | 0.000387 | 0.024735 | 7 |
| GO:0050673 | epithelial cell proliferation | 9/98 | 0.000388 | 0.024735 | 9 |
| GO:0010812 | negative regulation of cell-substrate adhesion | 4/98 | 0.000409 | 0.024746 | 4 |
| GO:1901224 | positive regulation of NIK/NF-kappaB signaling | 4/98 | 0.000409 | 0.024746 | 4 |
| GO:0042476 | odontogenesis | 5/98 | 0.000455 | 0.026872 | 5 |
| GO:0033673 | negative regulation of kinase activity | 7/98 | 0.000487 | 0.028063 | 7 |
| GO:0035296 | regulation of tube diameter | 5/98 | 0.000527 | 0.028624 | 5 |
| GO:0097746 | blood vessel diameter maintenance | 5/98 | 0.000527 | 0.028624 | 5 |
| GO:0035150 | regulation of tube size | 5/98 | 0.000546 | 0.028624 | 5 |
| GO:0045667 | regulation of osteoblast differentiation | 5/98 | 0.000546 | 0.028624 | 5 |
| GO:0007178 | transmembrane receptor protein serine/threonine kinase signaling pathway | 8/98 | 0.000555 | 0.028624 | 8 |
| GO:0003012 | muscle system process | 9/98 | 0.000584 | 0.029376 | 9 |
| GO:1901889 | negative regulation of cell junction assembly | 3/98 | 0.000605 | 0.029376 | 3 |
| GO:0061041 | regulation of wound healing | 5/98 | 0.000606 | 0.029376 | 5 |
| GO:0055123 | digestive system development | 5/98 | 0.000672 | 0.031302 | 5 |
| GO:0090101 | negative regulation of transmembrane receptor protein serine/threonine kinase signaling pathway | 5/98 | 0.000672 | 0.031302 | 5 |
| GO:0010614 | negative regulation of cardiac muscle hypertrophy | 3/98 | 0.000724 | 0.033088 | 3 |
| GO:0048738 | cardiac muscle tissue development | 6/98 | 0.000759 | 0.034021 | 6 |
| GO:1903960 | negative regulation of anion transmembrane transport | 3/98 | 0.000789 | 0.034743 | 3 |
| GO:0014741 | negative regulation of muscle hypertrophy | 3/98 | 0.000857 | 0.035874 | 3 |
| GO:0044743 | protein transmembrane import into intracellular organelle | 3/98 | 0.000857 | 0.035874 | 3 |
| GO:0048511 | rhythmic process | 7/98 | 0.000859 | 0.035874 | 7 |
| GO:0051348 | negative regulation of transferase activity | 7/98 | 0.000894 | 0.036077 | 7 |
| GO:0090287 | regulation of cellular response to growth factor stimulus | 7/98 | 0.000894 | 0.036077 | 7 |
| GO:1903792 | negative regulation of anion transport | 6/98 | 0.000945 | 0.037523 | 6 |
| GO:0010810 | regulation of cell-substrate adhesion | 6/98 | 0.000968 | 0.037637 | 6 |
| GO:0016525 | negative regulation of angiogenesis | 5/98 | 0.000989 | 0.037637 | 5 |
| GO:0045429 | positive regulation of nitric oxide biosynthetic process | 3/98 | 0.001006 | 0.037637 | 3 |
| GO:0016311 | dephosphorylation | 9/98 | 0.001033 | 0.037637 | 9 |
| GO:0071900 | regulation of protein serine/threonine kinase activity | 9/98 | 0.001048 | 0.037637 | 9 |
| GO:2000181 | negative regulation of blood vessel morphogenesis | 5/98 | 0.001051 | 0.037637 | 5 |
| GO:1901343 | negative regulation of vasculature development | 5/98 | 0.001083 | 0.037637 | 5 |
| GO:1904407 | positive regulation of nitric oxide metabolic process | 3/98 | 0.001085 | 0.037637 | 3 |
| GO:0001649 | osteoblast differentiation | 6/98 | 0.001088 | 0.037637 | 6 |
| GO:0043405 | regulation of MAP kinase activity | 7/98 | 0.001105 | 0.037682 | 7 |
| GO:0001503 | ossification | 8/98 | 0.001157 | 0.038536 | 8 |
| GO:0048340 | paraxial mesoderm morphogenesis | 2/98 | 0.00117 | 0.038536 | 2 |
| GO:0019935 | cyclic-nucleotide-mediated signaling | 4/98 | 0.001193 | 0.038536 | 4 |
| GO:0032642 | regulation of chemokine production | 4/98 | 0.001193 | 0.038536 | 4 |
| GO:0030509 | BMP signaling pathway | 5/98 | 0.001254 | 0.039518 | 5 |
| GO:0035272 | exocrine system development | 3/98 | 0.001256 | 0.039518 | 3 |
| GO:0010594 | regulation of endothelial cell migration | 6/98 | 0.001303 | 0.040352 | 6 |
| GO:0043114 | regulation of vascular permeability | 3/98 | 0.001348 | 0.040352 | 3 |
| GO:0055017 | cardiac muscle tissue growth | 4/98 | 0.00135 | 0.040352 | 4 |
| GO:1901216 | positive regulation of neuron death | 4/98 | 0.00135 | 0.040352 | 4 |
| GO:0032306 | regulation of prostaglandin secretion | 2/98 | 0.001425 | 0.041134 | 2 |
| GO:0032308 | positive regulation of prostaglandin secretion | 2/98 | 0.001425 | 0.041134 | 2 |
| GO:0032309 | icosanoid secretion | 3/98 | 0.001444 | 0.041134 | 3 |
| GO:0035094 | response to nicotine | 3/98 | 0.001444 | 0.041134 | 3 |
| GO:0032602 | chemokine production | 4/98 | 0.001519 | 0.042792 | 4 |
| GO:0032496 | response to lipopolysaccharide | 7/98 | 0.001559 | 0.043411 | 7 |
| GO:0048512 | circadian behavior | 3/98 | 0.001647 | 0.044424 | 3 |
| GO:1903034 | regulation of response to wounding | 5/98 | 0.001656 | 0.044424 | 5 |
| GO:0061448 | connective tissue development | 6/98 | 0.001686 | 0.044424 | 6 |
| GO:2000344 | positive regulation of acrosome reaction | 2/98 | 0.001705 | 0.044424 | 2 |
| GO:0007622 | rhythmic behavior | 3/98 | 0.001756 | 0.044424 | 3 |
| GO:0003018 | vascular process in circulatory system | 6/98 | 0.001757 | 0.044424 | 6 |
| GO:0071772 | response to BMP | 5/98 | 0.001793 | 0.044424 | 5 |
| GO:0071773 | cellular response to BMP stimulus | 5/98 | 0.001793 | 0.044424 | 5 |
| GO:0045765 | regulation of angiogenesis | 7/98 | 0.00182 | 0.044424 | 7 |
| GO:0045833 | negative regulation of lipid metabolic process | 4/98 | 0.001835 | 0.044424 | 4 |
| GO:0060419 | heart growth | 4/98 | 0.001835 | 0.044424 | 4 |
| GO:0006633 | fatty acid biosynthetic process | 5/98 | 0.001841 | 0.044424 | 5 |
| GO:1904707 | positive regulation of vascular associated smooth muscle cell proliferation | 3/98 | 0.001869 | 0.044424 | 3 |
| GO:2000379 | positive regulation of reactive oxygen species metabolic process | 4/98 | 0.001903 | 0.044424 | 4 |
| GO:0090092 | regulation of transmembrane receptor protein serine/threonine kinase signaling pathway | 6/98 | 0.001985 | 0.044424 | 6 |
| GO:0030195 | negative regulation of blood coagulation | 3/98 | 0.001986 | 0.044424 | 3 |
| GO:2000191 | regulation of fatty acid transport | 3/98 | 0.001986 | 0.044424 | 3 |
[truncated: 32,922 more chars]
